# NCI9673 (Part B): ETCTN Randomized Phase II Study of Nivolumab With or Without Ipilimumab in Refractory, Metastatic Squamous Cell Carcinoma of the Anal Canal

**DOI:** 10.1200/JCO-25-00929

**Published:** 2026-01-07

**Authors:** Van K. Morris, Kristen K. Ciombor, Lianchun Xiao, Joshua K. Ochieng, Enrica Marmonti, Blase Polite, Benjamin A. Weinberg, John C. Krauss, John Hays, Sarbajit Mukherjee, Olivia Aranha, Syma Iqbal, Tony Shields, Al B. Benson, Syed Kazmi, Christopher Lieu, Howard Hochster, Jennifer Whisenant, Cara Haymaker, Cathy Eng

**Affiliations:** ^1^ University of Texas—MD Anderson Cancer Center, Houston, TX; ^2^ Vanderbilt-Ingram Cancer Center, Nashville, TN; ^3^ University of Chicago, Chicago, IL; ^4^ Georgetown University, Washington, DC; ^5^ University of Michigan, Ann Arbor, MI; ^6^ The Ohio State University, Columbus, OH; ^7^ Baptist Miami Cancer Institute, Miami, FL; ^8^ Washington University School of Medicine, St. Louis, MO; ^9^ University of Southern California, Los Angeles, CA; ^10^ Karmanos Cancer Institute, Detroit, MI; ^11^ Northwestern University, Chicago, IL; ^12^ The University of Texas Southwestern Medical Center, Dallas, TX; ^13^ University of Colorado, Aurora, CO; ^14^ Rutgers University, Newark, NJ

## Abstract

**PURPOSE:**

In the previously completed NCI9673 (part A) single-arm study, the antiprogrammed death (PD)–ligand-1 antibody nivolumab demonstrated efficacy for patients with metastatic anal cancer. In NCI9673 (Part B), we evaluated the anticytotoxic T-cell lymphocyte antigen-4 (CTLA-4) antibody ipilimumab in combination with nivolumab for patients with incurable anal cancer.

**METHODS:**

In this phase II NCI ETCTN trial, 100 patients with refractory, incurable anal cancer were randomly assigned to receive nivolumab (480 mg IV once every 4 weeks) alone or with ipilimumab (1 mg/kg IV once every 8 weeks). The primary end point was progression-free survival (PFS). Secondary endpoints included radiographic response, overall survival (OS), and grade ≥3 adverse events. A log-rank test was used to compare survival between arms, with a one-sided alpha of 0.1 and power of 90%. Immune biomarkers were analyzed from baseline and on treatment tissue and blood collections.

**RESULTS:**

The median PFS for nivolumab versus nivolumab plus ipilimumab were 2.9 months (90% CI, 1.9 to 3.8) and 3.7 months (90% CI, 2.0 to 5.6), respectively (hazard ratio [HR], 0.86 [95% CI, 0.60 to 1.23]; *P* = .25). Response rates were similar for nivolumab (17.4%) and nivolumab plus ipilimumab (21.5%; *P* = .89). The median OS was 15.9 months for nivolumab and 20.0 months for nivolumab plus ipilimumab (HR, 0.98 [90% CI, 0.63 to 1.51]). Grade ≥3 treatment-related AEs occurred in 6 patients (12%) receiving nivolumab alone and in 12 patients (25%) receiving nivolumab plus ipilimumab. At week 9, circulating TIGIT+ CD8^+^ cells (*P* < .001) increased with nivolumab plus ipilimumab treatment relative to baseline.

**CONCLUSION:**

The addition of ipilimumab to nivolumab did not statistically improve overall response rate, PFS, or OS but may harbor increased toxicity. Paired blood samples identified TIGIT expression on peripheral T cells as a compensatory change unique to dual PD-1 plus CTLA-4 blockade.

## INTRODUCTION

The incidence of squamous cell carcinoma of the anal canal continues to rise in the United States, with nearly 11,000 new diagnoses anticipated in 2025.^1^ More than 90% of anal cancers are associated with the human papillomavirus (HPV)^2,3^ infection. Over recent decades, there has been an alarming trend toward a more advanced stage of disease at the time of initial presentation for anal cancer.^4^ In the first line of treatment, cytotoxic chemotherapy leads to responses in 55%-86% of patients with metastatic, incurable anal cancer.^5-7^ Nonetheless, the 5-year survival rate for metastatic anal cancer is <40%, and novel therapies that improve survival outcomes remain an outstanding need for this patient population.

**CONTEXT**

**Key Objective**
Does cytotoxic T-cell lymphocyte antigen-4 blockade improve outcomes when added to antiprogrammed death-1 therapy for patients with previously treated metastatic anal cancer?
**Knowledge Generated**
No differences in response rate or progression-free survival between nivolumab along or in combination with ipilimumab were observed for treatment of metastatic anal cancer. Increases in TIGIT expression on circulating T cells following dual immune checkpoint blockade may implicate TIGIT as a relevant immunotherapy target for future studies in this patient population.
**Relevance *(E.M. O'Reilly)***
Single agent or dual immune checkpoint blockade has modest activity in previously treated squamous cell cancer of the anus. New immunomodulatory and/or other therapeutic directions are needed in this rare malignancy.*
*Relevance section written by *JCO* Associate Editor Eileen M. O'Reilly, MD, FASCO.

Antiprogrammed death (PD)–ligand (L)-1 antibodies have demonstrated modest treatment efficacy for patients with metastatic anal cancer treated with prior chemotherapy. The NCI 9673 (part A) phase II trial evaluated nivolumab as anti–PD-1 monotherapy for patients with treatment-refractory, incurable anal cancer,^8^ with an overall response rate (ORR) of 24%, and the median progression-free survival (PFS) of 4.1 months. Subsequent studies evaluating other anti–PD-(L)1 antibodies in this setting reported similar response rates between 11% and 24%.^9-11^

Immune activation signatures have been linked to antitumor efficacy with immune checkpoint blockade for patients with metastatic anal cancer.^12-14^ The benefit of adding an anticytotoxic T-cell lymphocyte antigen-4 (CTLA-4) antibody to anti–PD-(L)1 therapies has not been evaluated in this setting. With the goal of promoting more durable survival for these patients, we conducted NCI9673 (part B), a randomized phase II trial of nivolumab with or without ipilimumab for patients with treatment-refractory metastatic anal cancer.

## METHODS

### Study Design and Study Participants

NCI9673 (part B) was a multi-institutional, randomized phase II trial comparing nivolumab versus nivolumab plus ipilimumab that was conducted across the Experimental Therapeutic Clinical Trials Network (ETCTN) in the United States. All participants had squamous cell carcinoma of the anus/rectum that was metastatic and/or locally advanced/unresectable following prior chemoradiation and radiographically measurable according to RECIST 1.1 criteria. HPV testing and PD-L1 expression status of pretreatment tumor tissue were not required. Study participants must have received at least one prior systemic therapy for incurable disease or new metastatic disease within 6 months of chemoradiation completion for localized anal cancer. No prior immunotherapy was permitted. Other relevant eligibility criteria are detailed in the study protocol. Participants living with HIV were eligible if their HIV viral load was undetectable, and CD4 count was ≥300/μL on antiretroviral therapy. The trial (ClinicalTrials.gov identifier: NCT02314169) was approved for research conduct at all participating institutions by a central Institutional Review Board. All participants provided informed consent before study entry. All study activity for this clinical trial was conducted in accordance with the Declaration of Helsinki. Amendments for this protocol are detailed in Data Supplement, Appendix 1, online only.

### Study Treatment

Participants were equally randomly assigned to receive nivolumab (480 mg) intravenously once every 4 weeks (Arm A) or in combination with intravenous ipilimumab (1 mg/kg) once every 8 weeks (Arm B). Adverse events were assessed according to CTCAE version 4.0. Study participants experiencing grade 2 or higher adverse events were required to hold treatment until improvement to grade 1 toxicity and with corticosteroid administration for a grade 3 or 4 treatment-related adverse event.

Treatment response was evaluated via independent assessment by computed tomography or magnetic resonance imaging every 8 weeks according to RECIST 1.1 criteria.^15^ Participants were allowed to continue study treatment until the development of clinical or radiographic progression, unacceptable toxicity, pregnancy, dosing delay beyond 6 weeks, or withdrawal of consent. Treatment beyond progression within the first 12 weeks of study treatment was permitted provided that the sum of target lesions did not exceed >40% baseline measurements, that the participant as otherwise clinically stable, and that a follow-up restaging 4-8 weeks later did not demonstrate further progressive disease. No crossover from Arm A to Arm B was allowed.

### Outcomes

The primary end point was PFS, calculated as the time between registration and progression or death (whichever occurred first) or the date of last follow-up (if alive without progression). Secondary end points included overall survival (OS), best radiographic response, and grade ≥3 adverse events. OS was calculated as the time between registration and death or last follow-up (if still alive at data cutoff).

The null hypothesis was that there would be no significant difference in PFS between the two arms. The alternative hypothesis was that combination of nivolumab + ipilimumab would improve PFS relative to nivolumab alone. With an expected median PFS time for nivolumab arm of 4 months based on the precedent NCI9673 (part A) study, we postulated an improvement in median PFS to 7 months with combination immunotherapy. With this assumption, a total of 87 events would have 90% power to detect this difference in median PFS between treatment arms at a one-sided alpha of 0.1.

The Kaplan-Meier method was used to estimate median PFS and OS (along with 90% CI). The log-rank test was used to compare survival times between treatment groups, and Cox proportional hazards regression analyses were used to estimate hazard ratios (HR). A one-sided log rank test was used to compare PFS between the treatment arms. All other tests were two-sided.

For patient characteristics, categorical variables were tabulated with frequency and percentage and compared using the Chi-square test or Fisher exact test while continuous variables were summarized with descriptive statistics and compared using the Wilcoxon rank sum test. Response rate and disease control rate were estimated along with the 95% CI and compared using the Chi-square test or Fisher exact test.

### Flow Cytometry

Fresh tumor tissue from 16 patients (baseline n = 11; week 9 [n = 7] with two paired samples) was available for flow cytometry analysis. Flow cytometry analysis was conducted on cryopreserved peripheral blood mononuclear cells (PBMCs) collected and processed at baseline (n = 90), week 9 (n = 53), and at end of treatment (EOT, n = 27) from a total of 96 patients (Data Supplement, Table S1). Further details of tissue and PBMC preparation and analyses are described in Data Supplement, Appendix 2.

## RESULTS

### Clinical Outcomes

**FIG 1.**
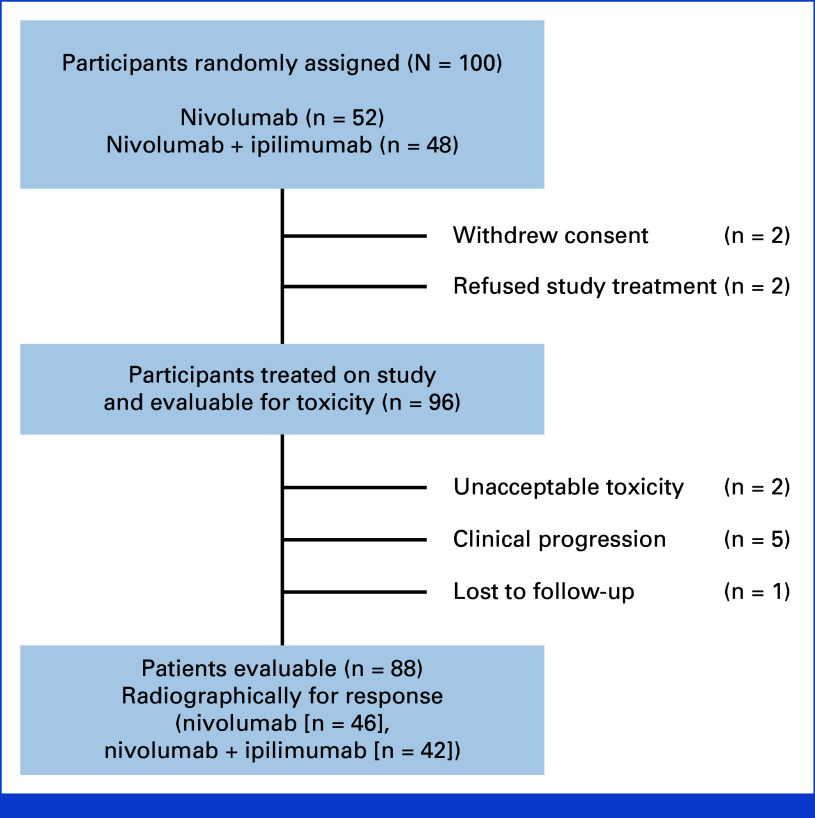
CONSORT diagram.

One-hundred participants received at least one dose of study treatment between October 2018 through May 2024 (Fig 1): 52 patients with nivolumab alone and 48 with nivolumab plus ipilimumab. As seen in Table 1, the majority of participants were female (77%) and Caucasian (96%). The mean ages were 60 years in both arms. No study participants had a diagnosis of HIV.

**TABLE 1.** Demographics According to Treatment Arm

Demographic	Nivolumab, No. (%; n = 52)	Nivolumab + Ipilimumab, No. (%; n = 48)
Sex		
Female	41 (79)	36 (75)
Male	11 (21)	12 (25)
Race		
Asian	1 (2)	0 (0)
African American	1 (2)	3 (6)
Caucasian	50 (96)	45 (94)
ECOG performance status		
0	25 (48)	28 (58)
1	27 (52)	20 (42)
Age (years; mean ± SD)	60 ± 8.8	60.6 ± 10.1

Abbreviations: ECOG, Eastern Cooperative Oncology Group; SD, standard deviation.

**FIG 2.**
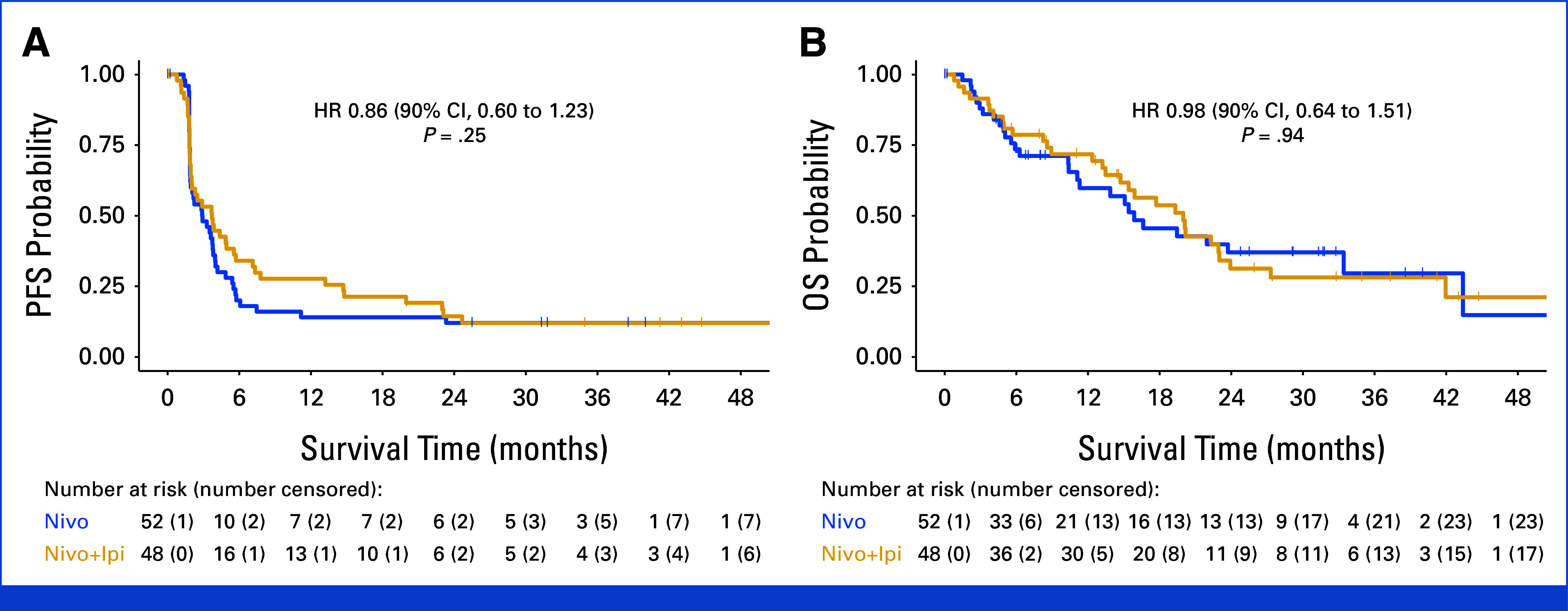
(A) PFS and (B) OS. HR, hazard ratio; OS, overall survival; PFS, progression-free survival.

The date for data cutoff was June 18, 2024. The median follow-up time was 31.3 months (95% CI, 25.9 to 37.3), during which time PFS events occurred in 85 participants. As seen in Figure 2A, the median PFS was 2.9 months (90% CI, 1.9 to 3.8) with nivolumab alone, and 3.7 months (90% CI, 2.0 to 5.6) for nivolumab plus ipilimumab (one-side log-rank test *P* = .25). No significant improvement in PFS was observed by the addition of ipilimumab to nivolumab (HR, 0.86 [90% CI, 0.60 to 1.23]). There were no differences in median PFS between treatment arms according to age, sex, race/ethnicity, or baseline Eastern Cooperative Oncology Group (ECOG) performance status (Data Supplement, Table S2). The median PFS for all study participants across both treatment arms was estimated at 3.3 months (90% CI, 2.2 to 3.9). The estimated 6-month PFS rates were 20% (90% CI, 13 to 32) and 34% (90% CI, 24 to 48) for nivolumab and nivolumab plus ipilimumab, respectively.

OS is shown in Figure 2B. No difference in OS was observed for nivolumab alone (15.9 months [90% CI, 11.3 to 33.4]) versus nivolumab and ipilimumab (20.0 months [90% CI, 15.4 to 23]; HR, 0.98 [90% CI, 0.64 to 1.51]). OS rates at 1 year and at 2 years with nivolumab were 59.8% (90% CI, 48.5 to 73.6) and 37.0% (90% CI, 26.1 to 52.5), respectively, and for the combination arm were 71.8% (90% CI, 61.6 to 83.6) and 31.3% (90% CI, 21.0 to 46.5), respectively. In a post hoc exploratory analysis, we observed no association between OS, age, sex, ECOG PS, or race/ethnicity (Data Supplement, Table S3).

**FIG 3.**
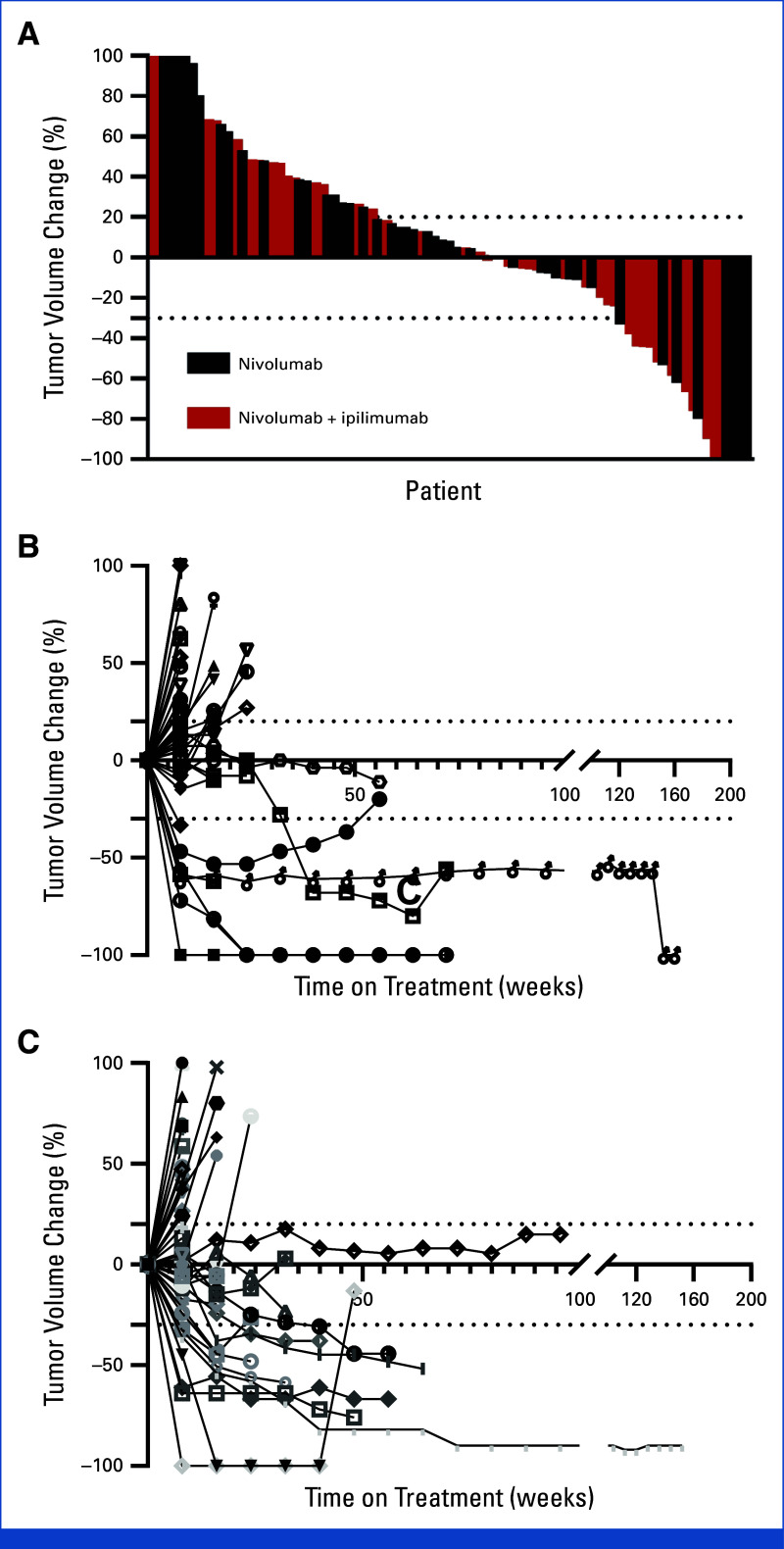
(A) Waterfall plot; spider plots for treatment with (B) nivolumab alone and with (C) nivolumab plus ipilimumab.

Among 88 participants evaluable for response (nivolumab: 46; nivolumab plus ipilimumab: 42; Fig 1), the ORR was 19.3% (Fig 3A). The ORR was 17.4% (95% CI, 9.1 to 31) for nivolumab and 21.5% (95% CI, 12 to 36) for nivolumab plus ipilimumab. No difference in ORR was measured between treatment arms (*P* = .89). There were three patients who achieved a complete radiographic response with nivolumab and two patients with nivolumab plus ipilimumab. As shown by the spider plots in Figures 3B and 3C, those with a complete response experienced sustained treatment benefit on study. Disease control rates for nivolumab alone and for nivolumab plus ipilimumab were 43.5% (95% CI, 30 to 58) and 47.6% (95% CI, 33 to 62), respectively. There were five participants (nivolumab: 2; nivolumab + ipilimumab: 3) treated beyond progression as defined in the study protocol, all with confirmed disease progression at the subsequent restaging.

The majority of participants (n = 97) experienced a treatment-related adverse event (Data Supplement, Table S4). The most notable was a grade 5 pneumonitis related to nivolumab plus ipilimumab, following a complete radiographic response. No grade 4 or 5 events related to nivolumab monotherapy were observed. Grades ≥3 treatment-related adverse events (Table 2) occurring in multiple study participants included pneumonitis (n = 4), hyperglycemia (n = 3), hyponatremia (n = 2), and elevated ALT (n = 2). Of the three patients who experienced grade 4 hyperglycemia, two events were new-onset type 1 diabetes mellitus requiring initiation of insulin therapy and attributed as probably related to nivolumab + ipilimumab. The third patient had type 2 diabetes at baseline, with transient grade 4 hyperglycemia not attributed to study treatment. The most common overall treatment-related adverse events (all grades) were fatigue (22%), diarrhea (13%), anemia (12%), nausea (12%), hypothyroidism (11%), anorexia (10%), and maculopapular rash (10%).

**TABLE 2.** Grade ≥3 Adverse Events According to Treatment Arm

Adverse Event	Nivolumab	Nivolumab + Ipilimumab			Total
Grade (No.)	3	3	4	5	
Pneumonitis	0	3	0	1	4
Hyperglycemia	0	0	3	0	3
Hyponatremia	2	1	0	0	3
ALT increased	0	2	0	0	2
Abdominal distension	1	0	0	0	1
Abdominal pain	0	1	0	0	1
Adrenal insufficiency	0	1	0	0	1
Anemia	0	1	0	0	1
AST increased	0	1	0	0	1
Blood bilirubin increased	0	1	0	0	1
Fatigue	0	1	0	0	1
Hypokalemia	0	1	0	0	1
Hypophosphatemia	1	0	0	0	1
Hypophysitis	0	1	0	0	1
Lipase increased	1	0	0	0	1
Lymphocyte count decreased	0	1	0	0	1
Malaise	0	1	0	0	1
Minimal change disease/nephrotic syndrome	1	0	0	0	1
Rash maculopapular	1	0	0	0	1
Vomiting	0	1	0	0	1

### Circulating Immune Activation and Inhibitory States Early On-Therapy Differ by Arm

**FIG 4.**
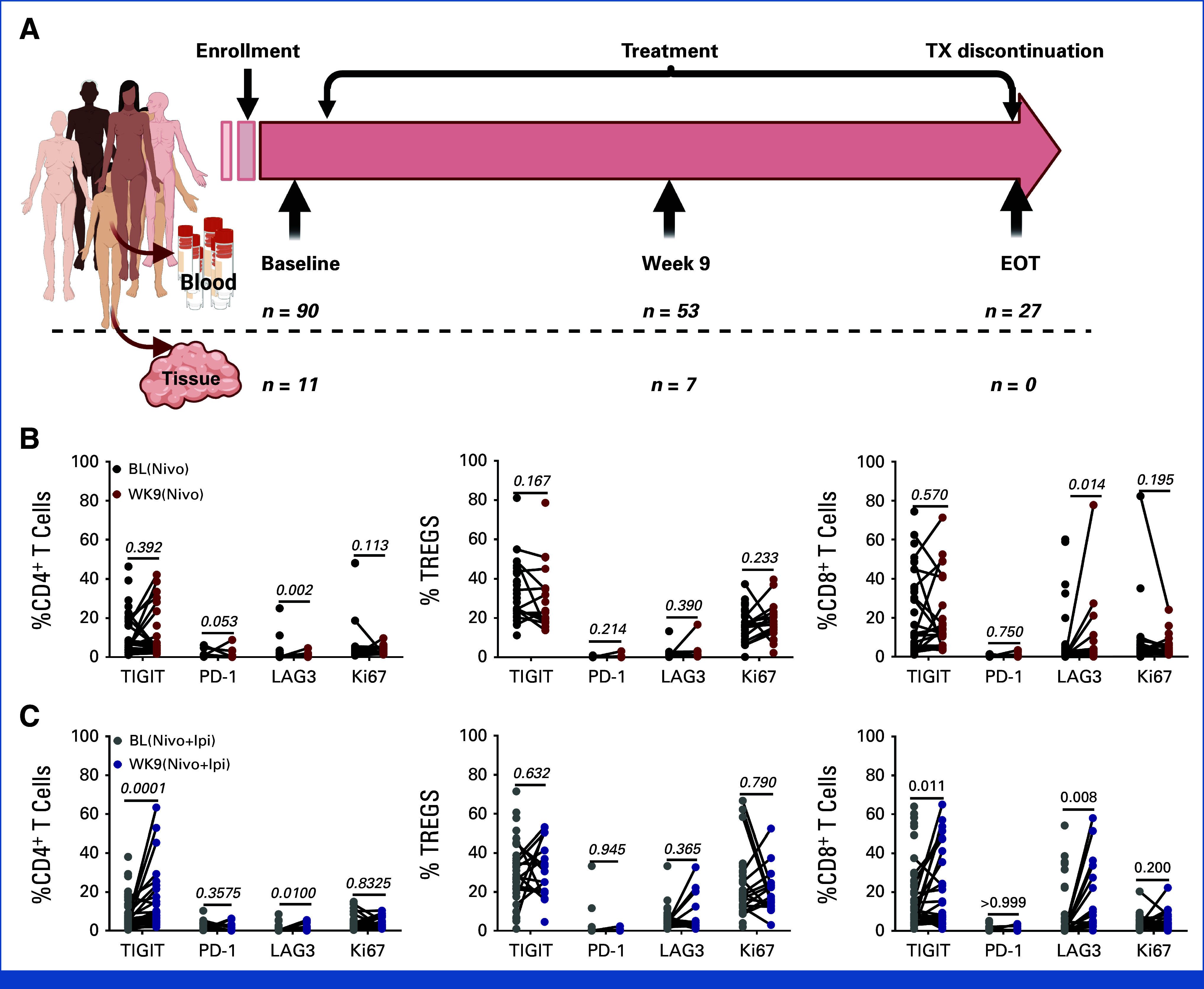
(A) Graphical representation of blood and tissue collections for translational research. The number (n) of samples per time point are shown for each time point. (B-C) Expression of assessed markers at BL and week 9 (W9) on CD4^+^ T cells (left), Tregs (middle) and CD8^+^ T cells (right) by arm (nivolumab, red, BB and nivolumab + ipilimumab, blue, CC). A QC threshold of 100 events was required for each level of subgating. In the nivolumab arm (CD4^+^ T cells, n = 36 BL, n = 21 W9; Tregs, n = 29 BL, n = 21 W9; CD8^+^ T cells n = 36 BL, n = 22 W9) and in the nivolumab + ipilimumab arm (CD4^+^ T cells, n = 41 BL, n = 27 W9; Tregs n = 40 BL, n = 27 W9; CD8^+^ T cells, n = 41 BL, n = 27 W9). Statistical significance was determined using a Wilcoxon signed-rank test. BL, baseline; EOT, end of treatment; QC, quality control; TREGS, T regulatory cells; TX, treatment.

To interrogate the impact of single-agent nivolumab or the combination of nivolumab + ipilimumab on the local tumor immune microenvironment and in circulation, longitudinally collected tumor biopsies and PBMCs were assessed using high dimensional flow cytometry (Fig 4A; Data Supplement, Fig S1). From a total of 16 patients, tumor biopsies were collected at baseline and W9 on-treatment with paired samples available for analysis from 44 patients treated with nivolumab alone and 33 patients treated with the combination of nivolumab plus ipilimumab. In both arms, the frequency of CD8^+^ T cells in the tumor tissue was higher than the CD4^+^ T-cell or NK cell counterparts (Data Supplement, Fig S2A). Expression of activation markers (OX40, ICOS), checkpoint receptors (TIGIT, Tim3, PD-1, LAG3, CTLA4), and proliferation (Ki67) were not statistically changed. However, PD-1 expression was reduced, as expected, in all patients at W9 in both arms as a result of nivolumab blocking the assay epitope (Data Supplement, Fig S2B).

We applied the same strategy to profiling longitudinal changes in circulating T-cell and NK cell subsets. As shown in the Data Supplement (Fig S2C), CD8^+^ T cells and NK cells were present at higher frequencies at all time points as compared with the CD4^+^ T cells and T regulatory cells (Tregs; defined as CD3^+^CD4^+^CD25+FoxP3+). In both arms, by W9, reduction in the CD8:Treg ratio suggesting either CD8^+^ T-cell attrition or Treg expansion, but this change was not statically significant (Data Supplement, Fig S2D). Conversely, unique changes in activation markers and checkpoint receptors were identified by W9 that differed by arm and cell type. In the nivolumab arm, the percentage of CD4^+^ T cells expressing LAG3, Tim3, or CTLA-4 was significantly higher at W9 (*P* = .002, *P* = .0054, *P* = .000404, respectively; Fig 4B, red [nivolumab] and Data Supplement [Fig S2E, red, nivolumab]). On the other hand, expression of TIGIT or LAG3 on CD4^+^ T cells was significantly higher at W9 in the nivolumab plus ipilimumab arm (*P* = .0001 and .01, respectively; Fig 4C, blue, nivolumab + ipilimumab). There were no phenotypic changes within the Treg subset based upon the markers assessed in either arm. LAG3 expression was also significantly upregulated on the circulating CD8^+^ T cells by W9 in both arms (*P* = .014 [red, nivolumab] and *P* = .008 [blue, nivolumab + ipilimumab]; Figs 4B and 4C) while TIGIT was upregulated only in the nivolumab plus ipilimumab arm (*P* = .011) and CTLA-4 only in the nivolumab arm (*P* = .0399399) in this subset (Fig 4C, blue, nivolumab + ipilimumab and Data Supplement, Fig S2E, red, nivolumab). NK cell phenotypic modulation was observed on in the nivolumab plus ipilimumab arm with OX40 (*P* = .041) and CTLA-4 (*P* = .0033) upregulation at W9 (Data Supplement, Fig S2F, blue, nivolumab + ipilimumab).

### Combination Therapy Induces the Proliferation of TIGIT+CD8^+^ T Cells in Circulation

**FIG 5.**
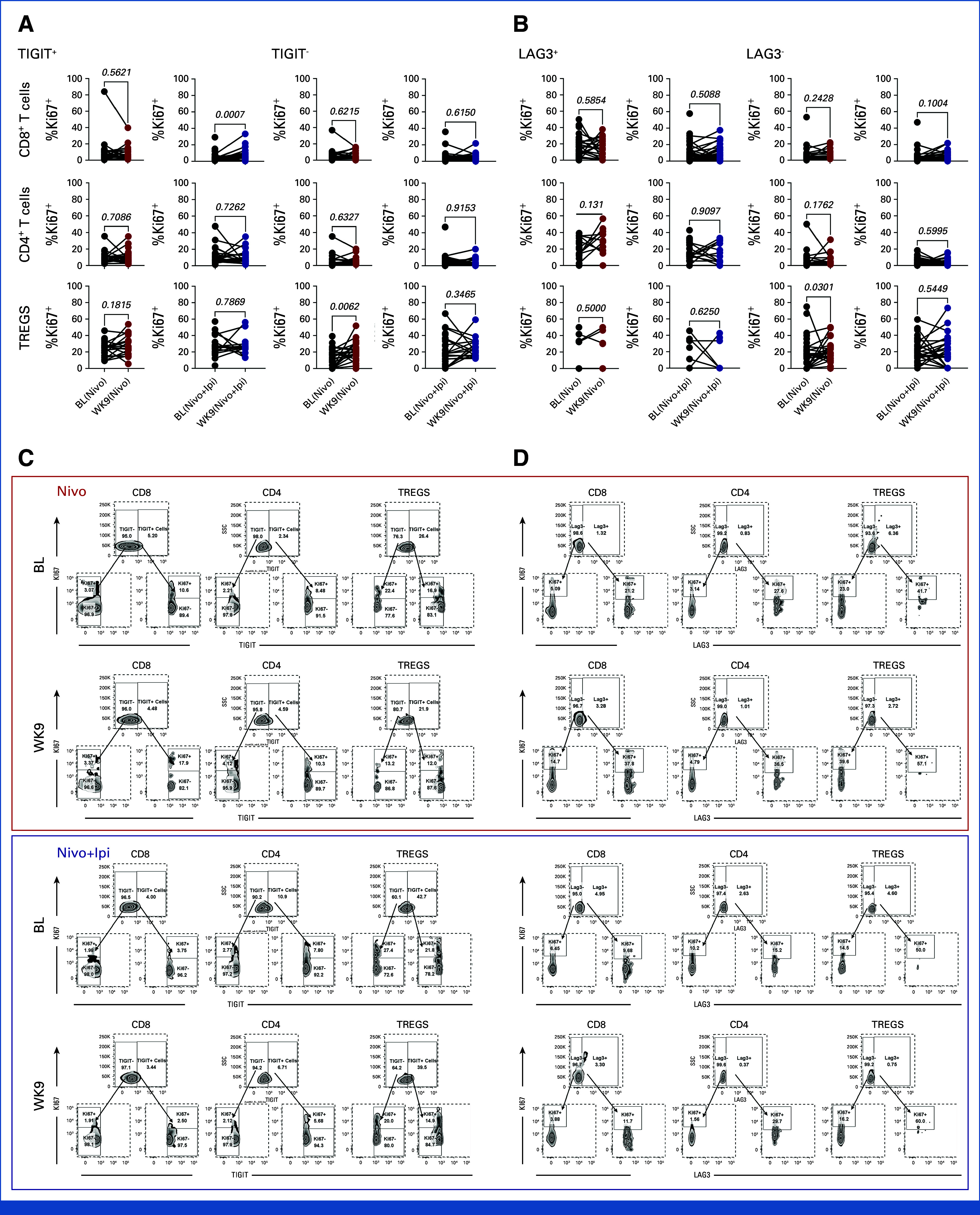
(A) T-cell subsets were subdivided into TIGIT+ and TIGIT– compartments and assessed for Ki67 expression as a proliferation marker. Expression was assessed at BL and Week 9 (W9) for CD4^+^ T cells (top row), Tregs (middle row) and CD8^+^ T cells (bottom row) by arm (nivolumab, red; nivolumab + ipilimumab, blue) In the nivolumab arm (CD8+TIGIT+ T cells, n = 32 BL, n = 22 W9; CD8+TIGIT– T cells, n = 34 BL, n = 22 W9; CD4+TIGIT+ T cells, n = 31 BL, n = 21 W9; CD4+TIGIT– T cells, n = 32 BL and n = 21 W9; TIGIT + Tregs, n = 26 BL, n = 18 W9; TIGIT-Tregs, n = 23 BL, n = 21 W9) and in the nivolumab + ipilimumab arm (CD8+TIGIT+ T cells, n = 34 BL, n = 23 W9; CD8+TIGIT– T cells, n = 35 BL, n = 22 W9; CD4+TIGIT+ T cells, n = 34 BL, n = 23 W9; CD4+TIGIT– T cells, n = 38 BL and n = 24 W9; TIGIT + Tregs, n = 24 BL, n = 15 W9; TIGIT-Tregs, n = 28 BL, n = 18 W9). (B) T-cell subsets were subdivided into LAG3+ and LAG3-compartments and assessed for Ki67 expression as a proliferation marker. Expression was assessed at baseline (BL) and week 9 (W9) for CD88^+^ T cells (top row), CD4^+^ T cells (middle row), and Tregs (bottom row) by arm (nivolumab, red; nivolumab + ipilimumab, blue) In the nivolumab arm (CD8+LAG3+ T cells, n = 34 BL, n = 22 W9; CD8^+^ LAG3– T cells, n = 36 BL, n = 20 W9; CD4^+^ LAG3+ T cells, n = 21 BL, n = 11 W9; CD4^+^ LAG3– T cells, n = 21 BL and n = 11 W9; LAG3+ Tregs, n = 5 BL, n = 5 W9; LAG3-Tregs, n = 28 BL, n = 21 W9) and in the nivolumab + ipilimumab arm (CD8^+^ LAG3+ T cells, n = 32 BL, n = 18 W9; CD8^+^ LAG3– T cells, n = 32 BL, n = 22 W9; CD4^+^ LAG3+ T cells, n = 18 BL, n = 10 W9; CD4^+^ LAG3– T cells, n = 33 BL and n = 22 W9; LAG3+ Tregs, n = 5 BL, n = 3 W9; LAG3-Tregs, n = 32 BL, n = 19 W9). (C) Representative zebra plots of each T-cell subset by arm (nivolumab, red; nivolumab + ipilimumab, blue) for TIGIT expression followed by Ki67 subgating. (D) Representative zebra plots of each T-cell subset by arm (nivolumab, red; nivolumab + ipilimumab, blue) for LAG3 expression followed by Ki67 subgating. A QC threshold of 100 events was required for each level of subgating. Statistical analysis performed using a Wilcoxon signed-rank test. BL, baseline; QC, quality control; TREGS, T regulatory cells.

Given the dynamic induction of TIGIT and LAG3 expression on CD4^+^ and CD8^+^ T cells in this study and the potential role of these receptors as phenotypic markers of T-cell exhaustion, we investigated whether nivolumab versus nivolumab plus ipilimumab drove a differential impact on the proliferation of each subset over time. To evaluate this, T-cell subsets were gated into TIGIT+ versus TIGIT– (Fig 5A) and LAG3+ versus LAG3– (Fig 5B) compartments and stratified by arm (nivolumab, red; nivolumab + ipilimumab, blue). Interestingly, although TIGIT expression on CD8^+^ T cells was increased in both arms, only the combination of nivolumab plus ipilimumab showed an increase in the proliferation of this subset at W9 relative to baseline (*P* = .0007; Fig 5A, top row, blue, nivolumab + ipilimumab). There was no differential ability of nivolumab alone or with ipilimumab to induce proliferation of CD4^+^ T cells regardless of TIGIT or LAG3 expression, or lack thereof (Figs 5A and 5B, middle row). Although the impact of either treatment strategy on Treg subsets was limited because of the low frequencies detected in circulation and low expression of LAG3 within the subset, proliferation of the TIGIT– and LAG3– subset was increased in the nivolumab arm only by W9 (*P* = .0062 and *P* = .0301, respectively, Figs 5A and 5B, bottom row, red, nivolumab). Representative plots for each cell type by time point and arm are shown for TIGIT subsets in Figure 4C and LAG3 subsets in Figure 5D.

## DISCUSSION

In this randomized phase II trial, addition of ipilimumab to nivolumab did not improve PFS compared with nivolumab alone in patients with treatment-refractory, unresectable/metastatic anal cancer. Treatment-related adverse events with nivolumab and ipilimumab appeared to be more significant than with nivolumab alone. We observed that over one third of participants received combination immunotherapy beyond the median PFS of 3.3 months, with a 34% 6-month PFS rate.

Findings reported here are similar for both arms to previous single-arm trials demonstrating antitumor efficacy in the same population of patients treated with anti–PD-(L)1 monotherapies.^5,8,9,11^ In those studies, the median PFS ranged between 2 and 4 months, consistent with those from nivolumab (2.9 months) and nivolumab plus ipilimumab (3.7 months) seen in NCI9673 (part B). On the basis of our results reported here, it does not appear that anti-CTLA4 blockade will meaningfully improve survival in the clinical management for most patients with incurable anal cancer. A phase III trial (POD1UM-303) evaluating chemotherapy with carboplatin/paclitaxel alone or in combination with the anti–PD-1 antibody retifanlimab in patients with newly diagnosed, previously untreated metastatic anal cancer demonstrated improved PFS with the chemoimmunotherapy combination.^16^ In that study, patients randomly assigned to receive carboplatin, paclitaxel, and retifanlimab who had disease control at 6 months continued with single-agent retifanlimab maintenance for up to 2 years. The median PFS was 9.3 months for that group, a result which affirms that anti–PD-1 monotherapy offers short-lived survival prolongation following cessation of chemotherapy in patients with metastatic anal cancer. EA2176 (ClinicalTrials.gov identifier: NCT04444921) is an National Cancer Institute (NCI)-supported phase III trial, now completely enrolled, evaluating carboplatin/paclitaxel ± nivolumab followed by maintenance nivolumab in treatment-naïve metastatic anal cancer patients. If EA2176 also fulfills its primary end point, the outcomes of these trials collectively support that, at present, anti–PD-1 therapy is best used with chemotherapy in the frontline setting and as monotherapy for treatment-refractory disease if no prior immunotherapy exposure.^17^

Patient-specific responses to immune checkpoint blockade were observed in tissue, although paired biopsies adequate for analysis were obtained from only five study participants. Immune modulation within the tumor microenvironment occurred for both treatment groups, with an increase in cytotoxic T cells following immunotherapy relative to changes in CD4^+^ T-cell and NK cell populations within the biopsied specimens. We did not observe any compensatory biomarker changes, including CTLA4, within the tumor microenvironment. One previous study has evaluated paired biopsies for a similar population of 20 patients with refractory metastatic anal cancer treated with immunotherapy with the combination of the anti–PD-L1 antibody atezolizumab and the anti-VEGF antibody bevacizumab.^18^ Prolonged PFS was linked with transcriptomic increases in immune activation signatures.^19^ Confirmation of those findings in our larger, multicenter study was limited by a low yield of paired biopsies that were amenable to flow cytometry.

One novelty to our translational studies is the differential patterns of immune modulation occurring in the peripheral circulation between treatment arms. Such an analysis has been unable to be performed previously since, to our knowledge, this is the first randomized trial to evaluate different immunotherapies directly for metastatic anal cancer. Relative to baseline, treatment with nivolumab and ipilimumab resulted in an increase of TIGIT^+^ CD8^+^ T cells and TIGIT^+^ CD4^+^ T cells within the blood at week 9, a trend not observed with nivolumab alone. Increases in proliferation of TIGIT^−^ CD8^+^ T cells and TIGIT^−^ CD4^+^ T cells alike were not observed following treatment with nivolumab and ipilimumab. Collectively, these findings suggest that compensatory immune-suppressing mechanisms via TIGIT may occur preferentially in response to dual immune checkpoint blockade, which could account for the lack of improvement in clinical outcomes for nivolumab plus ipilimumab reported here. Increased expression of TIGIT has been linked to inferior survival in other HPV-associated cancers.^20,21^ The clinical relevance of our translational findings suggesting TIGIT as an actionable target here remains hypothesis generating in light of the lack of positive outcomes in our study to satisfy its primary objective. Targeting TIGIT therefore may be a future strategy of interest for overcoming resistance to immune checkpoint blockade against PD-1 and CTLA-4 for patients with metastatic anal cancer.

Several limitations to interpretation of our findings should be considered. One limitation is that we were unable to collect fresh tissue biopsies from study participants across all sites, as many of the registrations occurred during the COVID-19 pandemic. Therefore, we were unable to assess broadly baseline tumor characteristics like PD-L1 expression in relation to response to immunotherapy. We acknowledge that these translational findings are exploratory and hypothesis generating toward new treatment approaches in the future. In another limitation, the median PFS was under 4 months, yet median OS exceeded 15 months for both arms of this treatment-refractory population. It is likely that most participants then received additional therapies after completion of study treatment. We do not have data available regarding subsequent treatment courses, which, if available, could have provided insights into outcomes with cytotoxic chemotherapy after progression of immunotherapy as treatment for metastatic anal cancer. In addition, that no study participants were living with HIV may reflect that the demographics of our trial may not be reflective of the broader population of patients in the United States who are diagnosed with anal cancer, since HIV is a risk factor for development of this malignancy.

In summary, the addition of the CTLA4 antibody ipilimumab did not statistically improve progression-free and OS relative to anti–PD-1 therapy alone for patients with previously treated metastatic anal cancer. To date, to our knowledge, this is the largest randomized, prospective clinical trial to directly compare different immunotherapy regimens for patients with this rare cancer. Treatment options continue to remain limited, and this highlights the ongoing unmet need for additional studies in this patient population.

## Data Availability

A data sharing statement provided by the authors is available with this article at DOI https://doi.org/10.1200/JCO-25-00929.
