# Supplementary material for: NCI9673 (Part B): ETCTN Randomized Phase II Study of Nivolumab With or Without Ipilimumab in Refractory, Metastatic Squamous Cell Carcinoma of the Anal Canal
Source: J Clin Oncol. 2026 Jan 7;44(6):497–507. doi: 10.1200/JCO-25-00929 (PMC12782278; doi:10.1200/JCO-25-00929)
Supplement: Supplementary file 2 [file jco-44-497-s002.pdf]

Supplementary Figure S1. Gating strategy for flow cytometry analysis of tumor tissue and PBMCs.

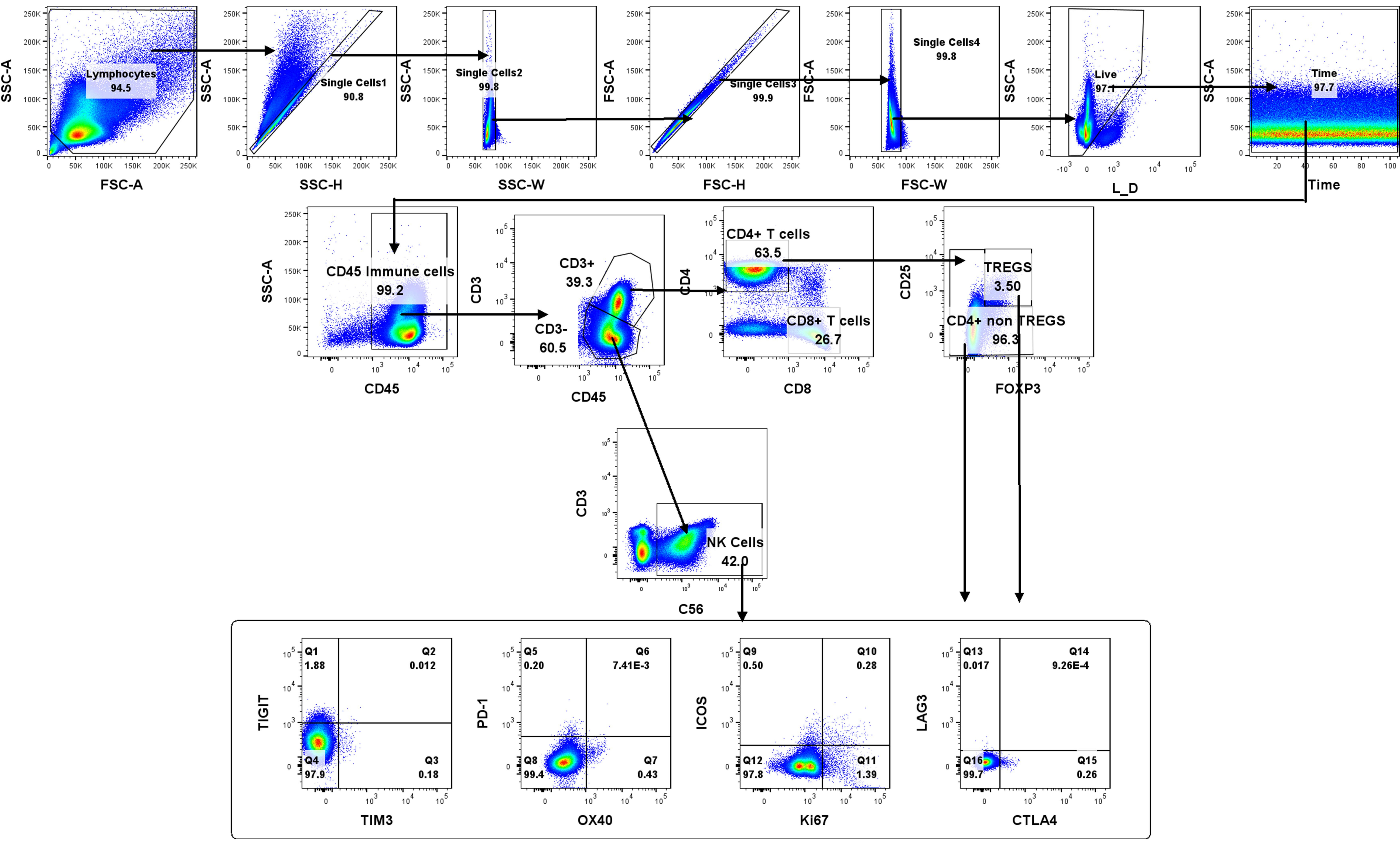

Supplementary Figure S2. (A). Radial plot illustrating immune cell populations in tissues stratified by arm (nivolumab ipilimumab combination, Nivo+Ipi and nivolumab, Nivo) (B). Frequencies of assessed biomarkers at baseline and WK9 from tumor biopsies by arm (Nivo, red, top row and Nivo+Ipi, blue, bottom row) Paired samples are indicated by the line connecting the timepoints per patient (C). A radial plot showing the frequency of immunophenotypes overlapping between the treatment arms in circulation. (D) Ratio of CD8+ T cells to Treg cells in circulation over time stratified by arm. (E) Expression of checkpoint receptors on CD4+ T cells (left), Tregs (middle) and CD8+ T cells (right) stratified by arm (Nivo, red; Nivo+Ipi, blue) in circulation. (F) Expression of checkpoint receptors on NK cells stratified by arm (Nivo, red; Nivo+Ipi, blue) in circulation. A QC threshold of 100 events was required for each level of subgating. In the Nivo arm [CD4+ T cells, N=36 BL, N=21 W9; Tregs, N=29 BL, N=21 W9; CD8+ T cells N=36 BL, N=22 W9; NK cells, N=36 BL, N=21 W9] and in the Nivo+Ipi arm [CD4+ T cells, N=41 BL, N=27 W9; Tregs N=40 BL, N=27 W9; CD8+ T cells, N=41 BL, N=27 W9; NK cells, N=36 BL, N=26 W9]. Statistical analysis performed using a Wilcoxon signed-rank test.

Supplementary Figure S2

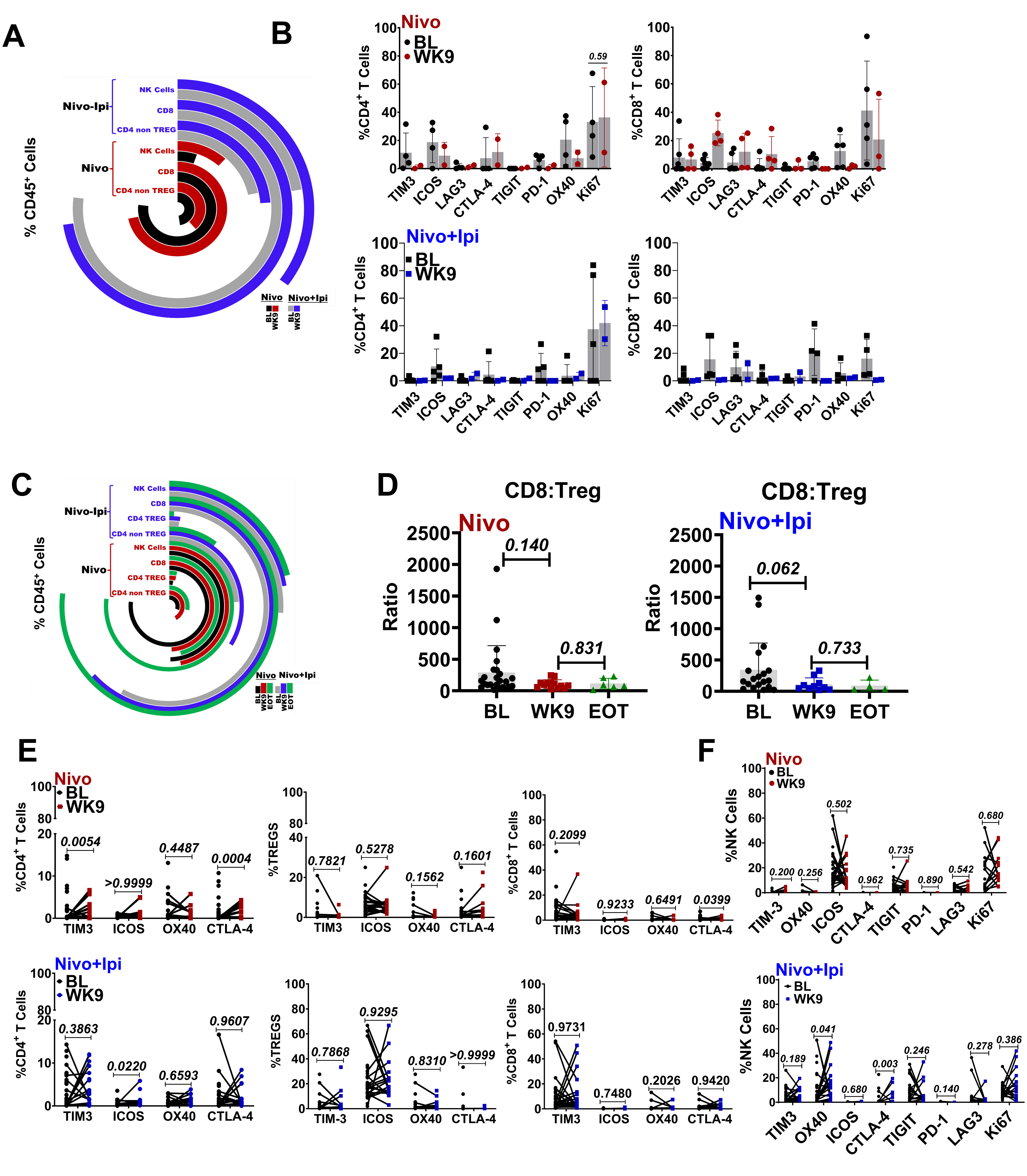

Supplementary Table 1: List of antibodies used for flow cytometry analysis

Antibody	Total Volume (µl)	Company	Catalog	Clone
TIGIT FITC	**5**	Life Technologies	11-9500-42	MBSA43
CD3 PerCP-Cy5.5	**10**	BD Biosciences	340949	SK7
Live/Dead Yellow	**1**	Life Technologies	L-34968	
TIM-3 (CD366) BV605	**4**	Biolegend	345018	F38-2E2
PD-1 (CD279) BV650	**3**	BD Biosciences	564104	EH12
OX40 (CD134) BV711	**5**	BD Biosciences	563664	ACT35
CTLA-4 (CD152) BV786	**3**	BD Biosciences	563931	BNI3
CD45 BUV395	**5**	BD Biosciences	563792	HI30
CD4 BUV496	**5**	BD Biosciences	612936	SK3
CD8 Alexa Fluor 700	**5**	BD Biosciences	557945	RPA-T8
CD25 APC-eFluor 780	**5**	Life Technologies	47-0259-42	BC96
Lag3 (CD223) PE	**5**	Life Technologies	12-2239-42	3DS223H
CD56 PE-CF594	**5**	BD Biosciences	562289	B159
ICOS (CD278) PE-Cy7	**3**	Life Technologies	25-9948-42	ISA-3
FoxP3 eFluor 450	**5**	Life Technologies	48-4776-42	PCH101
Ki67 APC	**5**	Life Technologies	17-5699-42	20Raj1

Supplementary Table 2: Univariate Cox proportional hazards model to estimate the association of covariates with PFS

		Coefficient	HR (95% CI)	P-value
Gender	Male vs. Female	0.02	1.02 (0.61-1.70)	0.93
Race	Asian vs. White	1.84	6.30 (0.84-47.6)	0.07
	Black or African American vs. White	0.13	1.14 (0.36-3.62)	0.82
ECOG PS	1 versus 0	0.15	1.16 (0.76-1.78)	0.49
Age	>=60 vs. <60	0.18	1.20 (0.78-1.83)	0.41

Supplementary Table 3: Univariable Cox proportional model to evaluate the association of OS with covariates

		Coefficient	HR (95% CI)	P-value
Gender	Male versus Female	0.14	1.15 (0.62-2.15)	0.65
ECOG PS	1 versus 0	0.50	1.65 (0.97-2.79)	0.06
Age	>=60 vs. <60	0.12	1.12 (0.67-1.89)	0.66

Supplementary Table 4: All Treatment-related Adverse Events by Arm and by Grade

	Number of patients experienced Treatment related AEs								
	Nivolumab			Nivolumab + ipilimumab					
Grade	1	2	3	1	2	3	4	5	Total
fatigue	6	4	0	9	2	1	0	0	22
diarrhea	5	2	0	4	2	0	0	0	13
anemia	1	4	0	6	0	1	0	0	12
nausea	4	0	0	6	2	0	0	0	12
hypothyroidism	2	2	0	3	4	0	0	0	11
anorexia	4	2	0	4	0	0	0	0	10
rash maculo-papular	3	0	1	5	1	0	0	0	10
pruritus	4	0	0	3	2	0	0	0	9
aspartate aminotransferase increased	1	0	0	2	2	1	0	0	6
hyperglycemia	1	0	0	2	0	0	3	0	6
mucositis oral	1	0	0	4	1	0	0	0	6
pneumonitis	1	0	0	0	1	3	0	1	6
abdominal pain	1	0	0	0	3	1	0	0	5
constipation	2	1	0	1	1	0	0	0	5
dyspnea	1	0	0	3	1	0	0	0	5
vomiting	2	0	0	2	0	1	0	0	5
alanine aminotransferase increased	0	0	0	1	1	2	0	0	4
arthralgia	1	2	0	1	0	0	0	0	4
cough	0	0	0	4	0	0	0	0	4
dry mouth	1	0	0	3	0	0	0	0	4
hyperthyroidism	3	0	0	1	0	0	0	0	4
hyponatremia	1	0	2	0	0	1		0	4

peripheral sensory neuropathy	0	1	0	3	0	0	0	0	4
arthritis	1	1	0	0	1	0	0	0	3
creatinine increased	1	1	0	1	0	0	0	0	3
dry skin	0	0	0	1	2	0	0	0	3
edema limbs	0	2	0	1	0	0	0	0	3
generalized muscle weakness	2	1	0	0	0	0	0	0	3
hypokalemia	0	0	0	2	0	1	0	0	3
infusion related reaction	1	1	0	0	1	0	0	0	3
lymphocyte count decreased	0	1	0	1	0	1	0	0	3
pain	0	1	0	2	0	0	0	0	3
white blood cell decreased	0	0	0	2	1	0	0	0	3
abdominal distension	0	0	1	1	0	0	0	0	2
alkaline phosphatase increased	0	0	0	0	2	0	0	0	2
blood bilirubin increased	0	0	0	1	0	1	0	0	2
chills	0	0	0	2	0	0	0	0	2
colitis	0	1	0	0	1	0	0	0	2
dizziness	0	0	0	1	1	0	0	0	2
dysgeusia	1	0	0	0	1	0	0	0	2
fever	1	1	0	0	0	0	0	0	2
headache	0	1	0	1	0	0	0	0	2
hypoalbuminemia	1	0	0	1	0	0	0	0	2
hypophosphatemia	0	0	1	1	0	0	0	0	2
lipase increased	0	0	1	1	0	0	0	0	2
platelet count decreased	1	0	0	1	0	0	0	0	2
adrenal insufficiency	0	0	0	0	0	1	0	0	1
allergic reaction	0	0	0	0	1	0	0	0	1
appetite decreased	0	0	0	1	0	0	0	0	1

blood lactate dehydrogenase increased	1	0	0	0	0	0	0	0	1
concentration impairment	0	0	0	1	0	0	0	0	1
covid-19	0	0	0	0	0	1	0	0	1
dehydration	0	0	0	0	1	0	0	0	1
dysarthria	0	0	0	1	0	0	0	0	1
dysuria	0	0	0	1	0	0	0	0	1
elevated thyroid-stimulating hormone	1	0	0	0	0	0	0	0	1
eosinophilia	0	0	0	1	0	0	0	0	1
epistaxis	0	0	0	1	0	0	0	0	1
erythema	0	0	0	0	1	0	0	0	1
face, neck, chest redness	0	0	0	1	0	0	0	0	1
facial muscle weakness	0	0	0	1	0	0	0	0	1
facial nerve disorder	0	0	0	0	1	0	0	0	1
flu like symptoms	0	0	0	1	0	0	0	0	1
flushing	1	0	0	0	0	0	0	0	1
hypermagnesemia	1	0	0	0	0	0	0	0	1
hypophysitis	0	0	0	0	0	1	0	0	1
intermittent jaw pain	1	0	0	0	0	0	0	0	1
jaw pain	1	0	0	0	0	0	0	0	1
lactate dehydrogenase increased	1	0	0	0	0	0	0	0	1
lip infection	0	0	0	0	1	0	0	0	1
lung infection	0	0	0	0	0	1	0	0	1
malaise	0	0	0	0	0	1	0	0	1
minimal change disease nephrotic syndrome	0	0	1	0	0	0	0	0	1
muscle cramp (left calf)	0	0	0	1	0	0	0	0	1
muscle weakness left-sided	0	0	0	1	0	0	0	0	1

myalgia	0	0	0	1	0	0	0	0	1
myositis	0	0	0	0	1	0	0	0	1
neurological other- difficulty word finding	1	0	0	0	0	0	0	0	1
neuropathy	0	0	0	1	0	0	0	0	1
neutrophil count decreased	0	0	0	0	1	0	0	0	1
peripheral motor neuropathy	0	0	0	1	0	0	0	0	1
photosensitivity	1	0	0	0	0	0	0	0	1
rash acneiform	0	0	0	1	0	0	0	0	1
redness	1	0	0	0	0	0	0	0	1
seizure	0	0	0	1	0	0	0	0	1
serum amylase increased	0	0	0	1	0	0	0	0	1
shingles	0	0	0	0	1	0	0	0	1
sinus cavity	0	0	0	0	1	0	0	0	1
skin infection	0	0	0	0	1	0	0	0	1
stomach pain	0	0	0	1	0	0	0	0	1
stomal hernia	0	0	0	1	0	0	0	0	1
vertigo	0	0	0	1	0	0	0	0	1
watering eyes	0	0	0	0	1	0	0	0	1
weight loss	0	0	0	0	1	0	0	0	1
wheezing	0	0	0	1	0	0	0	0	1

Supplementary Appendix 1

NCI #9673 Amendment Summary **Amendment #:**

1. Amendment was processed before study activation (related to part A – unrelated to current manuscript/randomized phase II part B trial).
2. Amendment was processed before study activation (related to part A – unrelated to current manuscript/randomized phase II part B trial).
3. Amendment was processed before study activation (related to part A – unrelated to current manuscript/randomized phase II part B trial).
4. Amendment was processed before study activation (related to part A – unrelated to current manuscript/randomized phase II part B trial).
5. Amendment to add the Guardant Health Assay language to describe the addition of cfDNA on the residual blood sample to optimize correlate information (related to part A – unrelated to current manuscript/randomized phase II part B trial).
6. Amendment to update the Comprehensive Adverse Events and Potential Risks (CAEPR) list for BMS-936558 (Nivolumab).
7. Amendment to expand the study to Part B to test combination of nivolumab and ipilimumab. *CTEP Disapproved.*
8. Amendment to expand the study to Part B to test combination of nivolumab and ipilimumab. *CIRB Approved pending modification*
9. Amendment to expand the study to Part B to test combination of nivolumab and ipilimumab. *CTEP Disapproved*
10. Amendment to expand the study to Part B to test combination of nivolumab and ipilimumab.
11. Amendment to update the CAEPR Tables.
12. Amendment to update staff contact information, clarify dosing information for nivolumab and ipilimumab, adverse event management, clarify correlative sample collection and shipping information, update Schedule of Events to reflect new testing to be performed, and additional regulatory items requested by CTEP.
13. Amendment to update the CAEPR Tables.
14. Amendment to update contact information of Principal Investigator who changed institutions during the conduct of the study.
15. Amendment to 1) improve clarity regarding treatment delays and re-initiating treatment after adverse event resolves; and 2) Change contact information for research staff
16. Amendment to add the option for sites to collect archival tissue for correlative analysis if fresh tissue is not collected, due to patient choice or institutional policy
17. Rapid amendment request by CTEP
18. Rapid amendment request by CTEP
19. CIRB requested amendment
20. CIRB requested amendment
21. Amendment to clarify the statistical analysis plan with regards to the timing of when the primary endpoint will be analyzed.

Supplementary Appendix 2

Tumor tissue was mechanically disaggregated using BD Medimachine System (BD Biosciences) and was subsequently filtered to generate single cell suspensions prior to staining. The samples were processed and stained within 24 hours of collection. Surface staining was performed in FACS Wash Buffer (1× DPBS with 1% BSA) for 30 min on ice using fluorochrome-conjugated monoclonal antibodies from BD Biosciences, BioLegend, and Life Technologies (Supplementary Table 1). Cells were then permeabilized using the BD Transcription factor kit according to the manufacturer's instructions. Samples were acquired using a BD Fortessa X20 and analyzed using FlowJo Software v10.9.0_CL. A Live/DeadTM fixable yellow dead dye (Invitrogen) was utilized to assess viability and exclude dead/dying cells from the analysis. Fluorescence minus one (FMO) controls were used for gating following doublet exclusion and viability assessment. For quality control (QC), subgating was only performed when more than 100 events were present in the parental population. The gating strategy is shown in Supplementary Figure S1.

Prior to staining, PBMCs were thawed, washed, and resuspended in FACS wash buffer (1× DPBS with 1% BSA). Flow cytometry staining and sample QC was performed as described above. Graphical analysis was performed using GraphPad Prism V10. We performed paired Wilcoxon rank t-test statistical analysis for comparison of biomarkers between baseline and week 9 (W9; time of first diagnostic imaging restaging for treatment response rate) in each arm.
