# Supplementary material for: NCI9673 (Part B): ETCTN Randomized Phase II Study of Nivolumab With or Without Ipilimumab in Refractory, Metastatic Squamous Cell Carcinoma of the Anal Canal
Source: J Clin Oncol. 2026 Jan 7;44(6):497–507. doi: 10.1200/JCO-25-00929 (PMC12782278; doi:10.1200/JCO-25-00929)
Supplement: Supplementary file 3 [file jco-44-497-s003.pdf]

SUMMARY OF CHANGES – PROTOCOL 9673

For Protocol Amendment #21: This protocol is being amended to clarify the statistical analysis plan with regards to the timing of when the primary endpoint will be analyzed.

Part A: A Multi-Institutional Phase 2 Study of Nivolumab in Refractory Metastatic Squamous Cell Carcinoma of the Anal Canal (Original Protocol)

Part B: A Multi-Institutional Phase 2 Study of Nivolumab or Nivolumab in Combination with Ipilimumab in Refractory Metastatic Squamous Cell Carcinoma of the Anal Canal (Amendment)

NCI Protocol #: 9673
Local Protocol #: NCI9673

NCI Version Date: March 24, 2023
Protocol Date: March 24, 2023

The changes reflect revisions from v11.14.2022 to 03.24.2023

#	Section	Page	Change
**1.**	TITLE	1	OLD TEXT:<br>*Version Date: ~~November 14, 2022~~*<br><br>NEW TEXT:<br>Version Date: **March 24, 2023**<br><br>RATIONALE: Updated protocol header so document version date matches most recent resubmission date.
**2.**	TITLE	3	NEW TEXT:<br>Amendment #21/Version Date: March 24, 2023<br><br>RATIONALE: Updated protocol title page with new amendment number and version date.
**3.**	14.6	127	NEW TEXT: The primary endpoint will be assessed when at least 8.2 months of followup after the last patient has been reached or when the study has observed 87 progression events, whichever occurs sooner.<br><br>RATIONALE: Added text to clarify the timing of when the primary endpoint will be analyzed.

NCI Protocol #: 9673

Local Protocol #: **NCI9673**

ClinicalTrials.gov Identifier: NCT02314169

TITLE: PART A: A MULTI-INSTITUTIONAL PHASE 2 STUDY OF NIVOLUMAB IN REFRACTORY METASTATIC SQUAMOUS CELL CARCINOMA OF THE ANAL CANAL (ORIGINAL PROTOCOL)

TITLE: PART B: A MULTI-INSTITUTIONAL PHASE 2 STUDY OF NIVOLUMAB OR NIVOLUMAB IN COMBINATION WITH IPILIMUMAB IN REFRACTORY METASTATIC SQUAMOUS CELL CARCINOMA OF THE ANAL CANAL (AMENDMENT)

Corresponding Organization: **LAO-CT018** / Yale University Cancer Center LAO

Principal Investigator: Cathy Eng, M.D., FACP
Vanderbilt-Ingram Cancer Center
2220 Pierce Avenue
Preston Research Building, Suite 777
Nashville, TN 37232
Telephone: 615-936-8422
Fax: 615-343-7602
Cathy.eng@vumc.org

Co-Principal Investigator: Van Morris, M.D.
1515 Holcombe Boulevard, Unit 426
University of Texas – MD Anderson Cancer Center
Houston, TX 77030
Telephone: (713) 792-2828
Fax: (713) 745-1163
vkmorris@mdanderson.org

Participating Organizations

**LAO-11030** / University Health Network Princess Margaret Cancer Center LAO
**LAO-CA043** / City of Hope Comprehensive Cancer Center LAO
**LAO-TX035** / University of Texas MD Anderson Cancer Center LAO
**LAO-MA036** / Dana-Farber - Harvard Cancer Center LAO
**LAO-MD017** / JHU Sidney Kimmel Comprehensive Cancer Center LAO
**LAO-MN026** / Mayo Clinic Cancer Center LAO

**LAO-NC010** / Duke University - Duke Cancer Institute LAO
**LAO-NJ066** / Rutgers University - Cancer Institute of New Jersey LAO
**LAO-OH007** / Ohio State University Comprehensive Cancer Center LAO
**LAO-PA015** / University of Pittsburgh Cancer Institute LAO
**LAO-NCI**/National Cancer Institute LAO
**EDDOP** / Early Drug Development Opportunity Program
**CATCHUP /** Creating Access to Targeted Cancer Therapy for Underserved Populations

Non-Member Collaborators

None

Statistician:
Lianchun Xiao
University of Texas –
MD Anderson Cancer Center
1515 Holcombe Boulevard, Unit 1411
Houston, TX 77030
Telephone: (713) 745-5984
lxiao@mdanderson.org

Study Coordinator:
Thomas Leonard-Martin
Vanderbilt-Ingram Cancer Center
Clinical Trials Shared Resource
3322 West End, Suite 1000
Nashville, TN 37203
Telephone: 615-936-6726
thomas.leonard-martin@vumc.org
Fax: 615-936-5850

Responsible Research Nurse:
Jennifer Teller
Vanderbilt-Ingram Cancer Center
Clinical Trials Shared Resource
2220 Pierce Avenue, 491 PRB
Nashville, TN 37232
Telephone: 615-875-9327
jennifer.l.teller@vumc.org

Responsible Data Manager:
Bo Whitson
Vanderbilt-Ingram Cancer Center
Clinical Trials Shared Resource
3322 West End, Suite 1000
Nashville, TN 37203
Telephone: 615-875-6515
bo.k.whitson@vumc.org

NCI-Supplied Agent:
Nivolumab (BMS-936558, MDX-1106, and ONO-4538) (NSC #748726)
Ipilimumab (MDX-010, NSC #732442)

IND #: *IND 125586*
IND Sponsor: DCTD, NCI
ClinicalTrials.gov Registration: NCT02314169

Protocol Type / Version # / Version Date: Original/ Version Date: October 30, 2014
Amendment # 1/Version Date: December 31, 2014
Amendment # 2/Version Date: January 27, 2015
Amendment # 3/Version Date: March 23, 2015
Amendment # 4/Version Date: March 2, 2016
Amendment # 5/Version Date: April 12, 2016
Amendment # 6/Version Date: February 22, 2017
Amendment # 7/Version Date: November 17, 2017 (CTEP Disapproved)
Amendment # 8/Version Date: December 19, 2017(CIRB approved pending modification)
Amendment # 9/Version Date: February 28, 2017 (CIRB Approved, CTEP Disapproved)
Amendment #10/Version Date: May 7, 2018
Amendment #11/Version Date: August 16, 2018
Amendment #12/Version Date: April 23, 2019
Amendment #13/Version Date: May 24, 2019
Ammendment 14/Version Date: June 25, 2019
Amendment #15/Version Date: November 19, 2019
Amendent #16/Version Date: July 15, 2020
Amendment #17/Version Date: August 27, 2020
Amendment #18/Version Date: February 3, 2021
Amendment #19/Version Date: March 18, 2021
Amendment #20/Version Date: November 14, 2022
Amendment #21/Version Date: March 24, 2023

PART A: SCHEMA
A Multi-Institutional Phase 2 Study of Nivolumab in Refractory Metastatic Squamous Cell Carcinoma of the Anal Canal (Original Protocol)

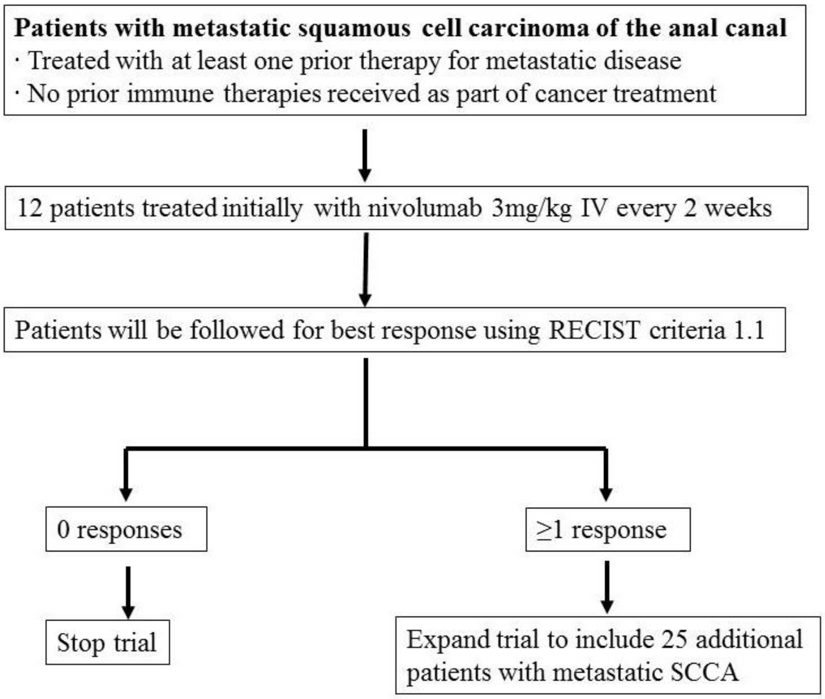

PART B: SCHEMA
A Multi-Institutional Phase 2 Study of Nivolumab or Nivolumab in Combination with Ipilimumab in Refractory Metastatic Squamous Cell Carcinoma of the Anal Canal (Amendment)

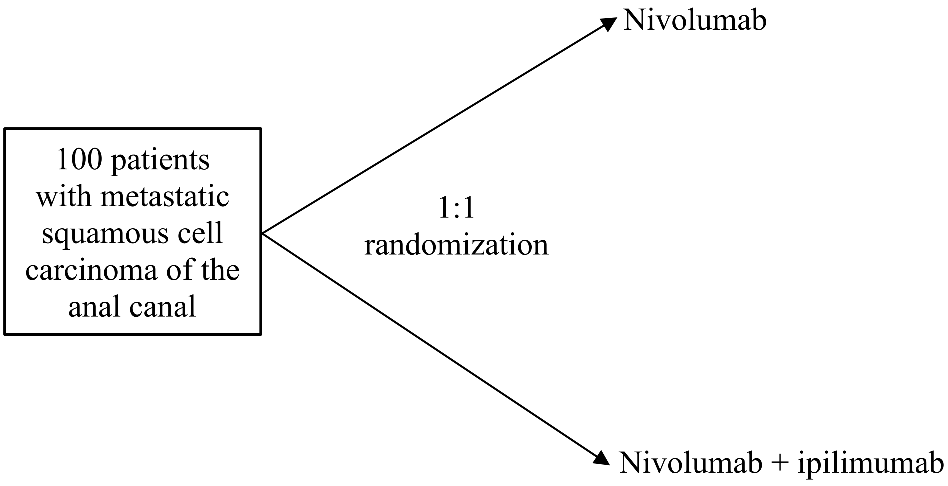

TABLE OF CONTENTS

TITLE: Part A: A Multi-Institutional Phase 2 Study of Nivolumab in Refractory Metastatic Squamous Cell Carcinoma of the Anal Canal (Original Protocol)....................1

TITLE: Part B: A Multi-Institutional Phase 2 Study of Nivolumab or Nivolumab in Combination with Ipilimumab in Refractory Metastatic Squamous Cell Carcinoma of the Anal Canal (Amendment)....................1

Part A: SCHEMA 4

Part B: SCHEMA 4

TABLE OF CONTENTS 5

1. OBJECTIVES 8
- 1.1 Primary Objectives....................8
- 1.2 Secondary Objectives....................8
- 1.3 Exploratory Objectives 8

2. BACKGROUND 9
- 2.1 Squamous Cell Carcinoma of the Anal Canal 9
- 2.2 Rationale (Parts A+B)....................27
- 2.3 Rationale for modified dosing for nivolumab and ipilimumab (Part B)....................30
- 2.4 Correlative Studies Background (Part A) 32
- 2.5 Correlative Studies Background (Part B) 35

3. PATIENT SELECTION 36
- 3.1 Eligibility Criteria (Parts A+B)....................36
- 3.2 Exclusion Criteria (Parts A+B)....................40
- 3.3 Inclusion of Women and Minorities 41

4. REGISTRATION PROCEDURES....................41
- 4.1 Investigator and Research Associate Registration with CTEP 41
- 4.2 Site Registration....................42
- 4.3 Patient Registration....................44
- 4.4 General Guidelines....................46

5. TREATMENT PLAN....................46
- 5.1 Treatment with nivolumab (Parts A+B) 46
- 5.2 Nivolumab Administration 46
- 5.3 General Concomitant Medication and Supportive Care Guidelines (Parts A+B) 47
- 5.4 Duration of Therapy (Parts A+B) 47
- 5.5 Duration of Follow Up....................48
- 5.6 Criteria for Removal from Study (Parts A+B)....................48

5.7 Criteria to Resume Treatment (Parts A+B) 49
5.8 Treatment of Nivolumab-Related Infusion Reactions (Parts A+B)....50
5.9 Ipilimumab Administration (Part B)....52
5.10 Prohibited and Restricted Therapies....61
5.11 Treatment Beyond Progression....62
5.12 Discontinuation of Study Therapy....62

6. DOSING DELAYS/DOSE MODIFICATIONS....63
6.1 Dosing Modifications....63
6.2 Dosing Delays....63

7. ADVERSE EVENTS: LIST AND REPORTING REQUIREMENTS....68
7.1 Comprehensive Adverse Events and Potential Risks List (CAEPR)....68
7.2 Adverse Event Characteristics....77
7.3 Expedited Adverse Event Reporting....778
7.4 Routine Adverse Event Reporting....799
7.5 Secondary Malignancy....790
7.6 Second Malignancy....80
7.7 Safety Monitoring Plan....80

8. PHARMACEUTICAL INFORMATION....801
8.1 CTEP IND Agent....801

9. BIOMARKER, CORRELATIVE, AND SPECIAL STUDIES (Part A Only, *see section 10 for part b)*....86
9.1 Integral Laboratory or Imaging Studies....86
9.2 Integrated Correlative Studies....86
9.3 Exploratory Correlative Studies....86
9.4 Immunohistochemical (IHC) Staining and Analysis for PD-L1, PD-1, CD_8^+, and CD_4^+ Tumor-Infiltrating Lymphocytes (Parts A and B)....92
9.5 Specimen Submission Summary....92
9.6 Special Studies - Analysis for the Presence of HPV....95
9.7 Optional Banking of Residual Tissue and Blood Samples for Future Research....96

10. Part B – Correlative and blood work:....96
10.1 Integral Laboratory or Imaging Studies....96
10.2 Integrated Correlative Studies....96
10.3 Exploratory Correlative Studies....96
10.4 Specimen Submission Summary....104
10.5 Optional Banking of Residual Tissue and Blood Samples for Future Research....106

11. STUDY CALENDAR....107
11.1 Part A:....107
11.2 Part B:....109

12. MEASUREMENT OF EFFECT 113
12.1 Antitumor Effect – Solid Tumors 113
12.2 Other Response Parameters 119

13. Study Oversight and DATA REPORTING / REGULATORY REQUIREMENTS 119
13.1 Study Oversight 119
13.2 Data Reporting 120
13.3 CTEP Multicenter Guidelines 122
13.4 Collaborative Agreements Language 122

14. STATISTICAL CONSIDERATIONS 124
14.1 Study Design/Endpoints (Part A) 124
14.2 Sample Size/Accrual Rate 125
14.3 Stratification Factors 125
14.4 Analysis of Secondary Endpoints 125
14.5 Analyses of Exploratory Endpoints: 126
14.6 Study Design/Endpoints (Part B) 126
14.7 Reporting and Exclusions 128

15. Study Status Updates and Study Closure 129
15.1 Definitions of Study Status Changes 129
15.2 Responsibility for Filing Protocol Status Update Forms 130

REFERENCES 131

APPENDIX A PERFORMANCE STATUS CRITERIA 139

APPENDIX B CTEP MULTICENTER GUIDELINES FOR NON-ETCTN TRIALS 140

APPENDIX C BIOASSAY TEMPLATES 141

APPENDIX D MANAGEMENT ALGORITHMS FOR ENDOCRINOPATHY, GASTROINTESTINAL, HEPATIC, NEUROLOGICAL, PULMONARY, RENAL, AND SKIN ADVERSE EVENTS 142

APPENDIX E ANALYSES OF SAMPLES FOR EXPLORATORY STUDIES 150

Appendix F ParticiPant Wallet Card 151

1. OBJECTIVES

1.1 Primary Objectives

To evaluate overall response rate (ORR) with nivolumab in patients with previously treated metastatic squamous cell carcinoma (SCCA) of the anal canal (Part A).

To determine an improvement in Progression-Free Survival (PFS) when nivolumab is combined with ipilimumab vs. nivolumab alone in patients with previously treated metastatic SCCA (Part B).

1.2 Secondary Objectives

<u>Part A:</u>

1.2.1 To evaluate progression-free survival (PFS) of nivolumab in patients with previously treated metastatic SCCA of the anal canal.

1.2.2 To evaluate overall survival (OS) in patients with previously treated metastatic SCCA of the anal canal treated with nivolumab.

1.2.3 To evaluate the grade 3 and 4 toxicity rate in patients with previously treated metastatic SCCA of the anal canal when treated with nivolumab.

<u>Part B:</u>

1.2.4 To evaluate the overall response rate (ORR) of nivolumab plus or minus ipilimumab in patients with previously treated metastatic SCCA of the anal canal.

1.2.5 To evaluate overall survival (OS) in patients with previously treated metastatic SCCA of the anal canal treated with nivolumab plus or minus ipilimumab.

1.2.6 To evaluate the grade 3 and 4 toxicity rate in patients with previously treated metastatic SCCA of the anal canal when treated with nivolumab plus or minus ipilimumab.

1.3 Exploratory Objectives

Part A:

1.3.1 To evaluate ORR, PFS, and OS based on expression of PD-L1, PD-1, peritumoral CD_8^+ tumor infiltrating lymphoctyes (TILs), peritumoral CD_4^+ TILs, and regulatory T cells as analyzed from tumor biopsies in previously treated patients with metastatic SCCA of the anal canal when treated with nivolumab.

1.3.2 To evaluate radiographic responses according to relative changes in proportions of anti-HPV specific CD_8^+ and CD_4^+ TILs and regulatory T cells in patients with previously treated metastatic SCCA of the anal canal following treatment with nivolumab, analyzed from serial peripheral blood samples.

Part B:

1.3.3 To evaluate ORR, PFS, and OS based on expression of PD-L1, PD-1, peritumoral CD_8^+ tumor infiltrating lymphoctyes (TILs), peritumoral CD_4^+ TILs, and regulatory T cells as analyzed from tumor biopsies in previously treated patients with metastatic SCCA of the anal canal when treated with nivolumab plus or minus ipilimumab.

1.3.4 To evaluate radiographic responses according to relative changes in proportions of anti-HPV specific CD_8^+ and CD_4^+ TILs and regulatory T cells in patients with previously treated metastatic SCCA of the anal canal following treatment with nivolumab plus or minus ipilimumab.

2. BACKGROUND

2.1 Squamous Cell Carcinoma of the Anal Canal

Squamous cell carcinoma (SCCA) of the anal canal accounts for an estimated 2% of all gastrointestinal malignancies in the US[1]. However, the annual incidence has risen steadily over the past two decades, with more than 8200 new cases expected in the United States in 2017[2,3]. Initially considered an orphan malignancy, the standard treatment paradigm of concurrent chemoradiation has largely remained unchanged in the US for greater than 3 decades[4,5]. Approximately 25% of patients initially treated with chemoradiation will develop locally advanced disease and/or distant metastases[6-9], and an additional 10% of patients will be diagnosed with stage IV disease at initial presentation[10]. There is currently no standard treatment approach for patients diagnosed with metastatic SCCA of the anal canal. Historically, chemotherapy regimens utilized in the treatment of these patients have largely been extrapolated from data in more common metastatic squamous cell carcinomas[11-15] (e.g., head and neck cancer and cervical cancer). A phase II international study has recently been initiated [InterAACT: C Eng (US Lead PI)] and is globally supported by the IRCI/NCI/EORTC/ECOG to identify the best chemotherapy backbone in treatment-naïve (HIV+ and HIV-) patients with SCCA of the anal canal*)*. To that end, large academic institutions serve as major referral sources for this disease given that outside providers are limited by the paucity of treatment options available to this population of patients.

2.1.1 **Nivolumab (Parts A and B)**

Nivolumab (BMS-936558, MDX-1106, and ONO-4538) is a fully human monoclonal immunoglobulin G4 (IgG4) antibody (HuMAb) that is specific for human programmed death-1 (PD-1, cluster of differentiation 279 [CD279]) cell surface membrane receptor[16]. PD-1 is a negative regulatory molecule that is expressed transiently following T-cell activation and on chronically stimulated T cells characterized by an "exhausted" phenotype. Nivolumab binds to cynomolgus monkey PD-1 but not mouse, rat, or rabbit molecules. Clinical activity of nivolumab has been observed in patients with melanoma, non-small cell lung cancer (NSCLC), and renal cell carcinoma (RCC). The combination of nivolumab and ipilimumab (anti-cytotoxic T lymphocyte associated antigen-4 [anti-CTLA-4]) in a phase II trial showed enhanced clinical activity relative to other agent alone, with an acceptable safety profile, in melanoma patients[17].

The clinical use of monoclonal antibodies to T-cell inhibitory receptors has provided transformative information on the nature of the immune system and cancer. An emerging picture suggests that endogenous immune responses can mediate effective tumor regression and/or improved survival even in patients with large volume tumors resistant to other forms of therapy. Some of the unique features of this type of therapy, based largely on experience in advanced melanoma, include: improved overall survival (OS) with or without radiographic responses or improved progression-free survival (PFS); responses that may be delayed or occur after radiographic disease progression; combinations of immune modulators with enhanced or novel activities (in the example of ipilimumab and nivolumab); and toxicity that is almost exclusively immune or inflammatory in nature. It is not yet clear what factors determine responses and which components of the immune system are needed for this to occur. It seems likely that both memory helper and effector cells would be needed to sustain long-term responses. Increasing emphasis has been placed on understanding the relationships of the tumor, cellular infiltrate, and immunologic milieu surrounding each tumor.

PD-1, a 55-kDa type 1 transmembrane protein, is a member of the CD28 family of T-cell co-stimulatory receptors that include Ig super family member CD28, CTLA-4, inducible co-stimulator (ICOS), and B and T lymphocyte attenuator (BTLA)[16]. PD-1 is transiently but highly expressed on activated T cells functioning to limit immune effectors at the site of activation. Chronic stimulation may prevent the re-methylation of the PD-1 gene leading to continuous expression and characterizes a state of "exhausted" T cells that lose function and proliferative capacity while enhancing a suppressive tumor microenvironment. PD-1 may act together with other T-cell modulating molecules, including CTLA-4, TIM-3, lymphocyte-activation gene 3 (LAG-3) as well as indoleamine-pyrrole 2, 3-dioxygenase 1 (IDO-1), cytokines, and transforming growth factor beta (TGF-beta).

Two ligands specific for PD-1 have been identified: PD-ligand 1 (PD-L1, also known as B7-H1 or CD274, expressed on tumor, antigen-presenting cells [APCs], and dendritic cells [DCs]) and PD-L2 (also known as B7-DC or CD273, expressed on endothelial cells). The interaction of PD-1 with PD-L1 and PD-L2 results in negative regulatory stimuli that down-modulate the activated T-cell immune response through SHP-1 phosphatase.

PD-1 knockout mice develop strain-specific lupus-like glomerulonephritis (C57BL/6) and

cardiomyopathy (BALB/c). In transplantable tumor models that expressed PD-1 and LAG-3 on tumor-infiltrating $CD4^+$ and $CD8^+$ T cells dual anti-LAG-3/anti-PD-1 antibody treatment cured most mice of established tumors that were largely resistant to single antibody treatment[18]. Despite minimal immunopathologic sequelae in PD-1 and LAG-3 single knockout mice, dual knockout mice abrogated self-tolerance with resultant autoimmune infiltrates in multiple organs, leading to eventual lethality.

PD-L1 expression is found on a number of tumors, and is associated with poor prognoses based on OS in many tumors, including melanoma[19], renal[20-22], esophageal[23], gastric[24], ovarian[25], pancreatic[26], lung[27], and other cancers[16].

The PD-1/PD-L1 axis plays a role in human infections, particularly in hepatitis C virus (HCV) and human immunodeficiency virus (HIV). In these cases, high expression levels of PD-1 were found in viral-specific $CD8^+$ T cells that also display a non-responsive or exhausted phenotype. Non-responsive PD-1-high T cells were observed in simian immunodeficiency virus (SIV) infection in rhesus macaques. Treatment of SIV-infected macaques with an anti-PD-1 mAb (3 mg/kg x4) resulted in decreased viral loads and increased survival along with expanded T cells with increased T-cell functionality.

2.1.1.1 Nonclinical Development of Nivolumab (Parts A and B)

In intravenous (IV) repeat-dose toxicology studies in cynomolgus monkeys, nivolumab alone was well tolerated[16]. Combination studies have highlighted the potential for toxicity when combined with ipilimumab, MDX-1408, and BMS-986016. Nivolumab bound specifically to PD-1 (and not to related members of the CD28 family such as CD28, ICOS, CTLA-4, and BTLA) with a K_d = 3.06 nM. A surrogate rat anti-mouse PD-1 antibody (4H2) was derived and expressed as chimeric IgG1 murine antibody. Antitumor activity was seen for several tumor models, including colon carcinoma and fibrosarcoma.

2.1.1.2 Clinical Development of Nivolumab (Parts A and B)

Nivolumab is being evaluated as monotherapy and in combination with cytotoxic chemotherapy, other immunotherapy (such as ipilimumab), anti-angiogenesis therapy, and targeted therapies in completed and ongoing BMS-sponsored clinical trials in NSCLC, melanoma, RCC, hepatocellular carcinoma (HCC), gastrointestinal (GI) malignancies including microsatellite instability (MSI) in colorectal cancer, and triple-negative breast cancer (TNBC) with an expanding group of indications[16]. In addition, two investigator-sponsored trials (ISTs) of nivolumab in combination with a peptide vaccine in melanoma are being conducted in the adjuvant setting and advanced disease.

Seven nivolumab studies were conducted in Japan, including six studies in advanced solid tumors and recurrent or unresectable stage III/IV melanoma sponsored by Ono Pharmaceuticals Co. Ltd., and one IST in recurrent or advanced platinum-refractory ovarian cancer.

2.1.1.2.1. Pharmacokinetics (Parts A and B)

Pharmacokinetics (PK) of nivolumab was linear in the range of 0.3 to 10 mg/kg, with dose-proportional increases in maximum serum concentration (C_{max}) and area under the concentration-time curve from time zero to infinity ($AUC_{0-\infty}$), with low to moderate inter-subject variability observed at each dose level[16]. Clearance of nivolumab is independent of dose in the dose range (0.1 to 10 mg/kg) and tumor types studied. Body weight normalized dosing showed approximately constant trough concentrations over a wide range of body weights. The mean terminal elimination half-life of BMS-936558 is 17 to 25 days consistent with the half-life of endogenous IgG4.

2.1.1.2.2. Efficacy (Parts A and B)

In a phase 1 (1, 3, and 10 mg/kg nivolumab doses) dose-escalation study the 3 mg/kg dose was chosen for expanded cohorts. Among 236 patients, objective responses (ORs) (complete or partial responses [CR or PR]) were seen in NSCLC, melanoma, and RCC. ORs were observed at all doses[28]. Median OS was 16.8 months across doses and 20.3 months at the 3 mg/kg dose. Median OS across all dose cohorts was 9.2 months and 9.6 months for squamous and non-squamous NSCLC, respectively[29]. In the RCC cohort, median duration of response was 12.9 months for both doses with 5 of the 10 responses lasting ≥1 year[30].

In an advanced melanoma phase 1 study, nivolumab and ipilimumab were administered IV every 3 weeks for 4 doses followed by nivolumab alone every 3 weeks for 4 doses (concurrent regimen) [17]. The combined treatment was subsequently administered every 12 weeks for up to 8 doses. In a sequenced regimen, patients previously treated with ipilimumab received nivolumab every 2 weeks for up to 48 doses. In the concurrent regimen (53 patients), 53% of patients had an OR at doses 1 mg/kg nivolumab and 3 mg/kg ipilimumab, with tumor reduction of 80% or more (modified World Health Organization [mWHO] criteria). In the sequenced-regimen (33 patients), the objective response rate (ORR) was 20%.

In a phase 1 study of nivolumab plus platinum-based doublet chemotherapy (PT-doublet) in chemotherapy-naïve NSCLC patients, 43 patients were treated with nivolumab + PT-doublet[31]. No dose-limiting toxicities (DLTs) were reported and total/confirmed ORRs were 43/33%, 40/33%, and 31/31% in nivolumab/gemcitabine/cisplatin, nivolumab/pemetrexed/cisplatin, and nivolumab/carboplatin/paclitaxel arms, respectively.

2.1.1.2.3. Toxicology (Parts A and B)

A maximum tolerated dose (MTD) of nivolumab was not defined[32]. Serious adverse events (SAEs) occurred in 32 of 296 patients (11%) similar to the immune-related inflammatory events seen with ipilimumab: pneumonitis, vitiligo, colitis, hepatitis, hypophysitis, and thyroiditis (with noted pulmonary toxicity resulting in 3 deaths. Renal failure, symptomatic pancreatic and DM, neurologic events, and vasculitis have also been reported.). In combination with ipilimumab in the concurrent-regimen group[17], grade 3 or 4 treatment-related events were noted in 53% of patients. Skin rash represents the majority of these events.

2.1.1.2.4. Pharmacodynamics/Biomarkers (Parts A and B)

Tumor-cell expression (melanoma) of PD-L1 was characterized in combination with ipilimumab with the use of IHC staining and pharmacodynamics changes in the peripheral-blood absolute lymphocyte count[17]. With PD-L1 positivity defined as expression in at least 5% of tumor cells, biopsy specimens from 21 of 56 patients (38%) were PD-L1–positive. Among patients treated with the concurrent regimen of nivolumab and ipilimumab, ORs were observed in patients with either PD-L1–positive tumor samples (6 of 13 patients) or PD-L1–negative tumor samples (9 of 22). In the sequenced regimen cohorts, a higher number of overall responses was seen among patients with PD-L1–positive tumor samples (4 of 8 patients) than among patients with PD-L1–negative tumor samples (1 of 13) suggesting the possibility that these tumors have higher response rates to the combination. The relationship between PDL-1 expression and responses may not be present in patients treated with the combination. Tissue expression of PDL-2, interferon-γ (IFN- γ), IDO, and T cell $CD8^+$ are of current interest. Until more reliable data based on standardized procedures for tissue collection and assays are available, PD-L1 status cannot be used to select patients for treatment at this time.

2.1.2 Ipilimumab (Part B)

Ipilimumab (MDX-010, MDX-CTLA4, BMS-734016) is being developed by CTEP as an anticancer agent in collaboration with Bristol-Myers-Squibb (BMS). On March 25, 2011, the FDA approved ipilimumab injection (YERVOY, BMS) for the treatment of unresectable or metastatic melanoma. Ipilimumab is a human $IgG_1\kappa$ monoclonal antibody (mAb); it is specific for human cytotoxic T lymphocyte-associated antigen-4 (CTLA-4, CD152) expressed on activated T cells. Ipilimumab is now produced and formulated from transfected Chinese hamster ovary (CHO) cells.

CTLA-4 is a negative regulator of T-cell responses following T-cell stimulation[33,34]. CTLA-4 knockout mice suffer from a fatal lymphoproliferative disorder, supporting the idea that CTLA-4 functions as a negative regulator of T-cell responses *in vivo*[35-37]. Disrupting CTLA-4 interaction with its ligands B7-1 (CD80) and B7-2 (CD86), which are expressed on antigen-presenting cells (APCs), with ipilimumab, augments immune responses (Investigator Brochure, 2011). *In vivo* blockade of CTLA-4, utilizing anti-CTLA-4 mAb, induced regression of established tumors and enhanced antitumor immune responses in several murine tumor models. Blockade of CTLA-4-mediated signals is effective in inducing rejection of immunogenic cancers in mice. These findings suggest that CTLA-4 blockade, alone or in combination with antigenic stimulation and other immune modulating agents can induce a potent antitumor response.

2.1.2.1 Pharmacology of Ipilimumab (Part B)

In vitro studies were performed with ipilimumab to demonstrate that it is specific for CTLA-4, actively inhibits CTLA-4 interactions with B7.1 and B7.2, does not show any cross-reactivity with human B7.1 or B7.2 negative cell lines, and stains the appropriate cells without non-specific cross-reactivity in normal human tissues. Ipilimumab does cross-react with CTLA-4 in non-human primates including cynomolgus monkeys. Blockade of CTLA-4/B7 interactions enhanced T-cell responses to CD3 / CD28, peptide antigens, or superantigens in mice[38-41]. CTLA-4 knockout mice appear to have spontaneously activated T cells evident at approximately 1 week after birth, followed by rampant lymphoproliferation and lymphadenopathy. These mice

die at approximately 3 weeks of age, either as a result of polyclonal T-cell expansion and tissue destruction or as a result of toxic shock resulting from lymphokine production. Genetically engineered mice heterozygous for CTLA-4 (CTLA-4+/-), appeared healthy and gave birth to healthy CTLA-4+/- heterozygous offspring. Mated CTLA-4+/- heterozygous mice also produced offspring deficient in CTLA-4 (homozygous negative, CTLA-4-/-). Since thymocyte differentiation and selection proceed normally in CTLA-4-deficient mice, the rampant T-cell expansion that occurs in the mice indicates that CTLA-4 plays a critical role in down-regulating post- thymic T-cell responses in the periphery following stimulation of naïve, memory, and effector T cells[41].

2.1.2.2 Pharmacokinetics (Part B)

The pharmacokinetics (PK) of ipilimumab was studied in 499 patients with unresectable or metastatic melanoma who received doses of 0.3, 3, or 10 mg/kg administered once every 3 weeks (q3w) for four doses. Peak concentration (Cmax), trough concentration (Cmin), and area under the curve (AUC) of ipilimumab were found to be dose proportional within the dose range examined. Upon repeated dosing of ipilimumab administered q3w, ipilimumab clearance was found to be time-invariant, and minimal systemic accumulation was observed as evident by an accumulation index of 1.5-fold or less. Ipilimumab steady-state concentration was reached by the third dose. The following mean (percent coefficient of variation) parameters were generated through population PK analysis: terminal half-life of 14.7 days (30.1%); systemic clearance (CL) of 15.3 mL/h (38.5%); and volume of distribution at steady-state (Vss) of 7.21 L (10.5%). The mean (±SD) ipilimumab Cmin achieved at steady-state with the 3-mg/kg regimen was 21.8 mcg/mL (±11.2).

Specific Populations: Cross-study analyses were performed on data from patients with a variety of conditions, including 420 patients with melanoma who received single or multiple infusions of ipilimumab at doses of 0.3, 3, or 10 mg/kg. The effects of various covariates on ipilimumab PK were assessed in population PK analyses.

Ipilimumab CL increased with increasing body weight; however, no dose adjustment of ipilimumab is required for body weight after administration on a mg/kg basis.

The following factors had no clinically meaningful effect on the CL of ipilimumab: age (range 26 to 86 years), gender, concomitant use of budesonide, performance status, HLA-A2*0201 status, positive anti-ipilimumab antibody status, prior use of systemic anticancer therapy, or baseline lactate dehydrogenase (LDH) levels. The effect of race was not examined as there were insufficient numbers of patients in non-Caucasian ethnic groups.

Renal Impairment: Creatinine clearance at baseline did not have a clinically important effect on ipilimumab PK in patients with calculated creatinine clearance values of 29 mL/min or greater.

Hepatic Impairment: Baseline AST, total bilirubin, and ALT levels did not have a clinically important effect on ipilimumab PK in patients with various degrees of hepatic impairment.

2.1.2.3 Clinical Pharmacodynamics (Part B)

In clinical studies, ipilimumab increased absolute lymphocytes counts (ALC) in peripheral blood (Investigator Brochure, 2011). However, CD4+/CD8+ ratio did not appear to be affected. Across three phase 2 studies in 463 subjects with advanced melanoma, ipilimumab increased ALC in a dose-dependent manner, with the largest increase observed at 10 mg/kg dose. ALC continued to increase over time during the induction treatment at least until week 12 at the 3 mg/kg and 10 mg/kg dose, but not at the 0.3 mg/kg dose. The slope of ALC increase also suggested the 10 mg/kg dose is more biologically active than the 3.0 mg/kg or 0.3 mg/kg dose.

2.1.2.4 Mechanism of Action (Part B)

The proposed mechanism of action for ipilimumab is T-cell potentiating through interference of the interaction of CTLA-4 with B7 (CD80 or CD86) molecules on APCs, with subsequent blockade of the inhibitory function of CTLA-4 (Investigator Brochure, 2011). Ipilimumab impacts tumor cells indirectly, and measurable clinical effects emerge after the immunological effects. Tumor infiltration with lymphocytes and the associated inflammation is likely the cornerstone of the effect of ipilimumab and can manifest in various patterns of clinical activity leading to tumor control. These immunologic responses may take time to develop and so tumor responses may be delayed and tumor progression may occur during the initial period followed by responses. In some cases, tumor response based on tumor infiltration with immune cells may be preceded by an apparent increase in initial tumor volume and/or the appearance of new lesions, which may be taken for tumor progression on radiological evaluations. Delayed responses following increasing tumor size or appearance of new lesions have been seen in approximately 10-20% of patients with metastatic melanoma. For patients who are not experiencing rapid clinical deterioration, allowing sufficient time to observe responses including disease stabilization or confirmation of progression is recommended; as discussed in the section “Overall Risk/Benefit Assessment” may allow better assessment of clinical activity and avoid unnecessarily initiating additional therapies in subjects who might be benefitting from treatment. Immune-related (ir) response criteria were developed based on these observations in patients with melanoma to systematically categorize novel patterns of clinical activity and are currently being prospectively evaluated in clinical studies.

2.1.2.5 Nonclinical Toxicology (Part B)

Please note relevant toxicity for single agent ipilimumab has been almost completely derived from clinical studies.

In a study using cynomolgus macaques, anti-melanocyte responses were observed in animals given up to four doses of 10 mg/kg ipilimumab after receiving a melanoma cell vaccine[42]. Depigmentation has been observed in other nonclinical immunotherapy studies that involve treatment with melanoma peptides[43-48]. The symptoms in animals appear to resemble vitiligo observed in clinical immunotherapy trials of melanoma patients and may be an unavoidable consequence of treatment[49].

Additional repeat-dose toxicity studies conducted using cynomolgus macaques demonstrated that the IV administration of ≤30 mg/kg every 3 days for three doses, 10 mg/kg weekly for 1 month, 1 mg/kg weekly for 10 weeks, or 10 mg/kg monthly for 6 months was generally well tolerated,

without significant clinical, immunotoxicological, or histopathological findings (Investigator Brochure, 2011). However, when ipilimumab was administered in combination with another immunomodulatory antibody (BMS-663513, a fully human anti-CD137 mAb) and simian immunodeficiency virus (SIV) DNA, two immune-related adverse events (irAEs) were observed: severe colitis requiring euthanasia in one monkey and reversible dermatitis/rash in the inguinal area and peripheral lymphadenopathy in another monkey.

Complete information on the pre-clinical toxicology studies can be found in the Ipilimumab Investigator Brochure (IB). Non-clinical toxicity assessments included *in vitro* cynomolgus monkeys alone and in the presence of vaccines. Low to moderate ADCC activity was noted at concentrations up to 50 mcg/mL. These data are consistent with the requirement of high levels of antigen expression on the surface of target cells for efficient ADCC or CDCC. No mortality or signs of toxicity were observed in three independent 14-day intravenous (IV) toxicology studies in cynomolgus monkeys at multiple doses up to 30 mg/kg/dose. Furthermore, ipilimumab was evaluated in sub-chronic and chronic toxicology studies in cynomolgus monkeys with and without Hepatitis B (HepB) Vaccine and Melanoma Vaccine. Ipilimumab was well tolerated alone or in combination in all studies. There were no significant changes in clinical signs, body weight values, clinical pathology values or T-cell activation markers. In addition, there were no significant histopathology changes in the stomach or colon.

2.1.2.6 Clinical Development of Ipilimumab (Part B)

<u>Company-Sponsored Studies</u>
BMS and Medarex (acquired by BMS in September 2009) have co-sponsored an extensive clinical development program for ipilimumab, encompassing more than 4000 subjects in several cancer types in 33 completed and ongoing studies (Investigator Brochure, 2011). The focus of the clinical program is in melanoma, prostate cancer, and lung cancer, with advanced melanoma being the most comprehensively studied indication. Ipilimumab is being investigated both as single agent and in combination with other modalities such as chemotherapy, radiation therapy, and other immunotherapy.

Phase 3 programs are ongoing in melanoma and prostate cancer (Investigator Brochure, 2011). In the phase 3 combination study of ipilimumab with glycoprotein 100 (gp100) peptide vaccine (melanoma study MDX010-20), ipilimumab was administered at a dose of 3 mg/kg of body weight, with or without gp100 q3w for up to four treatments[50]. The median overall survival (OS) in the ipilimumab plus gp100 group was 10.0 months (95% confidence interval [CI], 8.5 to 11.5 months) compared with 6.4 months (95% CI, 5.5 to 8.7 months) in the gp100-alone group (hazard ratio [HR] for death, 0.68; p<0.001). Grade 3 or 4 immune related events (irAEs) occurred in 10 to 15% of patients treated with ipilimumab and in 3% of patients treated with gp100 alone.

A second phase 3 study in melanoma was reported for ipilimumab in combination with dacarbazine versus dacarbazine alone in previously untreated advanced melanoma. CA184024 evaluated the addition of 10 mg/kg ipilimumab to dacarbazine in patients with previously untreated, metastatic melanoma. A total of 502 patients were randomized to receive up to 8 cycles of dacarbazine 850 mg/m^2 q3w, with either ipilimumab 10 mg or placebo for cycles 1-4

and as maintenance after completion of chemotherapy. Ipilimumab AEs were consistent with previous studies and predominantly affected skin, GI tract, liver, and the endocrine system. Events were managed with established guidelines and were generally responsive to dose interruption/discontinuation, corticosteroids and/or other immunosuppresants.

Checkmate 227 (NCT02477826) is an ongoing phase III trial of nivolumab, nivolumab plus ipilimumab, nivolumab plus platinum doublet therapy or platinum doublet chemotherapy in treatment naïve stage IV non-small cell lung cancer.

There are two ongoing studies of ipilimumab as adjuvant monotherapy for high-risk Stage III melanoma (CA184029) and ECOG 1609 (www.clinicaltrials.gov).

Two phase 3 studies are ongoing in subjects with castration-resistant prostate cancer who have received prior chemotherapy (CA184043, ipilimumab in combination with radiation therapy) and those who are chemotherapy-naïve (CA184095, ipilimumab monotherapy).

An ongoing large phase 2 study (CA184041) is investigating the addition of ipilimumab to carboplatin and paclitaxel using two different schedules (concurrent and phased) in subjects with non-small cell lung cancer (NSCLC) or small cell lung cancer (SCLC) (Investigator Brochure, 2011). The results demonstrate an improved immune-related progression-free survival (irPFS) for the subjects who received combination therapy with paclitaxel/carboplatin/ipilimumab compared with paclitaxel/carboplatin alone. The improvement in irPFS with the combination therapy met the prespecified protocol criteria for significance for both the concurrent and phased schedules (p=0.0935 and 0.0258, respectively). Using modified World Health Organization (mWHO) criteria (a key secondary endpoint), PFS was significantly improved with the phased schedule but not the concurrent schedule (p=0.0240 and 0.2502, respectively).

2.1.2.7 Other Clinical Studies with Ipilimumab (Part B)

Renal cell carcinoma (RCC)

Yang and colleagues presented data on a phase 2 study of ipilimumab conducted in patients with metastatic RCC[51]. Sequential cohorts received either 3 mg/kg followed by 1 mg/kg or all doses at 3 mg/kg q3w. One of 21 patients receiving the lower dose had a PR. Five of 40 patients at the higher dose had PRs (95% CI, cohort response rate 4 to 27%) and responses were seen in patients who had previously not responded to IL-2. Thirty-three percent of patients experienced grade 3 or 4 irAEs. There was a highly significant association between autoimmune events and tumor regression (response rate = 30% with AE, 0% without AE). The authors concluded that CTLA-4 blockade with ipilimumab induced cancer regression in some patients with metastatic clear cell renal cancer, even if they had not responded to other immunotherapies.

Melanoma (ipilimumab plus bevacizumab)

At the 2011 ASCO meeting Hodi and colleagues presented results on 21 evaluable patients (22 patients enrolled) with unresectable stage III or stage IV melanoma treated with the combination of 10 mg/kg ipilimumab and 7.5 mg/kg bevacizumab on a phase 1 study[52]. AEs included giant cell arteritis (1), hypophysitis (3), thyroiditis (4), grade 3-4 hepatitis (2), bilateral uveitis (2), and grade 2 colitis (2); 5 patients required systemic steroids and stopped treatment. All toxicities

were resolved. Eight PRs and 6 SDs were observed. All responses were durable (>6 months). Post-treatment biopsies in 12 patients revealed activated vessel endothelium with extensive T-cell trafficking non-productive central angiogenesis, and peripheral blood monitoring revealed a marked increase in CD4/CCR7/CD45RO central memory cells in the majority of patients, not seen with ipilimumab alone. The authors concluded that the combination of ipilimumab with bevacizumab can be safely administered with clinical activity and correlatives suggesting synergistic effects.

<u>Bladder cancer</u>
Carthon and colleagues reported immunodulatory effects following a brief exposure of anti-CTLA-4 in patients with urothelial carcinoma of the bladder requiring surgery (BMS study CA184027)[53]. 12 patients were enrolled (6 patients received 3 mg/kg/dose of ipilimumab and another 6 patients received 10 mg/kg/dose for two doses prior to surgery). The treatment was found to be tolerable in the cohort of patients with 11 of 12 patients receiving both doses of antibody. Grade 1-2 diarrhea and rash were the most common drug-related AEs. The only noted grade 3 irAEs were ischemic papillopathy and diarrhea, which were both responsive to treatment with steroids.

Liakou and colleagues found that CD4 T cells from peripheral blood and tumor tissues of all bladder cancer patients treated with anti-CTLA-4 antibody had markedly increased expression of inducible costimulator (ICOS)[54]. These $CD4^+ICOS^{hi}$ T cells produced interferon-gamma (IFN-γ) and could recognize the tumor antigen NY-ESO-1. Increase in $CD4^+ICOS^{hi}$ cells led to an increase in the ratio of effector to regulatory T cells. The authors indicated that these immunologic changes were reported in both tumor tissues and peripheral blood as a result of treatment with anti-CTLA-4 antibody, and they may be used to guide dosing and scheduling of this agent to improve clinical responses. A sustained increased frequency of $CD4^+ICOS^{hi}$ T cells may serve as a biomarker of anti-CTLA-4 activity and/or of clinical benefit for patients who are being treated with this novel agent[53].

<u>Pancreatic cancer</u>
Royal and colleagues presented the results on 27 patients (metastatic disease: 20 and locally advanced: 7)[55]. Three subjects experienced ≥ grade 3 irAEs (colitis:1, encephalitis:1, hypophysitis:1). One subject experienced a delayed response after initial progressive disease. In this subject, new metastases after 2 doses of ipilimumab established progressive disease. However, continued administration of the agent per protocol resulted in significant delayed regression of the primary lesion and 20 hepatic metastases with normalization of tumor markers and clinically significant improvement of performance status. The investigators concluded that single agent ipilimumab at 3.0 mg/kg/dose was ineffective for the treatment of advanced pancreatic cancer. However, a significant delayed response in one subject of this trial suggests that immunotherapeutic approaches to pancreatic cancer deserve further exploration.

<u>CTEP-Sponsored Studies</u>
The DCTD, NCI, has sponsored nine studies with ipilimumab including one pilot study (NCI #5744, lymphoma), three phase 1 studies (5708 [ovarian], 6082 [solid tumors], and 7458 [solid tumors]), one phase 1/2 study (6359 [non-Hodgkin's lymphoma]) with single agent ipilimumab, two phase 1 combination studies in prostate cancer with GM-CSF (6032) and with prostate-

specific antigen (PSA)-TRICOM vaccine (7207), one phase 2 combination study of ipilimumab with GM-CSF (E1608, melanoma) and one phase 3 study (E1609) of adjuvant ipilimumab therapy versus high-dose interferon alpha-2b in patients with resected high-risk melanoma.

Results from 11 patients (colon, n=3; non-Hodgkin's lymphoma, n=4; prostate, n=4) who received ipilimumab on study 5744 included tumor regression in 2 patients with lymphoma; 1 of whom (follicular lymphoma patient) had a partial response (PR) of 14-month duration[56]. Ipilimumab was well tolerated with predominantly grade 1/2 toxicities. One drug-related grade 3 AE was observed. Tregs, as detected by expression of $CD4^{+}CD25^{+}CD62L^{+}$, declined at early time points but rebounded to levels at or above baseline values at the time of the next infusion. The investigators concluded that ipilimumab treatment depressed Treg numbers at early time points in the treatment cycle but was not accompanied by an increase in vaccine-specific CD8+ T-cell responses in these patients previously treated with a variety of investigational anticancer vaccines.

Hodi and colleagues reported preliminary results on 20 patients (11 metastatic melanoma patients and 9 metastatic ovarian carcinoma patients) on study 5708[57]. None of the 11 patients from the metastatic melanoma cohort manifested grade 3 or 4 inflammatory toxicities; however, all subjects revealed mild inflammatory pathologies associated with low-level constitutional symptoms. The most common toxicity (10/11 subjects) was a grade 1-2 reticular and erythematous rash on the trunk and/or extremities that arose between 3 days and 3 weeks after antibody administration and then gradually resolved without specific intervention. Biopsies of involved skin revealed low-grade interface dermatitis, minor to moderate mononuclear infiltrates surrounding the superficial dermal vasculature, and increased mucin deposition in the papillary and reticular dermis. These pathologic features resembled those observed in mild cutaneous forms of systemic lupus erythematosis. Three PRs (range, 21-34+ months) and five events of stable disease (SD) (range, 4-25 months) were observed. One PR and three SDs (2, 4, and 6+ months) were observed in the ovarian carcinoma group. The investigators concluded that selective targeting of antitumor regulatory T cells (Treg) may constitute a complementary strategy for combination of ipilimumab and GM-CSF-based antigen tumor cell vaccine therapy.

Results from 29 patients with malignancies that were recurrent or progressive after allogeneic hematopoietic cell transplantation (allo-HCT) demonstrated that drug was well tolerated at single doses up to 3 mg/kg[58]. Four patients experienced organ-specific irAEs of reversible grade 3 arthritis, grade 2 hyperthyroidism, dyspnea, and grade 4 pneumonitis. Three patients had objective responses: one PR lasting for 2 months, and two durable complete responses (CRs). Two additional patients with Hodgkin's disease who had evidence of rapid disease progression prior to ipilimumab treatment achieved SD for 3 and 6 months, following infusion at the 3 mg/kg dose level. Median OS was 24.7 months. At a 3.0 mg/kg dose, active serum concentrations of ipilimumab were maintained for >30 days following a single infusion. Zhou and colleagues reported immunophenotypes of peripheral blood T cells, including T-cell reconstitution, activation, and Treg expression, in 29 patients before and after a single-dose infusion of ipilimumab[59]. CTLA-4 blockade by a single infusion of ipilimumab increased $CD4^{+}$ and $CD4^{+}HLA\text{-}DR^{+}$ T lymphocyte counts and intracellular CTLA-4 expression at the highest dose level (3.0 mg/kg). There was no significant change in Treg cell numbers after ipilimumab infusion. These data demonstrate that significant changes in T-cell populations occur on

exposure to a single dose of ipilimumab.

Harzstark and colleagues reported results on 36 patients with hormone refractory metastatic prostate cancer[60]. Of six patients treated with ipilimumab at a dose of 3 mg/kg, three patients had confirmed PSA declines of ≥50%, with a time to progression (TTP) of 22, 26, and 103 weeks. One of these patients had a PR in hepatic metastases. Grade 3 IrAEs consisted of rash in five patients, panhypopituitarism in one patient, temporal arteritis in one patient, and diarrhea in three patients. Non-irAEs included grade 3 and 4 cerebrovascular events (one patient each), grade 3 angina (one patient), grade 3 atrial fibrillation (one patient), grade 3 fatigue (four patients), and grade 5 pulmonary embolism (one patient). One patient treated at 10 mg/kg had a PSA decline of ≥50% with a TTP of 39 weeks. Higher doses of treatment with MDX-010 + GM-CSF induced the expansion of activated circulating $CD25^+$, $CD69^+$, and $CD8^+$ T cells more frequently than was seen in patients who received the same doses of either MDX-010 or GM-CSF alone[61]. The sera screening with protein arrays showed that the treatment can induce antibody responses to the testicular antigen NY-ESO-1.

Patients with metastatic prostate cancer were treated with ProstVac vaccine and ipilimumab before chemotherapy. The median OS for all patients on study was 31.8 months with a 74% survival probability at 24 months[62]. The median Halabi predicted OS for all patients was 18.5 months. There was no significant difference in OS at different dose levels of antibody (range 1-10 mg). A unique effect of the vaccines on the rate of tumor growth may be a novel method to evaluate the anti-tumor effects of the vaccine[63]. The authors suggested that the addition of immune checkpoint inhibition may augment the clinical benefit of vaccines.

Ansell and colleagues reported data on 18 treated patients with NHL[64]. Two clinical responses were observed: one patient with diffuse large B-cell lymphoma (BCL) had an ongoing CR (>31 months), and one with follicular lymphoma had a PR lasting 19 months. In 5 of 16 cases tested, T-cell proliferation to recall antigens was >2 fold increased after ipilimumab therapy. The investigators have found that blockade of CTLA-4 signaling with the use of ipilimumab is well tolerated at the doses used. Ipilimumab has antitumor activity in patients with BCL, resulting in durable responses in a minority of patients. Ipilimumab at 3 mg/kg monthly for 4 months can be given safely and is the dose that recommended for future combination studies.

2.1.2.8 Clinical Safety

<u>Safety Experience</u>

The most common treatment-related AEs (those considered possibly, probably, or definitely related to study drug by the investigator) associated with the use of ipilimumab were immune related irAEs (Investigator Brochure, 2011). The irAEs primarily involved the gastrointestinal (GI) tract (*e.g.*, diarrhea and colitis) and skin (*e.g.*, pruritus and rash), and less frequently, the liver, endocrine glands (including the thyroid, pituitary, and adrenal glands) and nervous system. IrAEs were generally managed with either symptomatic therapy (grade 1-2 events), systemic corticosteroids (grade 3-4 events), or other immunosuppressants (*e.g.*, infliximab, mycophenolate mofetil) for steroid-unresponsive GI or hepatic irAEs, as appropriate. Management of irAEs was usually paired with omission of dosing for mild or moderate events and permanent discontinuation for severe irAEs. Ipilimumab can result in severe and fatal immune-mediated

reactions due to T-cell activation and proliferation. Fatalities due to GI perforation, hepatic failure, toxic epidermal necrolysis, and Guillain-Barré syndrome have been reported in clinical trials of ipilimumab.

Clinical trials are conducted under widely varying conditions so that extrapolation to novel settings and combinations regarding rates and severity of events may be unreliable. Given the expected rate of toxicity which may require stopping study drug but may also be related to a therapeutic immunologic response alternative DLT criteria are discussed in Section 6.

Min and colleagues reported three patients who received ipilimumab alone or combined with bevacizumab therapy and developed thyroiditis, and the first report of euthyroid Graves' ophthalmopathy[65]. They recommend that all patients on ipilimumab alone or combined with bevacizumab therapy have baseline thyroid function tests and careful monitoring for new onset of thyroid disease, particularly during the first 3 months of treatment. See specific events in Section 5.

Safety Profile of Ipilimumab at a Dose of 10 mg/kg (Phase 2 data)
The safety profile of ipilimumab as monotherapy over multiple doses at a dose of 10 mg/kg in 325 subjects was determined from 4 completed melanoma studies. Overall, the incidence of grade 3/4 AEs attributable to study drug was 31%. The target organ system, the incidence, and the severity of the most commonly observed irAEs vary among studies and with drug combinations. Typically, the severity but not necessarily the overall incidence increases with dose. Additional information on specific events is provided in Section 7.1 and the IB:

Summary of irAE Safety Data for 10 mg/kg in Melanoma

	Total	Low-grade (Grade 1 - 2) (%)	High-grade (Grade 3 - 4) (%)	Median Time to Resolution of Grade 2 - 4 irAEs (weeks)
All irAEs	72.3	46.2	25.2	-
Skin (*e.g.*, rash, pruritus)	52.0	49.2	2.8	6.14
GI (*e.g.*, colitis, diarrhea)	37.2	24.9	12.3	2.29
Liver (*e.g.*, LFT elevations)	8.0	0.9	6.8	4.0
Endocrine (*e.g.*, hypophysitis, hypothyroid)	6.2	3.7	2.5	20.1

Pregnancy
Preliminary results are available in cynomolgus monkeys. Pregnant monkeys received ipilimumab every 21 days from the onset of organogenesis in the first trimester through delivery, at dose levels either 2.6 or 7.2 times higher than the clinical dose of 3 mg/kg of ipilimumab (by AUC). No treatment-related adverse effects on reproduction were detected during the first two trimesters of pregnancy. Beginning in the third trimester, the ipilimumab groups experienced higher incidences of abortion, stillbirth, premature delivery (with corresponding lower birth weight), and higher incidences of infant mortality in a dose-related manner compared to controls.

Based on animal data, ipilimumab may cause fetal harm. The use of ipilimumab during human pregnancy has not been formally studied in clinical trials. There have been 7 known pregnancies during ipilimumab treatment: in 3 female subjects and in the partners of 4 male study subjects. Two (2) of the 3 female pregnancies ended with elected terminations. The third female subject had a history of seizures and delivered the baby at 36 weeks gestation. The baby had respiratory complications that resolved by birth week 16. Three (3) of the 4 partners of male study subjects had full term, normal babies. The fourth baby had small ureters, which are expected to grow as the baby matures. Although these outcomes do not indicate that stillbirths or other severe abnormalities will occur, pregnancy should be avoided during treatment with ipilimumab.

Immunogenicity

In clinical studies, 1.1% of 1024 evaluable patients tested positive for binding antibodies against ipilimumab in an electrochemiluminescent (ECL) based assay. This assay has substantial limitations in detecting anti-ipilimumab antibodies in the presence of ipilimumab. Infusion-related or peri-infusional reactions consistent with hypersensitivity or anaphylaxis were not reported in these 11 patients nor were neutralizing antibodies against ipilimumab detected. Because trough levels of ipilimumab interfere with the ECL assay results, a subset analysis was performed in the dose cohort with the lowest trough levels. In this analysis, 6.9% of 58 evaluable patients, who were treated with 0.3 mg/kg dose, tested positive for binding antibodies against ipilimumab. These results are highly dependent on methodology, and comparison of incidence of antibodies to ipilimumab with the incidences of antibodies to other products may be misleading.

Study Results and Clinical Efficacy

The clinical efficacy of ipilimumab as a single agent at a dose of 3 mg/kg administered q3w for 4 doses has been established in MDX010-20 (a randomized, controlled study in second line, locally advanced/metastatic melanoma), which led to approval of ipilimumab by the FDA. In study CA184024, the addition of 10 mg/kg ipilimumab to dacarbazine led to a prolongation of overall survival in patients with previously untreated melanoma.

In melanoma studies, disease stabilization in subjects receiving ipilimumab is characteristic of anti-tumor activity. Stable disease, sometimes of long duration, or slow steady decline of tumor lesion size over long periods of time, has been observed. Some subjects demonstrate initial tumor volume increase before response, possibly due to T-cell infiltration as shown by biopsies or to the time required for immunologic activation. Consequently, an initial determination of progressive disease and consequently PFS may not capture all patterns response and may underestimate the clinical activity of ipilimumab. Please see section "Considerations for Using Immune-Related Tumor Assessment Criteria (irRC)."

MDX010-20 (Phase 3, 3 mg/kg, previously treated melanoma)

MDX010-20, a randomized (3:1:1), double-blind, double-dummy study included 676 randomized subjects with unresectable or metastatic melanoma previously treated with one or more of the following: aldesleukin, dacarbazine, temozolomide, fotemustine, or carboplatin. Of these 676 subjects, 403 were randomized to receive ipilimumab at 3 mg/kg in combination with an investigational peptide vaccine with incomplete Freund's adjuvant (gp100), 137 were randomized to receive ipilimumab at 3 mg/kg, and 136 were randomized to receive gp100 alone.

The study enrolled only subjects with HLA A2*0201 genotype; this HLA genotype facilitates the immune presentation of the investigational peptide vaccine. The study excluded subjects with active autoimmune disease or those receiving systemic immunosuppression for organ transplantation. The OS results are shown in the table below.

MDX010-20 Overall Survival Results

	**Ipilimumab n = 137**	**Ipilimumab + gp100 n = 403**	**gp100 n = 136**
Hazard Ratio (vs gp100) (95% CI)	0.66 (0.51, 0.87)	0.68 (0.55, 0.85)	
p-value	p = 0.0026[a]	p = 0.0004	
Hazard Ratio (vs ipilimumab) (95% CI)		1.04 (0.83, 1.30)	
Median (months) (95% CI)	10 (8.0, 13.8)	10 (8.5, 11.5)	6 (5.5, 8.7)

[a]Not adjusted for multiple comparisons.

<u>CA184024 (Phase 3, previously untreated melanoma, 10 mg/kg)</u>
CA184024 evaluated the addition of 10 mg/kg ipilimumab to dacarbazine in patients with previously untreated, metastatic melanoma. A total of 502 patients were randomized to receive up to 8 cycles of dacarbazine 850 mg/m^2 q3w, with either ipilimumab 10 mg/kg or placebo cycles 1-4, and as maintenance after completion of chemotherapy.

Patients on the ipilimumab arm received a median of 3 ipilimumab induction doses, versus 4 placebo induction doses on the placebo arm. A total of 17.4% and 21.1% of patients continued to receive maintenance ipilimumab or placebo, for a median of 4 and 2 doses, respectively. The number of patients who received all 8 dacarbazine doses was 12.2% in the ipilimumab arm, and 21.5% in the placebo arm.

The study met its primary end-point of prolonging overall survival in patients treated with ipilimumab (HR 0.72 [95% CI, 0.59 – 0.87], median OS 11.2 vs 9.1 months, p = 0.0009).

One, two, and three year survival rates were 47.3%, 28.5%, and 20.8% in the ipilimumab arm, and 36.3%, 17.9%, and 12.2% in the placebo arm.

PFS, a secondary end-point, was also prolonged by the addition of ipilimumab, HR 0.76 (95% CI, 0.63 - 0.93). The median PFS was 2.8 months in the ipilimumab vs 2.6 months in the placebo arm, p = 0.006.

Best overall response rate (BORR) was increased from 10.3% in the placebo arm to 15.2% in the ipilimumab arm. More importantly, duration of response was more than twice as long in the ipilimumab arm (19.3 months) than in the placebo arm (8.1 months).

<u>10 mg/kg Dosing with Ipilimumab</u>

Several additional trials studied the efficacy and safety of 10 mg/kg dosing, and additional information gained from these trials is listed below:
A dose of 10 mg/kg may be necessary to ensure blockade of the CTLA-4 pathway; *in vitro,* a concentration of 20 mcg/mL of ipilimumab was the minimal concentration able to fully abrogate the binding of CTLA-4 to B7.1 and B7.2. With a dose of 3 mg/kg q3w, 30% achieved a trough concentration of ipilimumab greater than 20 µg/mL, compared to 95% of subjects treated at 10 mg/kg q3w.

In addition, in all ipilimumab trials examined to date, mean Absolute Lymphocyte Count (ALC) increased after ipilimumab treatment throughout the 12-week induction-dosing period, in a dose-dependent manner. In an analysis of ipilimumab at 0.3, 3, or 10 mg/kg in melanoma studies CA184007, CA184008, and CA184022 combined, the rate of change in ALC after ipilimumab treatment was significantly associated with dose (p = 0.0003), with the largest rate at 10 mg/kg ipilimumab. Moreover, the rate of change in ALC over the first half of the induction-dosing period was significantly associated with clinical activity in these studies (p = 0.009), where clinical activity was defined as CR, PR, or prolonged SD (*i.e.*, SD lasting at least 6 months from first dose). Although these analyses alone could not determine whether the rate of change in ALC was specifically associated with clinical activity in response to ipilimumab treatment, as opposed to being generally prognostic, these results do suggest a potential benefit to higher rates of ALC increase after ipilimumab treatment. Among the 3 doses evaluated, 10 mg/kg ipilimumab led to the greatest such rates.

In the 3 primary studies conducted in advanced melanoma (CA184007, CA184008, and CA184022), subjects treated with 10 mg/kg single agent ipilimumab had the highest response, disease control rates, median OS as well as 1-year and 2-year survival rates compared to lower doses. The CA184022 data are summarized in the table below.

Summary of Phase 2 Response Data in Melanoma (CA184022 see IB)

	**10 mg/kg (n = 72)**	**3 mg/kg (n = 72)**	**0.3 mg/kg (n = 73)**
**BORR (mWHO) – % (95% CI)**	11.1 (4.9 - 20.7)	4.2 (0.9 - 11.7)	0 (0.0 - 4.9)
**DCR (mWHO) – % (95% CI)**	29.2 (19.0 - 41.1)	26.4 (16.7 - 38.1)	13.7 (6.8 - 23.8)
**Survival rate at 1 year - % %, 95% CI**	48.64 (36.84, 60.36)	39.32 (27.97, 50.87)	39.58 (28.20, 51.19)
**Survival rate at 2 year - % %, 95% CI**	29.81 (19.13, 41.14)	24.20 (14.42, 34.75)	18.43 (9.62, 28.22)
**Overall median survival 95%CI (months)**	11.43 (6.90, 16.10)	8.74 (6.87, 12.12)	8.57 (7.69, 12.71)

Dose, Schedule, and Regimen

While optimal doses and schedules for ipilimumab have not yet been determined, in proposed proof of principle studies demonstration of efficacy at 10 mg/kg would allow future studies to

explore biologic and clinical efficacy at lower doses with reduced toxicity. For most studies in new combinations or settings, a short phase 1 component at 3 mg would be appropriate with a 5 or 6 mg/kg dose added as an additional cohort if needed.

A recommended dose of 10 mg/kg is proposed by the manufacturer for most studies of ipilimumab. In melanoma, a similar survival benefit was demonstrated in phase 3 trials at the 3 mg/kg and at 10 mg/kg with DTIC. However, the incidence of grade ≥3 toxicity was 15 and 25% respectively.

Based on Phase 2 studies, response rates of ipilimumab appear to be dose dependent up to 10 mg/kg. Exposure-response analyses [C_{min}ss Analysis of PK data from patients treated with ipilimumab at 0.3 mg/kg (N=47), 3 mg/kg (N=60) and 10 mg/kg (N=311)], showed that the target C_{min}ss target threshold of 20 mcg/ml was exceeded in 0%, 30% and 95% subjects respectively. The slope of change in absolute lymphocyte count (ALC) correlated with clinical benefit and T-cell activation markers such as HLA-class II expression may also be dose dependent. Responses have not been compared systematically in randomized phase 2 or phase 3 studies in patients with tumor types other than melanoma.

Regarding schedule, the typical schedule for advanced melanoma at present is once q3w for four doses followed by a maintenance phase of four doses every 12 weeks. Of interest, ipilimumab was evaluated in NSCLC and SCLC using a dose of 10 mg/kg given concomitantly or following initial paclitaxel/cisplatin. When used in the phased schedule, 10 mg significantly improved irPFS and mWHO defined responses but not PFS determined by Response Evaluation Criteria in Solid Tumors (RECIST). There also was a trend for an improvement in OS in both indications. Doses less than 10 mg/kg have not been evaluated in either NSCLC or SCLC.

Studies comparing doses in non-melanoma and combinations have not been widely done. There are also no clear data that peak levels, Cmin, AUC, exposure and number of doses given, or the occurrence of autoimmune events, predict responses in individual patients. We note that the incidence of specific events such as hypophysitis may vary from study to study and with different combinations of agents. The severity and possibly time to onset but not necessarily the frequency of events increases with dose. In addition, there are rare but serious events such as toxic epidermal necrolysis (TEN) for which a dose relationship has not been established. Case report forms should include data on the prior treatment, timing, number of doses, duration of event, response to treatment, and complications to allow comparisons among studies.

<u>Considerations in Using Immune-Related Tumor Assessment Criteria (irRC)</u>
New end point definitions for trials of immunologic agents have been proposed based on novel patterns of clinical activity in malignant melanoma[66,67]. These alternative definitions allow time for immunologically mediated effectors to develop that may result in late tumor responses even after initial progression by RECIST. Also, in some patients, tumor necrosis and inflammation may increase tumor size radiographically prior to response. Changing the definitions of OR and PD may alter (increase) the number patients achieving responses and the duration of PFS.

On a protocol by protocol basis, we would consider allowing study treatment to continue during initial progression up to the 12-16 week assessment to allow time for responses to be observed, if

the patient is clinically stable, there is no deterioration in PS, and there is no need for immediate additional treatment. While maintaining standard definitions of progression and response, we would allow new lesions and some progression beyond 20% increases in tumor measurements during the initial treatment period to allow time for responses to develop (these delayed tumor responses may be seen in 10-20% of melanoma patients who initially progress during the initial treatment cycles and evaluation). We do not have experience with response patterns with combination therapy nor in diseases other than melanoma. Please use standard response definitions as the primary end point in these studies.

Note that the proposed irRC may be incorporated as secondary end points to compare to standard criteria and evaluate alternative patterns of response in various disease setting and treatment regimens. A copy of the proposed criteria is presented in Appendix D.

Patients who demonstrate mixed responses, stable disease, or objective responses by standard RECIST following initial progression may be identified separately as “delayed SD, PR, or CR”.

<u>Overall Risk/Benefit Assessment</u>
The unique immune-based mechanism of action is reflected in the clinical patterns of anti-cancer activity in some patients. Ipilimumab affects tumor cells indirectly, and measurable clinical effects emerge after the immunological effects. Tumor infiltration with lymphocytes and the associated inflammation (documented by biopsy in some subjects) is likely the cornerstone of the effect of ipilimumab and can manifest in various patterns of clinical activity leading to tumor control. In some cases, response may be preceded by an apparent increase in initial tumor volume and/or the appearance of new lesions, which may be mistaken for tumor progression on radiological evaluations. Therefore, in subjects who are not experiencing rapid clinical deterioration, confirmation of progression is recommended, at the investigator’s discretion, to better understand the prognosis as well as to avoid unnecessarily initiating potentially toxic alternative therapies in subjects who might be benefitting from treatment. Immune-related (ir) response criteria were developed based on these observations to systematically categorize novel patterns of clinical activity and are currently being prospectively evaluated in clinical studies.

In metastatic diseases, stabilization is more common than response, and in some instances is associated with slow, steady decline in tumor burden over many months, sometimes improving to partial and/or complete responses. Thus, the immune-based mechanism of action of ipilimumab results in durable disease control, sometimes with novel patterns of response, which contribute to its improvement in OS.

The immune-based mechanism of action is also reflected in the safety profile. The most common drug-related AEs are immune-mediated, consistent with the mechanism of action of the drug and generally medically manageable with topical and/or systemic immunosuppressants. As previously discussed, the immune-mediated adverse reactions primarily involve the GI tract, skin, liver, endocrine glands, and nervous system.

The early diagnosis of immune-mediated adverse reactions is important to initiate therapy and minimize complications. Immune-mediated adverse reactions are generally manageable using symptomatic or immunosuppressive therapy as recommended through detailed diagnosis and

management guidelines, as described fully in the current IB. The management guidelines for general immune-mediated adverse reactions and ipilimumab-related GI toxicities, hepatotoxicity, endocrinopathy, and neuropathy are provided in the appendices of the current IB.

2.2 Rationale (Parts A+B)

The development of SCCA of the anal canal is often triggered by enduring infection with high-risk strands of human papillomavirus (HPV), most commonly HPV-16 and HPV-18[68-71]. Approximately 75% of all sexually active adults will become infected with HPV during their lifetimes[72]. While most will clear infection of HPV without any required intervention[73], the virus may persist in a fraction of patients and cause cancer when viral DNA becomes incorporated into the host genome. The oncoproteins E6 and E7, translated products of HPV DNA integrated into the host cell, promote oncogenesis in mucosal squamous cells of anal canal through multiple mechanisms. For example, E6 binds p53 to generate a complex which results in ubiquitin-mediated p53 degradation[74-76]. Likewise, the interaction between E7 and the retinoblastoma protein Rb triggers a phosphorylation of Rb and allows continuation of cell cycle progression through the loss of tumor suppressor function by Rb[77-79].

The presence of high-risk HPV has been detected in approximately 75-95% of all reported cases of SCCA of the anal canal[68-71]. In addition, this virus has also been correlated to the development of other malignancies like SCCAs of the head and neck (over 70% of which are HPV-positive tumors), cervix (> 90%), penis (>60%), vulva (~70%), and vagina (75%) [16,30,80-85]. A clear association between altered immunity and the development of SCCA is apparent, and a functional immune system may be responsible for clearing HPV infection. Immunosuppression from HIV/AIDS, use of immunosuppressive medications following organ transplantation, and coexisting autoimmune disease are all recognized risk factors for developing SCCA[19,23,28,68,86]. Furthermore, the HPV-generated oncoproteins E6 and E7 are immunogenic and trigger an anti-tumor host immune response via activation of tumor infiltrating lymphocytes (TILs) [87-91]. These neoantigens are examples of "non-self" molecules recognized by T cells which are produced following viral incorporation into the host genome. However, suppression of such anti-tumor activity may allow a tumor to evade an immune response and survive in HPV-related malignancies. This notion has been verified in invasive cervical cancer, in which HPV-specific CD8+ cytotoxic T cells and CD4+ helper T-cells are lost and inhibitory HPV-specific regulatory T cells[92-94] become activated.

In a preventative trial, vaccination with a quadrivalent HPV vaccine (covering HPV-16) showed fewer cases of precancerous high-grade anal intraepithelial neoplasia when compared to people who received placebo, and no cases of invasive carcinoma[95]. Likewise, similar results in preventing development of pre-cancerous cervical dysplasia have been described by vaccinating young women with the quadrivalent HPV vaccine[87]. Despite such clinical promise, recent studies have suggested that fewer than 30% of eligible females in the United States have received the complete series of HPV vaccinations, and this proportion is even lower in males[96]. In underdeveloped countries, where the prevalence of HPV infection and HPV-associated malignancies are higher than in developed nations, providing access to these preventative vaccinations are currently in development, so that at present the majority of citizens do not receive these vaccines. In addition, patients with HPV-associated anogenital malignancies are

exposed to an increased risk of developing a second HPV-related cancer from the effects of field cancerization caused by HPV infection[97]. Therefore, for these reasons it is expected that the incidence of SCCA of the anal canal will continue to rise and will continue to worsen in global prevalence for several decades.

Tumor cells express the programmed death receptor ligand-1 (PD-L1) as a means to down regulate T cell activation and thwart the local anti-tumor immune response by binding the inhibitory receptor programmed death-1(PD-1) on the surface of T cells[21,98]. Expression of PD-L1 on resected oral squamous cell tumors demonstrated a negative correlation with intratumoral CD8+ TIL density[99]. Additionally, in one study of HPV+ tumors of the oropharynx, 14 of 20 specimens (70%) demonstrated increased PD-L1 expression by immunohistochemistry (IHC), whereas only 2 of 7 (29%) HPV- tumors stained positive for PD-L1[100]. These results support the notion that HPV+ tumors may utilize the PD-1: PD-L1 interaction between the tumor cell and nearby T cell in order to disrupt an antitumor host immune response.

Nivolumab is a monoclonal antibody against PD-1. In vitro, disruption of PD-1: PD-L1 communication by nivolumab perpetuates T-cell activity against tumor cells[101,102]. When translated to a phase I study of nivolumab as a single agent, objective responses have been observed in over 20% of patients with refractory metastatic melanoma, non-small cell lung cancer, and renal cell carcinoma[32]. Additionally, among tumor specimens for which PD-L1 expression could be evaluated by IHC, a positive correlation between PD-L1 expression and response to nivolumab was detected, and no tumors that lacked PD-L1 expression demonstrated a response to nivolumab.

The prognostic impact of HPV in patients with metastatic SCCA of the anal canal remains unreported. In a series of 72 patients with metastatic SCCA of the anal canal who had undergone biopsy and/or surgical resection of their tumors at MD Anderson for survival outcomes[103], tumors were tested for the presence of HPV using in-situ hybridization and by immunohistochemistry with monoclonal antibodies against P16, a protein regarded as a surrogate biomarker for HPV infection[104] with upregulated expression following loss of E7-mediated Rb function[105,106]. HPV was detected in 68 of the 72 patients (95%) analyzed. Per Figure 1, no differences in survival were measured between the HPV-positive and HPV-negative cohorts, although a trend towards improved survival was seen in the HPV-positive patients. This result may be attributed to an under powering to detect true differences because the overwhelming majority of patients with metastatic SCCA of the anal canal treated at M.D. Anderson Cancer Center have detectable HPV.

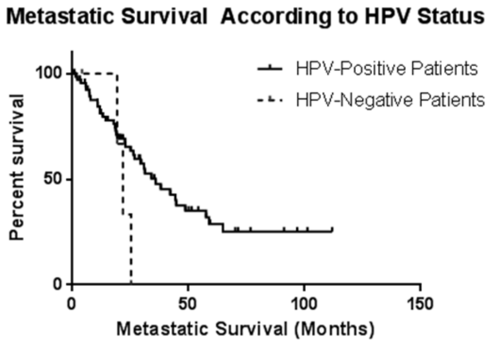

Figure 1: Median survival from the time of detection of metastatic disease was 35.9 months in the HPV-positive group and 27.1 months in the HPV-negative group (p-value = 0.11).

Rationale for the combination (Part B):
The immunogenicity of tumor cells renders them susceptible to immune recognition and attack by cytotoxic T cells. In response, multiple adaptive mechanisms within the tumor

microenvironment, through inhibitory interactions of the intratumoral T cells with both the tumor cell antigen-presenting cells, can thwart the anti-tumor immune response. For example, binding of B7 on APCs with CTLA-4 on activated T cells can downregulate the effector function of cytotoxic T cells specific to a given tumor [40-42]. Ipilimumab is a monoclonal antibody against CTLA-4 which impairs this B7:CTLA-4 interaction at the T cell surface and retains T cell activation [43,44]. This drug has received FDA approval for the treatment of metastatic melanoma following multiple positive phase III trials [45,46], and recent reports have demonstrated that 20% of patients with this disease remain alive after three years following treatment with ipilimumab[47]. Therefore, manipulation of the immune response for advanced tumors like melanoma improve patient outcomes when standard cytotoxic therapies are less effective and generate significant optimism for these findings to be similar in other immunogenic tumors like metastatic SCCA.

Other surface proteins on the tumor cells themselves like programmed death receptor ligand-1 (PD-L1) can be expressed to downregulate T cell activation by binding the inhibitory receptor PD-1 on the surface of T cells [48,49]. Expression of PD-L1 on resected oral squamous cell tumors demonstrated a negative correlation with intratumoral CD8+ TIL density[50]. Additionally, in one study of HPV+ tumors of the oropharynx, 14 of 20 specimens (70%) demonstrated increased PD-L1 expression by immunohistochemistry (IHC), whereas only 2 of 7 (29%) HPV- tumors stained positive for PD-L1[51]. These results support the notion that HPV+ tumors may likewise utilize the PD-1:PD-L1 interaction between the tumor cell and nearby T cell in order to disrupt an antitumor hose immune response. Nivolumab is a monoclonal antibody against PD-1, and in vitro, disruption of PD-1:PD-L1 communication by nivolumab perpetuates T-cell activity against tumor cells[52,53]. As a single agent, nivolumab has demonstrated significant clinical activity for patients with refractory metastatic melanoma, non-small cell lung cancer, renal cell carcinoma, bladder cancer, and Hodgkin's lymphoma[54].bladder cancer, and Hodgkin's lymphoma[54].

Anti-PD1 therapy has activity in patients with previously treated metastatic SCCA: We completed a single-arm, multi-institutional ETCTN phase II trial of nivolumab for patients with refractory SCCA (NCI9673)[107]. This trial accrued all 37 patients within 6 months. Objective responses were noted in 9 of 37 treated patients (response rate 24%), with a median progression-free survival of 4.1 months. The responses rate met the predefined statistical significance for efficacy in this study. The median number of administered doses of nivolumab was 6 (interquartile range, 3-10). Treatment was overall safe and well tolerated. These findings suggest that anti-PD1 therapy may be a novel effective treatment approach for patients with metastatic SCCA.

Based on our preliminary findings suggesting that durable responses to anti-PD-1 therapy can be observed for patients with metastatic SCCA in the presence of an inflammatory peritumoral milieu, it is feasible that additional agents targeting the immune checkpoint axes can further deepen the anti-tumor response and improve clinical outcomes in this setting. While nivolumab has earned FDA approval as a single-agent therapy for advanced malignancies like melanoma and non-small cell lung cancer (NSCLC), the combination of nivolumab with ipilimumab appears to improve response rates further for affected patients. Indeed, in a phase II trial comparing nivolumab and ipilimumab revealed improved clinical outcomes for patients treated with the combination relative to ipilimumab alone. Analogously, recent results from the CheckMate-012 study reported response rates of 39% in patients with NSCLC who received

nivolumab 3 mg/kg every 2 weeks and ipilimumab 1 mg/kg every 6 weeks. The combination of immune checkpoint agents targeting PD-1 and CTLA-4 together appeared to have improved clinical outcomes for NSCLC when compared to prior studies with the response rates of single agent nivolumab of approximately 20%. Recent data supports the PD-1 and CTLA-4 combination in two phase III trials in treatment naïve advanced melanoma patients[108]. NCT02231749 (Checkmate 214) is an ongoing phase III trial of nivolumab + ipilimumab versus sunitinib in treatment-naïve advanced or metastatic renal cell carcinoma patients[109].

Collectively, these findings suggest that dual blockade of non-overlapping immune checkpoint targets may augment the anti-tumor immune response as manifested by improved clinical outcomes for patients treated with anti-PD-1 and anti-CTLA-4 agents together.

To expand upon the success of nivolumab as a single-agent observed in the NCI 9673 study, we have amended NCI9673 for a randomized phase II trial of nivolumab plus/minus ipilimumab for patients with previously treated metastatic anal cancer. We have demonstrated through the aforementioned NCI 9673 trial that patients with this disease can successfully be accrued through collaboration between multiple institutions within the ETCTN network. Based on the findings of this study, we envision future trials in which nivolumab, with/or without ipilimumab (pending the efficacy of the immunotherapy combination), could be compared to traditional cytotoxic chemotherapeutic regimens commonly used in the management of this disease in order to identify the optimal treatment approach for patients with this disease.

2.3 Rationale for modified dosing for nivolumab and ipilimumab (Part B)

Nivolumab

Nivolumab monotherapy has been extensively studied in multiple tumor types, including melanoma, NSCLC, renal cell carcinoma (RCC), classical Hodgkin's lymphoma (cHL), head and neck (H&N) and urothelial carcinoma (UC), using body weight normalized dosing (mg/kg), and has been safely administered at doses up to 10 mg/kg Q2W. Nivolumab is currently approved in the US for use in melanoma, NSCLC, RCC, and UC as 240 mg Q2W, and in cHL and H&N as 3 mg/kg Q2W.

The nivolumab dose of 480 mg Q4W was selected for this study based on clinical data and modeling and simulation approaches using population PK (PPK) and exposure-response (ER) analyses examining relationships between nivolumab exposures and efficacy (e.g. OS, OR) and safety responses, using data from studies in multiple tumor types (melanoma, NSCLC, and RCC) with body weight-normalized dosing (mg/kg). The PPK analyses have shown that exposure to nivolumab increased dose proportionally over the dose range of 0.1 to 10 mg/kg administered Q2W, and no clinically meaningful differences in PK across ethnicities and tumor types were observed. Nivolumab clearance and volume of distribution were found to increase as body weight increases but less than proportionally with increasing weight, indicating that milligram-per-kilogram dosing represents an over-adjustment for the effect of body weight on nivolumab PK.

Using the PPK and ER models, nivolumab exposures and probabilities of efficacy responses and risks of AEs were predicted following nivolumab 480 mg Q4W and compared to those following nivolumab 3 mg/kg Q2W. The overall distributions of average nivolumab steady state exposures

(Cavgss) are comparable following administration with either nivolumab 3 mg/kg Q2W or nivolumab 480 mg Q4W. Nivolumab 480 mg Q4W is predicted to result in approximately 43% greater steady state peak concentrations (Cmaxss) compared to nivolumab 3 mg/kg Q2W; however, these exposures are predicted to be lower than the exposure ranges observed at doses up to nivolumab 10 mg/kg Q2W used in the nivolumab clinical program. Although the Cmaxss of nivolumab is expected to be greater following nivolumab 480 mg Q4W compared to nivolumab 3 mg/kg Q2W, the predicted Cmaxss following nivolumab 480 mg Q4W is well below the median Cmaxss achieved following administration of nivolumab 10 mg/kg Q2W, a safe and tolerable dose level.

Exposure-safety analysis demonstrated that the exposure margins for safety are maintained following nivolumab 480 mg Q4W, and the predicted risks of AE-DC/D, AE Grade 3+, and AE-IM Grade 2+, are predicted to be similar following nivolumab 480 mg Q4W relative to nivolumab 3 mg/kg Q2W across tumor types. Safety analyses using available data following nivolumab 3 mg/kg Q2W and 10 mg/kg Q2W administration indicated there were no differences in AE profiles across body weight groups. Finally, initial evidence demonstrates that following administration of nivolumab 480 mg Q4W, nivolumab has been shown to be well tolerated.

Nivolumab 480 mg Q4W is predicted to be approximately 16% lower steady-state trough concentrations (Cminss) compared to nivolumab 3 mg/kg Q2W. While these exposures are predicted to be lower, they are on the flat part of the exposure response curves and are not predicted to affect efficacy, Exposure-efficacy analyses of multiple PK measures and efficacy endpoints (e.g. OS, OR) indicated that following administration of nivolumab 480 mg Q4W efficacy is predicted to be similar to that following administration of nivolumab 3 mg/kg Q2W across multiple tumor types. Based on these data, nivolumab 480 mg Q4W is expected to have similar efficacy and safety profiles to nivolumab 240 mg Q2W or nivolumab 3 mg/kg Q2W.

Ipilimumab

In CA209012, ipilimumab 1 mg/kg using Q6W and Q12W schedules were assessed and were found to be safe in combination with nivolumab 3 mg/kg Q2W. Both the Q6W and Q12W arms were associated with improved and manageable tolerability compared to the arms with more frequent ipilimumab dosing (Q3W).

Rationale for shorter infusion time:

Administration of nivolumab using a 30-minute infusion time has been evaluated in subjects with cancer. Previous clinical studies of nivolumab monotherapy for the treatment of cancer have used a 60-minute infusion duration wherein nivolumab has been safely administered up to 10 mg/kg over long treatment periods. Infusion reactions including high-grade hypersensitivity reactions have been uncommon across the nivolumab clinical program. In CA209010, a dose association was observed for infusion site reactions and hypersensitivity reactions (1.7% at 0.3 mg/kg, 3.7% at 2 mg/kg and 18.5% at 10 mg/kg). All the events were Grade 1-2 and were manageable. An infusion duration of 30 minutes for 3 mg/kg nivolumab (30% of the dose provided at 10 mg/kg) is not expected to present any safety concerns compared to the prior experience at 10 mg/kg nivolumab dose infused over a 60-minute duration. The safety of nivolumab 3 mg/kg administered as a 30-min infusion was assessed in CA209153 in patients

(n=322) with previously treated advanced NSCLC. Overall, there were no clinically meaningful differences in the frequency of hypersensitivity/infusion-related reactions (of any cause or treatment-related) in cancer patients administered nivolumab over a 30 min infusion compared with that reported for patients with the 60 min infusion. Thus, it was shown that nivolumab can be safely infused over 30 min in subjects with cancer.

- Similarly, ipilimumab at 10 mg/kg has been safely administered over 90 minutes. In the CA184022 study, where ipilimumab was administered up to a dose of 10 mg/kg, on-study drug-related hypersensitivity events (Grade 1-2) were reported in 1 (1.4%) participant in the 0.3 mg/kg and in 2 (2.8%) participants in the 10 mg/kg group. There were no drug-related hypersensitivity events reported in the 3 mg/kg group. Across the 3 treatment groups, no Grade 3-4 drug-related hypersensitivity events were reported, and there were no reports of infusion reactions. Ipilimumab 10 mg/kg monotherapy has also been safely administered as 90-minute infusion in large Phase 3 studies in prostate cancer (CA184043) and as adjuvant therapy for stage 3 melanoma (CA184029), with no infusion reactions occurring in participants. Administering 1 mg/kg of ipilimumab represents one-tenth of the 10 mg/kg dose.

Overall, infusion reactions including high-grade hypersensitivity reactions have been uncommon across nivolumab or ipilimumab clinical studies or the combination of nivolumab and ipilimumab. Furthermore, a 30-minute break after the first infusion for the combination cohort will ensure the appropriate safety monitoring before the start of the second infusion. Overall, a change in safety profile is not anticipated with 30-minute infusion of nivolumab, ipilimumab, or combination.

2.4 Correlative Studies Background (Part A)

2.4.1 Role of PD-L1 and PD-1 in the response of SCCA of the anal canal to nivolumab

2.4.1.1 Rationale

While PD-L1 expression has been reported in a variety of solid tumors[21,29,85,110,111], no data exist describing the expression of this protein in SCCA of the anal canal.

2.4.1.2 Background

Unpublished data from a small cohort of surgically resected tumors from patients with metastatic SCCA of the anal canal was collected for PD-L1 expression using an assay at MD Anderson which had previously been optimized to detect successfully cell surface PD-L1 expression in HPV-positive cervical and penile cancers. Tumors were stained using a monoclonal antibody against PD-L1 (Epitomics; Burlingame, CA) and defined as "PD-L1 positive" if at least 5% of tumor cells demonstrated PD-L1 surface expression (based on criteria used in prior published studies with nivolumab)[32]. As exemplified in Figure 2, staining in PD-L1 was seen in two of the four (50%) tumors analyzed. However, all four tumors exhibited peritumoral lymphocytes, a feature which has been associated with a more avid anti-tumor immune response in patients with other solid malignancies[112]. These data demonstrate that PD-L1 may be expressed in patients with HPV-positive metastatic SCCA of the anal canal.

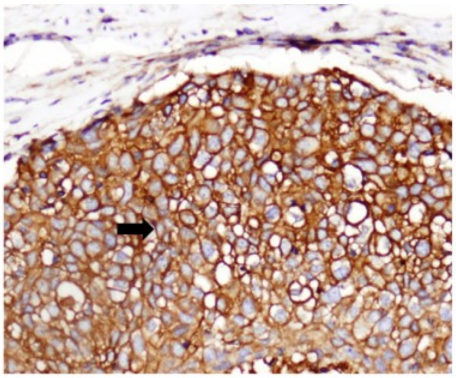

Figure 2: Using a monoclonal antibody against the PD-L1 protein, diffuse cell surface staining of PD-L1 (brown) is observed on the surfaces of tumors cells from a resected SCCA specimen. The arrow points to a representative single cell staining positive for PD-L1 around its entire membrane.

2.4.1.3 Hypothesis

Patients with metastatic SCCA of the anal canal will respond to therapy with nivolumab if their tumors stain positive for PD-L1 prior to treatment. Durable responses to therapy may be observed in patients whose tumors maintain PD-L1staining on immunohistochemistry after two cycles of therapy. Analogously, we hypothesize that the detection of PD-1, present on T-cells to interact with PD-L1, will also be associated with an anti-tumor response for patients treated with nivolumab.

2.4.2 Role of CD_8^+ Infiltrating T-cells in the response of SCCA of the anal canal to nivolumab

2.4.2.1 Rationale

The associations between the presence of CD_8^+ infiltrating T cells and (1) responses to nivolumab and (2) survival outcomes have never been described in a prospective study for patients with metastatic SCCA of the anal canal. This analysis would not only inform on the relevance of these immune cells in this particular disease for the first time, but could also be used in understanding further the role of CD_8^+ infiltrating T cells as a potential biomarker for immune checkpoint inhibitors like nivolumab across various HPV-related malignancies. The role of HPV-specific CD8+ T cells after nivolumab treatment may be of especial importance for clinical response. It will also be easy to assay since we already know their specificity (see 2.3.4. below).

2.4.2.2 Background

Tumor specimens from a series of 38 patients with locoregional, non-metastatic SCCA of the

anal canal were reviewed retrospectively for a correlation between the presence of CD_8^+ infiltrating T cells[113]. No correlation between the higher numbers of these T cells and clinical outcomes were observed. However, patients with metastatic disease were not included in this analysis, and descriptions of an association between CD_8^+ infiltrating T cells, and not only overall survival in patients with metastatic SCCA of the anal canal but also responses to anti-PD-L1 therapy have not been described.

2.4.2.3 Hypothesis

Given the cytotoxic nature of CD_8^+ infiltrating T cells against tumors, we hypothesize that a positive correlation between CD_8^+ infiltrating T cells and response to nivolumab will be observed in this study. However, we recognize that the sample size for this phase II study is underpowered to detect a statistically relevant association, and therefore we plan to incorporate these findings as the foundation for larger studies in the future.

2.4.3 Role of CD_4^+ Infiltrating T-cells in the response of SCCA of the anal canal to nivolumab

2.4.3.1 Rationale

The associations between the presence of CD_4^+ infiltrating T cells and (1) responses to nivolumab and (2) survival outcomes have never been described in a prospective study for patients with metastatic SCCA of the anal canal. This analysis would not only inform on the relevance of these immune cells in this particular disease for the first time, but could also be used in understanding further the role of CD_4^+ infiltrating T cells as a potential biomarker for immune checkpoint inhibitors like nivolumab across various HPV-related malignancies.

2.4.3.2 Background

A correlation between the presence of CD_4^+ infiltrating T-cells and improved clinical outcomes has been previously described in a cohort of patients with squamous cell carcinoma of the head and neck [114], an important finding given that a large proportion of these patients have tumors driven by HPV infection. However, whether or not an association may exist between the presence of CD_4^+ infiltrating T-cells and (1) response to nivolumab therapy and (2) survival outcomes remains unknown for patients with metastatic SCCA of the anal canal.

2.4.3.3 Hypothesis

Given that CD_4^+ infiltrating T-cells assist CD_8^+ infiltrating T-cells in maintaining an anti-tumor response in the tumor microenvironment, we hypothesize that patients with metastatic SCCA of the anal canal whose tumors contain CD_4^+ infiltrating T cells may respond to nivolumab in this study. However, we recognize that the sample size for this phase II study is underpowered to detect a statistically relevant association, and therefore we plan to incorporate these findings as the foundation for larger studies in the future. We will also study the potential of antigen-specific CD4+ T regulatory cells to decrease the response to nivolumab in this study (see 2.3.4., below).

2.4.4 Role of anti-HPV CD_8^+ TILs, CD_4^+ TILs and anti-HPV regulatory T cells in the response of SCCA of the anal canal to nivolumab

2.4.4.1 Rationale

While the presence of CD_8^+ and CD_4^+ infiltrating T-cells appear important in the anti-tumor response for HPV-associated malignancies, it is important to ensure that these immune cells are specific to tumor cells expressing immunogenic, viral neoepitopes introduced on the cell surface following incorporation of viral HPV DNA into the host cell genome.

2.4.4.2 Background

The presence of intratumoral anti-HPV T-cells has been described in other HPV-associated malignancies like cervical cancer and squamous cell carcinoma of the head and neck [94,114] and are associated with locoregional control of disease. However, any associations between the presence of immune cells specific to HPV-infected cells and clinical outcomes have not been described for patients with metastatic SCCA of the anal canal.

2.4.4.3 Hypothesis

We hypothesize that patients with metastatic SCCA of the anal canal whose blood samples show an increase in frequency of anti-HPV CD_8^+ and/or CD_4^+ infiltrating T-cells may respond to nivolumab in this study. Likewise, no change or a decrease in the frequency of anti-HPV CD_8^+ and/or CD_4^+ infiltrating T-cells may be associated with a lack of response to nivolumab for patients with previously treated metastatic SCCA of the anal canal. We also hypothesize that a negative correlation may exist between the presence of anti-HPV regulatory T cells and response to nivolumab. However, we recognize that the sample size for this phase II study is underpowered to detect a statistically relevant association, and therefore we plan to incorporate these findings as the foundation for larger studies in the future.

2.5 Correlative Studies Background (Part B)

In NCI9673, higher tumor PD-1:PD-L1 expression on activated CD8+ T cells at baseline were associated with response to nivolumab: Twelve patients at MD Anderson who enrolled on NCI 9673 participated in an optional translational component for collection of two paired tumor biopsies, one pretreatment and the latter after 3 doses of nivolumab. Nine of these patients had adequate tissue available for both biopsies. Tissue was analyzed by immunohistochemistry and by flow cytometry to compare expression levels of various immune makers for patients with and without responses to nivolumab. Patients with responses to nivolumab demonstrated higher expression in baseline biopsies of CD8 (P=.01) and granzyme B (P=.005) by immunohistochemistry[56]. In addition, responders had higher levels of baseline PD-1 and PD-L1 expression by immunohistochemistry (P=.02 and P=.005, respectively) and by flow cytometry (P=.004 and P=.04, respectively) when compared to non-responders. Higher pretreatment levels of CD8+ T cells expressing both PD-1 and PD-L1 (Figure 2) at baseline were seen among the responders to single agent nivolumab. In addition, when flow cytometry was used to quantify CD8+ T cells co-expressing multiple inhibitory immune checkpoint proteins, higher levels of

dual PD1+/TIM3+ and dual PD1+/LAG3+ populations were seen among the patients with responses to anti-PD1 therapy (Figures 3). These cells trended down after three doses of nivolumab as seen at the time of second biopsy. Collectively, these results support the hypothesis that (1) the presence of a PD-1:PD-L1 axis is amenable to be blocked and (2) the presence of activated T cells amid a more inflamed tumor at baseline may together be associated with a response to anti-PD-1 therapies targeting immune checkpoint blockade.

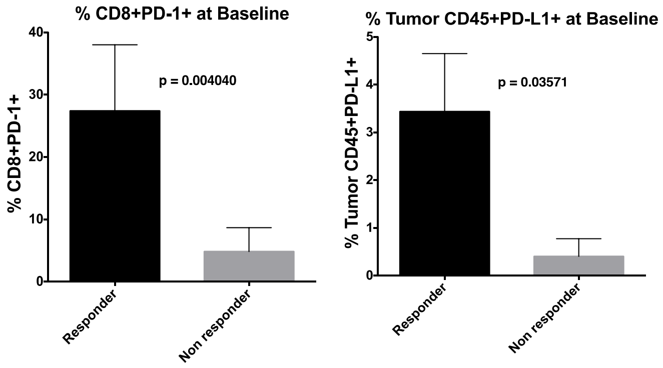

Figure 2: Increased CD8 + T cells expressing both PD-1 and PD-L1 in responders vs. nonresponders

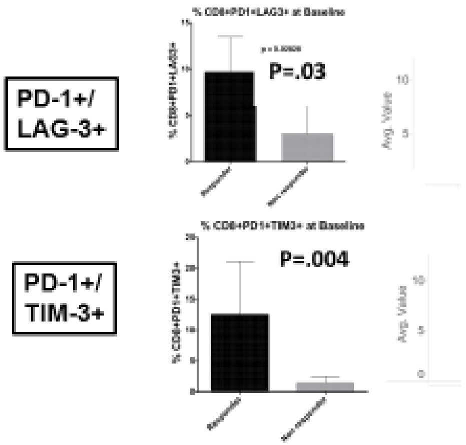

Figure 3: Increased dual PD1+/TIM3+ and PD1+/LAG3+ populations in responders vs. nonresponders

3. PATIENT SELECTION

3.1 Eligibility Criteria (Parts A+B)

3.1.1 Patients must have histologically or cytologically confirmed previously treated metastatic squamous cell carcinoma of the anal canal.

3.1.2 Patients must have measurable disease according to the standard RECIST version 1.1. See Section 12 for the evaluation of measurable disease. CT scans or MRIs used to assess the measurable disease must have been completed within 28 days prior to study drug initiation.

3.1.3 Patients must have been treated with at least one prior systemic treatment for incurable advanced or metastatic SCCA of the anal canal. Prior treatment for metastatic disease is not required for patients who develop new metastatic lesions during or within 6 months of completion of chemoradiation for limited-stage disease. Patients who receive chemotherapy for incurable advanced or metastatic SCCA of the anal canal must wait a minimum ≥ 28 days (6 weeks for nitrosoureas or mitomycin C) after the date of completion of chemotherapy prior to initiating treatment with nivolumab on this study. Patients who undergo palliative radiotherapy to a site of tumor must wait a minimum ≥ 28 days from the date of completion of radiotherapy prior to initiating treatment with nivolumab (Part A and B) or nivolumab +/- Ipilimumab (Part B) on this study.

3.1.4 Patients must be of age ≥18 years at the time of study registration. Because no dosing or adverse event data are currently available on the use of nivolumab in patients <18 years of age, children are excluded from this study, but will be eligible for future pediatric trials.

3.1.5 ECOG performance status 0 or 1 (Karnofsky ≥80%, see Appendix A).

3.1.6 Patients must have normal organ and marrow function as defined below:

- leukocytes ≥2,000/mcL
- absolute neutrophil count ≥1,500/mcL
- hemoglobin ≥ 9.0 gm/dL
- platelets ≥100,000/mcL
- total bilirubin ≤1.5× institutional upper limit of normal (ULN) (except patients with Gilbert Syndrome, who can have total bilirubin <3.0 mg/dL)
- AST(SGOT)/ALT(SGPT) ≤ 2.5× ULN
- Serum creatinine ≤1.5× ULN

  OR
- creatinine clearance (CrCl) ≥50 mL/min (if using the Cockcroft-Gault formula below):

$$\textit{Female}\ \text{CrCl} = \frac{(140 - \text{age in years}) \times \text{weight in kg} \times 0.85}{72 \times \text{serum creatinine in mg/dL}}$$

$$\textit{Male}\ \text{CrCl} = \frac{(140 - \text{age in years}) \times \text{weight in kg} \times 1.00}{72 \times \text{serum creatinine in mg/dL}}$$

3.1.7 Before study enrollment, women of child bearing potential must be advised of the importance of avoiding pregnancy during study participation and the potential risk factors for an unintentional pregnancy. The subject must sign an informed consent form documenting this discussion

Part A+B: The effects of nivolumab and ipilimumab on the developing human fetus are unknown. For this reason, women of child-bearing potential (WOCBP) and men must agree to use adequate contraception (hormonal or barrier method of birth control; abstinence) prior to study entry and for the duration of study participation. Women of childbearing potential **MUST** have a negative serum or urine pregnancy test (minimum sensitivity 25 IU/L or equivalent units of HCG) within 24 hours prior to the start of nivolumab with or without ipilimumab. The minimum sensitivity of the pregnancy test must be 25 IU/L or equivalent units of HCG. If the pregnancy test is positive, the subject must not receive nivolumab with or without ipilimumab and must not be enrolled in the study.

Women of childbearing potential (WOCBP) is defined as any female who has experienced menarche and who has not undergone surgical sterilization (hysterectomy or bilateral oophorectomy) or who is not postmenopausal. Menopause is defined clinically as 12 months of amenorrhea in a woman over 45 in the absence of other biological or physiological causes.

WOCBP receiving nivolumab (Parts A+B) or nivolumab and ipilimumab (Part B) will be instructed to adhere to contraception for a period of 5 months after the last dose of investigational product. Men receiving nivolumab (Parts A+B) or nivolumab and ipilimumab (Part B only) and who are sexually active with WOCBP will be instructed to adhere to contraception for a period of 7 months after the last dose of investigational product. These durations have been calculated using the upper limit of the half-life for nivolumab (25 days) and are based on the protocol requirement that WOCBP use contraception for 5 half-lives plus 30 days and men who are sexually active with WOCBP use contraception for 5 half-lives plus 90 days.

Should a woman become pregnant or suspect she is pregnant while she or her partner is participating in this study, she (or the participating partner) should inform the treating physician immediately.

If, following initiation of the investigational product, it is subsequently discovered that a study subject is pregnant or may have been pregnant at the time of investigational product exposure, including during at least 6 half-lives after product administration, the investigational product will be permanently discontinued in an appropriate manner (*e.g.*, dose tapering if necessary for subject safety). The investigator must immediately notify BMS of this event and record the pregnancy on the Pregnancy Surveillance Form (not an SAE form). Initial information on a pregnancy must be reported immediately to BMS,

and the outcome information provided once the outcome is known. Completed Pregnancy Surveillance Forms must be forwarded to BMS according to SAE reporting procedures.

Any pregnancy that occurs in a female partner of a male study participant should be reported to the sponsor. Information on this pregnancy will be collected on the Pregnancy Surveillance Form.

Protocol-required procedures for study discontinuation and follow-up must be performed on the subject unless contraindicated by pregnancy (*e.g.*, X-ray studies). Other appropriate pregnancy follow-up procedures should be considered if indicated. In addition, the investigator must report and follow-up on information regarding the course of the pregnancy, including perinatal and neonatal outcome. Infants should be followed for a minimum of 8 weeks.

3.1.8 Ability to understand and the willingness to sign a written informed consent document.

3.1.9 Brain metastases are allowed if they have been adequately treated with radiotherapy or surgery and have been stable for at least three months prior to registration. Eligible subjects should be neurologically asymptomatic. There is no magnetic resonance imaging (MRI) evidence of progression for a minimum of 4 weeks after treatment is complete and within 28 days prior to the first dose of nivolumab administration. There must also be no requirement for immunosuppressive doses of systemic corticosteroids (>10 mg/day prednisone equivalents) for at least 2 weeks prior to study drug administration.

3.1.10 Willingness for evaluation of cardiac function including EKG and ECHO cardiogram for any patients with a history of CHF or at risk because of underlying cardiovascular disease or exposure to cardiotoxic drugs as clinically indicated.

3.1.11 All patients must be willing to undergo testing for HIV testing if not tested within the past 6 months.

3.1.12 If HIV+ positive, all patients infected with Human Immunodeficiency Virus (HIV) may be eligible for study provided that their CD4+ count ≥ 300/µL; their viral load is undetectable; they are currently receiving Highly Active Antiretroviral Therapy (HAART).

3.1.13 All HIV+ patients will be under the care of an Infectious Diseases specialist. If a relationship with an Infectious Diseases specialist is not established, Infectious Disease specialist will be consulted. Records of all viral counts and peripheral T-cell counts must be sent to the Study Coordinator in order to follow these values over the course of treatment.

3.1.14 All patients must be willing to be tested for Hepatitis screening. Patients co-infected with hepatitis B virus and/or hepatitis C virus may be included in this study provided that their liver function tests remain within the limits listed above. Patients must be followed by a hepatologist during the course of this study.

3.2 Exclusion Criteria (Parts A+B)

3.2.1 Patients who have had chemotherapy or radiotherapy within 4 weeks (6 weeks for nitrosoureas or mitomycin C) prior to entering the study or those who have not recovered from adverse events (AEs) due to agents administered more than 4 weeks earlier (i.e., grade ≥ 2 AE present). Palliative (limited-field) radiation therapy is permitted, as long as the lesion being considered for palliative radiation is not a target lesion.

3.2.2 Patients who are receiving any other investigational agents.

3.2.3 Patients should be excluded if they have had prior treatment with an anti-PD-1, anti-PD-L1, anti-PD-L2, anti-CTLA-4 antibody, or any other antibody or drug specifically targeting T-cell co-stimulation or immune checkpoint pathways.

3.2.4 History of allergic reactions attributed to compounds of similar chemical or biologic composition to nivolumab (Parts A+ B) and/or ipilimumab (Part B).

3.2.5 History of severe hypersensitivity reaction to any monoclonal antibody.

3.2.6 Uncontrolled intercurrent illness including, but not limited to, ongoing or active infection, symptomatic congestive heart failure, unstable angina pectoris, cardiac arrhythmia, or psychiatric illness/social situations that would limit compliance with study requirements.

3.2.7 Patients with active autoimmune disease or history of autoimmune disease that might recur, which may affect vital organ function or require immune suppressive treatment including chronic prolonged systemic corticosteroids (defined as corticosteroid use of duration one month or greater), should be excluded. These include but are not limited to patients with a history of immune related neurologic disease, multiple sclerosis, autoimmune (demyelinating) neuropathy, Guillain-Barre syndrome, myasthenia gravis; systemic autoimmune disease such as SLE, connective tissue diseases, scleroderma, inflammatory bowel disease (IBD), Crohn's, ulcerative colitis, and patients with a history of toxic epidermal necrolysis (TEN), Stevens-Johnson syndrome, or anti-phospholipid syndrome should be excluded because of the risk of recurrence or exacerbation of disease.

3.2.8 Patients should be excluded if they have a condition requiring systemic treatment with either corticosteroids (>10 mg daily prednisone equivalents) or other immunosuppressive medications within 14 days of study drug administration. Inhaled or topical steroids and adrenal replacement doses ≤10 mg daily prednisone equivalents are permitted in the absence of active autoimmune disease. Patients are permitted to use topical, ocular, intra-articular, intranasal, and inhalational corticosteroids (with minimal systemic absorption). Physiologic replacement doses of systemic corticosteroids are permitted, even if ≤10 mg/day prednisone equivalents. A brief course of corticosteroids for prophylaxis (*e.g.*, contrast dye allergy) or for treatment of non-autoimmune conditions (*e.g.*, delayed-type hypersensitivity reaction caused by contact allergen) is permitted.

3.2.9 No other prior malignancy is allowed except for the following: adequately treated basal cell or squamous cell skin cancer, *in situ* cervical cancer, adequately treated Stage I or II cancer from which the patient is currently in complete remission, or any other cancer from which the patient has been disease free for at least three years.

3.3 Inclusion of Women and Minorities

Both men and women of all races and ethnic groups are eligible for this trial.

4. REGISTRATION PROCEDURES

4.1 Investigator and Research Associate Registration with CTEP

4.1.1 CTEP Registration Procedures

Food and Drug Administration (FDA) regulations and National Cancer Institute (NCI) policy require all individuals contributing to NCI-sponsored trials to register and to renew their registration annually. To register, all individuals must obtain a Cancer Therapy Evaluation Program (CTEP) Identity and Access Management (IAM) account (https://ctepcore.nci.nih.gov/iam). In addition, persons with a registration type of Investigator

(IVR), Non-Physician Investigator (NPIVR), or Associate Plus (AP) (i.e., clinical site staff requiring write access to OPEN or RAVE or acting as a primary site contact) must complete their annual registration using CTEP's web-based Registration and Credential Repository (RCR) (https://ctepcore.nci.nih.gov/rcr). Documentation requirements per registration type are outlined in the table below.

Documentation Required	IVR	NPIVR	AP	A
FDA Form 1572	✔	✔		
Financial Disclosure Form	✔	✔	✔	
NCI Biosketch (education, training, employment, license, and certification)	✔	✔	✔	
HSP/GCP training	✔	✔	✔	
Agent Shipment Form (if applicable)	✔			
CV (optional)	✔	✔	✔	

4.1.2 CTEP Associate Registration Procedures / CTEP-IAM Account

An active CTEP-IAM user account and appropriate RCR registration is required to access all CTEP and CTSU (Cancer Trials Support Unit) websites and applications. In addition, IVRs and NPIVRs must list all clinical practice sites and IRBs covering their practice sites on the FDA Form 1572 in RCR to allow the following:

- Added to a site roster
- Assigned the treating, credit, consenting, or drug shipment (IVR only) tasks in OPEN
- Act as the site-protocol PI on the IRB approval
- Assigned the Clinical Investigator (CI) role on the Delegation of Tasks Log (DTL).

4.1.3 For Questions and Support

Additional information can be found on the CTEP website at <https://ctep.cancer.gov/investigatorResources/default.htm>. For questions, please contact the RCR ***Help Desk*** by email at < RCRHelpDesk@nih.gov >.

4.2 Site Registration

Each investigator or group of investigators at a clinical site must obtain Institutional Review Board (IRB) approval for this protocol and submit all required regulatory documents to the CTSU Regulatory Office before they can be approved to enroll patients. Assignment of site registration status in the CTSU Regulatory Support System (RSS) uses extensive data to make a determination of whether a site has fulfilled all regulatory criteria including but not limited to the following:

- An active Federal Wide Assurance (FWA) number
- An active roster affiliation with the Lead Network or a participating organization
- A valid IRB approval
- Compliance with all protocol specific requirements

In addition, the site-protocol Principal Investigator (PI) must meet the following criteria:

- Active registration status
- The IRB number of the site IRB of record listed on their Form FDA 1572
- An active status on a participating roster at the registering site

Sites participating on the NCI CIRB initiative that are approved by the CIRB for this study are not required to submit IRB approval documentation to the CTSU Regulatory Office. For sites using the CIRB, IRB approval information is received from the CIRB and applied to the RSS in an automated process. Signatory Institutions must submit a Study Specific Worksheet for Local Context (SSW) to the CIRB via IRBManager to indicate their intent to open the study locally. The CIRB's approval of the SSW is then communicated to the CTSU Regulatory Office. In order for the SSW approval to be processed, the Signatory Institution must inform the CTSU which CIRB-approved institutions aligned with the Signatory Institution are participating in the study.

4.2.1 Downloading Regulatory Documents

- Go to https://www.ctsu.org and log in using your CTEP IAM username and password
- Click on the Protocols tab in the upper left of your screen
- Either enter the protocol # in the search field at the top of the protocol tree, or
- Click on the By Lead Organization folder to expand, then select LAO-TX035, and **protocol #9673**.
- Click on LPO Documents, select the Site Registration documents link, and download and complete the forms provided. (Note: For sites under the CIRB initiative, IRB data load to RSS as described above.)

4.2.2 Submitting Regulatory Documents

Submit required forms and documents to the CTSU Regulatory Office, where they will be entered and tracked in the CTSU RSS.

Regulatory Submission Portal: www.ctsu.org (members' area) → Regulatory Tab →Regulatory Submission

When applicable, original documents should be mailed to:
CTSU Regulatory Office
1818 Market Street, Suite 3000
Philadelphia, PA 19103

Institutions with patients waiting that are unable to use the Portal should alert the CTSU Regulatory Office immediately at 1-866-651-2878 in order to receive further instruction and support

- IRB approval (For sites not participating via the NCI CIRB; local IRB documentation, an IRB-signed CTSU IRB Certification Form, Protocol of Human Subjects Assurance Identification/IRB Certification/Declaration of Exemption Form, or combination is accepted)

4.2.3 Checking **Site** Registration Status

You can verify your site registration status on the members' section of the CTSU website.

- Go to https://www.ctsu.org and log in to the members' area using your CTEP-IAM username and password
- Click on the Regulatory tab at the top of your screen
- Click on the Site Registration tab
- Enter your 5-character CTEP Institution Code and click on Go

Note: The status given only reflects compliance with IRB documentation and institutional compliance with protocol-specific requirements as outlined by the Lead Network. It does not reflect compliance with protocol requirements for individuals participating on the protocol or the enrolling investigator's status with the NCI or their affiliated networks.

4.3 Patient Registration

4.3.1 OPEN / IWRS

Patient enrollment will be facilitated using the Oncology Patient Enrollment Network (OPEN). OPEN is a web-based registration system available to users on a 24/7 basis. It is integrated with the CTSU Enterprise System for regulatory and roster data interchange and with the Theradex Interactive Web Response System (IWRS) for retrieval of patient registration/randomization assignment. Patient enrollment data entered by Registrars in OPEN / IWRS will automatically transfer to the NCI's clinical data management system, Medidata Rave.

The OPEN system will provide the site with a printable confirmation of registration and treatment information. Please print this confirmation for your records.

4.3.2 OPEN/IWRS User Requirements

OPEN/IWRS users must meet the following requirements:

- Have a valid CTEP-IAM account (*i.e.*, CTEP username and password).
- To enroll patients: Be on an ETCTN Corresponding or Participating Organization roster with the role of Registrar. Registrars must hold a minimum of an AP registration type.
- To access cohort management: Be identified to Theradex as the "Client Admin" for the study.
- Have regulatory approval for the conduct of the study at their site.

Prior to accessing OPEN/IWRS, site staff should verify the following:

- All eligibility criteria have been met within the protocol stated timeframes. Site staff should use the registration forms provided on the CTSU web site as a tool to verify eligibility.
- If applicable, all patients have signed an appropriate consent form and HIPAA authorization form.

4.3.3 OPEN/IWRS Questions?

Further instructional information on OPEN is provided on the OPEN tab of the CTSU website at https://www.ctsu.org or at https://open.ctsu.org. For any additional questions contact the CTSU Help Desk at 1-888-823-5923 or ctsucontact@westat.com.

Theradex has developed a Slot Reservations and Cohort Management User Guide, which is available on the Theradex website: http://www.theradex.com/clinicalTechnologies/?National-Cancer-Institute-NCI-11

This link to the Theradex website is also on the CTSU website OPEN tab. For questions about the use of IWRS for slot reservations, contact the Theradex Helpdesk: 609-619-7862 or

Theradex main number 609-799-7580; CTMSSupport@theradex.com.

4.4 General Guidelines

Following registration, patients should begin protocol treatment within 7 days. Issues that would cause treatment delays should be discussed with the Principal Investigator. If a patient does not receive protocol therapy following registration, the patient's registration on the study may be canceled. The Study Coordinator should be notified of cancellations as soon as possible.

5. TREATMENT PLAN

5.1 Treatment with nivolumab (Parts A+B)

Treatment will be administered on an outpatient basis. For patients randomized to Part B, nivolumab will be administered prior to ipilimumab. Reported adverse events and potential risks are described in Section 7. Appropriate dose modifications are described in Section 6. No investigational or commercial agents or therapies other than those described below may be administered with the intent to treat the patient's malignancy.

5.1.1 Nivolumab

There are no premedications recommended for nivolumab on the first dose. Subjects should be carefully monitored for infusion reactions during nivolumab administration. If an acute infusion reaction is noted, subjects should be managed according to Section 5.8.

5.1.2 Other Modalities or Procedures

N/A

5.2 Nivolumab Administration

Part A: Nivolumab will be given intravenously every two weeks (±3 days) at a dose of 3 mg/kg IV. Patients may be dosed no fewer than 12 days from the previous dose of drug.

Part B: Nivolumab will be given intravenously every four weeks (±3 days) at a dose of at a flat dose of 480 mg IV over 30 minutes. Patients may be dosed no fewer than 12 days from the previous dose of drug.

Nivolumab is to be administered as an IV infusion through a 0.2-micron to 1.2- micron pore size, low-protein binding (polyethersulfone membrane) in-line filter at the protocol-specified doses and infusion times. It is not to be administered as an IV push or bolus injection. When the dose is based on patient weight (ie, mg/kg), nivolumab injection can be infused undiluted (10 mg/mL) or diluted with 0.9% Sodium Chloride Injection, USP or 5% Dextrose Injection, USP to protein concentrations as low as 0.35 mg/mL. **When the dose is fixed** (eg, **240 mg, 360 mg, or 480 mg flat dose), nivolumab injection can be infused undiluted or diluted** so as **not to exceed a total infusion volume of 160 mL.**

For patients weighing less than 40 kilograms (kg), the total volume of infusion must not exceed 4 mL per kg of patient weight.

5.3 General Concomitant Medication and Supportive Care Guidelines (Parts A+B)

Although there is not a potential for interaction of nivolumab with other concomitantly administered drugs through the cytochrome P450 system, the case report form must still capture the concurrent use of all other drugs, over-the-counter medications, or alternative therapies.

5.4 Duration of Therapy (Parts A+B)

In the absence of treatment delays due to adverse event(s), treatment may continue until one of the following criteria applies:

- Disease progression,
- Intercurrent illness that prevents further administration of treatment,
- Unacceptable adverse event(s) which include the following (see also Section 7 and specific algorithms in Appendix D):
  - Any grade 4 events except as noted below.
  - Grade 3 drug-related autoimmune or inflammatory events including uveitis, pneumonitis, diarrhea, colitis, neurologic adverse events, hypersensitivity reaction, or infusion reaction of any duration requires discontinuation except as noted below:
    - Any other grade 3 non-skin, drug-related AE lasting < 7 days including fatigue.
    - Any grade 3 or 4 drug-related laboratory abnormality or electrolyte abnormality, not associated with underlying organ pathology that does not require treatment except for electrolyte replacements **does not** require treatment discontinuation.
    - Grade 3 or 4 amylase or lipase abnormalities that are not associated with diabetes mellitus (DM), associated liver or gall bladder inflammation or clinical manifestations of pancreatitis and which decrease to ≤ Grade 2 within 1 week of onset **may** resume study treatment when resolved.
  - Any grade 2 drug-related uveitis or eye pain or blurred vision that does not respond to topical therapy and does not improve to Grade 1 severity within the re-treatment period OR requires systemic treatment.

- Patients requiring > two dose delays for the same type of event should go off protocol therapy.

- Any adverse event, laboratory abnormality, or intercurrent illness which, in the judgment of the investigator, presents a substantial clinical risk to the subject with continued study drug dosing

- Any dosing interruption lasting > 6 weeks, with the following exceptions:

  - Patients being tapered after high dose corticosteroids over one month followed by a two-week observation period will be allowed an additional two weeks to restart treatment (a maximum eight week interruption). Dosing interruptions >6 weeks that occur for non-drug-related reasons may be allowed if approved by the Investigator. Prior to re-initiating treatment in a subject with a dosing interruption lasting >6 weeks, the Principal Investigator must be consulted.

- Tumor assessments should continue as per protocol even if dosing is interrupted.

- Any patients who require additional immune suppressive treatment beyond steroids should go off study treatment.

- Patient decides to withdraw from the study.

- General or specific changes in the patient's condition render the patient unacceptable for further treatment in the judgment of the investigator.

5.5 Duration of Follow Up

All patients will be followed for adverse events for 100 days after last dose of nivolumab +/- ipilimumab. Patients who discontinue treatment for unacceptable adverse event(s) will be followed until resolution or stabilization of the adverse event. If a patient stops treatment due to unacceptable adverse event(s) but has not demonstrated disease progression, then the patient will be followed with imaging studies every 6 weeks until the time of progression radiographically according to RECIST 1.1 criteria. In the event that a radiographic response is detected, then this event will be included as a response in the final analysis, and the time of progression used in calculation of the survival analysis.

Patients will be followed for survival status every 3 months for 2 years after treatment discontinuation or until death, whichever occurs first.

5.6 Criteria for Removal from Study (Parts A+B)

Patients will be removed from study when any of the applicable criteria, including progressive disease, adverse events, patient withdrawal or inability to follow study protocol as listed in

Section 5.4. The reason for study removal and the date the patient was removed must be documented in the Case Report Form.

5.7 Criteria to Resume Treatment (Parts A+B)

Some patients may continue to benefit from treatment, maintaining or improving responses after progression including those treated with steroids.

Restarting applies only to grade 2 events and some grade 3 events (skin rash and thyroiditis).

For non-autoimmune or non-inflammatory events patients may resume treatment with study drug when the drug-related AE(s) resolve to Grade ≤1 or baseline value, with the following exceptions:

- Patients may resume treatment in the presence of Grade 2 fatigue
- Evaluation to exclude any additional immune mediated events endocrine, GI, and liver / pancreas function as clinically indicated must be made prior to restarting.
- Non-drug-related toxicity including hepatic, pulmonary toxicity, diarrhea, or colitis, must have resolved to baseline before treatment is resumed.

If the criteria to resume treatment are met, the patient should restart treatment at the earliest convenient point that is within the six week delay period.

If treatment is delayed for >6 weeks, (>8 weeks for patients on a steroid taper), the patient must be permanently discontinued from study therapy, except as specified in Section 5.4 (Duration of Therapy).

For patients treated with corticosteroids:

Grade 2 events must resolve to ≤ Grade 1 before considering retreatment.

All patients treated with steroids for grade ≥2 events should have study treatment held until resolution to ≤ Grade 1 for at least 2 weeks following complete removal from steroid treatment except for maintenance replacement doses for adrenal insufficiency (preferably no greater than 10mg prednisone equivalent daily).

All patients treated with steroids for grade ≥3 events should have study treatment discontinued. Patients with grade 3 thyroiditis and skin rash may continue therapy as for grade 2 events with resolution and stable replacement treatment.

Patients with hepatitis, pancreatitis, pneumonitis, and colitis are at risk for exacerbation with retreatment if there is residual inflammation and should resolve to Grade 0 or baseline before retreatment. Baseline can mean the initial grade *i.e.* grade <1 where permitted on study.

Patients with thyroiditis or hypopituitarism who are stable as above may be restarted with replacement hormones including thyroid hormone and physiologic doses only of corticosteroids.

Please note that grading and for hypophysitis with symptoms of headache, visual or neurologic changes or radiologic evidence of pituitary enlargement and other CNS events such as aseptic meningitis or encephalitis should be considered grade 3 events.

New immune related events or exacerbation of existing events during steroid treatment or taper suggest the presence of ongoing immune activation and should require permanent discontinuation of nivolumab.

A patient who is treated with steroids, evaluated, and found to not have an autoimmune or inflammatory event requiring steroid treatment, may be restarted if asymptomatic off steroids for 2 weeks and other restarting criteria are met.

Prior to starting corticosteroids or hormone replacement for any reason, appropriate endocrine testing including cortisol, ACTH, TSH and T4 must be drawn if clinically feasible to document baseline function and distinguish the pituitary from peripheral organ dysfunction and later from steroid (or thyroid) treatment associated ACTH (or TSH) suppression. Steroids should be started prior to obtaining results based on clinical indications.

5.8 Treatment of Nivolumab-Related Infusion Reactions (Parts A+B)

Since nivolumab contains only human immunoglobulin protein sequences, it is unlikely to be immunogenic and induce infusion or hypersensitivity reactions. However, if such a reaction were to occur, it might manifest with fever, chills, rigors, headache, rash, urticaria, angioedema, pruritis, arthralgias, hypo- or hypertension, bronchospasm, or other symptoms.

All Grade 3 or 4 infusion reactions should be reported as an SAE if criteria are met. Infusion reactions should be graded according to NCI CTCAE version 5.0 guidelines.

Treatment recommendations are provided below and may be modified based on local treatment standards and guidelines as medically appropriate:

For Grade 1 symptoms: (Mild reaction; infusion interruption not indicated; intervention not indicated)

Remain at bedside and monitor subject until recovery from symptoms. Infusion rate may be slowed. If the infusion is interrupted, then restart the infusion at 50% of the original infusion rate when symptoms resolve; if no further complications ensue after 30 minutes, the rate may be increased to 100% of the original infusion rate. Monitor patient closely.

The following prophylactic premedications are recommended for future infusions: diphenhydramine 50 mg (or equivalent) and/or paracetamol 325 to 1000 mg (acetaminophen) at least 30 minutes before additional nivolumab administrations, slowing infusion rate as above.

For Grade 2 symptoms: (Moderate reaction requires therapy or infusion interruption but responds promptly to symptomatic treatment [*e.g.*, antihistamines, non-steroidal anti-inflammatory drugs, narcotics, corticosteroids, bronchodilators, IV fluids]; close observation for

recurrence and treatment medications may need to be continued for 24-48 hours).

Stop the nivolumab infusion, begin an IV infusion of normal saline, and treat the subject with diphenhydramine 50 mg IV (or equivalent) and/or paracetamol 325 to 1000 mg (acetaminophen); remain at bedside and monitor patient until resolution of symptoms. Corticosteroid or bronchodilator therapy may also be administered as appropriate. If the infusion is interrupted, then restart the infusion at 50% of the original infusion rate when symptoms resolve; if no further complications ensue after 30 minutes, the rate may be increased to 100% of the original infusion rate. Monitor patient closely. If symptoms recur, then no further nivolumab will be administered at that visit. Administer diphenhydramine 50 mg IV, and remain at bedside and monitor the patient until resolution of symptoms. The amount of study drug infused must be recorded on the electronic case report form (eCRF). The following prophylactic premedications are recommended for future infusions: diphenhydramine 50 mg (or equivalent) and (acetaminophen) (or paracetamol) 325 to 1000 mg should be administered at least 30 minutes before additional nivolumab administrations. If necessary, corticosteroids (recommended dose: up to 25 mg of IV hydrocortisone or equivalent) may be used.

For Grade 3 symptoms: (Severe reaction), Grade 3 symptoms: prolonged [*i.e.*, not rapidly responsive to symptomatic medication and/or brief interruption of infusion]; recurrence of symptoms following initial improvement; hospitalization indicated for other clinical sequelae [*e.g.*, renal impairment, pulmonary infiltrates]).

Grade 4 symptoms: (life threatening; pressor or ventilatory support indicated).

Immediately discontinue infusion of nivolumab. Begin an IV infusion of normal saline, and treat the subject as follows. Recommend bronchodilators, epinephrine 0.2 to 1 mg of a 1:1,000 solution for subcutaneous administration or 0.1 to 0.25 mg of a 1:10,000 solution injected slowly for IV administration, and/or diphenhydramine 50 mg IV with methylprednisolone 100 mg IV (or equivalent), as needed.

Patient should be monitored until the investigator is comfortable that the symptoms will not recur. Nivolumab will be permanently discontinued. Investigators should follow their institutional guidelines for the treatment of anaphylaxis. Remain at bedside and monitor patient until recovery from symptoms. In the case of late-occurring hypersensitivity symptoms (*e.g*., appearance of a localized or generalized pruritis within 1 week after treatment), symptomatic treatment may be given (*e.g*., oral antihistamine, or corticosteroids).

Please note that late occurring events including isolated fever and fatigue may represent the presentation of systemic inflammation. Please evaluate accordingly.

5.9 Ipilimumab Administration (Part B)

Ipilimumab will be administered IV over 30 minutes at 1 mg/kg every 8 weeks until progression, unacceptable toxicity, or other reasons specified in the protocol. The dose of ipilimumab does not have to be recalculated at each cycle unless patient has a weight change greater than 10% at the time, as per FDA.

Ipilimumab is given undiluted or further diluted in 0.9% NaCl Injection, USP or 5% Dextrose Injection, USP in concentrations between 1 mg/mL and 4 mg/mL. Ipilimumab is stable in a polyvinyl chloride (PVC), non-PVC/non DEHP (di-(2-ethylhexyl) phthalate) IV bag or glass container up to 24 hours refrigerated at (2^0 to 8^0 C) or at room temperature/ room light.

Can use a volumetric pump to infuse ipilimumab at the protocol-specific dose(s) and rate(s) via a PVC IV infusion set with an in-line, sterile, non-pyrogenic, low-protein-binding filter (0.2 micron to 1.2 micron).

Recommended safety measures for preparation and handling include protective clothing, gloves, and safety cabinets.

5.9.1 Dose Delays

Decisions to skip an ipilimumab dose must be made on specified safety criteria. Treatment with ipilimumab will be skipped or discontinued if the subject experiences at least one adverse event, specified below, considered by the investigator to be **"possibly," probably," or "definitely" related to ipilimumab treatment.**

Any adverse event that will prompt a skipped dose or discontinuation of ipilimumab must be reported.

Co-investigators and/or research team members at every participating site will participate in a teleconference once monthly with the PI and with the NCI to review AE's over the course of the study.

The investigator should contact the study PI or drug monitor to discuss any questions.

The following criteria will be used to determine dose skipping, restarting doses, or discontinuing ipilimumab.

<u>It is necessary to avoid study drug dosing and initiate appropriate evaluation and/or treatment for the following adverse events:</u>

- Any ≥ grade 3 skin related adverse event regardless of causality.
- Any ≥ grade 2 non-skin related adverse event (including immune-mediated adverse reactions), except for easily correctly laboratory abnormalities that do not reflect underlying organ pathology.
- Any ≥ grade 3 laboratory abnormality.
- Any adverse event, laboratory abnormality or intercurrent illness that, in the judgment of the investigator, presents a substantial clinical risk to the subject with continued dosing.

It may be necessary to hold study drug to evaluate Grade 1 events that suggest ongoing or incipient autoimmune disease including GI toxicity, diarrhea, pancreatitis, hepatitis, pituitary insufficiency, early evidence of neurologic events, skin toxicity until diagnosis and progression are determined.

3 Criteria to Resume Treatment

<u>Ipilimumab may be restarted if/when the adverse event(s) resolve(s) to severity or returns to baseline within 2 weeks of initial dose administration:</u>

- If the adverse event has been determined not be related to ipilimumab or is not an autoimmune/inflammatory event. If >1 dose is to be skipped or > 2 week delay is expected due to current events not related to study the dosing schedule modifications must be discussed with the principal investigator prior to implementation.
- If the adverse event has resolved to **<u>≤ grade 1</u>**, ipilimumab dosing may be restarted at the next scheduled time point per protocol. Please follow guidelines for specific events. Please note that re-initiating treatment may be associated with recurrence or exacerbation of autoimmune or inflammatory events. In some instances clinical resolution of events such as colitis may be associated with residual pathologic changes and should require evaluation of complete resolution prior to restarting therapy.
- If the adverse event has not resolved in the protocol-specified dosing window (3 weeks [±3 days]), the next scheduled dose will be skipped and dosing will be resumed at the subsequently scheduled dose.
- Events which require intervention with immunosuppressant therapy, steroids, surgery, or hormone replacement generally require permanently stopping study treatment. Consult guidelines for exceptions and specific events.
- Autoimmune/inflammatory events are presumably related to the mechanisms of action of ipilimumab and potentially to a therapeutic effect. The incidence and severity of these events may be dose related, but once initiated, there is no evidence that lowered doses

can be administered without continued autoimmune activity and there has so far been no demonstrable benefit to continuing ipilimumab after an autoimmune event during the initial treatment. The significance and benefit of toxicity or continued treatment in the maintenance phase has not been determined. Typically no dose modification is used for ipilimumab.

5.9.2 Immune-Related Adverse Events (irAEs) Reactions and Immune-mediated Adverse Reactions: Definition, Monitoring, and Treatment

For the purposes of this study, an immune-related adverse reaction is defined as an adverse reaction of unknown etiology associated with drug exposure and consistent with an immune phenomenon. Efforts should be made to rule out neoplastic, infectious, metabolic, toxin or other etiologic causes prior to labeling an event an irAEs. Serologic, immunologic, and histologic (biopsy) data should be used to support the diagnosis of an immune-related toxicity. Suspected immune-related adverse reactions must be documented on an AE or SAE form. Another term for an irAE is an immune-mediate adverse reaction, as it is termed in the Ipilimumab US Prescribing Information. Both terms may be used in this protocol document.

Patients should be informed of and carefully monitored for evidence of clinically significant systemic immune-mediated adverse reactions (e.g., systemic lupus erythematosus-like diseases) or organ-specific immune-mediated adverse reaction (e.g., rash, colitis, uveitis, hepatitis or thyroid disease). If an immune-mediated adverse reaction is noted, appropriate work-up (including biopsy if possible) should be performed, and steroid therapy may be considered if clinically necessary.

It is unknown if systemic corticosteroid therapy has an attenuating effect on ipilimumab activity. However, clinical anti-tumor responses have been maintained in patients treated with corticosteroids and discontinued from ipilimumab. If utilized, corticosteroid therapy should be individualized for each patient. Prior experience suggests that colitis manifested as ≥ grade 3 diarrhea requires corticosteroid treatment.

Recommended guidelines for specific immune-mediated adverse reactions are included in Section 6.2.4 below, in Appendix C, and the package insert. These recommendations should be utilized as clinically appropriate for the treatment of individual patients.

Please contact the PI or drug monitor for any questions.

5.9.3 Permanent Discontinuation of ipilimumab for Related Adverse Events (Part B)

Permanently discontinue ipilimumab for any of the following:

- Persistent adverse reactions that requires holding more than 2 treatment doses. Any grade 3 or 4 event (see exceptions below)
- Any event that requires immunosuppressive treatment or systemic steroids

Severe or life-threatening adverse reactions, including any of the following:

- Colitis with abdominal pain, fever, ileus, or peritoneal signs; increase in stool frequency (7 or more over baseline), stool incontinence, need for IV hydration for more than 24 hours, gastrointestinal hemorrhage, and gastrointestinal perforation
- Aspartate aminotransferase (AST) or alanine aminotransferase (ALT) >5 times the upper limit of normal or total bilirubin >3 times the upper limit of normal
- Stevens-Johnson syndrome, toxic epidermal necrolysis, or rash complicated by full thickness dermal ulceration, or necrotic, bullous, or hemorrhagic manifestations
- Severe motor or sensory neuropathy, Guillain-Barré syndrome, or myasthenia gravis
- Severe immune-mediated reactions involving any organ system (*e.g.*, nephritis, pneumonitis, pancreatitis, non-infectious myocarditis)
- Immune-mediated ocular disease that is unresponsive to topical immunosuppressive therapy
- Any adverse event, laboratory abnormality or intercurrent illness which, in the judgment of the investigator, presents organ specific injury and/or a substantial clinical risk to the patient with continued dosing.

<u>The following neurological adverse event requires permanent discontinuation of ipilimumab and defines unacceptable neurotoxicity:</u>

- Any motor neurologic toxicity >/= grade 3 regardless of causality
- Any >/= grade 3 treatment related sensory neurologic toxicity

Please refer to Appendix C and the IB for specific treatment algorithms.

5.9.4 Exceptions to Permanent Discontinuation of Ipilimumab

- Potentially reversible inflammation (< grade 4), attributable to a local anti-tumor reaction and a potential therapeutic response. This includes inflammatory reactions at sites of tumor resections or in draining lymph nodes, or at sites suspicious for, but not diagnostic of metastasis.
- Hospitalization for ≤ grade 2 adverse events where the primary reason for hospitalization is to expedite the clinical work-up.
- Patients with the following conditions where in the investigator's opinion continuing study drug administration is justified based on the potential for continued clinical benefit:
  - Patients treated with systemic steroids for less than 2 weeks without evidence of autoimmune disease requiring steroids treatment
  - Grade 2 skin rash treated with topical steroids for less than 4 weeks
  - Grade 2 Ocular toxicity that has completely responded to topical therapy within 4 weeks
  - Endocrinopathies where clinical symptoms are controlled with appropriate hormone replacement therapy. **Note:** Ipilimumab may not be restarted while the patient is being treated with systemic corticosteroids except for patients on stable doses of hormone replacement therapy such as hydrocortisone.

The following guidance is provided for the management of ipilimumab treatment related events. These recommendations, treatment algorithms in Appendix D, and further information in the IB, should be considered in the context of appropriate medical treatment for each patient.

5.9.5 Treatment of Infusion Reactions Associated with Ipilimumab

Since ipilimumab contains only human protein sequences, it is less likely that any allergic reaction will be seen in patients. However, it is possible that infusion of ipilimumab will induce a cytokine release syndrome that could be evidenced by fever, chills, rigors, rash, pruritus, hypotension, hypertension, bronchospasm, or other symptoms. No prophylactic pre-medication should be given unless indicated by previous experience in an individual patient. Reactions should be treated based upon the following recommendations.

For mild symptoms (*e.g.*, localized cutaneous reactions such as mild pruritus, flushing, rash):

- o Decrease the rate of infusion until recovery from symptoms, remain at bedside and monitor patient.
- o Complete the ipilimumab infusion at the initial planned rate.
- o Diphenhydramine 50 mg IV may be administered at the discretion of the treating physician and patients may receive additional doses with close monitoring.
- o Premedication with diphenhydramine may be given at the discretion of the investigator for subsequent doses of ipilimumab.
    - For moderate symptoms (any symptom not listed above [mild symptoms] or below [severe symptoms] such as generalized pruritus, flushing, rash, dyspnea, hypotension with systolic BP >80 mmHg):
- o Interrupt ipilimumab.
- o Administer diphenhydramine 50 mg IV.
- o Monitor patient closely until resolution of symptoms.
- o Corticosteroids may abrogate any beneficial immunologic effect, but may be administered at the discretion of the treating physician.
- o Resume ipilimumab infusion after recovery of symptoms.
- o At the discretion of the treating physician, ipilimumab infusion may be resumed at one half the initial infusion rate, then increased incrementally to the initial infusion rate.
- o If symptoms develop after resumption of the infusion, the infusion should be discontinued and no additional ipilimumab should be administered that day.
- o The next dose of ipilimumab will be administered at its next scheduled time and may be given with pre-medication (diphenhydramine and acetaminophen) and careful monitoring, following the same treatment guidelines outlined above.
- o At the discretion of the treating physician additional oral or IV antihistamine may be administered prior to dosing with ipilimumab.
    - For severe symptoms (e.g., any reaction such as bronchospasm, generalized urticaria, systolic blood pressure <80 mm Hg, or angioedema):
- o Immediately discontinue infusion of ipilimumab, and disconnect infusion tubing from the subject.
- o Consider bronchodilators, epinephrine 1 mg IV or subcutaneously, and/or diphenhydramine 50 mg IV, with solumedrol 100 mg IV, as needed.

- o Patients should be monitored until the investigator is comfortable that the symptoms will not recur.
- o No further ipilimumab will be administered.
  - In case of late-occurring hypersensitivity symptoms (e.g., appearance within one week after treatment of a localized or generalized pruritus), symptomatic treatment may be given (e.g., oral antihistamine, or corticosteroids).

5.9.6 Treatment of Ipilimumab-Related Isolated Drug Fever

In the event of isolated drug fever, the investigator must use clinical judgment to determine if the fever is related to the ipilimumab or to an infectious etiology. If a patient experiences isolated drug fever, for the next dose, pre-treatment with acetaminophen or non-steroidal anti-inflammatory agent (investigator discretion) should be instituted and a repeated antipyretic dose at 6 and 12 hours after ipilimumab infusion, should be administered. The infusion rate will remain unchanged for future doses. If a patient experiences recurrent isolated drug fever following premedication and post dosing with an appropriate antipyretic, the infusion rate for subsequent dosing should be decreased to 50% of the previous rate. If fever recurs following infusion rate change, the investigator should assess the patient's level of discomfort with the event and use clinical judgment to determine if the patient should receive further ipilimumab.

5.9.7 Monitoring and Management of Immune-mediated Adverse Reactions

Immune-mediated enterocolitis

- The clinical presentation of GI immune-related AEs included diarrhea, increase in the frequency of bowel movements, abdominal pain, or hematochezia, with or without fever. However inflammation may occur in any part of the GI tract including esophagitis and gastritis. Fatalities due to GI perforation have been reported in clinical trials of ipilimumab. Patients should be carefully monitored for GI symptoms that may be indicative of immune-related colitis, diarrhea, or GI perforation. Diarrhea or colitis occurring after initiation of ipilimumab therapy should be evaluated to exclude infectious or alternate etiologies. In clinical trials, immune-related colitis was associated with evidence of mucosal inflammation, with or without ulcerations, and lymphocytic infiltration.

- Monitor patients for signs and symptoms of enterocolitis (such as diarrhea, abdominal pain, mucus or blood in stool, with or without fever) and bowel perforation (such as peritoneal signs and ileus). In symptomatic patients, rule out infectious etiologies and consider endoscopic evaluation to establish etiology and for persistent or severe symptoms. *C.difficile* toxin has been detected in several patients with colitis and may be an independent entity or may co-exist with ipilimumab induced inflammatory colitis.

- Withhold ipilimumab dosing for any patients with enterocolitis pending evaluation; administer anti-diarrheal treatment and, if persistent evaluate with colonoscopy and initiate systemic corticosteroids at a dose of 0.5 mg/kg/day prednisone or equivalent.

- Permanently discontinue ipilimumab in patients with severe enterocolitis and initiate systemic corticosteroids at a dose of 1 to 2 mg/kg/day of prednisone or equivalent. Upon improvement to grade 1 or less, initiate corticosteroid taper and continue to taper over at least one month. In clinical trials, rapid corticosteroid tapering has resulted in recurrence or worsening symptoms of enterocolitis in some patients.

- Patients have been treated with anti-TNF agents for persistent colitis not responding to steroids.

- Please note autoimmune pancreatitis may cause abdominal pain and should be included in all evaluations. Enteritis may occur occasionally with other autoimmune events including hepatitis, pancreatitis, and endocrine insufficiency, which should be evaluated as clinically indicated.

Immune-mediated Hepatitis and Pancreatitis

- Hepatic immune-related AEs were mostly clinically silent and manifested as transaminase or bilirubin laboratory abnormalities. Fatal hepatic failure has been reported in clinical trials of ipilimumab. **Serum transaminase and bilirubin and lipase levels must be evaluated before each dose of ipilimumab as early laboratory changes may be indicative of emerging immune-related hepatitis/ pancreatitis and elevations in liver function tests (LFTs) may develop in the absence of clinical symptoms.** Increase in LFT or total bilirubin should be evaluated to exclude other causes of hepatic injury, including infections, disease progression, or other medications, and monitored until resolution. Liver biopsies from patients who had immune-related hepatotoxicity showed evidence of acute inflammation (neutrophils, lymphocytes, and macrophages).

- Monitor liver function tests (hepatic transaminase and bilirubin levels, lipase) and assess patients for signs and symptoms of hepatotoxicity/ pancreatitis before each dose of ipilimumab. In patients with hepatotoxicity, rule out infectious or malignant causes and increase frequency of liver function test monitoring until resolution. Withhold ipilimumab in patients with grade 2 hepatotoxicity.

- Permanently discontinue ipilimumab in patients with grade 3–5 hepatotoxicity/pancreatitis and administer systemic corticosteroids at a dose of 1 to 2 mg/kg/day of prednisone or equivalent. When liver function tests show sustained improvement or return to baseline, initiate corticosteroid tapering and continue to taper over 1 month. Across the clinical development program for ipilimumab, mycophenolate treatment has been administered in patients who have persistent severe hepatitis despite high-dose corticosteroids.

Immune-mediated Dermatitis

- Skin immune-related AEs presented mostly frequently as a rash and/or pruritus. Some subjects reported vitiligo associated with ipilimumab administration. Fatal toxic epidermal necrolysis has been reported in clinical trials of ipilimumab.

- Monitor patients for signs and symptoms of dermatitis such as rash and pruritus. Unless an alternate etiology has been identified, signs or symptoms of dermatitis should be considered immune-mediated.

- Permanently discontinue ipilimumab in patients with Stevens-Johnson syndrome, toxic epidermal necrolysis, or rash complicated by full thickness dermal ulceration, or necrotic, bullous, or hemorrhagic manifestations. Administer systemic corticosteroids at a dose of 1 to 2 mg/kg/day of prednisone or equivalent. When dermatitis is controlled, corticosteroid tapering should occur over a period of at least 1 month. Withhold ipilimumab dosing in patients with moderate to severe signs and symptoms.

- For mild to moderate dermatitis, such as grade 2 localized rash and pruritus, treat symptomatically. For persistent grade 2, grade 3, or greater, topical steroids may be administered. Administer topical or systemic corticosteroids as indicated if there is no improvement of symptoms within 1 week.

Immune-related Neurological Events

- Fatal Guillain-Barré syndrome has been reported in clinical trials of ipilimumab. Patients may present with muscle weakness and myasthenia gravis, cranial nerve palsy (n VII Bell's palsy), and aseptic meningitis, encephalopathy. Unexplained motor neuropathy, muscle weakness, or sensory neuropathy lasting more than 4 days should be evaluated and non-inflammatory causes such as disease progression, infections, metabolic syndromes, nerve entrapment, and medications should be excluded as causes.

- Withhold ipilimumab dosing in patients with any evidence of neuropathy pending evaluation.

- Monitor for symptoms of motor or sensory neuropathy such as unilateral or bilateral weakness, sensory alterations, or paresthesia. Permanently discontinue ipilimumab in patients with severe neuropathy (interfering with daily activities) such as Guillain-Barré-like syndromes. Institute medical intervention as appropriate for management of neuropathy and other neurologic events. Consider initiation of systemic corticosteroids at a dose of 1 to 2 mg/kg/day prednisone or equivalent for severe neuropathies.

Immune-mediated Endocrinopathies

- Ipilimumab can cause inflammation of endocrine organs including thyroid (Hashimoto's thryroiditis with positive antibodies) and adrenal glands, hypophysitis, hypopituitarism, and resulting thyroid and adrenal insufficiency, low ADH, prolactin, FSH, LH. Hyperthyroid with Graves' disease and positive antibody has been reported. Patients may present with subtle and nonspecific symptoms. The most common clinical presentation includes headache and fatigue. Symptoms may also include visual field defects, behavioral changes, and electrolyte disturbances including hyponatremia and hypotension. Adrenal crisis as a cause of the patient's symptoms should be excluded. Based on the available data with known outcome, most of the subjects symptomatically improved with hormone replacement therapy. Long-term hormone replacement therapy

with HC and synthroid will typically be required for subjects developing hypophysitis/hypopituitarism after treatment with ipilimumab. Some patients have regained partial function following steroid treatment.

- Monitor patients for clinical signs and symptoms of hypophysitis, adrenal insufficiency (including adrenal crisis), and hyper- or hypothyroidism. Headache is often the first symptoms of hypophysitis. Patients may present with fatigue, headache, mental status changes, loss of libido, abdominal pain, unusual bowel habits, and hypotension, or nonspecific symptoms which may resemble other causes such as brain metastasis or underlying disease. Unless an alternate etiology has been identified, signs or symptoms of endocrinopathies should be considered immune-mediated and drug withheld pending evaluation. Patients may demonstrate both central (hypophysitis) and peripheral adrenal and thyroid insufficiency. Evaluation of hypophysitis should include pituitary MRI.

- ***Endocrine evaluation, including TSH, should be performed at baseline prior to initial treatment.** Monitor thyroid function tests and clinical chemistries at the start of treatment and hold blood for possible evaluation should clinical events require determining baseline function and anti-thyroid antibodies. A plan for evaluating endocrine function at each visit either by history or monitoring TSH should be included in the protocol with further evaluation as clinically indicated. Endocrine evaluation, including TSH, should be performed at baseline prior to treatment, and the protocol must include a plan for continued monitoring of endocrine and pituitary function. The package insert for ipilimumab includes a recommendation for monitoring TSH prior to each infusion; as an early indication for pituitary dysfunction and hypophysitis, clinical monitoring of symptoms may be equally or more sensitive as an initial presentation. Clinical monitoring is required for all protocols as above, and should include any requirements per protocol for laboratory evaluation, both periodically or as clinically indicated, consistent with good medical practice. In a limited number of patients, hypophysitis was diagnosed by imaging studies through enlargement of the pituitary gland.

- Withhold ipilimumab dosing in patients symptomatic for hypophysitis. Initiate systemic corticosteroids at a dose of 1 to 2 mg/kg/day of prednisone or equivalent, and initiate appropriate hormone replacement therapy.

<u>Other Immune-mediated Adverse Reactions, Including Ocular Manifestations</u>

- Ocular inflammation, manifested as grade 2 or grade 3 episcleritis or uveitis, was associated with concomitant diarrhea in a few subjects (<1%) and occasionally occurred in the absence of clinically apparent GI symptoms. Other presumed immune-related AEs reported include, but were not limited to, arthritis/arthralgias, pneumonitis, pancreatitis, autoimmune (aseptic) meningitis, autoimmune nephritis, pure red cell aplasia, noninfective myocarditis, polymyositis, and myasthenia gravis, of which were individually reported for <1% of subjects.

- The following clinically significant immune-mediated adverse reactions were seen in less than 1% of ipilimumab-treated patients in Study 1: nephritis, pneumonitis, pulmonary

granuloma resembling sarcoidosis, meningitis, pericarditis, uveitis, iritis, ITP, neutropenia and hemolytic anemia.

- Across the clinical development program for ipilimumab, the following likely immune-mediated adverse reactions were also reported with less than 1% incidence: myocarditis, angiopathy, temporal arteritis, vasculitis, polymyalgia rheumatica, conjunctivitis, blepharitis, episcleritis, scleritis, leukocytoclastic vasculitis, erythema multiforme, psoriasis, pancreatitis, arthritis, and autoimmune thyroiditis.

- Permanently discontinue ipilimumab for clinically significant or severe immune-mediated adverse reactions. Initiate systemic corticosteroids at a dose of 1 to 2 mg/kg/day prednisone or equivalent for severe immune-mediated adverse reactions.

- Administer corticosteroid eye drops to patients who develop uveitis, iritis, or episcleritis. Permanently discontinue ipilimumab for immune-mediated ocular disease that is unresponsive to local immunosuppressive therapy.

- Overall, immune-related AEs commonly started within 3 to 10 weeks from first dose, were successfully managed in most instances by omitting doses, discontinuing dosing, and/or through administering symptomatic or immunosuppressive therapy, including corticosteroids, as mentioned above and detailed in Section 7. Immune-related AEs generally resolved within days to weeks in the majority of subjects.

5.9.8 Precautions

Combination therapy may result in unexpected toxicity especially in novel combinations with other immune modifying agents. A striking example in macaques is presented in Vaccari*, et al.* 2012.

Please note that while unproven, there is a suggestion that autoimmune events, including hepatitis, may occur more frequently at sites of metastases or prior injury.

Caution is advised when considering treatment with high-dose IL-2 in patients who have previously been administered ipilimumab, particularly in patients who experienced ipilimumab-related diarrhea/colitis.

Colonoscopy or sigmoidoscopy with biopsy may be advisable prior to IL-2 administration once the patient is no longer receiving ipilimumab. The management guidelines for general inflammatory AEs and ipilimumab-related GI toxicities, hepatotoxicity, endocrinopathy, and neuropathy (Investigator Brochure, 2011) are provided in Appendix C.

Patients who have received ipilimumab may potentially develop autoimmune disease with subsequent therapy including the appearance of colitis, hypophysitis or adrenal insufficiency.

5.10 Prohibited and Restricted Therapies

- Patients in this study may use standard vaccines. Where possible, routine vaccination for influenza, pneumococcal pneumonia should be given prior to the start of therapy but may be administered during treatment when clinically indicated. Vaccination should be given when there is enough separation to distinguish any vaccine reactions from drug toxicity. There is no experience using live attenuated vaccination during ipilimumab therapy, so that live vaccine should be used cautiously during treatment.

5.11 Treatment Beyond Progression

A minority of subjects treated with immunotherapy may derive clinical benefit either delayed responses, stable disease, or increased overall survival despite initial evidence of progressive disease (PD) with nivolumab +/- ipilimumab.

Patients may be permitted to continue treatment beyond initial RECIST 1.1-defined PD occurring during the initial treatment period (up to 12 weeks) as long as they meet the following criteria:

- No more than 4 new lesions, total sum of the longest diameter (SHORT diameter for LN) cannot exceed 40% of the initial sum including new lesions
- Patients must be clinically stable with no change in performance status due to disease progression
- No indication for immediate alternative treatment
- Patient [assessed by the investigator] is showing clinical benefit and tolerates study drug. The assessment of clinical benefit should take into account whether the subject is clinically stable or deteriorating and likely or unlikely to receive further benefit from continued treatment.
- The time of progression is noted from the first assessment that exceeds standard criteria

New lesions are considered measurable at the time of initial progression if the longest diameter is at least 10 mm (except for pathological lymph nodes, which must have a short axis of at least 15 mm). Any new lesion considered non-measurable at the time of initial progression may become measurable and therefore included in the tumor burden measurement if the longest diameter increases to at least 10 mm (except for pathological lymph nodes, which must have an increase in short axis to at least 15 mm).

Patients are allowed to continue treatment for 1 additional dose (4-8 weeks) and reassessed. Treatment may continue up to an additional 10% or 30% total single diameter increased over baseline. New measureable lesions are not permitted with this schema.

5.12 Discontinuation of Study Therapy

Subjects MUST be discontinued from study therapy AND withdrawn from the study for the following reasons:

- The development of progression PD by RECIST with modifications to allow continued treatment defined in Section 5.11. Treatment may continue under the guidelines

discussed in Section 5.7. While patients maintain minimal progression and performance status unless further benefit from ipilimumab, dosing is unlikely or requires a change of therapy.

- Withdrawal of informed consent (subject's decision to withdraw for any reason).
- Any clinical adverse event, laboratory abnormality or intercurrent illness which, in the opinion of the investigator, indicates that continued treatment with study therapy is not in the best interest of the subject.
- Termination of the study by the sponsor.
- Inability to continue treatment.
- Pregnancy
    - All WOCBP should be instructed to contact the investigator immediately if they suspect they might be pregnant (*e.g.*, missed or late menstrual period) at any time during study participation. Institutional policy and local regulations should determine the frequency of on study pregnancy tests for WOCBP enrolled in the study.
    - The investigator must immediately notify CTEP in the event of a confirmed pregnancy in a patient participating in the study.

6. DOSING DELAYS/DOSE MODIFICATIONS

6.1 Dosing Modifications

There will be no dose modifications allowed for management of toxicities.

6.2 Dosing Delays

**ALL OTHER EVENTS*^**	**Management/Next Dose for Nivolumab +/- Ipilimumab**
≤ Grade 1	No change in dose
Grade 2	Hold until ≤ Grade 1 OR baseline Resume at same dose level.
Grade 3	Hold* until ≤ Grade 1 continue at investigator discretion
Grade 4	Off protocol therapy
* **Not agent related**, or agent related non-immunologically mediated<br>^Per investigator discretion, unrelated Grade 2 anemia does not require dose delay.	
Recommended management: As clinically indicated	
**ALL OTHER EVENTS****	**Management/Next Dose for Nivolumab +/- Ipilimumab**
≤ Grade 1	No change in dose
Grade 2	Hold until ≤ Grade 1 OR baseline* When resolved < or following steroids resume at same dose level .
Grade 3	Off protocol therapy (exceptions noted in 5.4)
Grade 4	Off protocol therapy
* *immunologically mediated, with the exception of the immune-related events mentioned on pages 64-65	
Recommended management: As clinically indicated	

**Skin Rash and Oral Lesions**	**Management/Next Dose for Nivolumab +/- Ipilimumab**
Grade 1-2	Refer to Skin Adverse Event management algorithm (Appendix D).
Grade 3	Delay therapy. Refer to Skin Adverse Event management algorithm (Appendix D).
Grade 4	Off protocol therapy
*Patients with purpuric or bullous lesions must be evaluated for vasculitis, Steven-Johnson syndrome, TEN, and autoimmune bullous disease including oral lesions of bullous pemphigus/ pemphigoid. Pruritus may occur with or without skin rash and should be treated symptomatically if there is no associated liver or GI toxicity. Note skin rash typically occurs early and may be followed by additional events particularly during steroids tapering. Rule out non-inflammatory causes. If non-inflammatory cause, then treat accordingly and continue nivolumab therapy.	

**Renal Function**	**Management/Next Dose for Nivolumab +/- Ipilimumab**
Grade 1-2	Refer to Renal Adverse Event management algorithm (Appendix D).
Grade 3	Delay therapy. Refer to Renal Adverse Event management algorithm (Appendix D).
Grade 4	Off protocol therapy
**Liver Function (AST, ALT, total bilirubin)**	**Management/Next Dose for Nivolumab +/- Ipilimumab**
Grade 1	Refer to Hepatic Adverse Event management algorithm (Appendix D).
Grade 2	Delay therapy. Refer to Hepatic Adverse Event management algorithm (Appendix D).
Grade 3	Delay therapy. Refer to Hepatic Adverse Event management algorithm (Appendix D).
Grade 4	Off protocol therapy.
Continued treatment of active immune mediated hepatitis may exacerbate ongoing inflammation. Holding drug to evaluate LFT changes and to rule out non-inflammatory causes. Early treatment is recommended. LFT changes may occur during steroid tapers from other events and may occur together with other GI events including cholecystitis/pancreatitis.	

**Diarrhea/ Colitis**	**Management/Next Dose for Nivolumab +/- Ipilimumab**
Grade 1	Refer to GI Adverse Event management algorithm (Appendix D).
Grade 2	Delay therapy. Refer to GI Adverse Event management algorithm (Appendix D).
Grade 3	Delay therapy. Refer to GI Adverse Event management algorithm (Appendix D).
Grade 4	Off protocol therapy.
See GI AE Algorithm for management of symptomatic colitis. Patients with grade 2 symptoms but normal colonoscopy and biopsies may be retreated after resolution. Evaluation for all patients for additional causes includes *C. diff*, acute and self-limited infectious and	

**Diarrhea/ Colitis**	**Management/Next Dose for Nivolumab +/- Ipilimumab**
foodborne illness, ischemic bowel, diverticulitis, and IBD.	
Recommended management: see GI AE management Algorithm	

**Pancreatitis Amylase/Lipase**	**Management/Next Dose for Nivolumab +/- Ipilimumab**
Grade 1	Hold dose until grade 0
Grade 2	Hold dose until Grade 0. Resume at same dose level if asymptomatic.
Grade 3-4	Hold* dose until Grade 0. Resume at same dose level if asymptomatic. Patients who develop symptomatic pancreatitis or DM should be taken off treatment. *
*Patients may develop symptomatic and radiologic evidence of pancreatitis as well as DM and DKA. Lipase elevation may occur during the period of steroid withdrawal and with other immune mediated events or associated with colitis, hepatitis, and patients who have asymptomatic lipase elevation typically have self-limited course and may be retreated.<br>For treatment management of symptomatic pancreatitis please follow the Hepatic Adverse Event Management Algorithm	

**Pneumonitis**	**Management/Next Dose for Nivolumab +/- Ipilimumab**
Grade 1	Hold dose pending evaluation and resolution to Grade 0 or baseline including baseline pO2.*
Grade 2	Hold dose pending evaluation. Refer to Pulmonary Adverse Event management algorithm (appendix D).
Grade 3	Hold dose pending evaluation. Refer to Pulmonary Adverse Event management algorithm (appendix D).
Grade 4	Off protocol therapy.
Distinguishing inflammatory pneumonitis is often a diagnosis of exclusion for patients who do not respond to antibiotics and have no causal organism identified including influenza. Most patients with respiratory failure or hypoxia will be treated with steroids. Bronchoscopy may be required and analysis of lavage fluid for lymphocytic predominance may be helpful. Patients with new lung nodules should be evaluated for sarcoid like granuloma. Please consider recommending seasonal influenza killed vaccine for all patients.<br>*Note: this is more conservative management than the Pulmonary Adverse Event Management algorithm (appendix D) suggests for Grade 1 pneumonitis	

**Other GI Nausea/Vomiting**	**Management/Next Dose for Nivolumab +/- Ipilimumab**
≤ Grade 1	No change in dose.
Grade 2	Hold pending evaluation for gastritis duodenitis and other immune adverse events or other causes. Resume at same dose level after resolution to ≤ Grade 1.
Grade 3	Hold pending evaluation until ≤ Grade 1. Resume at same dose level. If symptoms do not resolve within 7 days with symptomatic treatment patients should go off protocol therapy
Grade 4	Off protocol therapy
Patients with grade 2 or 3 N-V should be evaluated for upper GI inflammation and other immune related	

**Other GI Nausea/Vomiting**	**Management/Next Dose for Nivolumab +/- Ipilimumab**
events.	

**Fatigue**	**Management/Next Dose for Nivolumab +/- Ipilimumab**
≤ Grade 1	No change in dose.
Grade 2	No change in dose
Grade 3	Hold until ≤ Grade 2. Resume at same dose level
Grade 4	Off protocol therapy
Fatigue is the most common adverse event associated with immune checkpoint therapy. Grade 2 or greater fatigue should be evaluated for associated or underlying organ involvement including pituitary, thyroid, and hepatic, or muscle (CPK) inflammation	

**Neurologic events**	**Management/Next Dose for Nivolumab +/- Ipilimumab**
Grade 1	Hold dose pending evaluation and observation. Resume with no change in dose. * Refer to Neurological Adverse Event management algorithm (Appendix D).
Grade 2	Hold dose pending evaluation and observation.* Refer to Neurological Adverse Event management algorithm (Appendix D).
Grade 3-4	Off protocol therapy
*Patients with any CNS events including aseptic meningitis, encephalitis, symptomatic hypophysitis, or myopathy, peripheral demyelinating neuropathy, cranial neuropathy (other than peripheral n. VII), GB syndrome, or myasthenia gravis should be off study.	
Recommended management: See Neurologic Adverse Event Management Algorithm	

**Endocrine Hypophysitis or Adrenal Insufficiency**	**Management/Next Dose for Nivolumab +/- Ipilimumab**
Grade 1-2	Asymptomatic TSH elevation * Hold pending evaluation, endocrine consult . Refer to Endocrinopathy Adverse Event management algorithm (Appendix D).
Grade 3-4	Off protocol therapy
Note all patients with symptomatic pituitary enlargement, exclusive of hormone deficiency, but including severe headache or enlarged pituitary on MRI should be considered grade 3 events. Isolated thyroid or testosterone deficiency may be treated as grade 2 if there are no other associated deficiencies and adrenal function is monitored.<br>Please evaluate pituitary function before beginning steroid therapy or replacement therapy of any kind.<br>*Note patients with thyroiditis may be retreated on replacement therapy. Patients must be evaluated to rule out pituitary disease prior to initiating thyroid replacement.	
Recommended management: See Endocrine Management Algorithm	

Fever	Management/Next Dose for Nivolumab +/- Ipilimumab
≤ Grade 1	Continue therapy at same dose. If no improvement after 5-7 days or worsening, then treat as Grade 2.
Grade 2	Hold until ≤ Grade 1. Resume at same dose level.
Grade 3	Hold until ≤ Grade 1. Resume at same dose level.
Grade 4	Off treatment
Patients with fever should be evaluated as clinically appropriate. Patients may experience isolated fever during infusion reactions or up to several days after infusion. Evaluation over the course of 1-2 weeks should be done for other autoimmune events that may present as fever	
See section 5. infusion reactions	

Cardiac*	Management/Next Dose for (Nivolumab) + Ipilimumab Cardiac Toxicities
≤ Grade 1	Hold dose pending evaluation and observation.** Evaluate for signs and symptoms of CHF, ischemia, arrhythmia or myositis. Obtain history EKG, CK (for concomitant myositis), CK-MB. Repeat troponin, CK and EKG 2-3 days. If troponin and labs normalize may resume therapy. If labs worsen or symptoms develop then treat as below. Hold pending evaluation
Grade ≥2 with suspected myocarditis	Hold dose.** Admit to hospital. Cardiology consult. Rule out MI and other causes of cardiac disease. Cardiac Monitoring. Cardiac Echo. Consider cardiac MRI and cardiac biopsy. Initiate high dose methylprednisolone. If no improvement within 24 hours, add either infliximab, ATG or tacrolimus. Resume therapy if there is a return to baseline and myocarditis is excluded or considered unlikely.
Grade ≥2 with confirmed myocarditis	Off protocol therapy. Admit to CCU (consider transfer to nearest Cardiac Transplant Unit). Treat as above. Consider high dose methylprednisolone.<br>Add ATG or tacrolimus if no improvement.
**Including CHF, LV systolic dysfunction, Myocarditis, CPK, and troponin*<br>***Patients with evidence of myositis without myocarditis may be treated according as "other event"*<br>Note: The optimal treatment regimen for immune mediated myocarditis has not been established. Since this toxicity has caused patient deaths, an aggressive approach is recommended.	

If treatment is delayed >6 weeks (>8 weeks for patients on high dose steroids) with recommended 4 weeks taper and 2 week observation, the patient must be permanently discontinued from study therapy, except as specified in Section 5.7 (Criteria to Resume Treatment.)

Patients requiring a delay of >6 weeks (>8 weeks for patients on high dose steroids with required

4 weeks minimal taper and 2 week observation), should go off protocol therapy.
Patients requiring > two dose delays for the same event should go off protocol therapy.

Prior to starting corticosteroids or hormone replacement for any reason, appropriate endocrine testing including cortisol, ACTH, TSH and T4 must be obtained to document baseline.

Patients may be dose-delayed for evaluation and restarted depending on results.

Any patient started on corticosteroids initially who is determined to not require steroids treatment for an autoimmune adverse event may resume therapy after a 2 week observation period without further symptoms at the discretion of the PI or investigator.

7. ADVERSE EVENTS: LIST AND REPORTING REQUIREMENTS

Adverse event (AE) monitoring and reporting is a routine part of every clinical trial. The following list of AEs (Section 7.1) and the characteristics of an observed AE (Section 7.2) will determine whether the event requires expedited reporting via the CTEP Adverse Event Reporting System (CTEP-AERS) **in addition** to routine reporting.

7.1 Comprehensive Adverse Events and Potential Risks List (CAEPR)

The Comprehensive Adverse Event and Potential Risks list (CAEPR) provides a single list of reported and/or potential adverse events (AE) associated with an agent using a uniform presentation of events by body system. In addition to the comprehensive list, a subset of AEs, the Specific Protocol Exceptions to Expedited Reporting (SPEER), appears in a separate column and is identified with ***bold*** and ***italicized*** text. The SPEER is a list of events that are protocol-specific exceptions to expedited reporting to NCI (except as noted below). Refer to the 'CTEP, NCI Guidelines: Adverse Event Reporting Requirements' http://ctep.cancer.gov/protocolDevelopment/adverse_effects.htm for further clarification.

NOTE: The highest grade currently reported is noted in parentheses next to the AE in the SPEER. Report **ONLY** AEs higher than this grade expeditiously.

7.1.1 CAEPR for CTEP IND Agent Nivolumab (Parts A+B)

Comprehensive Adverse Events and Potential Risks list (CAEPR)
for
BMS-936558 (Nivolumab, MDX-1106, NSC 748726)

The Comprehensive Adverse Events and Potential Risks list (CAEPR) provides a single list of reported and/or potential adverse events (AE) associated with an agent using a uniform presentation of events by body system. In addition to the comprehensive list, a subset, the Specific Protocol Exceptions to Expedited Reporting (SPEER), appears in a separate column and is identified with bold and italicized text. This subset of AEs (SPEER) is a list of events that are protocol specific exceptions to expedited reporting to NCI (except as noted below).

Refer to the 'CTEP, NCI Guidelines: Adverse Event Reporting Requirements' http://ctep.cancer.gov/protocolDevelopment/electronic_applications/docs/aeguidelines.pdf for further clarification. Frequency is provided based on 2678 patients. Below is the CAEPR for Ipilimumab (MDX-010).

NOTE: Report AEs on the SPEER **ONLY IF** they exceed the grade noted in parentheses next to the AE in the SPEER. If this CAEPR is part of a combination protocol using multiple investigational agents and has an AE listed on different SPEERs, use the lower of the grades to determine if expedited reporting is required.

Version 2.4, December 2, 2020[1]

**Adverse Events with Possible Relationship to Nivolumab (CTCAE 5.0 Term) [n= 2069]**			**Specific Protocol Exceptions to Expedited Reporting (SPEER)**
**Likely (>20%)**	**Less Likely (<=20%)**	**Rare but Serious (<3%)**	
BLOOD AND LYMPHATIC SYSTEM DISORDERS			
	Anemia		***Anemia (Gr 3)***
CARDIAC DISORDERS			
		Cardiac disorders - Other (cardiomyopathy)	
		Myocarditis	
		Pericardial tamponade[2]	
		Pericarditis	
ENDOCRINE DISORDERS			
	Adrenal insufficiency[3]		
	Hyperthyroidism[3]		
	Hypophysitis[3]		
	Hypothyroidism[3]		
EYE DISORDERS			
		Blurred vision	
		Dry eye	
		Eye disorders - Other (diplopia)[3]	
		Eye disorders - Other (Graves ophthalmopathy)[3]	
		Eye disorders - Other (optic neuritis retrobulbar)[3]	
		Eye disorders - Other (Vogt-Koyanagi-Harada)	
	Uveitis		
GASTROINTESTINAL DISORDERS			
	Abdominal pain		***Abdominal pain (Gr 2)***
	Colitis[3]		
		Colonic perforation[3]	
	Diarrhea		***Diarrhea (Gr 3)***
	Dry mouth		***Dry mouth (Gr 2)***
		Enterocolitis	
		Gastritis	
		Mucositis oral	
	Nausea		***Nausea (Gr 2)***
	Pancreatitis[4]		
GENERAL DISORDERS AND ADMINISTRATION SITE CONDITIONS			
Fatigue			***Fatigue (Gr 3)***
	Fever		***Fever (Gr 2)***
	Injection site reaction		***Injection site reaction (Gr 2)***
HEPATOBILIARY DISORDERS			
		Hepatobiliary disorders - Other (immune-mediated hepatitis)	
IMMUNE SYSTEM DISORDERS			

**Adverse Events with Possible Relationship to Nivolumab (CTCAE 5.0 Term) [n= 2069]**			**Specific Protocol Exceptions to Expedited Reporting (SPEER)**
**Likely (>20%)**	**Less Likely (<=20%)**	**Rare but Serious (<3%)**	
		Allergic reaction[3]	
		Autoimmune disorder[3]	
		Cytokine release syndrome[5]	
		Immune system disorders - Other (GVHD in the setting of allotransplant)[3,6]	
		Immune system disorders - Other (sarcoidosis)[3]	
INJURY, POISONING AND PROCEDURAL COMPLICATIONS			
	Infusion related reaction[7]		
INVESTIGATIONS			
	Alanine aminotransferase increased[3]		***Alanine aminotransferase increased[3] (Gr 3)***
	Aspartate aminotransferase increased[3]		***Aspartate aminotransferase increased[3] (Gr 3)***
	Blood bilirubin increased[3]		***Blood bilirubin increased[3] (Gr 2)***
	CD4 lymphocytes decreased		***CD4 lymphocyte decreased (Gr 4)***
	Creatinine increased		
	Lipase increased		
	Lymphocyte count decreased		***Lymphocyte count decreased (Gr 4)***
	Neutrophil count decreased		
	Platelet count decreased		
	Serum amylase increased		
METABOLISM AND NUTRITION DISORDERS			
	Anorexia		
		Hyperglycemia	***Hyperglycemia (Gr 2)***
		Metabolism and nutrition disorders - Other (diabetes mellitus with ketoacidosis)[3]	
MUSCULOSKELETAL AND CONNECTIVE TISSUE DISORDERS			
	Arthralgia		
		Musculoskeletal and connective tissue disorder - Other (polymyositis)	
		Myositis	
		Rhabdomyolysis	
NERVOUS SYSTEM DISORDERS			
		Encephalopathy[3]	
		Facial nerve disorder[3]	
		Guillain-Barre syndrome[3]	
		Myasthenia gravis[3]	
		Nervous system disorders - Other (demyelination myasthenic syndrome)	
		Nervous system disorders - Other (encephalitis)[3]	
		Nervous system disorders - Other (meningoencephalitis)	

Adverse Events with Possible Relationship to Nivolumab (CTCAE 5.0 Term) [n= 2069]			Specific Protocol Exceptions to Expedited Reporting (SPEER)
**Likely (>20%)**	**Less Likely (<=20%)**	**Rare but Serious (<3%)**	
		Nervous system disorders - Other (meningoradiculitis)[3]	
		Nervous system disorders - Other (myasthenic syndrome)	
		Peripheral motor neuropathy	
		Peripheral sensory neuropathy	
		Reversible posterior leukoencephalopathy syndrome[3]	
RENAL AND URINARY DISORDERS			
		Acute kidney injury[3]	
		Renal and urinary disorders - Other (immune-mediated nephritis)	
RESPIRATORY, THORACIC AND MEDIASTINAL DISORDERS			
	Pleural effusion[3]		
	Pneumonitis[3]		
		Respiratory, thoracic and mediastinal disorders - Other (bronchiolitis obliterans with organizing pneumonia)[3]	
SKIN AND SUBCUTANEOUS TISSUE DISORDERS			
		Erythema multiforme[3]	
	Pruritus[3]		***Pruritus[3] (Gr 2)***
	Rash maculo-papular[3]		***Rash maculo-papular[3] (Gr 2)***
		Skin and subcutaneous tissue disorders - Other (bullous pemphigoid)	
	Skin and subcutaneous tissue disorders - Other (Sweet's Syndrome)[3]		
	Skin hypopigmentation[3]		
		Stevens-Johnson syndrome	
		Toxic epidermal necrolysis	

[1]This table will be updated as the toxicity profile of the agent is revised. Updates will be distributed to all Principal Investigators at the time of revision. The current version can be obtained by contacting PIO@CTEP.NCI.NIH.GOV. Your name, the name of the investigator, the protocol and the agent should be included in the e-mail.

[2]Pericardial tamponade may be related to possible inflammatory reaction at tumor site.

[3] Nivolumab being a member of class of agents involved in the inhibition of "immune checkpoints", may result in severe and possibly fatal immune-mediated adverse events probably due to T-cell activation and proliferation. This may result in autoimmune disorders that can include (but are not limited to) autoimmune hemolytic anemia, acquired anti-factor VIII immune response, autoimmune aseptic meningitis, autoimmune hepatitis, autoimmune nephritis, autoimmune neuropathy, autoimmune thyroiditis, bullous pemphigoid, exacerbation of Churg-Strauss Syndrome, drug rash with eosinophilia systemic symptoms [DRESS] syndrome, facial nerve disorder (facial nerve paralysis), limbic encephalitis, hepatic failure, pure red cell aplasia, pancreatitis, ulcerative and hemorrhagic colitis, endocrine disorders (e.g., autoimmune thyroiditis, hyperthyroidism, hypothyroidism, autoimmune hypophysitis/hypopituitarism, thyrotoxicosis, and adrenal insufficiency), sarcoid granuloma, myasthenia gravis, polymyositis, and Guillain-Barre syndrome.

[4]Pancreatitis may result in increased serum amylase and/or more frequently lipase.

[5]Cytokine release syndrome may manifest as hemophagocytic lymphohistiocytosis with accompanying fever and pancytopenia.

[6]Complications including hyperacute graft-versus-host disease (GVHD), some fatal, have occurred in patients receiving allo stem cell transplant (SCT) after receiving Nivolumab. These complications may occur despite intervening therapy between receiving Nivolumab and allo-SCT.

[7]Infusion reactions, including high-grade hypersensitivity reactions which have been observed following administration of nivolumab, may manifest as fever, chills, shakes, itching, rash, hypertension or hypotension, or difficulty breathing during and immediately after administration of nivolumab.

Adverse events reported on Nivolumab trials, but for which there is insufficient evidence to suggest that there was a reasonable possibility that Nivolumab caused the adverse event:

BLOOD AND LYMPHATIC SYSTEM DISORDERS - Leukocytosis
CARDIAC DISORDERS - Atrial fibrillation; Atrioventricular block complete; Heart failure; Ventricular arrhythmia
EAR AND LABYRINTH DISORDERS - Vestibular disorder
EYE DISORDERS - Eye disorders - Other (iridocyclitis); Optic nerve disorder; Periorbital edema
GASTROINTESTINAL DISORDERS - Constipation; Duodenal ulcer; Flatulence; Gastrointestinal disorders - Other (mouth sores); Vomiting
GENERAL DISORDERS AND ADMINISTRATION SITE CONDITIONS - Chills; Edema limbs; Malaise; Pain
HEPATOBILIARY DISORDERS - Bile duct stenosis
IMMUNE SYSTEM DISORDERS - Anaphylaxis; Immune system disorders - Other (autoimmune thrombotic microangiopathy); Immune system disorders - Other (limbic encephalitis)
INFECTIONS AND INFESTATIONS - Bronchial infection; Lung infection; Sepsis; Upper respiratory infection
INVESTIGATIONS - Blood lactate dehydrogenase increased; GGT increased; Investigations - Other (protein total decreased); Lymphocyte count increased; Weight loss
METABOLISM AND NUTRITION DISORDERS - Dehydration; Hyperuricemia; Hypoalbuminemia; Hypocalcemia; Hyponatremia; Hypophosphatemia
MUSCULOSKELETAL AND CONNECTIVE TISSUE DISORDERS - Back pain; Musculoskeletal and connective tissue disorder - Other (musculoskeletal pain); Musculoskeletal and connective tissue disorder - Other (polymyalgia rheumatica); Myalgia; Pain in extremity
NEOPLASMS BENIGN, MALIGNANT AND UNSPECIFIED (INCL CYSTS AND POLYPS) - Neoplasms benign, malignant and unspecified (incl cysts and polyps) - Other (Histiocytic necrotizing lymphadenitis)
NERVOUS SYSTEM DISORDERS - Dizziness; Headache; Intracranial hemorrhage
PSYCHIATRIC DISORDERS - Insomnia
RENAL AND URINARY DISORDERS - Hematuria; Renal and urinary disorders - Other (tubulointerstitial nephritis)
RESPIRATORY, THORACIC AND MEDIASTINAL DISORDERS - Bronchospasm; Cough; Dyspnea; Hypoxia
SKIN AND SUBCUTANEOUS TISSUE DISORDERS - Alopecia; Dry skin; Hyperhidrosis; Pain of skin; Photosensitivity; Rash acneiform; Skin and subcutaneous tissue disorders - Other (rosacea)
VASCULAR DISORDERS - Flushing; Hypertension; Hypotension; Vasculitis

Note: Nivolumab in combination with other agents could cause an exacerbation of any adverse event currently known to be caused by the other agent, or the combination may result in events never previously associated with either agent.

7.1.2 Comprehensive Adverse Events and Potential Risks list (CAEPR)

for

Ipilimumab (MDX-010, NSCs 732442 and 720801)

The Comprehensive Adverse Events and Potential Risks list (CAEPR) provides a single list of reported and/or potential adverse events (AE) associated with an agent using a uniform presentation of events by body system. In addition to the comprehensive list, a subset, the Specific Protocol Exceptions to Expedited Reporting (SPEER), appears in a separate column and is identified with bold and italicized text. This subset of AEs (SPEER) is a list of events that are protocol specific exceptions to expedited reporting to NCI (except as noted below). Refer to the 'CTEP, NCI Guidelines: Adverse Event Reporting Requirements' http://ctep.cancer.gov/protocolDevelopment/electronic_applications/docs/aeguidelines.pdf for further clarification. *Frequency is provided based on 2678 patients.* Below is the CAEPR for Ipilimumab (MDX-010).

NOTE: Report AEs on the SPEER **ONLY IF** they exceed the grade noted in parentheses next to the AE in the SPEER. If this CAEPR is part of a combination protocol using multiple investigational agents and has an AE listed on different SPEERs, use the lower of the grades to determine if expedited reporting is required.

Version 2.10, March 29, 2019[1]

**Adverse Events with Possible Relationship to Ipilimumab (MDX-010) (CTCAE 5.0 Term) [n= 2678]**			**Specific Protocol Exceptions to Expedited Reporting (SPEER)**
**Likely (>20%)**	**Less Likely (<=20%)**	**Rare but Serious (<3%)**	
BLOOD AND LYMPHATIC SYSTEM DISORDERS			
		Blood and lymphatic system disorders - Other (acquired hemophilia)	
CARDIAC DISORDERS			
	Atrial fibrillation		
		Myocarditis[2]	
		Pericardial effusion	
EAR AND LABYRINTH DISORDERS			
	Hearing impaired		
ENDOCRINE DISORDERS			
	Adrenal insufficiency[2]		
	Hyperthyroidism[2]		
	Hypophysitis[2]		
	Hypopituitarism[2]		
	Hypothyroidism[2]		
	Testosterone deficiency[2]		
EYE DISORDERS			
	Eye disorders - Other (episcleritis)[2]		
	Uveitis[2]		
GASTROINTESTINAL DISORDERS			
	Abdominal pain		
	Colitis[2]		***Colitis[2] (Gr 3)***
		Colonic perforation[3]	
	Constipation		
Diarrhea			***Diarrhea (Gr 3)***
	Enterocolitis		
	Esophagitis		
		Ileus	
Nausea			***Nausea (Gr 3)***
	Pancreatitis[2]		
	Vomiting		

Adverse Events with Possible Relationship to Ipilimumab (MDX-010) (CTCAE 5.0 Term) [n= 2678]			Specific Protocol Exceptions to Expedited Reporting (SPEER)
**Likely (>20%)**	**Less Likely (<=20%)**	**Rare but Serious (<3%)**	
GENERAL DISORDERS AND ADMINISTRATION SITE CONDITIONS			
	Chills		
Fatigue			*Fatigue (Gr 3)*
	Fever		*Fever (Gr 2)*
		General disorders and administration site conditions - Other (Systemic inflammatory response syndrome [SIRS])	
		Multi-organ failure	
HEPATOBILIARY DISORDERS			
	Hepatobiliary disorders - Other (hepatitis)[2]		
IMMUNE SYSTEM DISORDERS			
	Autoimmune disorder[2]		
		Immune system disorders - Other (GVHD in the setting of allotransplant)[4]	
INFECTIONS AND INFESTATIONS			
		Infections and infestations - Other (aseptic meningitis)[2]	
INJURY, POISONING AND PROCEDURAL COMPLICATIONS			
	Infusion related reaction		
INVESTIGATIONS			
	Alanine aminotransferase increased		
	Aspartate aminotransferase increased		
		Lymphocyte count decreased	
	Neutrophil count decreased		
	Weight loss		
METABOLISM AND NUTRITION DISORDERS			
	Anorexia		
	Dehydration		
	Hyperglycemia		
		Metabolism and nutrition disorders - Other (exacerbation of pre-existing diabetes mellitus)	
MUSCULOSKELETAL AND CONNECTIVE TISSUE DISORDERS			
	Arthralgia		
	Arthritis		
		Generalized muscle weakness	
	Musculoskeletal and connective tissue disorder - Other (polymyositis)[2]		
NERVOUS SYSTEM DISORDERS			
		Ataxia	
	Facial nerve disorder[2]		
	Guillain-Barre syndrome[2]		
	Headache		
	Myasthenia gravis[2]		

Adverse Events with Possible Relationship to Ipilimumab (MDX-010) (CTCAE 5.0 Term) [n= 2678]			Specific Protocol Exceptions to Expedited Reporting (SPEER)
**Likely (>20%)**	**Less Likely (<=20%)**	**Rare but Serious (<3%)**	
		Nervous system disorders - Other (immune-mediated encephalitis)[2]	
		Peripheral motor neuropathy	
		Peripheral sensory neuropathy	
	Trigeminal nerve disorder		
PSYCHIATRIC DISORDERS			
		Psychiatric disorders - Other (mental status changes)	
RENAL AND URINARY DISORDERS			
	Acute kidney injury		
	Renal and urinary disorders - Other (granulomatous tubulointerstitial nephritis)		
RESPIRATORY, THORACIC AND MEDIASTINAL DISORDERS			
	Pneumonitis		
		Respiratory failure	
		Respiratory, thoracic and mediastinal disorders - Other (bronchiolitis obliterans with organizing pneumonia)	
		Respiratory, thoracic and mediastinal disorders - Other (lung infiltration)	
SKIN AND SUBCUTANEOUS TISSUE DISORDERS			
		Erythema multiforme	
	Pruritus		***Pruritus (Gr 3)***
Rash maculo-papular			***Rash maculo-papular (Gr 3)***
	Skin and subcutaneous tissue disorders - Other (Sweet's Syndrome)		
		Stevens-Johnson syndrome	
		Toxic epidermal necrolysis	
	Urticaria		
VASCULAR DISORDERS			
	Hypotension		

[1]This table will be updated as the toxicity profile of the agent is revised. Updates will be distributed to all Principal Investigators at the time of revision. The current version can be obtained by contacting PIO@CTEP.NCI.NIH.GOV. Your name, the name of the investigator, the protocol and the agent should be included in the e-mail.

[2]Ipilimumab can result in severe and fatal immune-mediated adverse events probably due to T-cell activation and proliferation. These can include (but are not limited to) autoimmune hemolytic anemia, acquired anti-factor VIII immune response, autoimmune aseptic meningitis, autoimmune hepatitis, autoimmune thyroiditis, hepatic failure, pure red cell aplasia, pancreatitis, ulcerative and hemorrhagic colitis, endocrine disorders (e.g., autoimmune thyroiditis, hyperthyroidism, hypothyroidism, autoimmune hypophysitis/hypopituitarism, and adrenal insufficiency), ocular manifestations (e.g., uveitis, iritis, conjunctivitis, blepharitis, and episcleritis), sarcoid granuloma, myasthenia gravis, polymyositis, and Guillain-Barre syndrome. The majority of these reactions manifested early during treatment; however, a minority occurred weeks to months after discontinuation of ipilimumab especially with the initiation of additional treatments.

[3]Late bowel perforations have been noted in patients receiving MDX-010 (ipilimumab) in association with subsequent IL-2 therapy.

[4]Complications including hyperacute graft-versus-host disease (GVHD), may occur in patients receving allo stem cell transplant (SCT) after receiving Ipilimumab (MDX-010). These complications may occur despite intervening therapy between receiving Ipilimumab (MDX-010) and allo-SCT.

[5]In rare cases diplopia (double vision) has occurred as a result of muscle weakness (Myasthenia gravis).

[6]Gastrointestinal hemorrhage includes Anal hemorrhage, Cecal hemorrhage, Colonic hemorrhage, Duodenal hemorrhage, Esophageal hemorrhage, Esophageal varices hemorrhage, Gastric hemorrhage, Hemorrhoidal hemorrhage, Ileal hemorrhage, Intra-abdominal hemorrhage, Jejunal hemorrhage, Lower gastrointestinal hemorrhage, Oral hemorrhage, Pancreatic hemorrhage, Rectal hemorrhage, Retroperitoneal hemorrhage, and Upper gastrointestinal hemorrhage under the GASTROINTESTINAL DISORDERS SOC.

[7]Infection includes all 75 sites of infection under the INFECTIONS AND INFESTATIONS SOC.

Adverse events reported on Ipilimumab (MDX-010) trials, but for which there is insufficient evidence to suggest that there was a reasonable possibility that Ipilimumab (MDX-010) caused the adverse event:

BLOOD AND LYMPHATIC SYSTEM DISORDERS - Anemia; Blood and lymphatic system disorders - Other (pure red cell aplasia)[2]; Febrile neutropenia
CARDIAC DISORDERS - Conduction disorder; Restrictive cardiomyopathy
EYE DISORDERS - Extraocular muscle paresis[5]; Eye disorders - Other (retinal pigment changes)
GASTROINTESTINAL DISORDERS - Colonic ulcer; Dyspepsia; Dysphagia; Gastrointestinal disorders - Other (gastroenteritis); Gastrointestinal hemorrhage[6]; Proctitis
GENERAL DISORDERS AND ADMINISTRATION SITE CONDITIONS - Flu like symptoms; Non-cardiac chest pain
HEPATOBILIARY DISORDERS - Hepatic failure[2]
IMMUNE SYSTEM DISORDERS - Allergic reaction
INFECTIONS AND INFESTATIONS - Infection[7]
INVESTIGATIONS - Creatinine increased; Investigations - Other (rheumatoid factor); Lipase increased; Platelet count decreased; Serum amylase increased; White blood cell decreased
METABOLISM AND NUTRITION DISORDERS - Tumor lysis syndrome
MUSCULOSKELETAL AND CONNECTIVE TISSUE DISORDERS - Back pain; Joint range of motion decreased; Myalgia; Pain in extremity
NEOPLASMS BENIGN, MALIGNANT AND UNSPECIFIED (INCL CYSTS AND POLYPS) - Tumor pain
NERVOUS SYSTEM DISORDERS - Dizziness; Dysphasia; Ischemia cerebrovascular; Seizure
PSYCHIATRIC DISORDERS - Anxiety; Confusion; Depression; Insomnia
RENAL AND URINARY DISORDERS - Proteinuria
RESPIRATORY, THORACIC AND MEDIASTINAL DISORDERS - Allergic rhinitis; Cough; Dyspnea; Laryngospasm
SKIN AND SUBCUTANEOUS TISSUE DISORDERS - Alopecia; Dry skin; Hyperhidrosis; Skin hypopigmentation
VASCULAR DISORDERS - Flushing; Hypertension; Vascular disorders - Other (temporal arteritis)

Note: Ipilimumab (BMS-734016; MDX-010 Transfectoma-derived) in combination with other agents could cause an exacerbation of any adverse event currently known to be caused by the other agent, or the combination may result in events never previously associated with either agent.

7.2 Adverse Event Characteristics

- **CTCAE term (AE description) and grade:** The descriptions and grading scales found in the revised NCI Common Terminology Criteria for Adverse Events (CTCAE) version 4.0 will be utilized for AE reporting. All appropriate treatment areas should have access to a copy of the CTCAE version 4.0. A copy of the CTCAE version 4.0 can be downloaded from the CTEP web site http://ctep.cancer.gov/protocolDevelopment/electronic_applications/ctc.htm.

- **For expedited reporting purposes only:**
  - AEs for the agent that are ***bold and italicized*** in the CAEPR (*i.e.*, those listed in the SPEER column, Section 7.1.1) should be reported through CTEP-AERS, however CTCAE version 5.0 must be used for serious AE reporting through CTEP-AERS, only if the grade is above the grade provided in the SPEER.
  - Other AEs for the protocol that do not require expedited reporting are outlined in section 7.3.4.

- **Attribution** of the AE:
  - Definite – The AE *is clearly related* to the study treatment.
  - Probable – The AE *is likely related* to the study treatment.
  - Possible – The AE *may be related* to the study treatment.
  - Unlikely – The AE *is doubtfully related* to the study treatment.
  - Unrelated – The AE *is clearly NOT related* to the study treatment.

7.3 Expedited Adverse Event Reporting

7.3.1 Expedited AE reporting for this study must use CTEP-AERS (CTEP Adverse Event Reporting System), accessed via the CTEP Web site (https://eapps-ctep.nci.nih.gov/ctepaers). The reporting procedures to be followed are presented in the "NCI Guidelines for Investigators: Adverse Event Reporting Requirements for DCTD (CTEP and CIP) and DCP INDs and IDEs" which can be downloaded from the CTEP Web site (http://ctep.cancer.gov/protocolDevelopment/electronic_applications/adverse_events.htm). These requirements are briefly outlined in the tables below (Section 7.3.3).

In the rare occurrence when Internet connectivity is lost, a 24-hour notification is to be made to CTEP by telephone at 301-897-7497. Once Internet connectivity is restored, the 24-hour notification phoned in must be entered electronically into CTEP-AERS by the original submitter at the site.

7.3.2 CTEP-AERS is programmed for automatic electronic distribution of reports to the following individuals: Principal Investigator and Adverse Event Coordinator(s) (if applicable) of the Corresponding Organization, the local treating physician, and the Reporter and Submitter. CTEP-AERS provides a copy feature for other e-mail recipients.

The Coordinating Center of the Corresponding Organization is responsible for submitting

to the CTSU documentation of AEs that they deem reportable for posting on the CTSU protocol web page and inclusion on the CTSU bi-monthly broadcast.

7.3.3 Expedited Reporting Guidelines

Use the NCI protocol number and the protocol-specific patient ID assigned during trial registration on all reports.

Note: A death on study requires both routine and expedited reporting regardless of causality. Attribution to treatment or other cause must be provided.

Death due to progressive disease should be reported as **Grade 5 "Disease progression" in the system organ class (SOC) "General disorders and administration site conditions."** Evidence that the death was a manifestation of underlying disease (*e.g.*, radiological changes suggesting tumor growth or progression: clinical deterioration associated with a disease process) should be submitted.

Late Phase 2 and Phase 3 Studies: Expedited Reporting Requirements for Adverse Events that Occur on Studies under an IND/IDE within 30 Days of the Last Administration of the Investigational Agent/Intervention[1, 2]

FDA REPORTING REQUIREMENTS FOR SERIOUS ADVERSE EVENTS (21 CFR Part 312)

NOTE: Investigators **MUST** immediately report to the sponsor (NCI) **ANY** Serious Adverse Events, whether or not they are considered related to the investigational agent(s)/intervention (21 CFR 312.64)

An adverse event is considered serious if it results in **ANY** of the following outcomes:

1) Death
2) A life-threatening adverse event
3) An adverse event that results in inpatient hospitalization or prolongation of existing hospitalization for ≥ 24 hours
4) A persistent or significant incapacity or substantial disruption of the ability to conduct normal life functions
5) A congenital anomaly/birth defect.
6) Important Medical Events (IME) that may not result in death, be life threatening, or require hospitalization may be considered serious when, based upon medical judgment, they may jeopardize the patient or subject and may require medical or surgical intervention to prevent one of the outcomes listed in this definition. (FDA, 21 CFR 312.32; ICH E2A and ICH E6).

ALL SERIOUS adverse events that meet the above criteria **MUST** be immediately reported to the NCI via electronic submission within the timeframes detailed in the table below.

Hospitalization	Grade 1 Timeframes	Grade 2 Timeframes	Grade 3 Timeframes	Grade 4 & 5 Timeframes
Resulting in Hospitalization ≥ 24 hrs	10 Calendar Days			24-Hour 5 Calendar Days
Not resulting in Hospitalization ≥ 24 hrs	Not required		10 Calendar Days	

NOTE: Protocol specific exceptions to expedited reporting of serious adverse events are found in the Specific Protocol Exceptions to Expedited Reporting (SPEER) portion of the CAEPR

Expedited AE reporting timelines are defined as:

- "24-Hour; 5 Calendar Days" - The AE must initially be submitted electronically within 24 hours of learning of the AE, followed by a complete expedited report within 5 calendar days of the initial 24-hour report.

o "10 Calendar Days" - A complete expedited report on the AE must be submitted electronically within 10 calendar days of learning of the AE.
[1]Serious adverse events that occur more than 30 days after the last administration of investigational agent/intervention and have an attribution of possible, probable, or definite require reporting as follows:<br>**Expedited 24-hour notification followed by complete report within 5 calendar days for:**<br>• All Grade 4, and Grade 5 AEs<br>**Expedited 10 calendar day reports for:**<br>• Grade 2 adverse events resulting in hospitalization or prolongation of hospitalization<br>• Grade 3 adverse events<br>[2]For studies using PET or SPECT IND agents, the AE reporting period is limited to 10 radioactive half-lives, rounded UP to the nearest whole day, after the agent/intervention was last administered. Footnote "1" above applies after this reporting period.<br>Effective Date: May 5, 2011

7.3.4 Additional Protocol-Specific Expedited Adverse Event Reporting Exclusions

N/A

7.4 Routine Adverse Event Reporting

All Adverse Events **must** be reported in routine study data submissions. **AEs reported expeditiously through CTEP-AERS must also be reported in routine study data submissions.**

Adverse event data collection and reporting, which are required as part of every clinical trial, are done to ensure the safety of patients enrolled in the studies as well as those who will enroll in future studies using similar agents. AEs are reported in a routine manner at scheduled times during the trial using Medidata Rave. For this trial the Adverse Event CRF is used for routine AE reporting in Rave.

7.5 Secondary Malignancy

A *secondary malignancy* is a cancer caused by treatment for a previous malignancy (*e.g.*, treatment with investigational agent/intervention, radiation or chemotherapy). A secondary malignancy is not considered a metastasis of the initial neoplasm.

CTEP requires all secondary malignancies that occur following treatment with an agent under an NCI IND/IDE be reported expeditiously via CTEP-AERS. Three options are available to describe the event:

- Leukemia secondary to oncology chemotherapy (*e.g.*, acute myelocytic leukemia [AML])
- Myelodysplastic syndrome (MDS)
- Treatment-related secondary malignancy

Any malignancy possibly related to cancer treatment (including AML/MDS) should also be reported via the routine reporting mechanisms outlined in each protocol. For this trial the Adverse Event CRF is used for routine AE reporting in Rave. Submission of the pathology report is not required.

7.6 Second Malignancy

A second malignancy is one unrelated to the treatment of a prior malignancy (and is **NOT** a metastasis from the initial malignancy). Second malignancies require **ONLY** routine AE reporting unless otherwise specified.

7.7 Safety Monitoring Plan

All participants will be carefully followed for safety. Participants are seen by their study doctor and research nurse before each dose of nivolumab (every 2 weeks). Safety evaluations at this time include a physical exam, vital signs, performance status assessment, and safety laboratory tests. The study team will continuously monitor participants for treatment side effects. Participants are instructed to inform their study doctor right away if they notice or feel anything different so the study doctor can check for side effects. The study doctor may be able to provide treatment for side effects. The study doctor may temporarily hold the study drug to reduce side effects. The study doctor will permanently stop the study drug if side effects are too severe and/or long lasting. All participants will be followed for side effects for 100 days from their last dose of nivolumab. Participants with ongoing side effects will continue to be followed until resolution or stabilization of the side effects. Because it is not known if nivolumab will be effective against anal cancer, to enrollment will stop after 12 participants are treated with nivolumab if none of them have their tumors shrink. Study team conferences will be held monthly or more frequently if needed.

8. PHARMACEUTICAL INFORMATION

A list of the adverse events and potential risks associated with the investigational agent administered in this study can be found in Section 7.1.

8.1 CTEP IND Agent

8.1.1 Nivolumab (NSC 748726) (Part A and B)

Amino Acid Sequence: 4 polypeptide chains, which include 2 identical heavy chains with 440 amino acids and 2 identical light chains.

Other Names: BMS-936558, MDX1106

Classification: Anti-PD-1MAb

M.W.: 146,221 daltons

Mode of Action: Nivolumab targets the programmed death–1 (PD-1, cluster of differentiation 279 [CD279]) cell surface membrane receptor. PD-1 is a negative regulatory receptor expressed by activated T and B lymphocytes. Binding of PD-1 to its ligands, programmed death–ligand 1 (PD-L1) and 2 (PD-L2), results in the down-regulation of lymphocyte activation. Nivolumab inhibits the binding of PD-1 to PD-L1 and PD-L2. Inhibition of the interaction between PD-1 and its ligands promotes immune responses and antigen-specific T-cell responses to both foreign antigens as well as self-antigens.

Description: Nivolumab Injection is a clear to opalescent, colorless to pale yellow liquid; light (few) particulates may be present. The drug product is a sterile, nonpyrogenic, single-use, isotonic aqueous solution formulated in sodium citrate, sodium chloride, mannitol, diethylenetriamine pentacetic acid (pentetic acid) and polysorbate 80 (Tween® 80), and water for injection. Dilute solutions of hydrochloric acid and/or sodium hydroxide may be used for pH adjustment (pH 5.5-6.5).

How Supplied: Nivolumab is supplied by Bristol-Myers Squibb and distributed by the Pharmaceutical Management Branch, CTEP/DCTD/NCI as 100 mg vials (10 mg/mL) with a 0.7 mL overfill. It is supplied in 10 mL type I flint glass vials, with fluoropolymer film-laminated rubber stoppers and aluminum seals.

Preparation:

Nivolumab injection is to be administered as an IV infusion through a 0.2-micron to 1.2- micron pore size, low-protein binding (polyethersulfone membrane) in-line filter at the protocol-specified doses and infusion times. It is not to be administered as an IV push or bolus injection. When the dose is based on patient weight (ie, mg/kg), nivolumab injection can be infused undiluted (10 mg/mL) or diluted with 0.9% Sodium Chloride Injection, USP or 5% Dextrose Injection, USP to protein concentrations as low as 0.35 mg/mL. **When the dose is fixed** (eg, **240 mg, 360 mg, or 480 mg flat dose), nivolumab injection can be infused undiluted or diluted** so as **not to exceed a total infusion volume of 160 mL. For patients weighing less than 40 kilograms (kg), the total volume of infusion must not exceed 4 mL per kg of patient weight.**

Storage and Stability: The administration of nivolumab infusion must be completed within 24 hours of preparation. If not used immediately, the infusion solution may be stored under refrigeration conditions (2°C to 8°C, 36°F to 46°F) for up to 24 hours, and a maximum of 8 hours of the total 24 hours can be at room temperature (20°C to 25°C, 68°F to 77°F) and room light. The maximum of 8 hours under room temperature and room light conditions includes the product administration period. The unopened vials can be stored at room temperature (up to 25°C, 77°F) and room light for up to 48 hours.

Stability: Shelf-life surveillance of the intact vials is ongoing.

CAUTION: The single-use dosage form contains no antibacterial preservative or bacteriostatic agent. Therefore, it is advised that the product be discarded 8 hours after initial entry.

Route of Administration: Intravenous infusion over 30 minutes. Nivolumab can be safely infused over 30 minutes in subjects with cancer (Part B only). Do not administer as an IV push or bolus injection.

Method of Administration: Administer through a 0.2 micron to 1.2 micron pore size, low-protein binding polyethersulfone membrane in-line filter.

Potential Drug Interactions: No incompatibilities between nivolumab injection and polyvinyl chloride (PVC), non-PVC/non-DEHP (di[2-ethylhexyl]phthalate) IV components, or glass bottles have been observed.

Availability

Nivolumab is an investigational agent supplied to investigators by the Division of Cancer Treatment and Diagnosis (DCTD), NCI.

Nivolumab is provided to the NCI under a Collaborative Agreement between the Pharmaceutical Collaborator and the DCTD, NCI (see Section 12.3).

8.1.2 Ipilimumab (NSC 732442)

Chemical Name or Amino Acid Sequence: 4 polypeptide chains, 2 identical heavy chains with 447 amino acids and 2 identical light chains consisting of 215 amino acids.

Other Names: MDX-CTLA-4, MDX-010, Yervoy™

Classification: Human monoclonal antibody

M.W.: 147,991 Daltons

Mode of Action: Ipilimumab is specific for the CTLA4 antigen expressed on a subset of activated T-cells. CTLA4 interaction with the B7 molecule, one of its ligands expressed on professional antigen presenting cells, can down-regulate T-cell response. Ipilimumab is, thought to act by blocking the interaction of CTLA4 with the B7 ligand, resulting in a blockade of the inhibitory effect of T-cell activation. The CTLA4/B7 creates the interaction.

Description: Ipilimumab is a fully human immunoglobulin ($IgG_1\kappa$) with two manufacturing processes – ongoing trials have been using substances manufactured using Process B. New clinical trials will be using ipilimumab that is manufactured by Process

C. The Process C has been developed using a higher producing sub-clone of the current Master Cell Bank, and modified cell culture and purification steps.

How Supplied: Bristol-Myers-Squibb (BMS) supplies Ipilimumab to the DCTD/NCI. Ipilimumab injection, 200 mg/40 mL (5 mg/mL), is formulated as a clear to slightly opalescent, colorless to pale yellow, sterile, nonpyrogenic, single-use, isotonic aqueous solution that may contain particles.

Each vial is a Type I flint glass vial with gray butyl stoppers and sealed with aluminum seals.

Preparation: Do not shake. Allow the vials to stand at room temperature for approximately 5 minutes prior to preparation of infusion. Ipilimumab is given undiluted or further diluted in 0.9% NaCl Injection, USP or 5% Dextrose Injection, USP in concentrations between 1 mg/mL and 4 mg/mL. Ipilimumab is stable in a polyvinyl chloride (PVC), non-PVC/non DEHP (di-(2-ethylhexyl) phthalate) IV bag or glass container up to 24 hours refrigerated at (2° to 8° C) or at room temperature/ room light.

Recommended safety measures for preparation and handling include protective clothing, gloves, and safety cabinets.

Storage: Store intact vials refrigerated at (2° to 8° C), protected from light. Do not freeze.

Stability: Shelf-life surveillance of the intact vials is ongoing. Solution as described above is stable up to 24 hours refrigerated at (2° to 8° C) or at room temperature/ room light.

CAUTION: Ipilimumab does not contain antibacterial preservatives. Use prepared IV solution immediately. Discard partially used vials.

Route(s) of Administration: Intravenous infusion over 30 minutes. Do not administer ipilimumab as an IV push or bolus injection.

Method of Administration: Can use a volumetric pump to infuse ipilimumab at the protocol-specific dose(s) and rate(s) via a PVC IV infusion set with an in-line, sterile, non-pyrogenic, low-protein-binding filter (0.2 micron to 1.2 micron).

Availability: Ipilimumab is an investigational agent supplied to investigators by the Division of Cancer Treatment and Diagnosis (DCTD), NCI.

Ipilimumab is provided to the NCI under a Collaborative Agreement between the Pharmaceutical Collaborator and the DCTD, NCI (see Section 12.3).

8.1.3 Agent Ordering and Agent Accountability

8.1.3.1 NCI -supplied agents may be requested by eligible participating investigators (or their authorized designee) at each participating institution. The CTEP-assigned protocol number must be used for ordering all CTEP-supplied investigational agents. The eligible participating investigators at each participating institution must be registered with CTEP, DCTD through an annual submission of FDA Form 1572 (Statement of Investigator), NCI Biosketch, Agent Shipment Form, and Financial Disclosure Form (FDF). If there are several participating investigators at one institution, CTEP-supplied investigational agents for the study should be ordered under the name of one lead participating investigator at that institution.

No starter supplies will be provided. Study agents must be ordered after the patient is enrolled on the assigned treatment arm. If expedited shipment is required, sites should provide an express courier account through the Online Agent Order Processing (OAOP) application.

Active CTEP-registered investigators and investigator-designated shipping designees and ordering designees can submit agent requests through the PMB Online Agent Order Processing (OAOP) application. Access to OAOP requires the establishment of a CTEP Identity and Access Management (IAM) account and the maintenance of an “active” account status, and a “current” password, and active person registration status. For questions about drug orders, transfers, returns, or accountability, call or email PMB any time. Refer to the PMB’s website for specific policies and guidelines related to agent management.

8.1.3.2 Agent Inventory Records – The investigator, or a responsible party designated by the investigator, must maintain a careful record of the inventory and disposition of all agents received from DCTD using the NCI Drug Accountability Record Form (DARF). (See the NCI Investigator’s Handbook for Procedures for Drug Accountability and Storage.)

8.1.3.3 **Investigator Brochure Availability** – The current versions of the IBs for the agents will be accessible to site investigators and research staff through the PMB OAOP application. Access to OAOP requires the establishment of a CTEP IAM account and the maintenance of an “active” account status, a “current” password and active person registration status. Questions about IB access may be directed to the PMB IB Coordinator via email.

8.1.3.4 **Useful Links and Contacts**

- CTEP Forms, Templates, Documents: http://ctep.cancer.gov/forms/
- NCI CTEP Investigator Registration: RCRHelpDesk@nih.gov

- PMB policies and guidelines: http://ctep.cancer.gov/branches/pmb/agent_management.htm
- PMB Online Agent Order Processing (OAOP) application: https://ctepcore.nci.nih.gov/OAOP/
- CTEP Identity and Access Management (IAM) account: https://ctepcore.nci.nih.gov/iam/
- CTEP IAM account help: ctepreghelp@ctep.nci.nih.gov
- IB Coordinator: IBCoordinator@mail.nih.gov
- PMB email: PMBAfterHours@mail.nih.gov

PMB phone and hours of service: (240) 276-6575 Monday through Friday between 8:30 am and 4:30 pm (ET)

9. BIOMARKER, CORRELATIVE, AND SPECIAL STUDIES (PART A ONLY, ***SEE SECTION 10 FOR PART B***)

9.1 Integral Laboratory or Imaging Studies

Not applicable

9.2 Integrated Correlative Studies

Not applicable

9.3 Exploratory Correlative Studies

9.3.1 Exploratory Studies – Methodology

Providers must offer and encourage optional tissue biopsies for tumor tissue for conduction of exploratory studies to all patients on this trial. Blood collections for correlative studies will be mandatory. Tumor tissue and blood samples will be collected for immune monitoring as previously published[54,116-118], under the supervision of the Immunotherapy Platform at MD Anderson. In tumor tissues, immunohistochemical studies will be performed to evaluate CD_4^+ and CD_8^+ T cells and regulatory T cells. In peripheral blood, we will also evaluate T cell populations including, but not limited to, CD4 cells, CD_8^+ cells, and regulatory T cells in pre- and post-therapy samples.

Peripheral blood
Up to 100 mL (within 24 hours) of peripheral blood will be collected for testing of biomarkers described in this clinical protocol at the following time points:

- At screening (any time prior to first dose of nivolumab)
- Before doses 2, 4, and 6
- At treatment discontinuation

For those patients who come off study due to reasons other than progression while on treatment (e.g., excessive toxicity, prolonged treatment break, withdrawal of consent), blood at the time of treatment discontinuation will not be required. The patient's hemoglobin concentration must be ≥ 10.0 g/dL in order for the blood sample to be collected. The treating physician or designee will have the option to cancel the laboratory protocol collection for patient safety without protocol deviation.

Please refer to Appendix E For additional details regarding sample analyses.

9.3.2 Blood Processing and Specimen Storage Management

The MD Anderson Immunotherapy Platform (IMT) has worked on a number of methods testing different factors affecting the yield and quality of peripheral blood mononuclear cells (PBMCs) from blood samples of cancer patients. We have arrived at an optimal method that involves collecting blood in heparinized "Green Top" tubes, processing the blood within 24 hours of the

blood draw and diluting the blood 5 times with D-PBS. All samples are maintained at ambient temperature (room temperature) during transport and processing up until freezing or cell cryopreservation. This method ensures maximum PBMC recovery, especially with patients who are lymphopenic.

An important aspect of our PBMC isolation and manipulation of cells, after thawing for experiments, is the use of an automated viable cell counting instrument (Cellometer; Nexcelom Bioscience, USA) instead of a hemocytometer. This instrument has been shown to be essential for consistent cell counting. We have also tracked the viability of the PBMC after thawing samples processed and frozen from melanoma patients to verify the quality of our cryopreservation techniques. The viabilities have been consistently good (80%-98%) after thawing. At the same time, we have also monitored the time periods between blood draws and processing in the lab and found that shipping cells at room temperature (25ºC) greatly improves cell recovery when processing is carried out within 24 hours.

We have on hand an interactive web-based and powerful software system developed with Aptia Systems (Houston, TX) to track all the cryopreserved cell and serum samples in this trial. The software, called the "Visual Specimen Manager" or "VSM" for short, has its own independent server, backed up hourly, and allows each authorized user to place in or take out vials of samples in a Windows-based interface. It is CFR 21 Part 11 compliant, with only specific users allowed access with passwords. The VSM system is capable of storing all relevant information on each sample stored by simply moving over it with a mouse. In addition, it allows rapid vial labeling and identification of samples by automatically generating a unique bar code. It can also print the specimen information on a cryolabel as the user enters it in a set template. The bar code system then allows for quick identification and localization of the specimens. The system works for liquid nitrogen (LN) tanks as well as -80 freezers.

Immune monitoring screening for blood specimens is mandatory for this study and must occur:
(1) Pre-Treatment: Within 14 days prior to treatment start.
(2) During Treatment: Before nivolumab treatment doses 2, 4, and 6.
(3) At study discontinuation (for patients who come off study due to disease progression while on treatment).

Kits for the immune monitoring part of the protocol will be shipped by the MD Anderson Immunotherapy Platform (IMT) to the enrolling institution prior to enrollment. A total of 2 kits will be sent. All kits are for blood draws as per the protocol and are interchangeable from patient to patient. Samples must be processed as per the immune-specific processing instructions below. The collection, processing, and shipping conditions must be followed.

NOTE: Samples must be shipped to the IMT during the weekdays (Monday-Thursday morning) by overnight priority shipping/courier at room/ambient temperature (20-30 ºC). Blood and serum should not be collected from Thursday afternoon through Friday to avoid an over-the-weekend delay in receipt by the IMT that will compromise sample quality. **Under no circumstances can immune monitoring blood products collected at any site be frozen or placed in any fixative reagent.**

9.3.3 Due to the limitations of fresh tissue sampling in this high impact study, residual blood samples from MDACC and from our ETCTN collaborators will be considered for a noninvasive approach in mutation analysis to optimize all available information we have from all collaborators. For the purpose of this analysis, all correlative blood work is exploratory and will not be used for the purpose of treatment decision-making.

9.3.4 **Description of the Guardant Health Assay (Part A only)**

Guardant360 is a next-generation sequencing (NGS) panel of 70 clinically actionable onco- and tumor suppressor genes utilizing digital sequencing of cell-free circulating tumor DNA (cfDNA) isolated from a simple, non-invasive blood draw. It is medically indicated for the prevention of a repeat invasive biopsy in advanced cancer patients when the initial biopsy is insufficient (QNS) or unavailable/unobtainable as well as when cancer has progressed or recurred despite treatment. The test detects single nucleotide variants via complete exon sequencing in 70 genes, copy number amplifications in 16 genes, small indels in EGFR, ERBB2 and MET exon 14 skipping, and fusions in ALK, FGFR2, FGFR3, RET, ROS1 and NTRK1. The genes are selected because mutations in these genes have FDA-approved matched therapies or are eligible for late phase clinical trials, as well as non-druggable genes with high prevalence alterations that may be helpful in monitoring for molecular response/non-response such as TP53. The panel also includes genomic markers of acquired resistance that may require a change in pharmacotherapy, e.g. EGFR T790M, ALK or ESR1 mutations.

Guardant360 is an advanced diagnostic laboratory test (ADLT) offered by a sole source laboratory certified by the Clinical Laboratory Improvement Amendments (CLIA) for high complexity (molecular pathology) testing and accredited by the College of American Pathology (CAP). Due to high rates of false positives with traditional NGS assays when tumor DNA is in low concentrations, the majority of "liquid biopsy" methods interrogating cell-free DNA have been limited to hotspot analyses. In contrast, the ultra-high specificity (> 99.9999%) of the digital sequencing method enables the sequencing of long, targeted regions (146,000 base pairs) without false positives. Complete exons are sequenced for all exons in 30 genes and the critical exons (those reported as having a somatic mutation in COSMIC) in 40 additional genes. Thus, its key differentiating characteristic from other "liquid biopsy" methods is the ability to sequence complete exons in many genes, in contrast to gene hotspot testing.

Advantages of the Guardant360 cell-free DNA (cfDNA) NGS methodology versus solid tumor tissue-based NGS are:

1. An invasive needle or surgical biopsy is avoided with cfDNA, reducing costs and complications.
2. CfDNA provides a quantitative measure (concentration or mutant allele frequency) of mutations present whereas solid tumor biopsy typically provides a qualitative result (mutation either present or not detected). The quantitative cfDNA result may be followed over time to monitor response to treatment and evolution of acquired resistance.
3. CfDNA sequencing identifies both germline and somatic mutations in the same sample.
4. The assay failure rate is for cfDNA is less than 0.5% (in the first 9,000+ samples) compared to 15%-25% failure rates of tissue-based NGS related

to insufficient quantity of tissue (QNS).

Guardant360 utilizes algorithmic methods to encode and ultimately decode inputs and outputs from massively parallel deep sequencing analysis. By leveraging signal transduction processing technology where voice or image data is digitally encoded before transmission and then decoded post-transmission, this NGS method, known as Digital SequencingTM, enables signal interference to be reduced by two orders of magnitude or more [(88)]. Four validation studies have been published with concordance to tissue biopsy-based genomic testing [(88-91)]. With high enough sensitivity and specificity to robustly quantitate ctDNA from blood, this approach has the potential to evaluate the multiple genomic targets required in NCCN guidelines, to act as a "summary" of the different tumor clones in patients with intra-tumor and inter-tumor heterogeneity, and to prevent the time delays, costs and complications inherent in invasive biopsies. The analytical and clinical validation of Guardant360 is conducted in conformance with evidentiary standards established by the Standards for Reporting of Diagnostic Accuracy (STARD), REporting of tumor MARKer Studies (REMARK), Evaluation of Genomic Applications in Practice and Prevention (EGAPP), and the recent Next-generation Sequencing: Standardization of Clinical Testing (Nex-StoCT) biomarker guidelines [(92-95)].

Methodology

The gene panel was selected to focus on those genomic alterations that are currently actionable defined as being targets of sensitivity or resistance to an FDA-approved matched therapy and/or a targeted therapy in clinical trials. The test simultaneously sequences the 70 cancer-related genes to an average depth of coverage of greater than 8,000X. To summarize, cell-free DNA is extracted from plasma and genomic alterations are analyzed by massively parallel paired end synthesis-by-sequencing of amplified target genes utilizing an Illumina Next-Seq platform complemented by systematic end-to-end process optimization including conversion of cell-free DNA fragments into digital sequences, improvements in the Illumina next generation sequencing process itself, followed by bioinformatics algorithms which enable ctDNA to be measured as a quantitative percentage of total cell-free DNA.

Two 10mls of whole blood are collected in Streck Cell-Free DNA Blood Collection (Streck) tubes, which contain a proprietary formaldehyde-free preservative in that stabilizes white blood cells, preventing the release of genomic DNA and allowing shipping and stability for seven days without need for refrigeration, cold bricks or preliminary centrifugation prior to shipping.

After digital libraries are produced, the sample is sequenced and post-sequencing data is processed using bioinformatics algorithms to quantify the absolute number of unique DNA fragments at a given nucleotide position. This proprietary process is referred to as Digital SequencingTM and enables reporting of the fractional concentration (mutant allele frequency) of a given SNV. Circulating cell-free DNA is mostly derived from leukocyte lysis (germline) and generally a much smaller amount of tumor DNA is derived from cancer cell apoptosis/necrosis. All of the cell-free DNA fragments, including leukocyte-derived and tumor-derived, are simultaneously sequenced with up to single molecule sensitivity. In other words, both tumor DNA and "normal"/germline DNA are sequenced and measured in the same sequencing assay. The fractional concentration or mutant allele frequency for a given mutation is calculated as the

fraction of circulating tumor DNA harboring that mutation in a background of wild-type cell-free DNA fragments. The analytic sensitivity reaches detection of 1-2 single mutant cell-free DNA molecules from a 10 ml blood sample.

Gene list and genomic alterations in the Guardant360 Panel

Guardant360 Panel 2015

All NCCN Somatic Genomic Targets in a Single Test

POINT MUTATIONS - ***Complete*** or Critical Exon Coverage in 70 Genes*

AKT1	*ALK*	***APC***	***AR***	*ARAF*	***ARID1A***	*ATM*	***BRAF***	***BRCA1***	***BRCA2***
CCDN1	***CCND2***	***CCNE1***	*CDH1*	***CDK4***	***CDK6***	***CDKN2A***	***CDKN2B***	*CTNNB1*	***EGFR***
ERBB2	*ESR1*	*EZH2*	*FBXW7*	***FGFR1***	***FGFR2***	*FGFR3*	*GATA3*	*GNA11*	*GNAQ*
GNAS	*HNF1A*	***HRAS***	*IDH1*	*IDH2*	*JAK2*	*JAK3*	***KIT***	***KRAS***	*MAP2K1*
MAP2K2	***MET***	*MLH1*	*MPL*	***MYC***	***NF1***	*NFE2L2*	*NOTCH1*	*NPM1*	***NRAS***
NTRK1	***PDGFRA***	***PIK3CA***	***PTEN***	*PTPN11*	***RAF1***	***RB1***	*RET*	*RHEB*	*RHOA*
RIT1	*ROS1*	*SMAD4*	*SMO*	*SRC*	*STK11*	*TERT*	***TP53***	*TSC1*	*VHL*

AMPLIFICATIONS

AR	*BRAF*	*CCNE1*	*CDK4*	*CDK6*	*EGFR*	*ERBB2*	*FGFR1*
FGFR2	*KIT*	*KRAS*	*MET*	*MYC*	*PDGFRA*	*PIK3CA*	*RAF1*

FUSIONS

ALK	*FGFR2*	*FGFR3*	*RET*	*ROS1*	*NTRK1*

INDELS

EGFR exons 19/20	*ERBB2* exons 19/20	*MET* exon 14 skipping

GUARDANT HEALTH

Serum: 1 Red Top Tube (10 ml)

1. Label tube with Protocol number, collection date/time, protocol time-point collected (e.g. pretreatment, post-treatment), and clearly mark specimen as "serum".
2. Allow one red top tube to clot for 30 minutes at room temperature.
3. Spin in a standard clinical centrifuge at ~2500 RPM for 10 minutes at 4 ºC (preferred). If sites are unable to process samples at 4 ºC then spinning at room temperature is acceptable if done within 2 hours of draw but must be noted.
4. Place tube into Styrofoam container (together with the Green top tubes mentioned below), then into a biohazard bag and then place the bag into the Cardboard shipping (outer) box.
5. Ship to the address below the same day using overnight courier (FedEx), early morning delivery option.

Under no circumstances are collected blood specimens to be frozen or put in any sort of fixative.

PLEASE MAKE SURE THAT EVERY SPECIMEN IS LABELED and include collection time point on the Specimen Transmittal Form (STF).

Whole Blood: 8-9 Green Top Tubes (90 ml)

1. After collection, invert tube(s) multiple times to ensure adequate mixing of Sodium Heparin.
2. Label tube with protocol number, collection date/time, protocol time-point collected (e.g. pretreatment, post-treatment), and clearly mark specimens as "PBMC."
3. Place tubes into Styrofoam container (together with the Red top tube mentioned above), then into a biohazard bag and then place the bag into the Cardboard shipping (outer) box.
4. Ship to the address below the same day using overnight courier (FedEx), early morning delivery option.

Under no circumstances are collected blood specimens to be frozen or put in any sort of fixative.

PLEASE MAKE SURE THAT EVERY SPECIMEN IS LABELED and include collection time point on the STF.

9.3.3 Multicolor Flow Cytometry-Based Lymphocyte Phenotyping

To support the flow cytometry assays in this clinical trial, the IMT has developed robust multicolor flow cytometry (FACS) staining protocols for analyzing lymphocyte subsets in peripheral blood and in tumor isolates. The IMT has developed a standard research protocol (SRP) that details a comprehensive flow cytometry staining panel using validated antibodies (a copy of this SRP can be supplied on request).

Multicolor flow cytometry-based lymphocyte phenotyping will be carried out at: 1) Before nivolumab treatment and 2) Prior to the 2nd, 4th, 6th administration of nivolumab and at progression/discontinuation (if the patient receives as many doses prior to discontinuation).

9.4 Immunohistochemical (IHC) Staining and Analysis for PD-L1, PD-1, CD_8^+, and CD_4^+ Tumor-Infiltrating Lymphocytes (Parts A and B)

Tissue biopsies for exploratory correlative studies are to be offered to each patient on study, yet are optional for enrollment and treatment. Patients who develop thrombocytopenia (platelet count < 50,000/ µL) or are otherwise deemed to be at high risk for bleeding from biopsy by the investigator may have the second biopsy delayed by two additional weeks/ rescheduled to prior to receiving the next dose of nivolumab. If the patient is still deemed to be at too high risk for bleeding, then they may proceed with additional treatment without undergoing the second biopsy. Patients who choose not to undergo tissue biopsy will still be permitted to remain on study, as will patients who are unable to complete a second tissue biopsy due to high risk for bleeding.

Core biopsies under radiographic (ultrasound, CT, or MRI) – guided biopsy will be performed using a lumen no smaller than an 18-gauge needle pretreatment and 2 weeks following initiation of treatment with nivolumab (up to 3 days prior to 3rd dose of nivolumab). 5-8 core samples should ideally be taken, and stored as fresh frozen tissue and as paraffin-embedded tissue, alternating between the two with each successive tissue core biopsy.

Detection of the selected markers for this study will be performed using immunohistochemistry with Dako kits (Agilent Tehcnologies) provided by BMS. These kits will included all necessary reagents including, but not limited to, primary and secondary antibodies, needed in order to perform exploratory biomarker studies. After the staining, the slides will be digitally scanned in an Aperio AT system (Aperio™, Leica Biosystems™) to convert the IHC slides into digital pathology files for posterior analysis. IHC expression analyses of markers consider a thorough staining pattern evaluation including distribution (percentage of positive cells) and intensity in the form of H-score, and evaluating the IHC expression in the proper subcellular location (i.e. membrane, cytoplasm or nucleus). IHC analysis and scoring will be performed by a certified pathologist at MD Anderson using an image analysis software (Image Toolbox, Aperio™).

Outcome from the analysis will be calculated according to the H-score. The H-score ranges from 0 to 300, and it considers both intensity of the IHC (from 0 to 3) and distribution (percentage of the target cells positive, from 0 to 100). The scoring is the addition of the percentages of cells with intensity 0 + intensity 2 + intensity 3, thus the addition of the final percentage is 100% and the scoring will range from 0 to 300. Hence, the H score will incorporate both percentage of positive cells and intensity of marker expression.

9.5 Specimen Submission Summary

9.5.1 Submission of Blood Specimens

9.5.1.1 Handling Instructions

**Specimens for Immune Monitoring (required) for shipment to MD Anderson—Immunotherapy Platform: See Section 9.4**			
**Specimens taken from patient:**	**Collected when:**	**Submitted as:**	**Shipped: <u>Same day as Blood draw.</u>**
SERUM: 10 mL of whole blood in 1 red-top tube and centrifuge	Prior to nivolumab treatment start -at screening or any time prior to first dose of nivolumab), before doses 2,4, 6, and at study end/disease progression	Whole blood samples in red top tubes	Sent at room temperature via overnight carrier to MD Anderson Immunotherapy Platform
WHOLE BLOOD: 90 mL of whole blood in sodium heparin tubes	Prior to nivolumab treatment start -at screening (or any time prior to first dose of nivolumab), before doses 2, 4, 6,, and at study end/disease progression	Whole blood samples in 9 green top tubes (Na Heparin)	Sent at room temperature via overnight carrier to MD Anderson Immune Monitoring Lab

*If the patient discontinues therapy prior to dose 6, then only the blood draws prior to treatment administration and at progression/discontinuation will be required.

9.5.1.2 Shipping Instructions for Blood Specimens

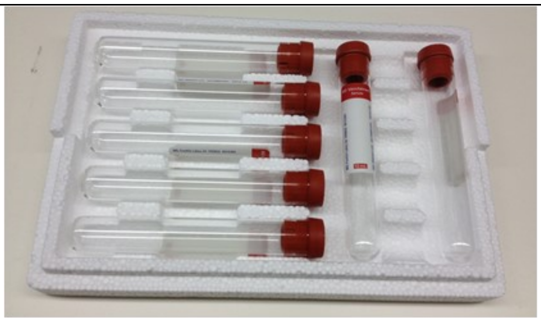

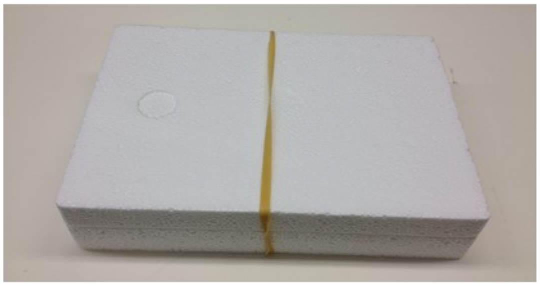

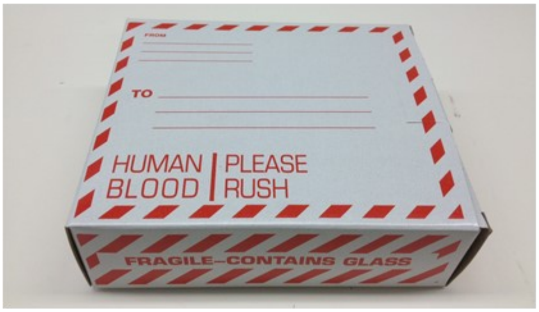

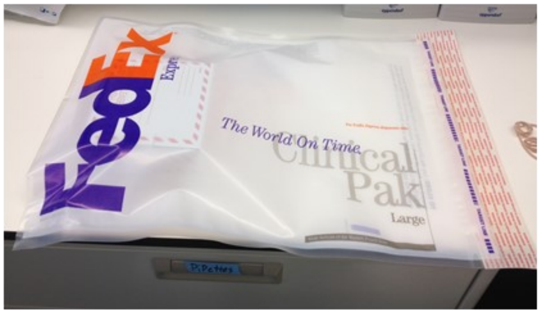

1. Place the blood tubes (red top, green top and PAXgene) in the Styrofoam container as shown in the figure. This figure shows only the positioning of the tubes: the color top does not depict what you will have.

2. Place the rubber band around the Styrofoam container as shown in this figure (this is only for added stability)

3. Place the Styrofoam container into the cardboard shipping (outer) box, and then place the cardboard box into a biohazard bag together with the Absorbent shipping materials.

4. Place everything into the FedEx shipping back and send to the address below (blood spesimens only):
   Immunotherapy Platform
   MD Anderson Cancer Center
   Attention: Karen Millerchip/Jaimol Peedikayil
   SCR3.3208
   7455 Fannin St.
   Houston, TX 77054-1901

9.5.2 Submission of Slides for IHC Analysis

9.5.2.1 Handling Instructions

Specimens for Central Pathology Review
for shipment to MD Anderson—Jorge Blando: See Section 9.5.2.2

**Specimens taken from patient:**	**Collected when:**	**Submitted as:**	**Shipped: <u>after paraffin embedding/slice cutting.</u>**
6-8 unstained tumor slides per biopsy or time point	Two separate biopsies: one prior to treatment initiation and another after 2 doses of treatment with nivolumab (i.e., up to 3 days prior to 3rd dose of nivolumab).	Paraffin-embedded unstained tumor slides	Overnight mail to Dr. Jorge Blando

9.5.2.2 Shipping address for IHC samples only:

Immunopathology Laboratory
Immunotherapy Platform (IMT)
UT MD Anderson Cancer Center,
Life Science Plaza Building
2130 W. Holcombe Blvd,
Unit 2951, Houston, TX 77030

9.6 Special Studies - Analysis for the Presence of HPV

When available, HPV testing, if not previously performed, will be conducted on archival tissue for each patient at the participating site treating each particular patient. The availability of remaining tissue for HPV testing is not required for participation on this study. Testing will include detection of HPV DNA by in-situ hybridization and/or detection of p16 by immunohistochemistry. Formalin-fixed, paraffin-embedded samples will be deparaffinized in preparation for DNA extraction. To determine whether or not HPV had been incorporated into

the host genome, in-situ hybridization will be utilized to assess for positive hybridization in the tumor cell nuclei using probes for various oncogenic subtypes of HPV. To determine whether or not p16 is expressed in tumor cells, IHC analysis using monoclonal antibodies against the p16 protein will performed. Tumors will considered to express p16 if 5% or greater of tumor cells demonstrate p16 staining on IHC. Patients will be considered to have HPV-positive tumors if HPV DNA is detected by in-situ hybridization and/or p16 is detected in the tumor cells by IHC. Patients will be deemed to have HPV-negative tumors if HPV DNA is absent and p16 is not detected.

9.7 Optional Banking of Residual Tissue and Blood Samples for Future Research

All patients who sign consent for this study also will be asked to consider participation in this optional part of the study. In consenting patients, any remaining blood and tissue samples not used for biomarker assessment will be banked for potential future research. The samples will be banked in the Immunotherapy Platform Laboratory, The University of Texas MD Anderson Cancer Center, South Campus Research Bldg SCR3.3208, 7455 Fannin Street, Houston, TX 77054.

IRB approval will be obtained prior to the use of the banked samples for any research not described in this protocol. The samples will be given a code number. No identifying information will be directly linked to the samples. Only the research team in charge of the bank will have access to the code numbers and be able to link the samples to the subject. If a patient withdraws his/her consent for banking of samples, the banked samples will be destroyed. However, if any samples were previously used for research prior to the withdrawal of consent, the samples will not be able to be destroyed.

10. PART B – CORRELATIVE AND BLOOD WORK:

We will quantitatively assess multiple immune markers in FFPE tumor histology sections, including immunohistochemistry (IHC) PD-L1 expression in malignant and inflammatory cells, as well as by immunofluorescence (mIF) TIL's, macrophages and myeloid cell sub-populations. All antibodies used will be optimized for IHC/MIF by examination of positive and negative controls and testing of the biomarker, correlative, and special studies.

10.1 Integral Laboratory or Imaging Studies

N/A

10.2 Integrated Correlative Studies

N/A

10.3 Exploratory Correlative Studies

10.3.1 Exploratory Studies – Methodology

Providers must offer and encourage optional tissue biopsies for tumor tissue for conduction of exploratory studies to all patients on this trial. Blood collections for correlative studies will be mandatory. Tumor tissue and blood samples will be collected for immune monitoring as previously published[54,116-118], under the supervision of the Translational Immunotherapy Platform/Molecular Pathology Platform at MD Anderson. In tumor tissues, immunohistochemical studies will be performed to evaluate CD_4^+ and CD_8^+ T cells and regulatory T cells. In peripheral blood, we will also evaluate T cell populations including, but not limited to, CD4 cells, CD_8^+ cells, and regulatory T cells in pre- and post-therapy samples.

Peripheral blood
Up to 70 mL (within 24 hours) of peripheral blood will be collected for testing of biomarkers described in this clinical protocol at the following time points:

- At screening.
- After cycle #1 (between weeks 9-10, one cycle = 8 weeks).
- At treatment discontinuation.

For those patients who come off study due to reasons other than progression while on treatment (e.g., excessive toxicity, prolonged treatment break, withdrawal of consent), blood at the time of treatment discontinuation will not be required. The patient's hemoglobin concentration must be ≥ 10.0 g/dL in order for the blood sample to be collected. The treating physician or designee will have the option to cancel the laboratory protocol collection for patient safety without protocol deviation.

Please refer to Appendix E For additional details regarding sample analyses.

10.3.2 Tissue Collection and processing

Fresh and formalin-fixed and paraffin-embedded (FFPE) core needle biopsies (CNBs) tumor tissues will be used for immune and molecular profiling. Core biopsy is typically performed using 21-18 gauge needles and, with conditions permitting, up to 5 cores should be collected, including two cores (#1-2) for overnight FFPE processing, and 3 additional cores (#3-5) as fresh tumor tissue that will be immediately frozen. In at least 20 cases, one fresh tumor tissue core (core #3) will be processed for TILs isolation and flow cytometry analysis. Up to 60 ml of whole blood will be collected at each time point and processed for isolation of plasma (for future analysis of, for example, circulating tumor DNA), and PMBCs for 14-color flow cytometry using methods previously published. All tissue specimens collected will be reviewed by reference pathologists. The following quality control activities for specimens collected will be performed: a) histology/cytology examination of the tissues and cells to confirm the presence of tumor cells, as well as their abundance; b) assessment of fresh tissue specimens for extraction of DNA and RNA, and to prepare histology specimens for immunohistochemistry (IHC), multiplex Immunofluorescence (mIF) and gene expression analysis of immune-oncology panels; and, c) assessment of DNA/RNA extracted. All histology stained slides will be scanned and digital images will be available for review.

Core needle biopsy (CNB): If FFPE and a fresh frozen diagnostic core needle biopsy (CNB) specimen is not sufficient for biomarker analysis, a fresh CNB will obtained solely with the

purpose of research studies (pre-treatment samples) and sent to the MD Anderson Institutional Tissue Bank at the Department of Translational Molecular Pathology (TMP, Ignacio I Wistuba) immediately after collection. The number of cores obtained will be affected by the patient clinical condition at the time of biopsy and determined by the radiologist who is performing the procedure. Up to 5 tissue cores should be collected, including 2 for clinical processing and 3 additional passes will be attempted to obtain the research core samples. These cores will be processed for:

<u>Cores 1 and 2</u>: Immediate and overnight fixation in 10% buffered formalin for paraffin embedding, usually within 20-24 hour after fixation. For biopsies performed on Friday, fixation time may extend to 48 hours (FFPE samples)

<u>Cores 3 - 5:</u> Flash freezing in liquid nitrogen for RNA-sequencing. If an additional core is available will be flash freezing in liquid nitrogen as back up. In at least 20 cases, core #3 will be processed fresh for flow cytometry analysis.

When the amount of tissue is more limited, priority will be assigned to FFPE analyses and RNA sequencing (at least 2 FFPE cores and at least 1 fresh frozen core). The FFPE sample is important as it also provides a histological confirmation for the presence and cellularity of tumor cells. FFPE samples will also be prioritized for immune gene expression profiling by exploratory immunohistochemistry assay, multiplex immunofluorescence (IF) and biobanking for other to be determined assays.

<u>For patients NOT participating in optional tissue collection, submission of ARCHIVAL tissue specimens are recommended:</u>

For patients who do NOT consent to undergo a biopsy (or are unable to do so) as part of this trial or for whom adequate tumor specimen is unavailable from the trial biopsies, **archival FFPE** tissue (<u>tumor with or without normal tissue</u>) may be submitted for translational studies.

- Either (1) one H&E slide with 10-15 unstained slides – all 4 micron thickness

OR

- (2) an FFPE block of MALIGNANT tumor may be mailed (to the address listed below) for further genomic and immune biomarker analysis.

Here, a tumor specimen with the **<u>largest</u>** amount of malignant tumor present should be prioritized and selected for optional specimen submission.

In addition, (1) one H&E slide with 10-15 unstained slides – all 4 micron thickness – or (2) an FFPE block of normal, NON-MALIGNANT tissue should be submitted if available.

Archival samples from patients who are deceased at the time of retroactive sample request may also be submitted to the address below for further analysis (due to waiver of consent).

All FFPE tissue should be mailed to:

University of Texas-MD Anderson Cancer Center
Ship AMBIENT To: MDACC
Attn: Institutional Tissue Bank (ITB)
1515 Holcombe Blvd, Rm G1.3586
Houston, TX 77030
T: 713.745.7047

10.3.3 Blood Processing and Specimen Storage Management

The MD Anderson Immunotherapy Platform (IMT) has worked on a number of methods testing different factors affecting the yield and quality of peripheral blood mononuclear cells (PBMCs) from blood samples of cancer patients. We have arrived at an optimal method that involves collecting blood in heparinized "Green Top" tubes, processing the blood within 24 hours of the blood draw and diluting the blood 5 times with D-PBS. All samples are maintained at ambient temperature (room temperature) during transport and processing up until freezing or cell cryopreservation. This method ensures maximum PBMC recovery, especially with patients who are lymphopenic.

An important aspect of our PBMC isolation and manipulation of cells, after thawing for experiments, is the use of an automated viable cell counting instrument (Cellometer; Nexcelom Bioscience, USA) instead of a hemocytometer. This instrument has been shown to be essential for consistent cell counting. We have also tracked the viability of the PBMC after thawing samples processed and frozen from melanoma patients to verify the quality of our cryopreservation techniques. The viabilities have been consistently good (80%-98%) after thawing. At the same time, we have also monitored the time periods between blood draws and processing in the lab and found that shipping cells at room temperature (25ºC) greatly improves cell recovery when processing is carried out within 24 hours.

We have on hand an interactive web-based and powerful software system developed with Aptia Systems (Houston, TX) to track all the cryopreserved cell and serum samples in this trial. The software, called the "Visual Specimen Manager" or "VSM" for short, has its own independent server, backed up hourly, and allows each authorized user to place in or take out vials of samples in a Windows-based interface. It is CFR 21 Part 11 compliant, with only specific users allowed access with passwords. The VSM system is capable of storing all relevant information on each sample stored by simply moving over it with a mouse. In addition, it allows rapid vial labeling and identification of samples by automatically generating a unique bar code. It can also print the specimen information on a cryolabel as the user enters it in a set template. The bar code system then allows for quick identification and localization of the specimens. The system works for liquid nitrogen (LN) tanks as well as -80 freezers.

Immune monitoring screening for blood specimens is mandatory for this study and must occur:
(1) Pre-Treatment: Within 14 days prior to treatment start.
(2) During Treatment: Before cycle #2.
(3) At Progression of Disease.

Kits for the immune monitoring part of the protocol will be shipped by the Department of Translational Molecular Pathology at MD Anderson Cancer Center (Houston, Texas) to the enrolling institution prior to enrollment. To request for kits, email Beatriz Sanchez Espiridion (bsanchez2@mdanderson.org). Include in the email your CTEP site code, PI, receiving individual, and address. A total of 2 kits will be sent. All kits are for blood draws as per the protocol and are interchangeable from patient to patient. Samples must be processed as per the immune-specific processing instructions below. The collection, processing, and shipping conditions must be followed.

NOTE: Samples must be shipped to the Institutional Tissue Bank, Department of Translational Molecular Pathology, at MD Anderson Cancer Center during the weekdays (Monday-Thursday morning) by overnight priority shipping/courier at room/ambient temperature (20-30 ºC). Blood and serum should not be collected from Thursday afternoon through Friday to avoid an over-the-weekend delay in receipt by the Institutional Tissue Bank that will compromise sample quality. **Under no circumstances can immune monitoring blood products collected at any site be frozen or placed in any fixative reagent.**

For the purpose of this analysis, all correlative blood work is exploratory and will not be used for the purpose of treatment decision-making. **Serum: 1 Red Top Tube (10 ml)**

1. Label tube with Protocol number, collection date/time, protocol time-point collected (e.g. pretreatment, post-treatment), and clearly mark specimen as "serum".
2. Allow one red top tube to clot for 30 minutes at room temperature.
3. Spin in a standard clinical centrifuge at ~2500 RPM for 10 minutes at 4 ºC (preferred). If sites are unable to process samples at 4 ºC then spinning at room temperature is acceptable if done within 2 hours of draw but must be noted.
4. Place tube into Styrofoam container (together with the Green top tubes mentioned below), then into a biohazard bag and then place the bag into the Cardboard shipping (outer) box.
5. Ship to the address below the same day using overnight courier (FedEx), early morning delivery option.

Under no circumstances are collected blood specimens to be frozen or put in any sort of fixative.

PLEASE MAKE SURE THAT EVERY SPECIMEN IS LABELED and include collection time point on the Specimen Transmittal Form (STF).

Whole Blood: 6 Green Top Tubes (60 ml)

1. After collection, invert tube(s) multiple times to ensure adequate mixing of Sodium Heparin.
2. Label tube with protocol number, collection date/time, protocol time-point collected (e.g. pretreatment, post-treatment), and clearly mark specimens as "PBMC."
3. Place tubes into Styrofoam container (together with the Red top tube mentioned above), then into a biohazard bag and then place the bag into the Cardboard shipping (outer) box.
4. Ship to the address below the same day using overnight courier (FedEx), early morning delivery option.

Under no circumstances are collected blood specimens to be frozen or put in any sort of fixative.

PLEASE MAKE SURE THAT EVERY SPECIMEN IS LABELED and include collection time point on the STF.

10.3.4 Correlative Biomarker Sample Analyses

Histology: H&E-stained sections from CNBs will be used to confirm the presence of tumor cells, as well as their abundance (tumor cellularity), stromal components and lymphocytic infiltrates. Hematoxylin and eosin (H&E)-stained sections from all FFPE diagnostic slides (tumor, normal and lymph nodes) will be scanned in Aperio™ digital pathology scanner analysis for pathological evaluation and selection of 1 or 2 blocks (depending on tumor availability) for biomarker analysis. From the CNB specimens, the following pathological analysis will be performed: 1) tumor diagnosis; 2) lowest degree of tumor differentiation; 3) percentage of areas of necrosis; 4) percentage of areas of fibrosis; 5) percentage of viable tumor tissue; and, 6) percentage of viable malignant cells.

Central Review: The scanned H&E-stained slides will be available for pathology analysis at the TMP Pathology and Biomarker Lab (TMP-IL) chaired by Dr. Ignacio I. Wistuba, MD Anderson Cancer Center.

Quality Control (QC): All tissue specimens collected will be reviewed by reference pathologists. At least, three types of QC activities for specimens collected will be performed: a) histology/cytology examination of the tissues and cells; b) tissue quality assessment of fresh specimens for extraction of DNA, RNA and proteins, and to prepare histology specimens such as whole sections for immunohistochemistry and immunofluorescence; and, c) quality assessment of DNA, RNA and protein extracted. All histology stained samples will be scanned and digital images will be available for review.

HPV Testing: PCR-based HPV detection and genotyping (Cervista assay, Holoantibodies standard methods, including Western blotting.

Immune Biomarker Testing: All pathology slides will be scanned into a digital image scanner and analyzed using image analysis software: IHC analysis will be performed using the Aperio Image Toolbox™ (Leica Biosystems) and IF analysis using the Vectra Inform™ (Perkin-Elmer) software. Five random 1-mm square areas within the tumor region will be selected for analysis. PD-L1 expression of markers in malignant cells will be evaluated using the Aperio™ digital H-score system which includes the percentage of positive cells (0 to 100) and intensity (0 to 3+), with a total score ranging from 0 to 300. For mIF analysis, we will use the Opal chemistry and multispectral microscopy Vectra system (Perkin-Elmer) and run two Vectra mIF panels. On the multiplex IHC system, each multiplex panel includes 6 to 7 antibodies that are stained on the same slide and labeled using a tyramide-signal amplification (TSA)-based kit (PerkinElmer). The scoring of PD-*L1* is expressed as percentage of positive cells in the tumor cell and in the tumor associated macrophages compartments defined by immune-labeling, e.g., tumor epithelial cells labeled with cytokeratins (AE1/AE3+ PDL1+) and macrophages (CD68+ PDL1+). T-cell subpopulations are evaluated as cell density (number of positive cells by mm square of tumor area) and also defined by co-expression of specific markers, e.g., T-helper (CD3+CD4+), T-cytotoxic (CD3+CD8+). Additional immune-markers are also expressed as cell density. Finally, the data is consolidated using Spotfire software (PerkinElmer) for the final report including percentage of tumor cells expressing PDL-1, percentage of tumor associated macrophages expressing PDL-1, and cell density for intratumoral T-cell infiltrates (including PD1, T-reg, T-memory, NK and cytotoxic) for each tumor case.

Nanostring Immune Oncology Pancancer Panel: Immune-related genes will be examined using RNA extracted from FFPE tumor tissue histology sections using 200ng of RNA, as previously published[119]. This panel includes 770 gene and combines markers for 24 different immune cell types and populations, 30 common cancer antigens and genes that represent all categories of immune response including key checkpoint blockade genes.

Whole exome sequencing (WES) and RNA-sequencing: Nucleic acids extraction from fresh frozen tumor tissue and PMBCs will be performed using standard methods. DNA and RNA quantity and integrity will be assessed using NanoDrop 1000 spectrophotometer (Nanodrop

Technologies) and Pico-green analyses. WES will be conducted to a minimum of 200-fold sequencing depth. Somatic mutations will be called using Mutect for point mutations \ and Pindel for insertions and deletions. A subset of variants will be re-sequenced using NGS of targeted PCR products to assess quality and false positive rates as previously described. Mutations will be further analyzed *in silico* to predict whether they would produce potentially immunogenic neoantigens using NetMHCpan. mRNA from each tumor will be extracted and sequenced by Illumina HiSeq, and gene expression will be quantified for the transcript models corresponding to the TCGA GAF2.1, using RSEM.

<u>Flow cytometry</u>: a 14-color panels will focus on a) delineation of major immune cell types (T cells, B cells, NK cells, DC); b) determination of T cell differentiation status and limited functionality (IFNγ, TNFα, GB); and, c) defining the expression level of co-stimulatory and co-inhibitory molecules on T cells and their respective receptors such as PD-L1 on infiltrating myeloid cells or tumor. Briefly, 50cc of heparinized peripheral blood will be processed fresh (within 24h of being drawn) for PBMCs isolation by MDACC ITB. PBMCs will be cryopreserved and stored in liquid nitrogen until use for cytometric analysis. All time points belonging to a patient will be stained and acquired at the same time to avoid any technical variation. In a subset of fresh tumor tissues, flow cytometry will be performed. Tumor tissue will be stored in HBSS for up to 24h before processing for flow cytometry. Fresh tissue will be mechanically disaggregated or digested according to needs and panel design. The cells will be processed as a single cell suspension tissue samples will be stained for surface and/or intracellular markers using standard procedures. Stained samples will be fixed and acquired on a flow cytometer (BD Fortessa X-20 or Canto II, BD Biosicence, San Jose, CA) within one week and analyzed using FlowJo software.

10.4 Specimen Submission Summary

**Specimens for Immune Monitoring (required) for shipment to Institutional Tissue Banks (ITB) MD Anderson—Department of Translational Molecular Pathology**			
**Specimens taken from patient:**	**Collected when:**	**Submitted as:**	**Shipped: <u>Same day as Blood draw.</u>**
SERUM: 10 mL of whole blood in 1 red-top tube and centrifuge	Prior to nivolumab/ipilimumab treatment start -at screening or any time prior to first dose and before doses 2,and at study end/disease progression	Whole blood samples in red top tubes	Sent at room temperature via overnight carrier to MD Anderson (Institutional Tissue Bank).
WHOLE BLOOD: 60 mL of whole blood in sodium heparin tubes	Prior to nivolumab/ipilimumab treatment start -at screening (or any time prior to first dose of nivolumab), before dose 2, and at study end/disease progression	Whole blood samples in 6 green top tubes (Na Heparin)	Sent at room temperature via overnight carrier to MD Anderson (Institutional Tissue Bank)
**Specimens taken from patient:**	**Collected when:**	**Submitted as:**	**Shipped: <u>after paraffin embedding/slice cutting.</u>**
10 unstained tumor slides or FFPE block per biopsy or time point	Two separate biopsies: one prior to treatment initiation and another after 2 doses of treatment with nivolumab (i.e., up to 3 days prior to 3rd dose of nivolumab).	Paraffin-embedded unstained tumor slides	Sent at room temperature via overnight carrier to MD Anderson (Institutional Tissue Bank)
(1)10-15 unstained slides of malignant and 10-15 unstained normal slides or (2) FFPE blocks from archival malignant and normal tissue	Archival specimens (for patients not participating in the optional biopsies or for patients in whom adequate malignant tissue is not available for biomarker analysis)	Paraffin-embedded unstained slides or block	Sent at room temperature via overnight carrier to MD Anderson (Institutional Tissue Bank)

10.4.1 Shipping Instructions for Blood Specimens

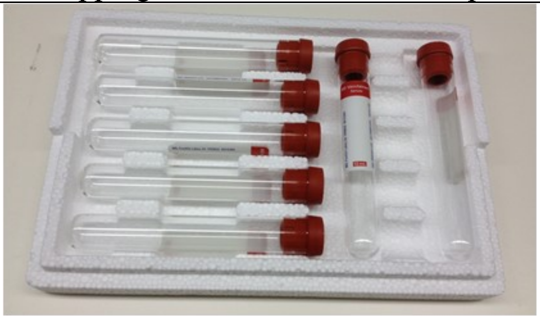

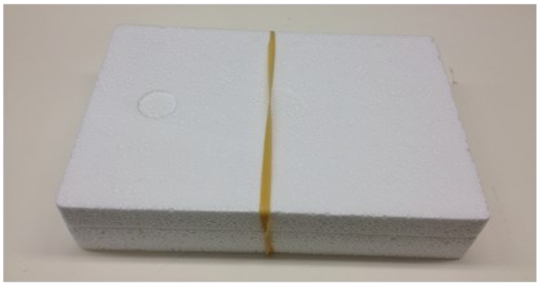

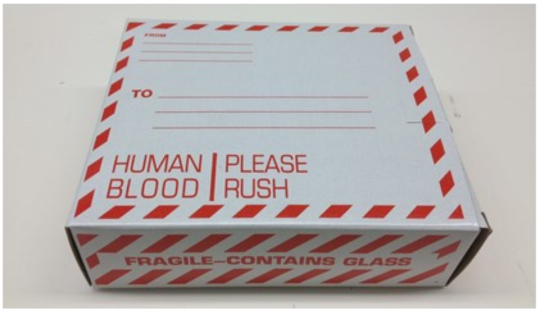

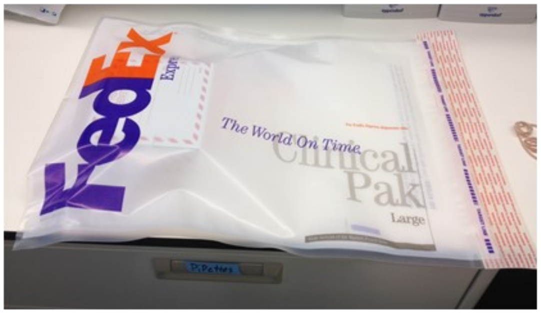

5. Place the blood tubes (red top, green top and PAXgene) in the Styrofoam container as shown in the figure. This figure shows only the positioning of the tubes: the color top does not depict what you will have.

6. Place the rubber band around the Styrofoam container as shown in this figure (this is only for added stability)

7. Place the Styrofoam container into the cardboard shipping (outer) box, and then place the cardboard box into a biohazard bag together with the Absorbent shipping materials.
8. Place everything into the FedEx shipping back and send to the address below (blood specimens only):

10.4.2 Submission of Slides for IHC Analysis

University of Texas-MD Anderson Cancer Center
Ship AMBIENT To: MDACC
Attn: Institutional Tissue Bank (ITB)
1515 Holcombe Blvd, Rm G1.3586
Houston, TX 77030
T: 713.745.7047

10.5 Optional Banking of Residual Tissue and Blood Samples for Future Research

All patients who sign consent for this study also will be asked to consider participation in this optional part of the study. In consenting patients, any remaining blood and tissue samples not used for biomarker assessment will be banked for potential future research. The samples will be banked in the Institutional Tissue Bank (ITB), at The University of Texas MD Anderson Cancer Center, South Campus Research Bldg SCR3.3208, 7455 Fannin Street, Houston, TX 77054.

IRB approval will be obtained prior to the use of the banked samples for any research not described in this protocol. The samples will be given a code number. No identifying information will be directly linked to the samples. Only the research team in charge of the bank will have access to the code numbers and be able to link the samples to the subject. If a patient withdraws his/her consent for banking of samples, the banked samples will be destroyed. However, if any samples were previously used for research prior to the withdrawal of consent, the samples will not be able to be destroyed.

11. STUDY CALENDAR

11.1 Part A:

Baseline evaluations are to be conducted within 1 week prior to start of protocol therapy. Scans and x-rays must be done $\leq$4 weeks prior to the start of therapy. Unless otherwise noted, a standard window of -1 day to + 2 days will be considered acceptable for all testing and evaluations (will not be considered study deviation). In the event that the patient's condition is deteriorating, laboratory evaluations should be repeated within 48 hours prior to initiation of the next dose of therapy.

	Pre-Study	Wk 1	Wk 2	Wk 3	Wk 4	Wk 5	Wk 6	Wk 7	Wk 8	Wk 9	Wk 10	Wk 11[h]	Wk 12	Treatment Discontinuation Visit	28 Days from Last Dose of Nivolumab	30 Days from End of Treatment Follow-up	100 Days from Last Dose of Nivolumab Follow-up
Nivolumab		A		A		A		A		A		A					
Informed consent	X																
Demographics	X																
Medical history	X																
Concurrent medications	X	X[g]		X		X		X		X		X					
Physical exam	X	X[g]		X		X		X		X		X		X	X		
Vital signs	X	X[g]		X		X		X		X		X		X	X		
Height	X																
Weight	X	X		X		X		X		X		X		X	X		
Performance status	X	X[g]		X		X		X		X		X		X	X		
CBC w/diff, plts	X	X[g]		X		X		X		X		X		X	X		
Serum chemistry[a]	X	X[g]		X		X		X		X		X		X	X		
Carcinoembryonic antigen	X					X				X				X	X	X	
Thyroid stimulating hormone[b]	X													X			
HIV Antibody[c]	X																
HIV Viral Load[d]	X			X				X				X		X	X		

	Pre-Study	Wk 1	Wk 2	Wk 3	Wk 4	Wk 5	Wk 6	Wk 7	Wk 8	Wk 9	Wk 10	Wk 11[h]	Wk 12	Treatment Discontinuation Visit	28 Days from Last Dose of Nivolumab	30 Days from End of Treatment Follow-up	100 Days from Last Dose of Nivolumab Follow-up
Hepatitis B Panel[e]	X																
Hepatitis C Viral Antibody	X																
EKG (as indicated)	X																
EKG and ECHO [p]	X[p]																
EKG, CPK, troponin, ECHO [q]	X[q]	X[q]															
Adverse event evaluation		X---X												X	X	X[j]	X[j]
Tumor measurements	X	Tumor measurements are repeated every 6 weeks. Documentation (radiologic) must be provided for patients removed from study for progressive disease.												X			
Radiologic evaluation: CT or MRI of Chest, Abdomen, Pelvis & other known or suspected sites of disease	X	Radiologic measurements should be performed every 6 weeks												X			
B-HCG	X[f]	Serum or urine pregnancy test must be performed every 6 weeks													X[f]	X[f]	
HPV testing	X[o]																
Blood for Correlative Studies[m]	X			X				X				X		X			
Tumor Biopsies for Correlative Studies[n]	X					X[l]											
Survival status																X[k]	

A: Nivolumab IV 3 mg/kg given every 2 weeks +/- 3 days.
a: Albumin, alkaline phosphatase, total bilirubin, bicarbonate, BUN, calcium, chloride, creatinine, glucose, LDH, phosphorus, potassium, total protein, SGOT [AST], SGPT [ALT], sodium.
b: Patients with TSH > 10.0 U/mL or TSH < 0.5 U/mL must be referred to an endrocrinologist with correction in a range between 0.5-10.0 U/mL prior to treatment initiation.
c: If not tested within past 6 months.
d: Only for patients with HIV infection as documented by the presence of a positive HIV antibody test.
e: Hepatitis B surface antibody, Hepatitis B surface antigen, hepatitis B core antibody.
f: Serum or urine pregnancy test (women of childbearing potential) must be performed with 24 hours prior to start of nivolumab, then every 8weeks. After discontinuation from nivolumab these should be repeated at approximately 30 days and approximately 70 days.
g. Tests/evaluations do not need to be repeated prior to first nivolumab dose if performed within past 7 days.
h. Nivolumab may continue to be given every 4weeks until any of the criteria outlined in section 5 require it be stopped.
i: Off-treatment evaluation, only for patients who discontinue treatment due to disease progression.
j. All patients will be followed for adverse events for 100 days from last dose of nivolumab via phone call. Patients with ongoing adverse event(s) will continue to be

	Pre-Study	Wk 1	Wk 2	Wk 3	Wk 4	Wk 5	Wk 6	Wk 7	Wk 8	Wk 9	Wk 10	Wk 11[h]	Wk 12	Treatment Discontinuation Visit	28 Days from Last Dose of Nivolumab	30 Days from End of Treatment Follow-up	100 Days from Last Dose of Nivolumab Follow-up
followed via phone call(s) until resolution or stabilization of the adverse event. Patients who discontinue therapy due to unacceptable toxicity but have not demonstrated radiographic disease progression will continue to be followed on schedule every 8 weeks with imaging studies until disease progression is documented.<br>k. Patients will be followed for survival status every 3 months for 2 years after treatment discontinuation or until death, whichever occurs first. Follow up may be accomplished by clinic visit, medical record review, phone contact or email.<br>l. 2 weeks after initiation of treatment with nivolumab (up to 3 days prior to 3rd dose of nivolumab).<br>m. Peripheral blood will be collected as described in section 9.3 at screening (any time prior to first dose of nivolumab) and before doses 2, 4, 6, and at treatment discontinuation (for patients who progress while on nivolumab). Blood should not be collected if the hemoglobin concentration is < 10.0 g/dL.<br>n. Optional, but not required, for participation on study.<br>o. If not previously performed, DNA in-situ hybridization and/or p16 by IHC testing if sufficient archival tissue is available will be performed at enrolling site.<br>p. For any patients with a history of CHF or at risk because of underlying cardiovascular disease or exposure to cardiotoxic drugs as clinically indicated.<br>q. For patients with evidence of CHF, MI, cardiomyopathy, or myositis cardiac evaluation including lab tests and cardiology consultations as clinically indicated including EKG, CPK, troponin, ECHO cardiogram.																	

11.2 Part B:

Baseline evaluations are to be conducted within 1 week prior to start of protocol therapy. Scans and x-rays must be done ≤4 weeks prior to the start of therapy. Unless otherwise noted, a standard window of -1 day to + 2 days will be considered acceptable for all testing and evaluations (will not be considered study deviation). In the event that the patient's condition is deteriorating, laboratory evaluations should be repeated within 48 hours prior to initiation of the next dose of therapy. Note: The below study calendar presents an example of the first 13 weeks on treatment, which will be repeated every 13 weeks. For example, Week 14 should follow Week 1 schedule, Week 15 should follow Week 2 schedule, and so forth until criteria for treatment discontinuation is met.

	Pre-Study	Wk 1	Wk 2	Wk 3	Wk 4	Wk 5	Wk 6	Wk 7	Wk 8	Wk 9	Wk 10	Wk 11[h]	Wk 12	Wk 13	Treatment Discontinuation Visit[i]	28 Days from Last Dose of Nivolumab and Ipilimumab	30 Days from End of Treatment Follow-up	100 Days from Last Dose of Nivolumab and Ipilimumab Follow-up
Nivolumab		A				A				A				A				
Ipilimumab*		B								B								
Informed consent	X																	
Demographics	X																	
Medical history	X																	

	Pre-Study	Wk 1	Wk 2	Wk 3	Wk 4	Wk 5	Wk 6	Wk 7	Wk 8	Wk 9	Wk 10	Wk 11[h]	Wk 12	Wk 13	Treatment Discontinuation Visit[i]	28 Days from Last Dose of Nivolumab and Ipilimumab	30 Days from End of Treatment Follow-up	100 Days from Last Dose of Nivolumab and Ipilimumab Follow-up
Concurrent medications	X	X[g]				X				X				X				
Physical exam	X	X[g]				X				X				X	X	X		
Vital signs	X	X[g]				X				X				X	X	X		
Height	X																	
Weight	X	X				X				X				X	X	X		
Performance status	X	X[g]				X				X				X	X	X		
CBC w/diff, plts	X	X[g]				X				X				X	X	X		
Carcinoembryonic antigen	X					X				X				X	X	X		
Serum chemistry[a]	X	X[g]				X				X				X	X	X		
Thyroid panel[b]	X									X					X			
HIV Antibody[c]	X																	
HIV Viral Load[d]	X					X				X					X	X		
Hepatitis B Panel[e]	X																	
Hepatitis C Viral Antibody	X																	
EKG (as indicated)	X																	
EKG and ECHO [p]	X[p]																	
EKG, CPK, troponin, ECHO [q]	X[q]			X[q]														
Adverse event evaluation		X---X													X	X	X[j]	Xj
Tumor measurements	X	Tumor measurements are repeated every 8 weeks. Documentation (radiologic) must be provided for patients removed from study for progressive disease.													X			
Radiologic evaluation: CT or MRI of Chest, Abdomen, Pelvis & other known or	X	Radiologic measurements should be performed every 8 weeks													X			

	Pre-Study	Wk 1	Wk 2	Wk 3	Wk 4	Wk 5	Wk 6	Wk 7	Wk 8	Wk 9	Wk 10	Wk 11[h]	Wk 12	Wk 13	Treatment Discontinuation Visit[i]	28 Days from Last Dose of Nivolumab and Ipilimumab	30 Days from End of Treatment Follow-up	100 Days from Last Dose of Nivolumab and Ipilimumab Follow-up
suspected sites of disease																		
B-HCG	X[f]			Serum or urine pregnancy test must be performed every 8 weeks												X[f]	X[f]	
HPV testing	X[o]																	
Blood for Correlative Studies[m]	X									X					X			
Tumor Biopsies for Correlative Studies[n]	X									X[l]					X			
Survival status																	X[k]	

A: Nivolumab IV 480 mg IV given every 4 weeks +/- 3 days.
B: Ipilimumab IV 1 mg/kg IV given every 8 weeks +/- 3 days.
*: For patients randoimized to receive ipilimumab in combination with nivolumab.
a: Albumin, alkaline phosphatase, total bilirubin, bicarbonate, BUN, calcium, chloride, creatinine, glucose, LDH, phosphorus, potassium, total protein, SGOT [AST], SGPT [ALT], sodium.
b: Thyroid panel including TSH, Free T3, and Free T4. Patients with TSH > 10.0 U/mL or TSH < 0.5 U/mL must be referred to an endrocrinologist with correction in a range between 0.5-10.0 U/mL prior to treatment initiation.
c: If not tested within past 6 months.
d: Only for patients with HIV infection as documented by the presence of a positive HIV antibody test.
e: Hepatitis B surface antibody, Hepatitis B surface antigen, hepatitis B core antibody.
f: Serum or urine pregnancy test (women of childbearing potential) must be performed with 24 hours prior to start of nivolumab, then every 8weeks. After discontinuation from nivolumab these should be repeated at approximately 30 days and approximately 70 days.
g. Tests/evaluations do not need to be repeated prior to first nivolumab dose if performed within past 7 days.
h. Nivolumab may continue to be given every 4 weeks until any of the criteria outlined in section 5 require it be stopped.
i: Off-treatment evaluation, only for patients who discontinue treatment due to disease progression.
j. All patients will be followed for adverse events for 100 days from last dose of treatment on study via phone call. Patients with ongoing adverse event(s) will continue to be followed via phone call(s) until resolution or stabilization of the adverse event. Patients who discontinue therapy due to unacceptable toxicity but have not demonstrated radiographic disease progression will continue to be followed on schedule every 8 weeks with imaging studies until disease progression is documented.
k. Patients will be followed for survival status every 3 months for 2 years after treatment discontinuation or until death, whichever occurs first. Follow up may be accomplished by clinic visit, medical record review, phone contact or email.
l. 8weeks after initiation of treatment with nivolumab (up to 3 days prior to 3rd dose of nivolumab).
m. Peripheral blood will be collected as described in section 10.3 at screening (any time prior to first dose of treatment), after cycle 1, 8 weeks, and at treatment discontinuation (for patients who progress while on treatment). Blood should not be collected if the hemoglobin concentration is < 10.0 g/dL.
n. Optional, but not required, for participation on study.
o. If not previously performed, DNA in-situ hybridization and/or p16 by IHC testing if sufficient archival tissue is available will be performed at enrolling site.
p. For any patients with a history of CHF or at risk because of underlying cardiovascular disease or exposure to cardiotoxic drugs as clinically indicated.
q. For patients with evidence of CHF, MI, cardiomyopathy, or myositis cardiac evaluation including lab tests and cardiology consultations as clinically indicated including EKG, CPK, troponin, echocardiogram (transthoracic).

	Pre-Study	Wk 1	Wk 2	Wk 3	Wk 4	Wk 5	Wk 6	Wk 7	Wk 8	Wk 9	Wk 10	Wk 11[h]	Wk 12	Wk 13	Treatment Discontinuation Visit[i]	28 Days from Last Dose of Nivolumab and Ipilimumab	30 Days from End of Treatment Follow-up	100 Days from Last Dose of Nivolumab and Ipilimumab Follow-up

12. MEASUREMENT OF EFFECT

12.1 Antitumor Effect – Solid Tumors

For the purposes of this study, patients should be re-evaluated for response every 8 weeks. In addition to a baseline scan, confirmatory scans should also be obtained 8 weeks following initial documentation of objective response.

Response and progression will be evaluated in this study using the new international criteria proposed by the revised Response Evaluation Criteria in Solid Tumors (RECIST) guideline (version 1.1)[120]. Changes in the largest diameter (unidimensional measurement) of the tumor lesions and the shortest diameter in the case of malignant lymph nodes are used in the RECIST criteria.

12.1.1 Definitions

Evaluable for toxicity. All patients will be evaluable for toxicity from the time of their first treatment with nivolumab.

Evaluable for objective response. Only those patients who have measurable disease present at baseline, have received at least one dose of therapy, and have had their disease re-evaluated will be considered evaluable for response. These patients will have their response classified according to the definitions stated below. (Note: Patients who exhibit objective disease progression after the first dose will also be considered evaluable.)

Evaluable Non-Target Disease Response. Patients who have lesions present at baseline that are evaluable but do not meet the definitions of measurable disease, have received at least one dose of therapy, and have had their disease re-evaluated will be considered evaluable for non-target disease. The response assessment is based on the presence, absence, or unequivocal progression of the lesions.

12.1.2 Disease Parameters

Measurable disease. Measurable lesions are defined as those that can be accurately measured in at least one dimension (longest diameter to be recorded) as ≥20 mm (≥2 cm) by chest x-ray or as ≥10 mm (≥1 cm) with CT scan, MRI, or calipers by clinical exam. All tumor measurements must be recorded in millimeters (or decimal fractions of centimeters).

Malignant lymph nodes. To be considered pathologically enlarged and measurable, a lymph node must be ≥15 mm (≥1.5 cm) in short axis when assessed by CT scan (CT scan slice thickness recommended to be no greater than 5 mm [0.5 cm]). At baseline and in follow-up, only the short axis will be measured and followed.

Non-measurable disease. All other lesions (or sites of disease), including small lesions (longest diameter <10 mm [<1 cm] or pathological lymph nodes with ≥10 to <15 mm [≥1

to <1.5 cm] short axis), are considered non-measurable disease. Bone lesions, leptomeningeal disease, ascites, pleural/pericardial effusions, lymphangitis cutis/pulmonitis, inflammatory breast disease, and abdominal masses (not followed by CT or MRI), are considered as non-measurable.

Note: Cystic lesions that meet the criteria for radiographically defined simple cysts should not be considered as malignant lesions (neither measurable nor non-measurable) since they are, by definition, simple cysts.

'Cystic lesions' thought to represent cystic metastases can be considered as measurable lesions, if they meet the definition of measurability described above. However, if non-cystic lesions are present in the same patient, these are preferred for selection as target lesions.

Target lesions. All measurable lesions up to a maximum of 2 lesions per organ and 5 lesions in total, representative of all involved organs, should be identified as **target lesions** and recorded and measured at baseline. Target lesions should be selected on the basis of their size (lesions with the longest diameter), be representative of all involved organs, but in addition should be those that lend themselves to reproducible repeated measurements. It may be the case that, on occasion, the largest lesion does not lend itself to reproducible measurement in which circumstance the next largest lesion which can be measured reproducibly should be selected. A sum of the diameters (longest for non-nodal lesions, short axis for nodal lesions) for all target lesions will be calculated and reported as the baseline sum diameters. If lymph nodes are to be included in the sum, then only the short axis is added into the sum. The baseline sum diameters will be used as reference to further characterize any objective tumor regression in the measurable dimension of the disease.

Non-target lesions. All other lesions (or sites of disease) including any measurable lesions over and above the 5 target lesions should be identified as **non-target lesions** and should also be recorded at baseline. Measurements of these lesions are not required, but the presence, absence, or in rare cases unequivocal progression of each should be noted throughout follow-up.

12.1.3 Methods for Evaluation of Measurable Disease

All measurements should be taken and recorded in metric notation using a ruler or calipers. All baseline evaluations should be performed as closely as possible to the beginning of treatment and never more than 4 weeks before the beginning of the treatment.

The same method of assessment and the same technique should be used to characterize each identified and reported lesion at baseline and during follow-up. Imaging-based evaluation is preferred to evaluation by clinical examination unless the lesion(s) being followed cannot be imaged but are assessable by clinical exam.

Clinical lesions Clinical lesions will only be considered measurable when they are superficial (*e.g.*, skin nodules and palpable lymph nodes) and ≥10 mm (≥1 cm) diameter as assessed using calipers (*e.g.*, skin nodules). In the case of skin lesions, documentation by color photography, including a ruler to estimate the size of the lesion, is recommended.

Chest x-ray Lesions on chest x-ray are acceptable as measurable lesions when they are clearly defined and surrounded by aerated lung. However, CT is preferable.

Conventional CT and MRI This guideline has defined measurability of lesions on CT scan based on the assumption that CT slice thickness is 5 mm (0.5 cm) or less. If CT scans have slice thickness greater than 5 mm (0.5 cm), the minimum size for a measurable lesion should be twice the slice thickness. MRI is also acceptable in certain situations (*e.g.* for body scans).

Use of MRI remains a complex issue. MRI has excellent contrast, spatial, and temporal resolution; however, there are many image acquisition variables involved in MRI, which greatly impact image quality, lesion conspicuity, and measurement. Furthermore, the availability of MRI is variable globally. As with CT, if an MRI is performed, the technical specifications of the scanning sequences used should be optimized for the evaluation of the type and site of disease. Furthermore, as with CT, the modality used at follow-up should be the same as was used at baseline and the lesions should be measured/assessed on the same pulse sequence. It is beyond the scope of the RECIST guidelines to prescribe specific MRI pulse sequence parameters for all scanners, body parts, and diseases. Ideally, the same type of scanner should be used and the image acquisition protocol should be followed as closely as possible to prior scans. Body scans should be performed with breath-hold scanning techniques, if possible.

PET-CT At present, the low dose or attenuation correction CT portion of a combined PET-CT is not always of optimal diagnostic CT quality for use with RECIST measurements. However, if the site can document that the CT performed as part of a PET-CT is of identical diagnostic quality to a diagnostic CT (with IV and oral contrast), then the CT portion of the PET-CT can be used for RECIST measurements and can be used interchangeably with conventional CT in accurately measuring cancer lesions over time. Note, however, that the PET portion of the CT introduces additional data which may bias an investigator if it is not routinely or serially performed.

Ultrasound Ultrasound is not useful in assessment of lesion size and should not be used as a method of measurement. Ultrasound examinations cannot be reproduced in their entirety for independent review at a later date and, because they are operator dependent, it cannot be guaranteed that the same technique and measurements will be taken from one assessment to the next. If new lesions are identified by ultrasound in the course of the study, confirmation by CT or MRI is advised. If there is concern about radiation exposure at CT, MRI may be used instead of CT in selected instances.

Endoscopy, Laparoscopy The utilization of these techniques for objective tumor

evaluation is not advised. However, such techniques may be useful to confirm complete pathological response when biopsies are obtained or to determine relapse in trials where recurrence following complete response (CR) or surgical resection is an endpoint.

Tumor markers Tumor markers alone cannot be used to assess response.

Cytology, Histology These techniques can be used to differentiate between partial responses (PR) and complete responses (CR) in rare cases (*e.g.*, residual lesions in tumor types, such as germ cell tumors, where known residual benign tumors can remain).

The cytological confirmation of the neoplastic origin of any effusion that appears or worsens during treatment when the measurable tumor has met criteria for response or stable disease is mandatory to differentiate between response or stable disease (an effusion may be a side effect of the treatment) and progressive disease.

FDG-PET While FDG-PET response assessments need additional study, it is sometimes reasonable to incorporate the use of FDG-PET scanning to complement CT scanning in assessment of progression (particularly possible 'new' disease). New lesions on the basis of FDG-PET imaging can be identified according to the following algorithm:

a. Negative FDG-PET at baseline, with a positive FDG-PET at follow-up is a sign of PD based on a new lesion.
b. No FDG-PET at baseline and a positive FDG-PET at follow-up: If the positive FDG-PET at follow-up corresponds to a new site of disease confirmed by CT, this is PD. If the positive FDG-PET at follow-up is not confirmed as a new site of disease on CT, additional follow-up CT scans are needed to determine if there is truly progression occurring at that site (if so, the date of PD will be the date of the initial abnormal FDG-PET scan). If the positive FDG-PET at follow-up corresponds to a pre-existing site of disease on CT that is not progressing on the basis of the anatomic images, this is not PD.
c. FDG-PET may be used to upgrade a response to a CR in a manner similar to a biopsy in cases where a residual radiographic abnormality is thought to represent fibrosis or scarring. The use of FDG-PET in this circumstance should be prospectively described in the protocol and supported by disease-specific medical literature for the indication. However, it must be acknowledged that both approaches may lead to false positive CR due to limitations of FDG-PET and biopsy resolution/sensitivity.

Note: A ‘positive’ FDG-PET scan lesion means one which is FDG avid with an uptake greater than twice that of the surrounding tissue on the attenuation corrected image.

12.1.4 Response Criteria:

12.1.4.1 Evaluation of Target Lesions

Complete Response (CR): Disappearance of all target lesions. Any pathological lymph nodes (whether target or non-target) must have reduction in short axis to <10 mm (<1 cm).

Partial Response (PR): At least a 30% decrease in the sum of the diameters of target lesions, taking as reference the baseline sum diameters.

Progressive Disease (PD): At least a 20% increase in the sum of the diameters of target lesions, taking as reference the smallest sum on study (this includes the baseline sum if that is the smallest on study). In addition to the relative increase of 20%, the sum must also demonstrate an absolute increase of at least 5 mm (0.5 cm). (Note: the appearance of one or more new lesions is also considered progressions).

Stable Disease (SD): Neither sufficient shrinkage to qualify for PR nor sufficient increase to qualify for PD, taking as reference the smallest sum diameters while on study.

12.1.4.2 Evaluation of Non-Target Lesions

Complete Response (CR): Disappearance of all non-target lesions and normalization of tumor marker level. All lymph nodes must be non-pathological in size (<10 mm [<1 cm] short axis).

Non-CR/Non-PD: Persistence of one or more non-target lesion(s) and/or maintenance of tumor marker level above the normal limits.

Progressive Disease (PD): Appearance of one or more new lesions and/or *unequivocal progression* of existing non-target lesions. *Unequivocal progression* should not normally trump target lesion status. It must be representative of overall disease status change, not a single lesion increase.

Although a clear progression of “non-target” lesions only is exceptional, the opinion of the treating physician should prevail in such circumstances, and the progression status should be confirmed at a later time by the review panel (or Principal Investigator).

12.1.4.3 Evaluation of Best Overall Response

The best overall response is the best response recorded from the start of the treatment until disease progression/recurrence (taking as reference for progressive disease the smallest measurements recorded since the treatment started). The patient's best response assignment will depend on the achievement of both measurement and confirmation criteria.

Responses will be assessed using CT scans or magnetic resonance imaging according to standard RECIST 1.1 criteria in order to assess disease progression. These criteria will also allow for patients who experience an initial disease flare, and as some patients who will have a delayed response may experience an initial disease flare, we will allow patients with radiographic disease progression to continue on the trial

provided that they have a stable ECOG performance status, no need for immediate alternative treatment, and progression of no more than 40% with three or fewer new lesions. If, at the next evaluation, these patients demonstrate further disease progression, then they will be labeled as non-responders to therapy. However, if they do meet the criteria for response according to RECIST 1.1, then they will be deemed as a responder and counted accordingly in the tabulations for determining whether or not to proceed to a second stage or achieving the study end point.

For Patients with Measurable Disease (*i.e.*, Target Disease)

<table>
<tr><th>Target Lesions</th><th>Non-Target Lesions</th><th>New Lesions</th><th>Overall Response</th><th>Best Overall Response when Confirmation is Required*</th></tr>
<tr><td>CR</td><td>CR</td><td>No</td><td>CR</td><td>≥4 wks. Confirmation**</td></tr>
<tr><td>CR</td><td>Non-CR/Non-PD</td><td>No</td><td>PR</td><td rowspan="3">≥4 wks. Confirmation**</td></tr>
<tr><td>CR</td><td>Not evaluated</td><td>No</td><td>PR</td></tr>
<tr><td>PR</td><td>Non-CR/Non-PD/not evaluated</td><td>No</td><td>PR</td></tr>
<tr><td>SD</td><td>Non-CR/Non-PD/not evaluated</td><td>No</td><td>SD</td><td>Documented at least once ≥4 wks. from baseline**</td></tr>
<tr><td>PD</td><td>Any</td><td>Yes or No</td><td>PD</td><td rowspan="3">no prior SD, PR or CR</td></tr>
<tr><td>Any</td><td>PD***</td><td>Yes or No</td><td>PD</td></tr>
<tr><td>Any</td><td>Any</td><td>Yes</td><td>PD</td></tr>
<tr><td colspan="5">* See RECIST 1.1 manuscript for further details on what is evidence of a new lesion.
** Only for non-randomized trials with response as primary endpoint.
*** In exceptional circumstances, unequivocal progression in non-target lesions may be accepted as disease progression.

<u>Note</u>: Patients with a global deterioration of health status requiring discontinuation of treatment without objective evidence of disease progression at that time should be reported as "symptomatic deterioration." Every effort should be made to document the objective progression even after discontinuation of treatment.</td></tr>
</table>

For Patients with Non-Measurable Disease (*i.e.*, Non-Target Disease)

<table>
<tr><th>Non-Target Lesions</th><th>New Lesions</th><th>Overall Response</th></tr>
<tr><td>CR</td><td>No</td><td>CR</td></tr>
<tr><td>Non-CR/non-PD</td><td>No</td><td>Non-CR/non-PD*</td></tr>
<tr><td>Not all evaluated</td><td>No</td><td>not evaluated</td></tr>
<tr><td>Unequivocal PD</td><td>Yes or No</td><td>PD</td></tr>
<tr><td>Any</td><td>Yes</td><td>PD</td></tr>
<tr><td colspan="3">* 'Non-CR/non-PD' is preferred over 'stable disease' for non-target disease since SD is increasingly used as an endpoint for assessment of efficacy in some trials so to assign this category when no lesions can be measured is not advised</td></tr>
</table>

12.1.5 Duration of Response

Duration of overall response: The duration of overall response is measured from the time measurement criteria are met for CR or PR (whichever is first recorded) until the first date that recurrent or progressive disease is objectively documented (taking as reference for progressive disease the smallest measurements recorded since the treatment started).

The duration of overall CR is measured from the time measurement criteria are first met for CR until the first date that progressive disease is objectively documented. [120]

Duration of stable disease: Stable disease is measured from the start of the treatment until the criteria for progression are met, taking as reference the smallest measurements recorded since the treatment started, including the baseline measurements.

12.1.6 Progression-Free Survival

Part A: PFS is defined as the duration of time from start of treatment to time of progression or death, whichever occurs first.

Part B: PFS is defined as the time period from the date of randomization to the date of PD or death whichever occurred first.

12.1.7 Response Review

All responses will be reviewed by an expert independent of the study at the study's completion. Simultaneous review of the patients' files and radiological images is the best approach.

12.2 Other Response Parameters

12.2.1 Overall Survival (OS)

Part A: OS is defined as the duration of time from start of treatment to time of death.
Part B: OS will be measured from the time of randomization to the time of death.

13. STUDY OVERSIGHT AND DATA REPORTING / REGULATORY REQUIREMENTS

Adverse event lists, guidelines, and instructions for AE reporting can be found in Section 7.0 (Adverse Events: List and Reporting Requirements).

13.1 Study Oversight

This protocol is monitored at several levels, as described in this section. The Protocol Principal Investigator is responsible for monitoring the conduct and progress of the clinical trial, including the ongoing review of accrual, patient-specific clinical and laboratory data, and routine and

serious adverse events; reporting of expedited adverse events; and accumulation of reported adverse events from other trials testing the same drug(s). The Protocol Principal Investigator and statistician have access to the data at all times through the CTMS web-based reporting portal.

During the Phase 2 portion of the study, the Protocol Principal Investigator will have, at a minimum, quarterly conference calls with the Study Investigators and the CTEP Medical Officer(s) to review accrual, progress, and pharmacovigilance. Decisions to proceed to the second stage of a Phase 2 trial will require sign-off by the Protocol Principal Investigator and the Protocol Statistician.

All Study Investigators at participating sites who register/enroll patients on a given protocol are responsible for timely submission of data via Medidata Rave and timely reporting of adverse events for that particular study. This includes timely review of data collected on the electronic CRFs submitted via Medidata Rave.

All studies are also reviewed in accordance with the enrolling institution's data safety monitoring plan.

13.2 Data Reporting

Data collection for this study will be done exclusively through Medidata Rave. Access to the trial in Rave is granted through the iMedidata application to all persons with the appropriate roles assigned in the Regulatory Support System (RSS). To access Rave via iMedidata, the site user must have an active CTEP IAM account (check at < https://ctepcore.nci.nih.gov/iam >) and the appropriate Rave role (Rave CRA, Read-Only, RAVE CRA, Lab Admin, SLA or Site Investigator) on either the LPO or participating organization roster at the enrolling site. To the hold Rave CRA role or RAVE CRA (Lab Admin) role, the user must hold a minimum of an AP registration type. To hold the Rave Site Investigator role, the individual must be registered as an NPIVR or IVR. Associates can hold read-only roles in Rave.

Upon initial site registration approval for the study in RSS, all persons with Rave roles assigned on the appropriate roster will be sent a study invitation e-mail from iMedidata. To accept the invitation, site users must log into the Select Login (https://login.imedidata.com/selectlogin) using their CTEP-IAM user name and password, and click on the "accept" link in the upper right-corner of the iMedidata page. Please note, site users will not be able to access the study in Rave until all required Medidata and study specific trainings are completed. Trainings will be in the form of electronic learnings (eLearnings), and can be accessed by clicking on the link in the upper right pane of the iMedidata screen.

Users that have not previously activated their iMedidata/Rave account at the time of initial site registration approval for the study in RSS will also receive a separate invitation from iMedidata to activate their account. Account activation instructions are located on the CTSU website, Rave tab under the Rave resource materials (Medidata Account Activation and Study Invitation Acceptance). Additional information on iMedidata/Rave is available on the CTSU members' website under the Rave tab or by contacting the CTSU Help Desk at 1-888-823-5923 or by e-mail at ctsucontact@westat.com.

13.2.1 Method

CTMS Routine Monitoring: This study will be monitored by the Clinical Trials Monitoring Service (CTMS). Data is to be submitted to CTMS at least once every two weeks via Medidata Rave (or other modality if approved by CTEP). Information on CTMS reporting is available at: http://www.theradex.com/clinicalTechnologies/?National-Cancer-Institute-NCI-11. On-site audits will be conducted on a 18-36 month basis as part of routine cancer center site visits. More frequent audits may be conducted if warranted by accrual or due to concerns regarding data quality or timely submission. For CTMS monitored studies, after users have activated their accounts, please contact the Theradex Help Desk at (609) 799-7580 or by email at CTMSSupport@theradex.com for additional support with Rave and completion of CRFs.

13.2.2 Responsibility for Data Submission

For ETCTN trials, it is the responsibility of the PI(s) at the site to ensure that all investigators at the ETCTN Sites understand the procedures for data submission for each ETCTN protocol and that protocol specified data are submitted accurately and in a timely manner to the CTMS via the electronic data capture system, Medidata Rave.

Data are to be submitted via Medidata Rave to CTMS on a real-time basis, but no less than once every 2 weeks. The timeliness of data submissions and timeliness in resolving data queries will be tracked by CTMS. Metrics for timeliness will be followed and assessed on a quarterly basis. For the purpose of Institutional Performance Monitoring, data will be considered delinquent if it is greater than 4 weeks past due.

Data from Medidata Rave and CTEP-AERS is reviewed by the CTMS on an ongoing basis as data is received. Queries will be issued by CTMS directly within Rave. The queries will appear on the Task Summary Tab within Rave for the CRA at the ETCTN to resolve. Monthly web-based reports are posted for review by the Drug Monitors in the IDB, CTEP. Onsite audits will be conducted by the CTMS to ensure compliance with regulatory requirements, GCP, and NCI policies and procedures with the overarching goal of ensuring the integrity of data generated from NCI-sponsored clinical trials, as described in the ETCTN Program Guidelines, which may be found on the CTEP (http://ctep.cancer.gov/protocolDevelopment/electronic_applications/adverse_events.htm) and CTSU websites.

An End of Study CRF is to be completed by the PI, and is to include a summary of study endpoints not otherwise captured in the database, such as (for phase 1 trials) the recommended phase 2 dose (RP2D), and a description of any dose-limiting toxicities (DLTs). CTMS will utilize a core set of eCRFs that are Cancer Data Standards Registry and Repository (caDSR) compliant (http://cbiit.nci.nih.gov/ncip/biomedical-informatics-resources/interoperability-and-semantics/metadata-and-models). Customized eCRFs will be included when appropriate to meet unique study requirements. The PI is encouraged

to review the eCRFs, working closely with CTMS to ensure prospectively that all required items are appropriately captured in the eCRFs prior to study activation. CTMS will prepare the eCRFs with built-in edit checks to the extent possible to promote data integrity.

CDUS data submissions for ETCTN trials activated after March 1, 2014, will be carried out by the CTMS contractor, Theradex. CDUS submissions are performed by Theradex on a monthly basis. The trial's lead institution is responsible for timely submission to CTMS via Rave, as above.

Further information on data submission procedures can be found in the ETCTN Program Guidelines (http://ctep.cancer.gov/protocolDevelopment/electronic_applications/adverse_events.htm).

See Section 12.1.1 for details on CDUS reporting. As the data management center for this trial, Theradex is responsible for compiling and submitting CDUS data to CTEP for all participants and for providing the data to the Principal Investigator for review.

13.3 CTEP Multicenter Guidelines

N/A

13.4 Collaborative Agreements Language

The agent supplied by CTEP, DCTD, NCI used in this protocol is/are provided to the NCI under a Collaborative Agreement (CRADA, CTA, CSA) between the Pharmaceutical Company (hereinafter referred to as "Collaborator(s)") and the NCI Division of Cancer Treatment and Diagnosis. Therefore, the following obligations/guidelines, in addition to the provisions in the "Intellectual Property Option to Collaborator" (http://ctep.cancer.gov/industryCollaborations2/intellectual_property.htm) contained within the terms of award, apply to the use of the Agent(s) in this study:

1. Agent(s) may not be used for any purpose outside the scope of this protocol, nor can Agent(s) be transferred or licensed to any party not participating in the clinical study. Collaborator(s) data for Agent(s) are confidential and proprietary to Collaborator(s) and shall be maintained as such by the investigators. The protocol documents for studies utilizing Agents contain confidential information and should not be shared or distributed without the permission of the NCI. If a copy of this protocol is requested by a patient or patient's family member participating on the study, the individual should sign a confidentiality agreement. A suitable model agreement can be downloaded from: http://ctep.cancer.gov.

2. For a clinical protocol where there is an investigational Agent used in combination with (an)other Agent(s), each the subject of different Collaborative Agreements, the access to and use of data by each Collaborator shall be as follows (data pertaining to such combination use shall hereinafter be referred to as "Multi-Party Data"):

a. NCI will provide all Collaborators with prior written notice regarding the existence and nature of any agreements governing their collaboration with NCI, the design of the proposed combination protocol, and the existence of any obligations that would tend to restrict NCI's participation in the proposed combination protocol.

b. Each Collaborator shall agree to permit use of the Multi-Party Data from the clinical trial by any other Collaborator solely to the extent necessary to allow said other Collaborator to develop, obtain regulatory approval or commercialize its own Agent.

c. Any Collaborator having the right to use the Multi-Party Data from these trials must agree in writing prior to the commencement of the trials that it will use the Multi-Party Data solely for development, regulatory approval, and commercialization of its own Agent.

3. Clinical Trial Data and Results and Raw Data developed under a Collaborative Agreement will be made available to Collaborator(s), the NCI, and the FDA, as appropriate and unless additional disclosure is required by law or court order as described in the IP Option to Collaborator (http://ctep.cancer.gov/industryCollaborations2/intellectual_property.htm). Additionally, all Clinical Data and Results and Raw Data will be collected, used and disclosed consistent with all applicable federal statutes and regulations for the protection of human subjects, including, if applicable, the *Standards for Privacy of Individually Identifiable Health Information* set forth in 45 C.F.R. Part 164.

4. When a Collaborator wishes to initiate a data request, the request should first be sent to the NCI, who will then notify the appropriate investigators (Group Chair for Cooperative Group studies, or PI for other studies) of Collaborator's wish to contact them.

5. Any data provided to Collaborator(s) for Phase 3 studies must be in accordance with the guidelines and policies of the responsible Data Monitoring Committee (DMC), if there is a DMC for this clinical trial.

6. Any manuscripts reporting the results of this clinical trial must be provided to CTEP by the Group office for Cooperative Group studies or by the principal investigator for non-Cooperative Group studies for immediate delivery to Collaborator(s) for advisory review and comment prior to submission for publication. Collaborator(s) will have 30 days from the date of receipt for review. Collaborator shall have the right to request that publication be delayed for up to an additional 30 days in order to ensure that Collaborator's confidential and proprietary data, in addition to Collaborator(s)'s intellectual property rights, are protected. Copies of abstracts must be provided to CTEP for forwarding to Collaborator(s) for courtesy review as soon as possible and preferably at least three (3) days prior to submission, but in any case, prior to presentation at the meeting or publication in the proceedings. Press releases and other media presentations must also be forwarded to CTEP prior to release. Copies of any manuscript, abstract and/or press release/ media presentation should be sent to:

Email: ncicteppubs@mail.nih.gov

The Regulatory Affairs Branch will then distribute them to Collaborator(s). No publication, manuscript or other form of public disclosure shall contain any of Collaborator's confidential/ proprietary information.

14. STATISTICAL CONSIDERATIONS

14.1 Study Design/Endpoints (Part A)

14.1.1 Primary Endpoint

Overall response rate is the primary endpoint for this study.

14.1.2 Study Design

This study is a two-staged, Simon Optimal phase II clinical trial of nivolumab as a single agent for the treatment of patients with locally advanced/unresectable or metastatic squamous cell carcinoma of the anal canal who have progressed through at least one prior line of therapy. Responses will be assessed using CT scans or magnetic resonance imaging according to standard RECIST 1.1 criteria in order to assess disease progression. These criteria will also allow for patients who experience an initial disease flare, and as some patients who will have a delayed response may experience an initial disease flare, we will allow patients with radiographic disease progression to continue on the trial provided that they have a stable ECOG performance status, no need for immediate alternative treatment, and progression of no more than 40% with three or fewer new lesions. If, at the next evaluation, these patients demonstrate further disease progression, then they will be labeled as non-responders to therapy. However, if they do meet the criteria for response according to RECIST 1.1, then they will be deemed as a responder and counted accordingly in the tabulations for determining whether or not to proceed to a second stage or achieving the study end point. If no response is observed among the 12 patients treated in the first stage at six months, then the trial will be stopped for futility. However, if one or more responses are observed, then the trial will be expanded to include an additional 25 patients, with 4 or more responses needed among the 37 total participants to declare an efficacy of this single-agent therapy.

14.1.3 Null/Alternative Hypotheses

For this Simon Optimal, two-stage phase II study, we propose a null hypothesis H_o: $p \leq 0.05$ and an alternative hypothesis H_a: $p \geq 0.20$, where $\underline{p}$ represents the percentage of patients with metastatic SCCA of the anal canal demonstrating either a partial response or a complete response according to RECIST 1.1 criteria when treated with nivolumab. If the null hypothesis is rejected and the alternative hypothesis is accepted based on the results of this study, then a phase III trial comparing nivolumab to best supportive care in patients with refractory metastatic squamous cell carcinoma of the anal canal will be proposed.

14.2 Sample Size/Accrual Rate

Using an $\alpha = 0.10$ and a $\beta = 0.10$, we estimate that at least twelve patients, and no more than 37 patients, will be enrolled onto this trial.

PLANNED ENROLLMENT REPORT (Part A)

Racial Categories	Ethnic Categories				Total
	Not Hispanic or Latino		Hispanic or Latino		
	Female	Male	Female	Male	
American Indian/ Alaska Native	0	0	0	0	0
Asian	2	0	0	0	2
Native Hawaiian or Other Pacific Islander	0	0	0	0	0
Black or African American	3	1	0	0	4
White	20	7	3	1	31
	0	0	0	0	0
**Total**	25	8	3	1	37

PHS 398 / PHS 2590 (Rev. 08/12 Approved Through 8/31/2015) OMB No. 0925-0001/0002

14.3 Stratification Factors

N/A

14.4 Analysis of Secondary Endpoints

Progression-free survival

Kaplan-Meier analysis will be performed to estimate the median progression-free survival with a 90% confidence interval. Progression-free survival will be calculated as the time from initiation of treatment with nivolumab until the time of disease progression according to RECIST version 1.1 criteria.

Overall survival

Kaplan-Meier analysis will be performed to estimate the median overall survival with a 90% confidence interval. Overall survival will be calculated as the time from initiation of treatment with nivolumab until death.

Toxicity

Adverse events will be monitored and recorded according to grade for each patient from the time of treatment initiation until 30 days (±7 days) after radiographic progression and end of treatment via phone call or visit. The frequency of each serious adverse event will be measured relative to the total number of patients treated. Toxicities will be tabulated by type and grade. All patients will be followed for adverse events for 100 days from last dose of treatment on study via phone call. Patients with ongoing adverse event(s) will continue to be followed via phone call(s) until resolution or stabilization of the adverse event.

14.5 Analyses of Exploratory Endpoints:

14.5.1 Descriptive Statistics of IHC and Blood Sample Studies.

Descriptive statistics including plots, mean, median and standard deviations will be used to summarize data. For continuous outcomes, t-test and ANOVA will be used to compare outcome measures across patient characteristics. Dunnett's and Tukey's test that properly adjust for multiplicity in multiple tests will be implemented. Pair-wise comparisons will be performed using pre- and post-therapy samples from each patient . The chi-square (c2) test or Fisher's exact test will be used to test the association between two categorical variables such as disease state and performance status. Both univariate and multivariate logistic regressions will be performed to model prognostic factors.

14.6 Study Design/Endpoints (Part B)

The primary endpoint of part B is progression-free survival (PFS). The event is progression of disease or death, whichever occurs first. The PFS time is defined as the time period from the date of randomization to the date of PD or death whichever occurred first. Patients who are alive without PD will be censored, and their PFS time is defined as the time period from the date of randomization to the date of last follow-up.

14.6.1 Study Design

This is a randomized phase II trial. A group sequential design with one interim analysis for futility will be implemented for this randomized study. Sample size calculation is based on comparison of PFS between the two treatment arms with the following assumptions:

- 1:1 randomization Median PFS of 4 months for nivolumab only arm
- Median PFS of 7 months for nivolumab + ipilimumab arm

- An interim analysis of PFS at 50% of the events with O'Brien-Fleming boundary

14.6.2 Null/alternative hypothesis

The null hypothesis is that there is no significant difference in distribution of PFS between the two treatment arms. It is expected that the median PFS time for nivolumab arm is 4 months. The alternative hypothesis is that combination of nivolumab + IPI can improve PFS compared with nivolumab. It would be encouraging that if the combination treatment can achieve a median PFS time of 7 months.

14.6.3 Sample size and accrual rate

With the above assumption, a total of 87 events will have 90% power to detect a difference of 4 months vs. 7 months in median PFS between treatment arms at a one-sided significance level of 0.1. The study will randomize 100 patients to obtain the 87 events. Assuming an accrual rate of 5 patients per month, the accrual time will be 20 months and maximum study duration will be about 28.2 months. Thus, the additional follow up time will be 8.2 months after the last patient have been enrolled. The primary endpoint will be assessed when at least 8.2 months of followup after the last patient has been reached or when the study has observed 87 progression events, whichever occurs sooner.

PLANNED ENROLLMENT REPORT (Part B)

Racial Categories	Ethnic Categories				Total
	Not Hispanic or Latino		Hispanic or Latino		
	Female	Male	Female	Male	
American Indian/ Alaska Native	0	0	0	0	**0**
Asian	5	2	0	0	**7**
Native Hawaiian or Other Pacific Islander	1	0	0	0	**1**
Black or African American	7	3	0	0	**10**
White	52	19	8	3	**82**
	0	0	0	0	**0**
**Total**	**65**	**24**	**7**	**3**	**100**

PHS 398 / PHS 2590 (Rev. 08/12 Approved Through 8/31/2015) OMB No. 0925-0001/0002

An interim analysis will be performed when 50% of events (i.e. 44 events) occurs. If the p value ≥ 0.586 the study will stopped early for futility corresponding to a boundary for the HR being 1.068.

A. Definition of analysis populations:

The safety analysis set consists of all subjects who take at least one dose of study drug. The safety analysis set will be used for safety analyses. The full analysis set consists of all randomized subjects. The full analysis set will be used for efficacy analyses.

14.6.4 Analysis of secondary endpoints

Secondary endpoints for part B include overall response rates, overall survival, and grade 3/4/5 adverse events. These endpoints will be analyzed as described in part A.

14.7 Reporting and Exclusions

14.7.1 Evaluation of Toxicity

All patients will be evaluable for toxicity from the time of their first treatment with nivolumab.

14.7.2 Evaluation of Response

All eligible patients included in the study must be assessed for response to treatment, even if there are major protocol treatment deviations. Each patient will be assigned one of the following categories: 1) complete response, 2) partial response, 3) stable disease, 4) progressive disease, 5) early death from malignant disease, 6) early death from toxicity, 7) early death because of other cause, or 9) unknown (not assessable, insufficient data). [Note: By arbitrary convention, category 9 usually designates the “unknown” status of any type of data in a clinical database.]

All of the patients who met the eligibility criteria (with the possible exception of those who received no study medication) should be included in the main analysis of the response rate. Patients in response categories 4-9 should be considered to have a treatment failure (disease progression). Thus, an incorrect treatment schedule or drug administration does not result in exclusion from the analysis of the response rate. Precise definitions for categories 4-9 will be protocol specific.

All conclusions should be based on all eligible patients. Subanalyses may then be performed on the basis of a subset of patients, excluding those for whom major protocol deviations have been identified (*e.g.*, early death due to other reasons, early discontinuation of treatment, major protocol violations, etc.). However, these subanalyses may not serve as the basis for drawing conclusions concerning treatment

efficacy, and the reasons for excluding patients from the analysis should be clearly reported. The overall response (CR and PR) rate along with the 95% confidence interval will be estimated.

15. STUDY STATUS UPDATES AND STUDY CLOSURE

15.1 Definitions of Study Status Changes

15.1.1 Temporarily Closed to Accrual

The study status is Temporarily Closed to Accrual when no patient slots are currently available, but there is the possibility that the trial will re-open for accrual (patient slots become available). Sites are not permitted to accrue additional patients until CTEP is notified of Re-Activation.

Study status will need to be changed to Temporarily Closed to Accrual when any of the following criteria are met:

- Sites are notified by CTEP (via Request for Rapid Amendment [RRA]) of changes in the risk/benefit ratio that necessitate changes to the patient Informed Consent document. Requested changes will be specified in the RRA and must be reviewed by the study's IRB.
- CTEP and the lead investigator agree that unacceptable toxicities necessitate a discussion to change the dosing/regimen.
- A protocol-defined benchmark has been achieved (such as an interim analysis before proceeding to the next stage).

15.1.2 Closed to Accrual

The study status is (permanently) Closed to Accrual when no more patient enrollment slots are available, and at least one patient is still actively receiving the study treatment. Sites are no longer permitted to enroll additional patients.

Patient slots are no longer available when the following criteria are met:

- The pre-specified number of evaluable patients has been successfully enrolled, treated, and evaluated.
- The study treatment has failed to meet the pre-specified efficacy goal at the stage 1 interim analysis.
- CTEP and the investigators agree that unacceptable toxicities preclude further enrollment.

15.1.3 Closed to Accrual and Treatment

The study status is Closed to Accrual and Treatment when no more patient enrollment slots are available <u>and</u> no patients are currently receiving the study treatment. Patients

may still be enrolled on the protocol only for the purposes of follow-up.

Patient accrual and treatment will be permanently halted when any of the following criteria are met:

- Enrollment was previously closed (study status of "Closed to Accrual"), and no patients are receiving the study treatment.
- CTEP and the investigators agree that unacceptable toxicities preclude further enrollment. In this case, CTEP and the investigators must collaborate to alter the regimen or to halt the study treatment altogether as soon as it can be safely done for patients currently receiving treatment.

CTEP and Theradex **must be notified** when patients are no longer receiving treatment [*i.e.*, when the last patient(s) to be receiving treatment is/are no longer receiving the study regimen for any reason].

15.1.4 Closed to Follow-Up

The study is considered Closed to Follow-Up when all protocol-defined follow-up procedures have been completed for all patients who have not been removed from the study for other reasons. That is, there are no outstanding follow-up procedures to be performed as mandated by the protocol.

CTEP does **not** need to be notified of a status change to "Closed to Follow Up."

15.1.5 Complete

Study is considered Complete if it has been at least thirty (30) days since the last patient follow-up evaluation.

A citation to a final study report (manuscript, meeting abstract, etc.) is required with the submission of the Protocol Status Update Form to CTEP PIO.

15.2 Responsibility for Filing Protocol Status Update Forms

CTEP must be notified of all study status changes in Section 14.1 (except for Closed to Follow-Up) by the Corresponding Organization via Protocol Status Update Form, available from the CTEP website at http://ctep.cancer.gov/protocolDevelopment/default.htm#amendments.

Theradex must be notified as soon as all patients are off treatment (*i.e.*, when study status changes to Closed to Accrual and Treatment). Theradex will produce a report within 90 days of this notification.

REFERENCES

1. Siegel R, Naishadham D, Jemal A. Cancer statistics for Hispanics/Latinos, 2012. *CA Cancer J Clin.* 2012;62(5):283-298.
2. Johnson LG, Madeleine MM, Newcomer LM, Schwartz SM, Daling JR. Anal cancer incidence and survival: the surveillance, epidemiology, and end results experience, 1973-2000. *Cancer.* 2004;101(2):281-288.
3. Siegel RL, Miller KD, Jemal A. Cancer statistics, 2017. *CA: a cancer journal for clinicians.* 2017;67(1):7-30.
4. Bartelink H, Roelofsen F, Eschwege F, et al. Concomitant radiotherapy and chemotherapy is superior to radiotherapy alone in the treatment of locally advanced anal cancer: results of a phase III randomized trial of the European Organization for Research and Treatment of Cancer Radiotherapy and Gastrointestinal Cooperative Groups. *J Clin Oncol.* 1997;15(5):2040-2049.
5. Flam M, John M, Pajak TF, et al. Role of mitomycin in combination with fluorouracil and radiotherapy, and of salvage chemoradiation in the definitive nonsurgical treatment of epidermoid carcinoma of the anal canal: results of a phase III randomized intergroup study. *J Clin Oncol.* 1996;14(9):2527-2539.
6. Schiller DE, Cummings BJ, Rai S, et al. Outcomes of salvage surgery for squamous cell carcinoma of the anal canal. *Ann Surg Oncol.* 2007;14(10):2780-2789.
7. Das P, Bhatia S, Eng C, et al. Predictors and patterns of recurrence after definitive chemoradiation for anal cancer. *Int J Radiat Oncol Biol Phys.* 2007;68(3):794-800.
8. Mullen JT, Rodriguez-Bigas MA, Chang GJ, et al. Results of surgical salvage after failed chemoradiation therapy for epidermoid carcinoma of the anal canal. *Ann Surg Oncol.* 2007;14(2):478-483.
9. Eng C. Anal cancer: current and future methodology. *Cancer Invest.* 2006;24(5):535-544.
10. Cummings BJ. Metastatic anal cancer: the search for cure. *Onkologie.* 2006;29(1-2):5-6.
11. Eng C, Rogers J, Chang GJ, et al. Choice of chemotherapy in the treatment of metastatic squamous cell carcinoma of th anal canal. Paper presented at: ASCO Annual Meeting2012; Chicago, Illinois.
12. Jaiyesimi IA, Pazdur R. Cisplatin and 5-fluorouracil as salvage therapy for recurrent metastatic squamous cell carcinoma of the anal canal. *Am J Clin Oncol.* 1993;16(6):536-540.
13. Evans TR, Mansi JL, Glees JP. Response of metastatic anal carcinoma to single agent carboplatin. *Clin Oncol (R Coll Radiol).* 1993;5(1):57-58.
14. Fisher WB, Herbst KD, Sims JE, Critchfield CF. Metastatic cloacogenic carcinoma of the anus: sequential responses to adriamycin and cis-dichlorodiammineplatinum(II). *Cancer Treat Rep.* 1978;62(1):91-97.
15. Eng C, Pathak P. Treatment options in metastatic squamous cell carcinoma of the anal canal. *Curr Treat Options Oncol.* 2008;9(4-6):400-407.
16. Sarkar FH, Miles BJ, Plieth DH, Crissman JD. Detection of human papillomavirus in squamous neoplasm of the penis. *J Urol.* 1992;147(2):389-392.
17. Wolchok JD, Kluger H, Callahan MK, et al. Nivolumab plus ipilimumab in advanced melanoma. *N Engl J Med.* 2013;369(2):122-133.
18. Woo SR, Turnis ME, Goldberg MV, et al. Immune inhibitory molecules LAG-3 and PD-1 synergistically regulate T-cell function to promote tumoral immune escape. *Cancer*

Res. 2012;72(4):917-927.
19. Ryan DP, Compton CC, Mayer RJ. Carcinoma of the anal canal. *N Engl J Med.* 2000;342(11):792-800.
20. Thompson RH, Gillett MD, Cheville JC, et al. Costimulatory B7-H1 in renal cell carcinoma patients: Indicator of tumor aggressiveness and potential therapeutic target. *Proc Natl Acad Sci U S A.* 2004;101(49):17174-17179.
21. Thompson RH, Gillett MD, Cheville JC, et al. Costimulatory molecule B7-H1 in primary and metastatic clear cell renal cell carcinoma. *Cancer.* 2005;104(10):2084-2091.
22. Thompson RH, Kuntz SM, Leibovich BC, et al. Tumor B7-H1 is associated with poor prognosis in renal cell carcinoma patients with long-term follow-up. *Cancer Res.* 2006;66(7):3381-3385.
23. Daling JR, Madeleine MM, Johnson LG, et al. Human papillomavirus, smoking, and sexual practices in the etiology of anal cancer. *Cancer.* 2004;101(2):270-280.
24. Wu C, Zhu Y, Jiang J, Zhao J, Zhang XG, Xu N. Immunohistochemical localization of programmed death-1 ligand-1 (PD-L1) in gastric carcinoma and its clinical significance. *Acta Histochem.* 2006;108(1):19-24.
25. Dong H, Chen L. B7-H1 pathway and its role in the evasion of tumor immunity. *J Mol Med (Berl).* 2003;81(5):281-287.
26. Nomi T, Sho M, Akahori T, et al. Clinical significance and therapeutic potential of the programmed death-1 ligand/programmed death-1 pathway in human pancreatic cancer. *Clin Cancer Res.* 2007;13(7):2151-2157.
27. Zitvogel L, Tesniere A, Kroemer G. Cancer despite immunosurveillance: immunoselection and immunosubversion. *Nat Rev Immunol.* 2006;6(10):715-727.
28. Sunesen KG, Norgaard M, Thorlacius-Ussing O, Laurberg S. Immunosuppressive disorders and risk of anal squamous cell carcinoma: a nationwide cohort study in Denmark, 1978-2005. *Int J Cancer.* 2010;127(3):675-684.
29. Hamanishi J, Mandai M, Iwasaki M, et al. Programmed cell death 1 ligand 1 and tumor-infiltrating CD8+ T lymphocytes are prognostic factors of human ovarian cancer. *Proc Natl Acad Sci U S A.* 2007;104(9):3360-3365.
30. Sturgis EM, Cinciripini PM. Trends in head and neck cancer incidence in relation to smoking prevalence: an emerging epidemic of human papillomavirus-associated cancers? *Cancer.* 2007;110(7):1429-1435.
31. Rizvi NA, Hellmann MD, Brahmer JR, et al. Nivolumab in Combination With Platinum-Based Doublet Chemotherapy for First-Line Treatment of Advanced Non-Small-Cell Lung Cancer. *J Clin Oncol.* 2016;34(25):2969-2979.
32. Topalian SL, Hodi FS, Brahmer JR, et al. Safety, activity, and immune correlates of anti-PD-1 antibody in cancer. *N Engl J Med.* 2012;366(26):2443-2454.
33. Thompson CB, Allison JP. The emerging role of CTLA-4 as an immune attenuator. *Immunity.* 1997;7(4):445-450.
34. Kuhns MS, Epshteyn V, Sobel RA, Allison JP. Cytotoxic T lymphocyte antigen-4 (CTLA-4) regulates the size, reactivity, and function of a primed pool of CD4+ T cells. *Proc Natl Acad Sci USA.* 2000;97(23):12711-12716.
35. Tivol EA, Borriello F, Schweitzer AN, Lynch WP, Bluestone JA, Sharpe AH. Loss of CTLA-4 leads to massive lymphoproliferation and fatal multiorgan tissue destruction, revealing a critical negative regulatory role of CTLA-4. *Immunity.* 1995;3(5):541-547.
36. Waterhouse P, Penninger JM, Timms E, et al. Lymphoproliferative disorders with early

lethality in mice deficient in Ctla-4. *Science.* 1995;270(5238):985-988.

37. Chambers CA, Sullivan TJ, Allison JP. Lymphoproliferation in CTLA-4-deficient mice is mediated by costimulation-dependent activation of CD4+ T cells. *Immunity.* 1997;7(6):885-895.
38. Walunas TL, Lenschow DJ, Bakker CY, et al. CTLA-4 can function as a negative regulator of T cell activation. *Immunity.* 1994;1(5):405-413.
39. Kearney ER, Walunas TL, Karr RW, et al. Antigen-dependent clonal expansion of a trace population of antigen-specific CD4+ T cells in vivo is dependent on CD28 costimulation and inhibited by CTLA-4. *J Immunol.* 1995;155(3):1032-1036.
40. Krummel MF, Allison JP. CD28 and CTLA-4 have opposing effects on the response of T cells to stimulation. *J Exp Med.* 1995;182(2):459-465.
41. Krummel MF, Sullivan TJ, Allison JP. Superantigen responses and co-stimulation: CD28 and CTLA-4 have opposing effects on T cell expansion in vitro and in vivo. *Int Immunol.* 1996;8(4):519-523.
42. Keler T, Halk ED, Vitale L, et al. Activity and Safety of CTLA-4 Blockade Combined with Vaccines in Cynomolgus Macaques. *J Immunol.* 2003;171:6251-6259.
43. Hara I, Nguyen H, Takechi Y, Gansbacher B, Chapman PB, Houghton AN. Rejection of mouse melanoma elicited by local secretion of interleukin-2: implicating macrophages without T cells or natural killer cells in tumor rejection. *Int J Cancer.* 1995;61(2):253-260.
44. Naftzger C, Takechi Y, Kohda H, Hara I, Vijayasaradhi S, Houghton AN. Immune response to a differentiation antigen induced by altered antigen: a study of tumor rejection and autoimmunity. *Proc Natl Acad Sci USA.* 1996;93(25):14809-14814.
45. Bloom MB, Perry-Lalley D, Robbins PF, et al. Identification of Tyrosinase-related Protein 2 as a Tumor Rejection Antigen for the B16 Melanoma. *J Exp Med.* 1997;185(3):453-460.
46. Overwijk WW, Tsung A, Irvine KR, et al. gp100/pmel 17 Is a Murine Tumor Rejection Antigen: Induction of "Self"-reactive, Tumoricidal T Cells Using High-affinity, Altered Peptide Ligand. *JEM.* 1998;188(2):277-286.
47. Weber T, Zemelman BV, McNew JA, et al. SNAREpins: minimal machinery for membrane fusion. *Cell.* 1998;92(6):759-772.
48. Overwijk WW, Lee DS, Surman DR, et al. Vaccination with a recombinant vaccinia virus encoding a "self" antigen induces autoimmune vitiligo and tumor cell destruction in mice: requirement for CD4(+) T lymphocytes. *Proc Natl Acad Sci USA.* 1999;96(6):2982-2987.
49. Rosenberg SA, White DE. Vitiligo in patients with melanoma: normal tissue antigens can be targets for cancer immunotherapy. *J Immunother Emphasis Tumor Immunol.* 1996;19(1):81-84.
50. Hodi FS, O'Day SJ, McDermott DF, et al. Improved survival with ipilimumab in patients with metastatic melanoma. *N Engl J Med.* 2010;363(8):711-723.
51. Yang JC, Hughes M, Kammula U, et al. Ipilimumab (anti-CTLA4 antibody) causes regression of metastatic renal cell cancer associated with enteritis and hypophysitis. *J Immunother.* 2007;30(8):825-830.
52. Hodi FS, Friedlander MB, Atkins DF, et al. A phase I trial of ipilimumab plus bevacizumab in patients with unresectable stage III or stage IV melanoma. *J Clin Oncol.* 2011;29(15_suppl):8511.

53. Carthon BC, Wolchok JD, Yuan J, et al. Preoperative CTLA-4 blockade: tolerability and immune monitoring in the setting of a presurgical clinical trial. *Clin Cancer Res.* 2010;16(10):2861-2871.
54. Liakou CI, Kamat A, Tang DN, et al. CTLA-4 blockade increases IFNgamma-producing CD4+ICOShi cells to shift the ratio of effector to regulatory T cells in cancer patients. *Proc Natl Acad Sci U S A.* 2008;105(39):14987-14992.
55. Royal RE, Levy C, Turner K, et al. Phase 2 trial of single agent Ipilimumab (anti-CTLA-4) for locally advanced or metastatic pancreatic adenocarcinoma. *J Immunother.* 2010;33(8):828-833.
56. O'Mahony D, Morris JC, Quinn C, et al. A pilot study of CTLA-4 blockade after cancer vaccine failure in patients with advanced malignancy. *Clin Cancer Res.* 2007;13(3):958-964.
57. Hodi FS, Butler M, Oble DA, et al. Immunologic and clinical effects of antibody blockade of cytotoxic T lymphocyte-associated antigen 4 in previously vaccinated cancer patients. *Proc Natl Acad Sci USA.* 2008;105(8):3005-3010.
58. Bashey A, Medina B, Corringham S, et al. CTLA4 blockade with ipilimumab to treat relapse of malignancy after allogeneic hematopoietic cell transplantation. *Blood.* 2009;113(7):1581-1588.
59. Zhou J, Bashey A, Zhong R, et al. CTLA-4 Blockade following Relapse of Malignancy after Allogeneic Stem Cell Transplantation Is Associated with T Cell Activation But Not with Increased Levels of T Regulatory Cells. *ASBMT.* 2011;17(5):682-692.
60. Harzstark AL, Fong L, Weinber VK, et al. Final results of a phase I study of CTLA-4 blockade in combination with GM-CSF for metastatic castration resistant prostate cancer (mCRPC). *J Clin Oncol.* 2010;28(15s):abstr 4689.
61. Fong L, Kwek SS, O'Brien S, et al. Potentiating endogenous antitumor immunity to prostate cancer through combination immunotherapy with CTLA4 blockade and GM-CSF. *Cancer Res.* 2009;69(2):609-615.
62. Madan RA, Mohebtash M, Arlen PM, et al. Overall survival (OS) analysis of a phase l trial of a vector-based vaccine (PSA-TRICOM) and ipilimumab (Ipi) in the treatment of metastatic castration-resistant prostate cancer (mCRPC). *J Clin Oncol.* 2010;28(15s):abstr 2550.
63. Stein WD, Gulley JL, Schlom J, et al. Tumor regression and growth rates determined in five intramural NCI prostate cancer trials: the growth rate constant as an indicator of therapeutic efficacy. *Clin Cancer Res.* 2011;17(4):907-917.
64. Ansell SM, Hurvitz SA, Koenig PA, et al. Phase I study of ipilimumab, an anti-CTLA-4 monoclonal antibody, in patients with relapsed and refractory B-cell non-Hodgkin lymphoma. *Clin Cancer Res.* 2009;15(20):6446-6453.
65. Min L, Vaidya A, Becker C. Thyroid autoimmunity and ophthalmopathy related to melanoma biological therapy. *Eur J Endocrinol.* 2011;164(2):303-307.
66. Wolchok JD, Hoos A, O'Day SJ, et al. Guidelines for the evaluation of immune therapy activity in solid tumors: immune-related response criteria. *Clin Cancer Res.* 2009;15(23):7412-7420.
67. Hoos A, Ibrahim R, Korman A, et al. Development of ipilimumab: contribution to a new paradigm for cancer immunotherapy. *Semin Oncol.* 2010;37(5):533-546.
68. Frisch M, Glimelius B, van den Brule AJ, et al. Sexually transmitted infection as a cause of anal cancer. *N Engl J Med.* 1997;337(19):1350-1358.

69. De Vuyst H, Clifford GM, Nascimento MC, Madeleine MM, Franceschi S. Prevalence and type distribution of human papillomavirus in carcinoma and intraepithelial neoplasia of the vulva, vagina and anus: a meta-analysis. *Int J Cancer.* 2009;124(7):1626-1636.
70. Hoots BE, Palefsky JM, Pimenta JM, Smith JS. Human papillomavirus type distribution in anal cancer and anal intraepithelial lesions. *Int J Cancer.* 2009;124(10):2375-2383.
71. Human papillomavirus-associated cancers - United States, 2004-2008. *MMWR Morb Mortal Wkly Rep.* 2012;61:258-261.
72. Koutsky L. Epidemiology of genital human papillomavirus infection. *Am J Med.* 1997;102(5A):3-8.
73. Stanley M. Pathology and epidemiology of HPV infection in females. *Gynecol Oncol.* 2010;117(2 Suppl):S5-10.
74. Werness BA, Levine AJ, Howley PM. Association of human papillomavirus types 16 and 18 E6 proteins with p53. *Science.* 1990;248(4951):76-79.
75. Scheffner M, Werness BA, Huibregtse JM, Levine AJ, Howley PM. The E6 oncoprotein encoded by human papillomavirus types 16 and 18 promotes the degradation of p53. *Cell.* 1990;63(6):1129-1136.
76. Li X, Coffino P. High-risk human papillomavirus E6 protein has two distinct binding sites within p53, of which only one determines degradation. *J Virol.* 1996;70(7):4509-4516.
77. Ruiz S, Santos M, Segrelles C, et al. Unique and overlapping functions of pRb and p107 in the control of proliferation and differentiation in epidermis. *Development.* 2004;131(11):2737-2748.
78. Balsitis SJ, Sage J, Duensing S, Munger K, Jacks T, Lambert PF. Recapitulation of the effects of the human papillomavirus type 16 E7 oncogene on mouse epithelium by somatic Rb deletion and detection of pRb-independent effects of E7 in vivo. *Mol Cell Biol.* 2003;23(24):9094-9103.
79. Knudsen ES, Knudsen KE. Retinoblastoma tumor suppressor: where cancer meets the cell cycle. *Exp Biol Med (Maywood).* 2006;231(7):1271-1281.
80. Kjaer SK, Frederiksen K, Munk C, Iftner T. Long-term absolute risk of cervical intraepithelial neoplasia grade 3 or worse following human papillomavirus infection: role of persistence. *J Natl Cancer Inst.* 2010;102(19):1478-1488.
81. Rodriguez AC, Schiffman M, Herrero R, et al. Longitudinal study of human papillomavirus persistence and cervical intraepithelial neoplasia grade 2/3: critical role of duration of infection. *J Natl Cancer Inst.* 2010;102(5):315-324.
82. Gillison ML, Koch WM, Capone RB, et al. Evidence for a causal association between human papillomavirus and a subset of head and neck cancers. *J Natl Cancer Inst.* 2000;92(9):709-720.
83. D'Souza G, Kreimer AR, Viscidi R, et al. Case-control study of human papillomavirus and oropharyngeal cancer. *N Engl J Med.* 2007;356(19):1944-1956.
84. Daling JR, Madeleine MM, Johnson LG, et al. Penile cancer: importance of circumcision, human papillomavirus and smoking in in situ and invasive disease. *Int J Cancer.* 2005;116(4):606-616.
85. Gargano JW, Wilkinson EJ, Unger ER, et al. Prevalence of human papillomavirus types in invasive vulvar cancers and vulvar intraepithelial neoplasia 3 in the United States before vaccine introduction. *J Low Genit Tract Dis.* 2012;16(4):471-479.
86. Frisch M, Biggar IJ, Goedert JJ. Human papillomavirus-associated cancers in patients

with human immunodeficiency virus infection and acquired immunodeficiency syndrome. *Journal of the National Cancer Institute.* 2000;92(18):1500-1510.

87. Kaufmann AM, Nieland JD, Jochmus I, et al. Vaccination trial with HPV16 L1E7 chimeric virus-like particles in women suffering from high grade cervical intraepithelial neoplasia (CIN 2/3). *Int J Cancer.* 2007;121(12):2794-2800.
88. de Jong A, van der Burg SH, Kwappenberg KM, et al. Frequent detection of human papillomavirus 16 E2-specific T-helper immunity in healthy subjects. *Cancer Research.* 2002;62(2):472-479.
89. de Jong A, van Poelgeest MI, van der Hulst JM, et al. Human papillomavirus type 16-positive cervical cancer is associated with impaired CD4+ T-cell immunity against early antigens E2 and E6. *Cancer Research.* 2004;64(15):5449-5455.
90. Welters MJ, de Jong A, van den Eeden SJ, et al. Frequent display of human papillomavirus type 16 E6-specific memory t-Helper cells in the healthy population as witness of previous viral encounter. *Cancer Research.* 2003;63(3):636-641.
91. van den Hende M, van Poelgeest MI, van der Hulst JM, et al. Skin reactions to human papillomavirus (HPV) 16 specific antigens intradermally injected in healthy subjects and patients with cervical neoplasia. *Int J Cancer.* 2008;123(1):146-152.
92. Bontkes HJ, de Gruijl TD, van den Muysenberg AJ, et al. Human papillomavirus type 16 E6/E7-specific cytotoxic T lymphocytes in women with cervical neoplasia. *Int J Cancer.* 2000;88(1):92-98.
93. de Vos van Steenwijk PJ, Piersma SJ, Welters MJ, et al. Surgery followed by persistence of high-grade squamous intraepithelial lesions is associated with the induction of a dysfunctional HPV16-specific T-cell response. *Clin Cancer Res.* 2008;14(22):7188-7195.
94. van der Burg SH, Piersma SJ, de Jong A, et al. Association of cervical cancer with the presence of CD4+ regulatory T cells specific for human papillomavirus antigens. *Proc Natl Acad Sci U S A.* 2007;104(29):12087-12092.
95. Palefsky JM, Giuliano AR, Goldstone S, et al. HPV vaccine against anal HPV infection and anal intraepithelial neoplasia. *N Engl J Med.* 2011;365(17):1576-1585.
96. Stokley S, Cohn A, Dorell C, et al. Adolescent vaccination-coverage levels in the United States: 2006-2009. *Pediatrics.* 2011;128(6):1078-1086.
97. Rabkin CS, Biggar RJ, Melbye M, Curtis RE. Second primary cancers following anal and cervical carcinoma: evidence of shared etiologic factors. *Am J Epidemiol.* 1992;136(1):54-58.
98. Ghebeh H, Mohammed S, Al-Omair A, et al. The B7-H1 (PD-L1) T lymphocyte-inhibitory molecule is expressed in breast cancer patients with infiltrating ductal carcinoma: correlation with important high-risk prognostic factors. *Neoplasia.* 2006;8(3):190-198.
99. Cho YA, Yoon HJ, Lee JI, Hong SP, Hong SD. Relationship between the expressions of PD-L1 and tumor-infiltrating lymphocytes in oral squamous cell carcinoma. *Oral Oncol.* 2011;47(12):1148-1153.
100. Lyford-Pike S, Peng S, Young GD, et al. Evidence for a role of the PD-1:PD-L1 pathway in immune resistance of HPV-associated head and neck squamous cell carcinoma. *Cancer Research.* 2013;73(6):1733-1741.
101. Iwai Y, Ishida M, Tanaka Y, Okazaki T, Honjo T, Minato N. Involvement of PD-L1 on tumor cells in the escape from host immune system and tumor immunotherapy by PD-L1 blockade. *Proc Natl Acad Sci U S A.* 2002;99(19):12293-12297.

102. Dong H, Strome SE, Salomao DR, et al. Tumor-associated B7-H1 promotes T-cell apoptosis: a potential mechanism of immune evasion. *Nat Med.* 2002;8(8):793-800.
103. Morris VK, Rashid A, Rodriguez-Bigas MA, et al. Human papillomavirus is associated with unique clinicopathologic features in patients with metastatic squamous cell carcinoma of the anal canal. Paper presented at: Gastrointestinal Cancers Symposium2014; San Francisco, California.
104. Kuo KT, Hsiao CH, Lin CH, Kuo LT, Huang SH, Lin MC. The biomarkers of human papillomavirus infection in tonsillar squamous cell carcinoma-molecular basis and predicting favorable outcome. *Mod Pathol.* 2008;21(4):376-386.
105. Geradts J, Kratzke RA, Niehans GA, Lincoln CE. Immunohistochemical detection of the cyclin-dependent kinase inhibitor 2/multiple tumor suppressor gene 1 (CDKN2/MTS1) product p16INK4A in archival human solid tumors: correlation with retinoblastoma protein expression. *Cancer Research.* 1995;55(24):6006-6011.
106. Kratzke RA, Greatens TM, Rubins JB, et al. Rb and p16INK4a expression in resected non-small cell lung tumors. *Cancer Research.* 1996;56(15):3415-3420.
107. Morris VK, Salem ME, Nimeiri H, et al. Nivolumab for previously treated unresectable metastatic anal cancer (NCI9673): a multicentre, single-arm, phase 2 study. *Lancet Oncol.* 2017;18(4):446-453.
108. Wolchok JD, Chiarion-Sileni V, Gonzalez R, et al. Overall Survival with Combined Nivolumab and Ipilimumab in Advanced Melanoma. *N Engl J Med.* 2017;377(14):1345-1356.
109. Nivolumab Combined With Ipilimumab Versus Sunitinib in Previously Untreated Advanced or Metastatic Renal Cell Carcinoma (CheckMate 214). 2017; (Identification No. NCT02231749). Available at: https://clinicaltrials.gov/ct2/show/record/NCT02231749.
110. Konishi J, Yamazaki K, Azuma M, Kinoshita I, Dosaka-Akita H, Nishimura M. B7-H1 expression on non-small cell lung cancer cells and its relationship with tumor-infiltrating lymphocytes and their PD-1 expression. *Clin Cancer Res.* 2004;10(15):5094-5100.
111. Dong H, Strome SE, Matteson EL, et al. Costimulating aberrant T cell responses by B7-H1 autoantibodies in rheumatoid arthritis. *J Clin Invest.* 2003;111(3):363-370.
112. Galon J, Costes A, Sanchez-Cabo F, et al. Type, density, and location of immune cells within human colorectal tumors predict clinical outcome. *Science.* 2006;313(5795):1960-1964.
113. Grabenbauer GG, Lahmer G, Distel L, Niedobitek G. Tumor-infiltrating cytotoxic T cells but not regulatory T cells predict outcome in anal squamous cell carcinoma. *Clin Cancer Res.* 2006;12(11 Pt 1):3355-3360.
114. Badoual C, Hans S, Rodriguez J, et al. Prognostic value of tumor-infiltrating CD4+ T-cell subpopulations in head and neck cancers. *Clin Cancer Res.* 2006;12(2):465-472.
115. Taube, J.M., R.A. Anders, G.D. Young, et al. (2012). Colocalization of inflammatory response with B7-H1 expression in human melanocytic lesions supports an adaptive resistance mechanism of immune escape. Sci Transl Med. 4:127ra137.
116. Carthon BC, Wolchok JD, Yuan J, et al. Preoperative CTLA-4 blockade: tolerability and immune monitoring in the setting of a presurgical clinical trial. *Clin Cancer Res.* 2010;16(10):2861-2871.
117. Tang MJ, Zhou ZB. Association of the CTLA-4 +49A/G polymorphism with rheumatoid arthritis in Chinese Han population. *Mol Biol Rep.* 2013;40(3):2627-2631.

118. Chen H, Fu T, Suh WK, et al. CD4 T cells require ICOS-mediated PI3K signaling to increase T-Bet expression in the setting of anti-CTLA-4 therapy. *Cancer Immunol Res.* 2014;2(2):167-176.
119. Cooper ZA, Reuben A, Spencer CN, et al. Distinct clinical patterns and immune infiltrates are observed at time of progression on targeted therapy versus immune checkpoint blockade for melanoma. *Oncoimmunology.* 2016;5(3):e1136044.
120. Eisenhauer EA, Therasse P, Bogaerts J, et al. New response evaluation criteria in solid tumours: revised RECIST guideline (version 1.1). *Eur J Cancer.* 2009;45(2):228-247.

APPENDIX A PERFORMANCE STATUS CRITERIA

<table>
<tr><th colspan="2">ECOG Performance Status Scale</th><th colspan="2">Karnofsky Performance Scale</th></tr>
<tr><th>Grade</th><th>Descriptions</th><th>Percent</th><th>Description</th></tr>
<tr><td rowspan="2">0</td><td rowspan="2">Normal activity. Fully active, able to carry on all pre-disease performance without restriction.</td><td>100</td><td>Normal, no complaints, no evidence of disease.</td></tr>
<tr><td>90</td><td>Able to carry on normal activity; minor signs or symptoms of disease.</td></tr>
<tr><td rowspan="2">1</td><td rowspan="2">Symptoms, but ambulatory. Restricted in physically strenuous activity, but ambulatory and able to carry out work of a light or sedentary nature (e.g., light housework, office work).</td><td>80</td><td>Normal activity with effort; some signs or symptoms of disease.</td></tr>
<tr><td>70</td><td>Cares for self, unable to carry on normal activity or to do active work.</td></tr>
<tr><td rowspan="2">2</td><td rowspan="2">In bed <50% of the time. Ambulatory and capable of all self-care, but unable to carry out any work activities. Up and about more than 50% of waking hours.</td><td>60</td><td>Requires occasional assistance, but is able to care for most of his/her needs.</td></tr>
<tr><td>50</td><td>Requires considerable assistance and frequent medical care.</td></tr>
<tr><td rowspan="2">3</td><td rowspan="2">In bed >50% of the time. Capable of only limited self-care, confined to bed or chair more than 50% of waking hours.</td><td>40</td><td>Disabled, requires special care and assistance.</td></tr>
<tr><td>30</td><td>Severely disabled, hospitalization indicated. Death not imminent.</td></tr>
<tr><td rowspan="2">4</td><td rowspan="2">100% bedridden. Completely disabled. Cannot carry on any self-care. Totally confined to bed or chair.</td><td>20</td><td>Very sick, hospitalization indicated. Death not imminent.</td></tr>
<tr><td>10</td><td>Moribund, fatal processes progressing rapidly.</td></tr>
<tr><td>5</td><td>Dead.</td><td>0</td><td>Dead.</td></tr>
</table>

APPENDIX B CTEP MULTICENTER GUIDELINES FOR NON-ETCTN TRIALS

N/A

APPENDIX C BIOASSAY TEMPLATES

No integrated or integral biomarkers will be used for this study.

APPENDIX D MANAGEMENT ALGORITHMS FOR ENDOCRINOPATHY, GASTROINTESTINAL, HEPATIC, NEUROLOGICAL, PULMONARY, RENAL, AND SKIN ADVERSE EVENTS

Please note that the abbreviation “I-O” in the following pages stands for “Immunotherapy-Oncology”

Endocrinopathy Management Algorithm

Rule out non-inflammatory causes. If non-inflammatory cause, treat accordingly and continue immuno-oncology (I-O) therapy.
Consider visual field testing, endocrinology consultation, and imaging.

Asymptomatic thyroid stimulating hormone (TSH) elevation →

- Continue I-O therapy per protocol
- If TSH $<$ 0.5 x lower limit of normal (LLN), or TSH $>$ 2 x ULN, or consistently out of range in 2 subsequent measurements: include free thyroxine (fT4) at subsequent cycles as clinically indicated; consider endocrinology consult

Symptomatic endocrinopathy →

- Evaluate endocrine function
- Consider pituitary scan

Symptomatic with abnormal lab/pituitary scan:
- Delay I-O therapy per protocol
- 1-2 mg/kg/day methylprednisolone IV or by mouth (PO) equivalent
- Initiate appropriate hormone therapy

No abnormal lab/pituitary MRI scan but symptoms persist:
- Repeat labs in 1-3 weeks /MRI in 1 month

→ If improves (with or without hormone replacement):
- Taper steroids over at least 1 month and consider prophylactic antibiotics for opportunistic infections
- Resume I-O therapy per protocol
- Patients with adrenal insufficiency may need to continue steroids with mineralocorticoid component

Suspicion of adrenal crisis (*e.g.* severe dehydration, hypotension, shock out of proportion to current illness →

- Delay or discontinue I-O therapy per protocol
- Rule out sepsis
- Stress dose of IV steroids with mineralocorticoid activity
- IV fluids
- Consult endocrinologist
- If adrenal crisis ruled out, then treat as above for symptomatic endocrinopathy

Patients on IV steroids may be switched to an equivalent dose of oral corticosteroids (e.g. prednisone) at start of tapering or earlier, once sustained clinical improvement is observed. Lower bioavailability of oral corticosteroids should be taken into account when switching to the equivalent dose of oral corticosteroids.

GI Adverse Event Management Algorithm

Rule out non-inflammatory causes. If non-inflammatory cause is identified, treat accordingly and continue I-O therapy. Opiates/narcotics may mask symptoms of perforation. Infliximab should not be used in cases of perforation or sepsis.

Grade of Diarrhea/ Colitis (NCI CTCAE v4)	Management	Follow-up
**Grade 1**<br>Diarrhea:< 4 stools/day over baseline;<br>Colitis: asymptomatic	• Continue I-O therapy per protocol<br>• Symptomatic treatment	• Close monitoring for worsening symptoms.<br>• Educate patient to report worsening immediately<br>If worsens:<br>• Treat as Grade (G) 2 or 3/4
**Grade 2**<br>Diarrhea: 4-6 stools per day over baseline; IV fluids indicated <24 hours (hrs); not interfering with ADL<br>Colitis: abdominal pain; blood in stool	• Delay I-O therapy per protocol<br>• Symptomatic treatment	If improves to grade 1:<br>• Resume I-O therapy per protocol<br>If persists > 5-7 days or recur:<br>• 0.5-1.0 mg/kg/day methylprednisolone or oral equivalent<br>• When symptoms improve to grade 1, taper steroids over at least 1 month, consider prophylactic antibiotics for opportunistic infections, and resume I-O therapy per protocol.<br>If worsens or persists > 3-5 days with oral steroids:<br>• Treat as grade 3/4
**Grade 3-4**<br>Diarrhea (G3): ≥7 stools per day over baseline; incontinence; IV fluids ≥24 hrs; interfering with activities of daily living (ADL)<br>Colitis (G3): severe abdominal pain, medical intervention indicated, peritoneal signs<br>G4: life-threatening, perforation	• Discontinue I-O therapy per protocol<br>• 1.0 to 2.0 mg/kg/day methylprednisolone IV or IV equivalent<br>• Add prophylactic antibiotics for opportunistic infections<br>• Consider lower endoscopy	If improves:<br>• Continue steroids until grade 1, then taper over at least 1 month<br>If persists > 3-5 days, or recurs after improvement:<br>• Add infliximab 5 mg/kg (if no contraindication). Note: Infliximab should not be used in cases of perforation or sepsis

Patients on IV steroids may be switched to an equivalent dose of oral corticosteroids (e.g. prednisone) at start of tapering or earlier, once sustained clinical improvement is observed. Lower bioavailability of oral corticosteroids should be taken into account when switching to the equivalent dose of oral corticosteroids.

Hepatic Adverse Event Management Algorithm

Rule out non-inflammatory causes. If non-inflammatory cause, treat accordingly and continue I-O therapy. Consider imaging for obstruction.

**Grade of Liver Test Elevation** (NCI CTCAE v4)	**Management**	**Follow-up**
**Grade 1**<br>AST or ALT > ULN to 3.0 x ULN and/or Total bilirubin (T. bili) > ULN - 1.5 x ULN	• Continue I-O therapy per protocol	• Continue liver function tests (LFT) monitoring per protocol<br>If worsens:<br>• Treat as Grade 2 or 3-4
**Grade 2**<br>AST or ALT > 3.0 to ≤ 5 x ULN and/or T. bili > 1.5 to ≤ 3 x ULN	• Delay I-O therapy per protocol<br>• Increase frequency of monitoring to every 3 days	If returns to baseline:<br>•Resume routine monitoring, resume I-O therapy per protocol<br><br>If elevations persist > 5-7 days or worsen :<br>• 0.5-1 mg/kg/day methylprednisolone or oral equivalent and when LFT returns to grade 1 or baseline, taper steroids over at least 1 month, consider prophylactic antibiotics for opportunistic infections, and resume I-O therapy per protocol
**Grade 3-4**<br>AST or ALT > 5 x ULN and /or T.bili >3 x ULN	• Discontinue I-O therapy*<br>• Increase frequency of monitoring to every 1-2 days<br>• 1.0 to 2.0 mg/kg/day methylprednisolone IV or IV equivalent**<br>• Add prophylactic antibiotics for opportunistic infections<br>• Consult gastroenterologist	If returns to grade 2:<br>• Taper steroids over at least 1 month<br><br>If does not improve in >3-5 days, worsens or rebounds:<br>• Add mycophenolate mofetil 1 gram (g) twice daily (BID)<br>• If no response within an additional 3-5 days, consider other immunosuppressants per local guidelines

Patients on IV steroids may be switched to an equivalent dose of oral corticosteroids (e.g. prednisone) at start of tapering or earlier, once sustained clinical improvement is observed. Lower bioavailability of oral corticosteroids should be taken into account when switching to the equivalent dose of oral corticosteroids.

***I-O therapy may be delayed rather than discontinued if AST/ALT ≤ 8 x ULN and T.bili ≤ 5 x ULN.**

**The recommended starting dose for grade 4 hepatitis is 2 mg/kg/day methylprednisolone IV.

Neurological Adverse Event Management Algorithm

Rule out non-inflammatory causes. If non-inflammatory cause, treat accordingly and continue I-O therapy.

Grade of Neurological Toxicity (NCI CTCAE v4)	Management	Follow-up
**Grade 1*** Asymptomatic or mild symptoms; Intervention not indicated	• Continue I-O therapy per protocol	Continue to monitor the patient.<br>If worsens:<br>• Treat as Grade 2 or 3-4
**Grade 2*** Moderate symptoms; Limiting instrumental ADL	• Delay I-O therapy per protocol<br>• Treat symptoms per local guidelines<br>• Consider 0.5 to 1.0 mg/kg/day methylprednisolone IV or PO equivalent	If improves to baseline:<br>•Resume I-O therapy per protocol when improved to baseline<br>If worsens:<br>• Treat as Grade 3-4
**Grade 3-4** Severe symptoms; Limiting self-care ADL; Life-threatening	• Discontinue I-O therapy per protocol<br>• Obtain neurology consult<br>• Treat symptoms per local guidelines<br>• 1.0-2.0 mg/kg/day IV methylprednisolone IV or IV equivalent<br>• Add prophylactic antibiotics for opportunistic infections	If improves to Grade 2:<br>• Taper steroids over at least 1 month<br>If worsens or atypical presentation:<br>• Consider IVIG or other immunosuppressive therapies per local guidelines

Patients on IV steroids may be switched to an equivalent dose of oral corticosteroids (e.g. prednisone) at start of tapering or earlier, once sustained clinical improvement is observed. Lower bioavailability of oral corticosteroids should be taken into account when switching to the equivalent dose of oral corticosteroids.

*Patients with any CNS events including aseptic meningitis, encephalitis, symptomatic hypophysitis, or myopathy, peripheral demyelinating neuropathy, cranial neuropathy (other than peripheral n. VII), GB syndrome, or myasthenia gravis should be off study.

Pulmonary Adverse Event Management Algorithm

Rule out non-inflammatory causes. If non-inflammatory cause, treat accordingly and continue I-O therapy. Evaluate with imaging and pulmonary consultation.

**Grade of Pneumonitis** (NCI CTCAE v4)	**Management**	**Follow-up**
**Grade 1**<br>Radiographic changes only	• Consider delay of I-O therapy<br>• Monitor for symptoms every 2-3 days<br>• Consider Pulmonary and Infectious Disease (ID) consults	• Re-image at least every 3 weeks<br><u>If worsens:</u><br>• Treat as Grade 2 or 3-4
**Grade 2**<br>Mild to moderate new symptoms	• Delay I-O therapy per protocol<br>• Pulmonary and ID consults<br>• Monitor symptoms daily, consider hospitalization<br>• 1.0 mg/kg/day methyl-prednisolone IV or oral equivalent<br>• Consider bronchoscopy , lung biopsy	• Re-image every 1-3 days<br><u>If improves</u>:<br>• When symptoms return to near baseline, taper steroids over at least 1 month and then resume I-O therapy per protocol and consider prophylactic antibiotics<br><u>If not improving after 2 weeks or worsening:</u><br>• Treat as Grade 3-4
**Grade 3-4**<br>Severe new symptoms; New/worsening hypoxia; Life-threatening	• Discontinue I-O therapy per protocol<br>• Hospitalize<br>• Pulmonary and ID consults<br>• 2-4 mg/kg/day methylprednisolone IV or IV equivalent<br>• Add prophylactic antibiotics for opportunistic infections<br>• Consider bronchoscopy, lung biopsy	<u>If improves to baseline:</u><br>• Taper steroids over at least 6 weeks<br><u>If not improving after 48 hours or worsening:</u><br>• Add additional immunosuppression (e.g. infliximab, cyclophosphamide, intravenous immunoglobulin (IVIG), or mycophenolate mofetil)

Patients on IV steroids may be switched to an equivalent dose of oral corticosteroids (e.g. prednisone) at start of tapering or earlier, once sustained clinical improvement is observed. Lower bioavailability of oral corticosteroids should be taken into account when switching to the equivalent dose of oral corticosteroids.

Renal Adverse Event Management Algorithm

Rule out non-inflammatory causes. If non-inflammatory cause, treat accordingly and continue I-O therapy

Grade of Creatinine Elevation (NCI CTCAE v4)	Management	Follow-up
**Grade 1**<br>Creatinine > upper limit of normal (ULN) and > than baseline but ≤ 1.5x baseline	• Continue I-O therapy per protocol<br>• Monitor creatinine weekly	<u>If returns to baseline</u>:<br>•Resume routine creatinine monitoring per protocol<br><u>If worsens:</u><br>•Treat as Grade 2 or 3/4
**Grade 2-3**<br>Creatinine > 1.5x baseline to ≤ 6x ULN	• Delay I-O therapy per protocol<br>• Monitor creatinine every 2-3 days<br>• 0.5 to 1.0 mg/kg/day methylprednisolone IV or oral equivalent<br>• Consider renal biopsy	<u>If returns to Grade 1:</u><br>•Taper steroids over at least 1 month, consider prophylactic antibiotics for opportunistic infections, and resume I-O therapy and routine creatinine monitoring per protocol<br><br><u>If elevations persist > 7 days or worsen:</u><br>•Treat as Grade 4
**Grade 4**<br>Creatinine > 6x ULN	• Discontinue I-O therapy per protocol<br>• Monitor creatinine daily<br>• 1.0-2.0 mg/kg/day methylprednisolone IV or IV equivalent<br>• Consult nephrologist<br>• Consider renal biopsy	<u>If returns to Grade 1</u>: Taper steroids over at least 1 month and add prophylactic antibiotics for opportunistic infections

Patients on IV steroids may be switched to an equivalent dose of oral corticosteroids (e.g. prednisone) at start of tapering or earlier, once sustained clinical improvement is observed. Lower bioavailability of oral corticosteroids should be taken into account when switching to the equivalent dose of oral corticosteroids.

Skin Adverse Event Management Algorithm

Rule out non-inflammatory causes. If non-inflammatory cause, treat accordingly and continue I-O therapy.

Grade of Rash (NCI CTCAE v4)	Management	Follow-up
**Grade 1-2** Covering ≤ 30% body surface area (BSA)*	• Symptomatic therapy (e.g. antihistamines, topical steroids)<br>• Continue I-O therapy per protocol	<u>If persists > 1-2 weeks or recurs:</u><br>• Consider skin biopsy<br>• Delay I-O therapy per protocol<br>• Consider 0.5-1.0 mg/kg/day methylprednisolone IV or oral equivalent. Once improving , taper steroids over at least 1 month, consider prophylactic antibiotics for opportunistic infections, and resume I-O therapy per protocol<br><u>If worsens:</u><br>• Treat as Grade 3-4
**Grade 3** Covering >30% BSA; Life threatening consequences*	• Delay or discontinue I-O therapy per protocol<br>• Consider skin biopsy<br>• Dermatology consult<br>• 1.0-2.0 mg/kg/day IV methylprednisolone IV or IV equivalent	<u>If improves to Grade 1:</u><br>• Taper steroids over at least 1 month and add prophylactic antibiotics for opportunistic infections<br>• Resume I-O therapy per protocol

GRADE 4 ADVERSE EVENT: I-O therapy should be discontinued and patient should come off study.

Patients on IV steroids may be switched to an equivalent dose of oral corticosteroids (e.g. prednisone) at start of tapering or earlier, once sustained clinical improvement is observed. Lower bioavailability of oral corticosteroids should be taken into account when switching to the equivalent dose of oral corticosteroids.
*Refer to NCI CTCAE v4 for term-specific grading criteria.

APPENDIX E ANALYSES OF SAMPLES FOR EXPLORATORY STUDIES

Fresh frozen tumor samples, if available, will be analyzed by RT-PCR to assess for mRNA expression of genes that may contribute to anti-tumor immune responses, e.g. IFN-γ and other cytokines. Tumor tissues will also be analyzed by immunohistochemistry (IHC) for markers that may include, but are not limited to, CD4, CD8, FoxP3, and Granzyme.

Fresh tumor samples, if available and assessed by physician adequate for selection, from routine surgical procedures, will be used for flow cytometry analysis of tumor infiltrating lymphocytes (TILs) when possible.

Lymphocytes from collected blood or from tumor infiltrating cells will be analyzed using 17 core panels by flow cytometry to identify sub-populations of T-cells (for example, CD4+ helper T-cells, CD8+ cytotoxic T-cells, CD4+, FoxP3+ regulatory T-cells). These studies require 20 cc of blood per blood draw.

Lymphocytes from collected blood will be analyzed by ELISPOT analyses, if possible, to identify T-cells that are functionally reactive against a HPV-specific antigens E6 and E7. For example, in an ELISPOT assay that uses HPV as the target antigen, a patient's T-cell will be analyzed to determine if a cytokine, such as interferon- gamma, is produced by the T-cell, in response to recognition of the HPV antigen in the context of an antigen-presenting cell. These studies require a minimum of 80 cc of blood per blood draw.

Funding for these tests will be provided by MD Anderson Cancer Center institutional sources and philanthropic donations.

APPENDIX F PARTICIPANT WALLET CARD

INFORMATION FOR PATIENTS AND PRESCRIBERS

You are enrolled on a clinical trial using experimental agents nivolumab with or without ipilimumab. This clinical trial is sponsored by the NCI. It is very important to:

- Take this card with you to the emergency room or any healthcare provider other than your study doctor. Tell all healthcare providers that you are being treated with nivolumab with or without ipilimumab and SHOW THEM THIS CARD. Tell your doctors if you stop taking regular medicine or if you start taking a new medicine. Do not take over-the-counter medications, dietary supplements, or prescription medications without the approval of your study doctor.

- Tell all of your prescribers (doctor, physicians' assistant, nurse practitioner, pharmacist) that you are taking part in a clinical trial.

- See your study doctor for help managing symptoms. Doctors who are not familiar with nivolumab or ipilimumab may not be aware of the appropriate management or side effects.

- If you have any of these signs or symptoms, call your study doctor or nurse right away: new or worsening shortness of breath, cough, or wheezing; diarrhea or any increase in the amount or number of bowel movements above normal, blood in stool or dark, tarry, sticky stools; stomach pain/cramps; yellowing of the white part of the eye(s); blurry vision, double vision, or other vision problems; eye pain or redness; severe rash, itching, or peeling; yellowing of the skin; fever; decreased strength or energy; dizziness; headaches; muscle weakness; numbness or tingling in the hands or feet; changes in behavior; dark urine; palpitations, or fast or irregular heartbeats.

- Before prescribing new medicines, your regular prescribers should contact your study doctor.

- Your study doctor's name is ______________________________

  and can be contacted at _________________________________.
